# Global, Regional, and National Burden of Myocarditis and Cardiomyopathy, 1990–2017

**DOI:** 10.3389/fcvm.2021.610989

**Published:** 2021-02-11

**Authors:** Haijiang Dai, Dor Lotan, Arsalan Abu Much, Arwa Younis, Yao Lu, Nicola Luigi Bragazzi, Jianhong Wu

**Affiliations:** ^1^Department of Cardiology, The Third Xiangya Hospital, Central South University, Changsha, China; ^2^Centre for Disease Modelling, Department of Mathematics and Statistics, York University, Toronto, ON, Canada; ^3^Leviev Heart Center, Sheba Medical Center, Ramat Gan, Israel; ^4^Sackler Faculty of Medicine, Tel-Aviv University, Tel-Aviv, Israel; ^5^Clinical Cardiovascular Research Center, University of Rochester Medical Center, Rochester, NY, United States

**Keywords:** myocarditis, cardiomyopathy, global burden of disease, prevalence, mortality

## Abstract

**Objective:** To estimate the burden of myocarditis (MC), alcoholic cardiomyopathy (AC), and other cardiomyopathy (OC) for 195 countries and territories from 1990 to 2017.

**Methods:** We collected detailed information on MC, AC, and OC between 1990 and 2017 from the Global Burden of Disease study 2017, which was designed to provide a systematic assessment of health loss due to diseases and injuries in 21 regions, covering 195 countries and territories. Estimates of MC, AC, and OC burden were produced using a standard Cause of Death Ensemble model and a Bayesian mixed-effects meta-regression tool, and included prevalence, deaths, years lived with disability (YLDs), and years of life lost (YLLs). All estimates were presented as counts, age-standardized rates per 100,000 people and percentage change, with 95% uncertainty intervals (UIs).

**Results:** Worldwide, there were 1.80 million (95% UI 1.64–1.98) cases of MC, 1.62 million (95% UI 1.37–1.90) cases of AC and 4.21 million (95% UI 3.63–4.87) cases of OC, contributing to 46,486 (95% UI 39,709–51,824), 88,890 (95% UI 80,935–96,290), and 233,159 (95% UI 213,677–248,289) deaths in 2017, respectively. Furthermore, globally, there were 131,376 (95% UI 90,113–183,001) YLDs and 1.26 million (95% UI 1.10–1.42) YLLs attributable to MC, 139,087 (95% UI 95,134–196,130) YLDs and 2.84 million (95% UI 2.60–3.07) YLLs attributable to AC, and 353,325 (95% UI 237,907–493,908) YLDs and 5.51 million (95% UI 4.95–5.99) YLLs attributable to OC in 2017. At the national level, the age-standardized prevalence rates varied by 10.4 times for MC, 252.6 times for AC and 38.1 times for OC; the age-standardized death rates varied by 43.9 times for MC, 531.0 times for AC and 43.3 times for OC; the age-standardized YLD rates varied by 12.4 times for MC, 223.7 times for AC, and 34.1 times for OC; and the age-standardized YLL rates varied by 38.4 times for MC, 684.8 times for AC, and 36.2 times for OC. Between 1990 and 2017, despite the decreases in age-standardized rates, the global numbers of prevalent cases, deaths, YLDs, and YLLs have increased for all the diseases.

**Conclusion:** Accurate assessment of the burden of MC, AC, and OC is essential for formulating effective preventative prevention and treatment programs and optimizing health system resource allocation. Our results suggest that MC, AC, and OC remain important global public health problems with increasing numbers of prevalent cases, deaths, YLDs, and YLLs over the past decades, and there are significant geographic variations in the burden of these diseases. Further research is warranted to expand our knowledge of potential risk factors and to improve the prevention, early detection and treatment of these diseases.

## Introduction

Cardiovascular disease (CVD) is the largest single contributor to global mortality and morbidity, imposing a severe burden in terms of lost productivity and disability in adults (1). Among CVD, myocarditis (MC) represents an often-underdiagnosed cause of several life-threatening conditions, including acute heart failure, dilated cardiomyopathy, and sudden death (2, 3). The incidence and specific causes of MC vary considerably across the globe, depending on the geographical area and the selected population (4, 5). In developed settings, MC is generally caused by viral pathogens, whereas in developing and resource-limited contexts, MC is mainly due to rheumatic carditis and infectious agents like *Trypanosoma cruzi* and diphtheria (6). Together with MC, cardiomyopathy is another often fatal disease contributing to about half of patients dying suddenly in childhood or adolescence or needing to undergo cardiac transplantation (7). Several types of cardiomyopathy exist, with new ones having been discovered and added recently and MC having been reclassified by some scholars as “inflammatory cardiomyopathy” (7, 8).

A thorough knowledge of the different epidemiological characteristics of MC and cardiomyopathy among various countries and populations can help public health decision- and policy-makers develop and implement targeted programs aimed at the prevention and management of the diseases. However, reliable and accurate epidemiological data regarding MC and cardiomyopathy are predominantly available from developed countries which apply well-established and consolidated diagnostic evaluations and criteria (7). The Global Burden of Disease study (GBD) is a continuous effort to evaluate the burden of more than 350 diseases and injuries for 195 countries and territories across the world and provides a unique opportunity to understand the landscape of MC and cardiomyopathy. Since 2016, cardiomyopathy was divided into two types in the GBD study, including alcoholic cardiomyopathy (AC) and other cardiomyopathy (OC) (9). In the current study, we aimed to provide the estimates of prevalence, deaths, years lived with disability (YLDs), and years of life lost (YLLs) for MC, AC, and OC for 195 countries and territories from 1990 to 2017, based on the most recent GBD 2017.

## Methods

### Overview

This study is part of GBD 2017, which was designed to provide a systematic assessment of health loss due to diseases and injuries at the global, regional, and national levels. In the latest iteration, GBD 2017, seven super-regions, 21 regions, and 195 countries and territories were included; and estimates for 359 diseases and injuries, 282 causes of death, and 84 risk factors were reported. The detailed methodology used for the estimation process can be found in previous GBD 2017 papers (10–13). Because GBD 2017 used de-identified, aggregated data, a waiver of informed consent was reviewed and approved by the University of Washington Institutional Review Board. All results and the original data sources used to produce estimates were publicly available on the website of Institute for Health Metrics and Evaluation (IHME), and can be accessed at http://ghdx.healthdata.org/.

### Case Definitions

MC, AC and OC were ascertained using the International Classification of Diseases version 9 (ICD-9) and version 10 (ICD-10) codes. Diseases coded as 422–422.9 in ICD-9 or B33.2, I40–I41.9, and I51.4 in ICD-10 were identified as MC; diseases coded as 425.5 in ICD-9 or I42.6 in ICD-10 were identified as AC; and diseases coded as 425.0–425.3, 425.7–425.8, and 429.0 in ICD-9 or I42.1–I42.5, I42.7–I42.8, and I43–I43.9 in ICD-10 were identified as OC (11).

### Estimation of Deaths

Death estimates were derived from vital registration data sources for MC and OC, and from vital registration and verbal autopsy data sources for AC. The ICD codes listed above were used to identify the diseases from the data sources. Data points that were implausibly high and discontinuous with the rest of the time series, or unable to be disaggregated appropriately were treated as outliers. Deaths due to MC, AC, and OC were estimated using standard Cause of Death Ensemble model (CODEm), a highly systematized tool that runs many different models using location-level predictive covariates and chooses an ensemble of models that best reflects all the available input data (12, 14). Predictive covariates incorporated into each cause-specific model can be found in the Supplementary Table 1. To ensure consistency with all-cause mortality estimates, CoDCorrect algorithm was used to adjust the cause-specific results of MC, AC and OC for each age-sex-year-location group.

### Non-fatal Estimation

The prevalence of MC, AC, and OC was estimated using DisMod-MR 2.1, a Bayesian mixed-effects meta-regression tool developed for GBD analyses (11). Cause-specific incidence, remission, and mortality rates were integrated to produce consistent estimates of prevalence for all locations. Apart from the hospital and claims data, the prevalence estimates didn't include any non-literature-based data. All outpatient data were excluded as they were implausibly low when compared with inpatient data from the same locations and claims data. For high-income North America, Central Europe, and Western Europe, any inpatient hospital data that were more than 2-fold higher or 0.5-fold lower than the median absolute deviation value were also excluded for that age-sex group. More information on the prevalence estimation can be found elsewhere (11).

YLDs were calculated by multiplying the prevalence (in number of cases) of each disease by the disability weight that quantifies the magnitude of heath loss associated with disease (11). Disability weight was measured on a scale from 0 to 1, where 0 represents full health and 1 is equivalent to death. Detailed explanation of the estimation process of disability weight has been described previously (11).

### Socio-Demographic Index

Socio-demographic Index (SDI) was a composite variable used to quantity the development level for each location-year (10–13). It ranged from 0 (less developed) to 1 (most developed), and was calculated based on lag distributed income per capita, mean education in the population aged 15 years or older, and total fertility rate under 25 years. One hundred ninety five countries and territories in GBD 2017 were divided into five groups by SDI quintile: low SDI, low-middle SDI, middle SDI, high-middle SDI, and high SDI (11).

### Compilation of Results

YLLs were calculated by multiplying the number of deaths in each age group with a standard life expectancy at that age (12). The standard life expectancy was based on the lowest observed mortality risk for each age group in all populations over 5 million (12). Age-standardized rates were computed by direct standardization using the global age structure. Uncertainty was propagated throughout the modeling process by sampling 1000 draws at each step of the calculations. Ninety-five percent uncertainty intervals (UIs) were determined as the 2.5th and 97.5th percentiles of the ordered 1,000 draws (10–13). Any report on significant differences was based on a 95% UI excluding zero.

## Results

### Prevalence in 2017

**Figure 1:**
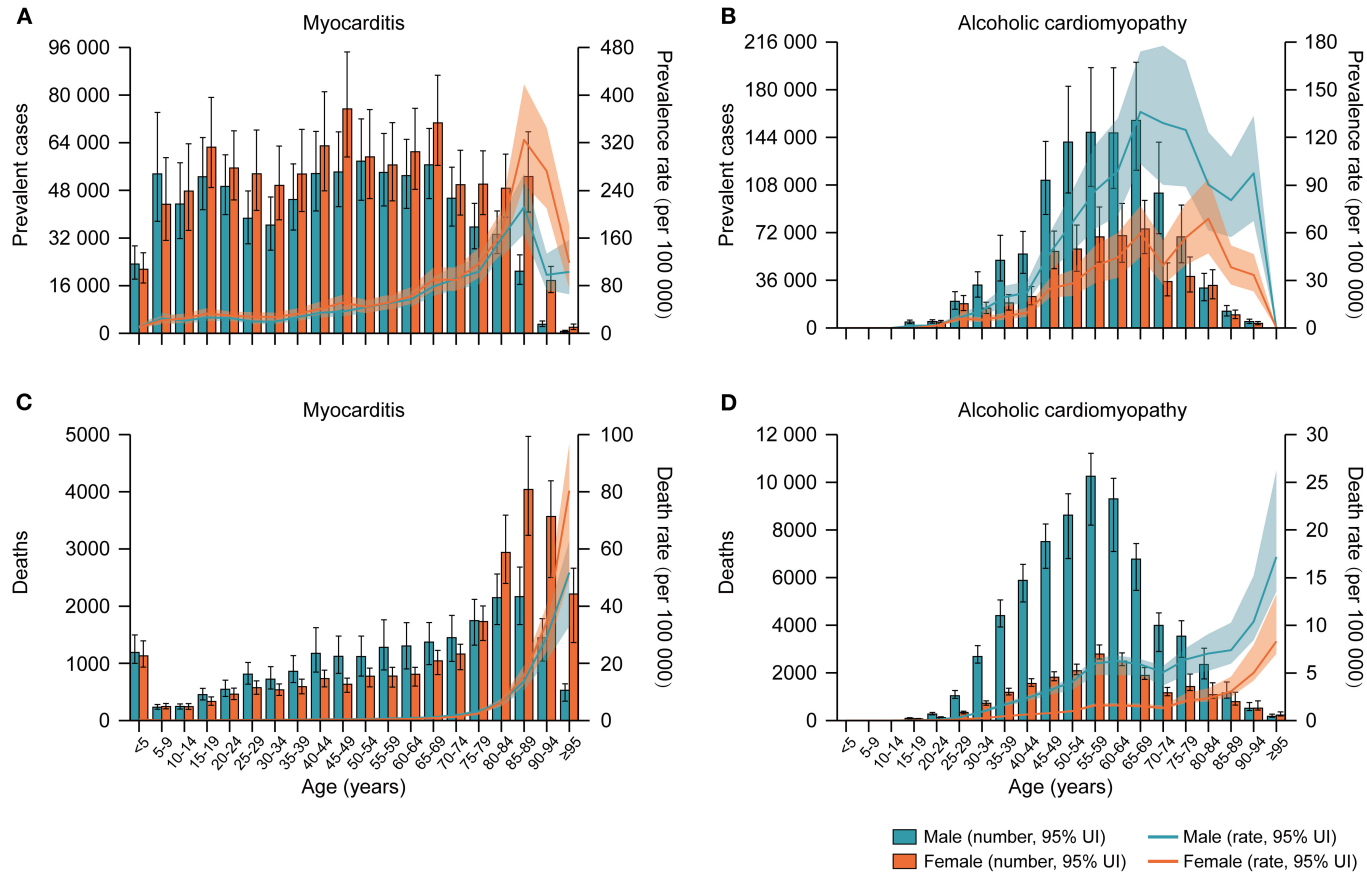
Age-specific numbers and rates of prevalent cases and deaths for myocarditis and alcoholic cardiomyopathy by sex, 2017. **(A)** Age-specific numbers and rates of myocarditis prevalent cases; **(B)** Age-specific numbers and rates of alcoholic cardiomyopathy prevalent cases; **(C)** Age-specific numbers and rates of myocarditis deaths; **(D)** Age-specific numbers and rates of alcoholic cardiomyopathy deaths.

Worldwide, there were 1.80 million (95% UI 1.64–1.98) cases of MC, 1.62 million (95% UI 1.37–1.90) cases of AC and 4.21 million (95% UI 3.63–4.87) cases of OC in 2017. The age-standardized prevalence rates of MC, AC, and OC per 100,000 people in 2017 were 23.2 (95% UI 21.0–25.5), 19.9 (95% UI 16.8–23.3), and 53.9 (95% UI 46.7–62.1), respectively (Table 1). By sex, the age-standardized prevalence rate of AC was significantly higher in males than females [27.7 (95% UI 23.4–32.4) vs. 12.6 (95% UI 10.6–14.7) per 100,000 people], whereas the age-standardized prevalence rates of MC and OC were similar between males and females (Table 1). Figure 1 and Supplementary Figure 1 showed the age-specific numbers and rates of prevalent cases by sex in 2017. In addition to extreme age groups, the numbers of MC prevalent cases were stable across different age groups in both sexes. The numbers of AC prevalent cases peaked at the ages of 65–69 years in both sexes, whereas the numbers of OC prevalent cases peaked at the ages of 65–69 years in males and 80–84 years in females.

**Table 1:** Age-standardized prevalence, death, YLD, and YLL rates of myocarditis, alcoholic cardiomyopathy, and other cardiomyopathy, and their percentage changes from 1990 to 2017, by sex and SDI quintile.

	**Prevalence**		**Deaths**		**YLDs**		**YLLs**	
	**2017 age-standardized rate per 100,000 people**	**Percentage change in age-standardized rates, 1990–2017**	**2017 age-standardized rate per 100,000 people**	**Percentage change in age-standardized rates, 1990–2017**	**2017 age-standardized rate per 100,000 people**	**Percentage change in age-standardized rates, 1990–2017**	**2017 age-standardized rate per 100,000 people**	**Percentage change in age-standardized rates, 1990–2017**
**MYOCARDITIS**								
Global	23.2 (21.0 to 25.5)	−6.9% (−7.8 to −5.9)	0.6 (0.5 to 0.7)	−6.2% (−15.0 to 3.4)	1.7 (1.2 to 2.4)	−7.2% (−8.6 to −5.9)	16.6 (14.5 to 18.5)	−23.5% (−32.6 to −11.4)
**Sex**								
Male	21.3 (19.4 to 23.4)	−4.7% (−5.6 to −3.7)	0.7 (0.5 to 0.8)	6.6% (−8.5 to 26.0)	1.5 (1.0 to 2.1)	−4.7% (−6.1 to −3.2)	18.3 (15.2 to 22.1)	−9.7% (−25.3 to 17.2)
Female	24.9 (22.4 to 27.4)	−8.1% (−9.2 to −6.9)	0.6 (0.5 to 0.6)	−15.6% (−24.4 to −0.3)	1.9 (1.3 to 2.6)	−8.6% (−10.3 to −6.8)	14.8 (12.7 to 16.7)	−35.5% (−44.9 to −22.8)
**SDI quintile**								
Low SDI	17.3 (15.8 to 19.0)	1.6% (0.5 to 2.7)	0.5 (0.3 to 0.6)	−16.6% (−30.7 to 2.7)	1.2 (0.8 to 1.6)	2.7% (0.2 to 5.1)	14.6 (10.2 to 19.1)	−30.1% (−43.7 to −10.3)
Low-middle SDI	19.6 (17.8 to 21.5)	3.3% (2.2 to 4.3)	0.6 (0.5 to 0.8)	−1.3% (−18.5 to 17.8)	1.4 (0.9 to 1.9)	4.3% (2.0 to 6.5)	18.3 (14.7 to 22.5)	−22.0% (−36.6 to −2.6)
Middle SDI	25.6 (23.0 to 28.3)	3.1% (2.0 to 4.2)	0.8 (0.6 to 0.9)	17.6% (3.8 to 33.5)	1.9 (1.3 to 2.6)	4.4% (2.1 to 6.8)	20.0 (16.8 to 23.5)	−19.8% (−30.6 to −5.0)
High-middle SDI	25.7 (23.1 to 28.4)	1.2% (−0.3 to 2.8)	0.7 (0.6 to 0.8)	−14.2% (−26.9 to 7.0)	1.9 (1.3 to 2.6)	4.0% (0.9 to 7.4)	17.0 (15.8 to 19.5)	−19.1% (−29.7 to −0.7)
High SDI	27.1 (24.7 to 29.5)	−17.8% (−19.6 to −15.6)	0.5 (0.4 to 0.5)	−13.1% (−36.9 to −1.5)	2.1 (1.4 to 2.9)	−18.9% (−21.5 to −16.2)	12.3 (10.1 to 13.2)	−14.8% (−35.6 to −5.1)
**ALCOHOLIC CARDIOMYOPATHY**								
Global	19.9 (16.8 to 23.3)	−26.2% (−28.2 to −24.0)	1.1 (1.0 to 1.2)	−19.6% (−34.0 to −11.3)	1.7 (1.2 to 2.4)	−25.7% (−28.0 to −23.2)	34.7 (31.7 to 37.5)	−7.6% (−20.7 to 3.2)
**Sex**								
Male	27.7 (23.4 to 32.4)	−22.2% (−24.6 to −19.4)	1.7 (1.5 to 1.9)	−6.2% (−18.7 to 6.6)	2.4 (1.6 to 3.3)	−21.8% (−24.6 to −18.6)	55.2 (47.0 to 60.4)	3.3% (−14.4 to 17.4)
Female	12.6 (10.6 to 14.7)	−33.6% (−35.4 to −31.6)	0.5 (0.4 to 0.6)	−45.7% (−53.6 to −40.6)	1.1 (0.7 to 1.5)	−32.8% (−35.2 to −30.1)	14.8 (13.6 to 16.9)	−32.8% (−36.7 to −26.5)
**SDI quintile**								
Low SDI	11.8 (9.6 to 14.3)	3.6% (1.0 to 6.2)	0.4 (0.3 to 0.7)	−28.9% (−48.8 to −0.4)	1.0 (0.7 to 1.5)	4.2% (0.1 to 8.6)	12.0 (7.8 to 17.8)	−31.5% (−49.8 to −5.1)
Low-middle SDI	9.4 (7.8 to 11.3)	5.8% (2.6 to 9.0)	0.4 (0.3 to 0.5)	−22.3% (−41.3 to 1.9)	0.8 (0.6 to 1.2)	6.1% (0.4 to 12.1)	11.5 (9.4 to 14.4)	−21.9% (−40.2 to 1.2)
Middle SDI	7.1 (5.9 to 8.4)	13.5% (11.5 to 15.5)	0.3 (0.3 to 0.4)	8.9% (−14.7 to 33.2)	0.6 (0.4 to 0.9)	13.5% (10.7 to 16.4)	9.1 (7.4 to 11.1)	12.3% (−11.5 to 34.0)
High-middle SDI	27.2 (22.8 to 31.9)	−32.0% (−33.5 to −30.4)	3.1 (2.9 to 3.5)	5.9% (−14.5 to 31.2)	2.3 (1.6 to 3.3)	−31.5% (−33.6 to −29.2)	108.4 (101.1 to 120.6)	19.6% (−3.8 to 52.9)
High SDI	40.0 (34.5 to 46.1)	−17.7% (−21.7 to −12.9)	0.8 (0.7 to 0.9)	−45.4% (−58.2 to −37.5)	3.4 (2.3 to 4.7)	−17.7% (−22.5 to −12.5)	22.8 (18.7 to 24.7)	−41.8% (−52.7 to −35.3)
**OTHER CARDIOMYOPATHY**								
Global	53.9 (46.7 to 62.1)	−15.5% (−18.4 to −12.7)	3.1 (2.8 to 3.3)	−30.7% (−35.2 to −17.8)	4.5 (3.1 to 6.3)	−15.0% (−17.8 to −12.1)	71.1 (64.0 to 77.0)	−20.8% (−26.6 to −12.2)
**Sex**								
Male	53.1 (45.9 to 61.2)	−9.5% (−12.8 to −6.1)	3.6 (3.1 to 3.9)	−22.9% (−30.5 to 7.8)	4.5 (3.0 to 6.2)	−9.3% (−12.6 to −5.8)	86.9 (77.0 to 95.1)	−11.9% (−21.0 to 3.5)
Female	54.5 (47.1 to 62.6)	−18.7% (−21.6 to −15.9)	2.6 (2.4 to 2.8)	−37.5% (−40.8 to −30.9)	4.6 (3.1 to 6.3)	−18.1% (−21.1 to −15.1)	55.7 (49.4 to 60.8)	−31.1% (−36.0 to −23.2)
**SDI quintile**								
Low SDI	48.6 (40.5 to 57.6)	4.9% (2.2 to 7.5)	3.0 (2.3 to 4.0)	−4.0% (−16.0 to 13.8)	4.1 (2.7 to 5.8)	5.6% (1.6 to 9.6)	80.2 (61.5 to 105.9)	−15.5% (−28.0 to 0.6)
Low-middle SDI	42.1 (35.5 to 49.5)	7.6% (4.8 to 10.1)	3.0 (2.6 to 3.4)	4.0% (−10.6 to 23.0)	3.5 (2.4 to 5.0)	8.2% (4.4 to 11.9)	76.3 (67.9 to 86.7)	−4.8% (−17.6 to 10.7)
Middle SDI	32.2 (27.5 to 37.4)	15.6% (13.8 to 17.6)	2.1 (1.8 to 2.2)	10.7% (0.9 to 25.5)	2.7 (1.9 to 3.8)	16.0% (13.7 to 18.5)	50.6 (44.7 to 54.6)	−2.9% (−13.3 to 7.9)
High-middle SDI	38.3 (33.0 to 44.3)	−3.6% (−6.2 to −0.8)	3.7 (3.1 to 3.9)	7.1% (−4.9 to 15.7)	3.2 (2.2 to 4.5)	−2.5% (−5.7 to 0.7)	88.0 (72.1 to 93.7)	17.4% (−3.5 to 30.0)
High SDI	97.8 (85.8 to 111.0)	−18.6% (−22.4 to −14.3)	3.2 (3.0 to 3.4)	−51.0% (−55.0 to −33.2)	8.2 (5.6 to 11.2)	−18.5% (−22.3 to −14.0)	64.1 (61.2 to 74.7)	−45.2% (−49.5 to −26.4)

*Data in parentheses are 95% uncertainty intervals. YLDs, years lived with disability; YLLs, years of life lost; SDI, Socio-demographic Index*.

**Figure 2:**
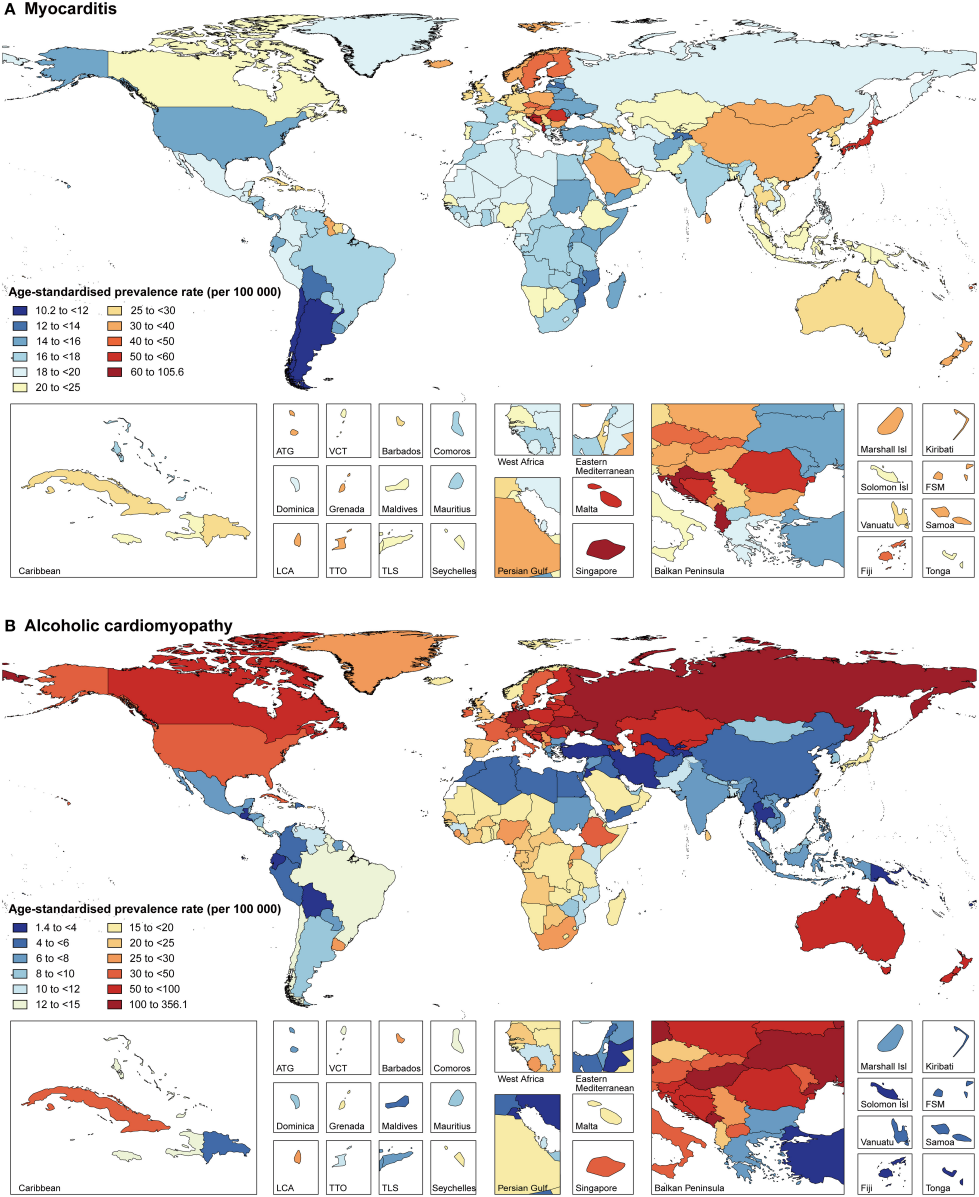
Age-standardized prevalence rates of myocarditis **(A)** and alcoholic cardiomyopathy **(B)** for 195 countries and territories, both sexes, 2017. ATG, Antigua and Barbuda; Isl, Islands; FSM, Federated States of Micronesia; LCA, Saint Lucia; TLS, Timor-Leste; TTO, Trinidad and Tobago; VCT, Saint Vincent and the Grenadines.

The prevalence of MC, AC, and OC in 2017 varied widely across geographic locations (Figure 2, Supplementary Figure 2, and Supplementary Tables 2–7). Regionally, High-income Asia Pacific had the highest age-standardized prevalence rate of MC [45.6 (95% UI 41.1–50.1) per 100,000 people], while the highest age-standardized prevalence rates of AC and OC were observed in Eastern Europe [115.6 (95% UI 97.1–135.8) per 100,000 people] and Southern Sub-Saharan Africa [150.3 (95% UI 120.5–184.0) per 100,000 people], respectively. Overall, national age-standardized prevalence rates of MC varied by 10.4 times across all countries, ranging from 10.2 (95% UI 9.0–11.4) per 100,000 people in Chile to 105.6 (95% UI 90.8–120.8) per 100,000 people in Albania (Figure 2 and Supplementary Table 5). National age-standardized prevalence rates of AC varied by as much as 252.6 times, ranging from 1.4 (95% UI 1.2–1.7) per 100,000 people in Tajikistan to 356.1 (95% UI 300.9–419.6) per 100,000 people in Montenegro (Figure 2 and Supplementary Table 6). Additionally, national age-standardized prevalence rates of OC varied by 38.1 times, ranging from 7.4 (95% UI 6.2–8.7) per 100,000 people in Kyrgyzstan to 280.3 (95% UI 236.2–330.6) per 100,000 people in Slovenia (Supplementary Figure 2 and Supplementary Table 7).

### Deaths, YLDs, and YLLs in 2017

In 2017, the global numbers of deaths attributable to MC, AC, and OC were 46,486 (95% UI 39,709–51,824), 88,890 (95% UI 80,935–96,290) and 233,159 (95% UI 213,677–248,289), respectively. The age-standardized death rates of MC, AC, and OC per 100,000 people in 2017 were 0.6 (95% UI 0.5–0.7), 1.1 (95% UI 1.0–1.2), and 3.1 (95% UI 2.8–3.3), respectively (Table 1). By sex, the age-standardized death rate of MC was similar between males and females, whereas the age-standardized death rates of AC and OC were higher in males than females (Table 1). By age group, the numbers of deaths peaked at the ages of 85–89 years for MC and 55–59 years for AC in both sexes, while the numbers of deaths attributable to OC peaked at the ages of 80–84 years in males and 85–89 years in females (Figure 1 and Supplementary Figure 1). Notably, the numbers of deaths attributable to MC and OC were much higher in children aged <5 years than other young people.

**Figure 3:**
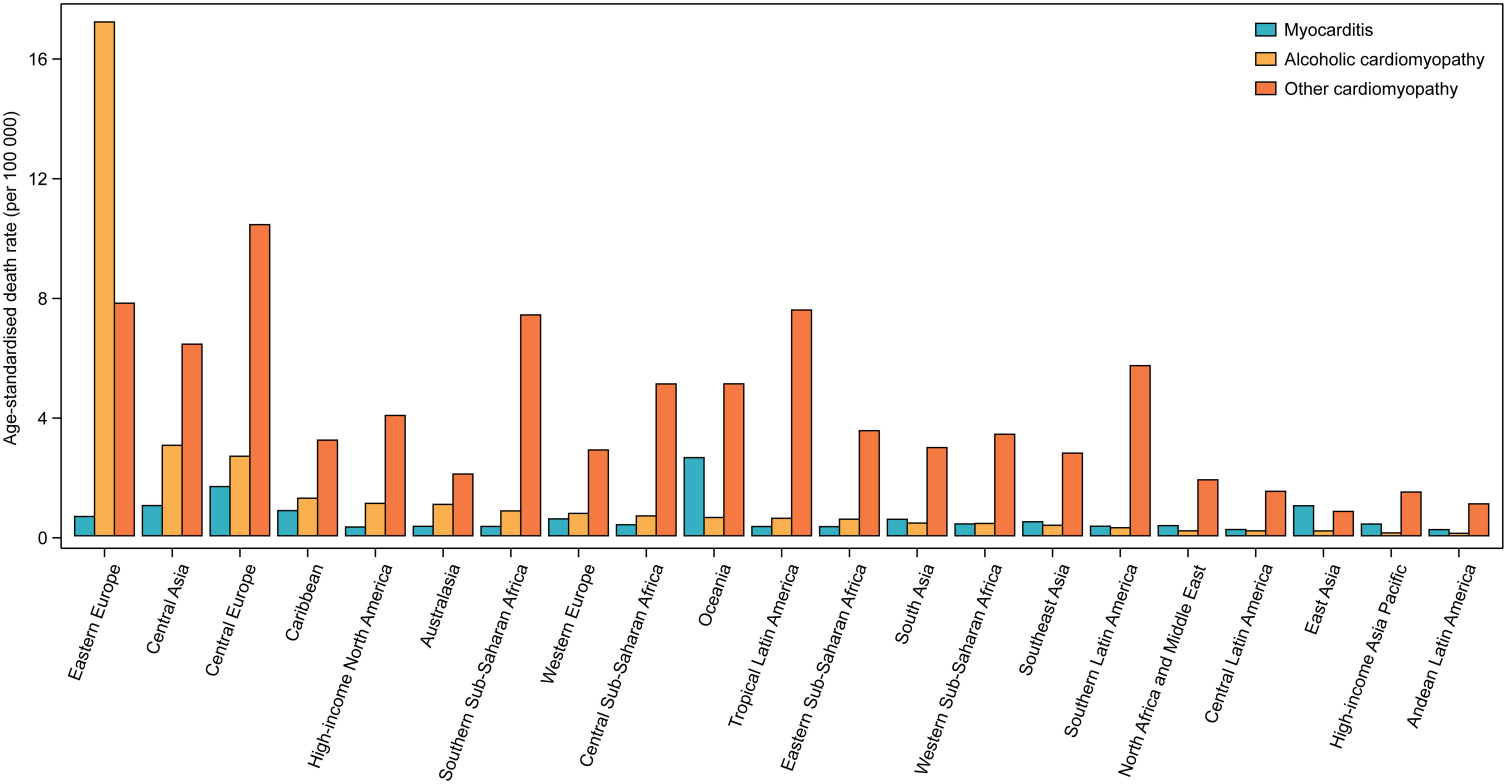
Age-standardized death rates of myocarditis, alcoholic cardiomyopathy, and other cardiomyopathy for 21 world regions, both sexes, 2017.

Across 21 world regions, Oceania had the highest age-standardized death rates of MC [2.6 (95% UI 2.0–3.4) per 100,000 people], whereas the highest age-standardized death rates of AC and OC were seen in Eastern Europe [17.2 (95% UI 16.2–19.1) per 100,000 people] and Central Europe [10.4 (95% UI 9.6–11.0) per 100,000 people], respectively (Figure 3). At the national level, the age-standardized death rates of MC varied by 43.9 times across all countries, ranging from 0.10 (95% UI 0.08–0.12) per 100,000 people in Bahrain to 4.3 (95% UI 3.4–5.4) per 100,000 people in Albania (Supplementary Figure 3 and Supplementary Table 5). National age-standardized death rates of AC varied by 531.0 times, ranging from 0.04 (95% UI 0.03–0.04) per 100,000 people in Turkey to 18.7 (95% UI 17.3–21.5) per 100,000 people in Russian Federation (Supplementary Figure 4 and Supplementary Table 6). National age-standardized death rates of OC varied by 43.3 times, ranging from 0.41 (95% UI 0.33–0.47) per 100,000 people in Kyrgyzstan to 17.6 (95% UI 15.5–19.3) per 100,000 people in Dominica (Supplementary Figure 5 and Supplementary Table 7).

Moreover, globally, there were 131,376 (95% UI 90,113–183,001) YLDs and 1.26 million (95% UI 1.10–1.42) YLLs attributable to MC, 139,087 (95% UI 95,134–196,130) YLDs and 2.84 million (95% UI 2.60–3.07) YLLs attributable to AC, and 353,325 (95% UI 237,907–493,908) YLDs and 5.51 million (95% UI 4.95–5.99) YLLs attributable to OC in 2017. As shown in Table 1, the age-standardized YLD and YLL rates per 100,000 people in 2017 were 1.7 (95% UI 1.2–2.4) and 16.6 (95% UI 14.5–18.5), respectively for MC; 1.7 (95% UI 1.2–2.4) and 34.7 (95% UI 31.7–37.5), respectively for AC; and 4.5 (95% UI 3.1–6.3) and 71.1 (95% UI 64.0–77.0), respectively for OC. Regionally, the highest age-standardized YLD rates of MC, AC and OC were observed in High-income Asia Pacific [3.6 (95% UI 2.5–5.0) per 100,000 people], Eastern Europe [9.8 (95% UI 6.7–13.8) per 100,000 people] and Southern Sub-Saharan Africa [12.3 (95% UI 8.0–17.3) per 100,000 people], respectively; while the highest age-standardized YLL rates of MC, AC, and OC were observed in Oceania [77.0 (95% UI 58.0–104.5) per 100,000 people], Eastern Europe [643.7 (95% UI 606.6–718.5) per 100,000 people], and Eastern Europe [223.0 (95% UI 135.2–256.6) per 100,000 people], respectively (Supplementary Tables 5–7). At the national level, the age-standardized YLD rates varied by 12.4 times for MC, 223.7 times for AC, and 34.1 times for OC; and the age-standardized YLL rates varied by 38.4 times for MC, 684.8 times for AC, and 36.2 times for OC (Supplementary Tables 5–7). More details about the age-standardized YLD and YLL rates of these diseases by sex, SDI quintile, and geographic location can be seen in Table 1 and Supplementary Tables 5–7.

### Temporal Trends of Burden From 1990 to 2017

Between 1990 and 2017, the age-standardized prevalence rates of MC, AC and OC decreased by −6.9% (95% UI −7.8 to −5.9), −26.2% (95% UI −28.2 to −24.0), and −15.5% (95% UI −18.4 to −12.7), respectively, with greater decreases in females than males for all the diseases (Table 1). Despite the decreases, the global numbers of MC, AC, and OC have increased by 49.8% (95% UI 45.7–54.0), 39.0% (95% UI 35.3–43.1), and 65.3% (95% UI 60.2–70.8), respectively. By SDI quintile, the greatest decreases in age-standardized prevalence rates were found in countries in the high SDI quintile for both MC and OC, but in countries in the high-middle SDI quintile for AC (Table 1).

**Figure 4:**
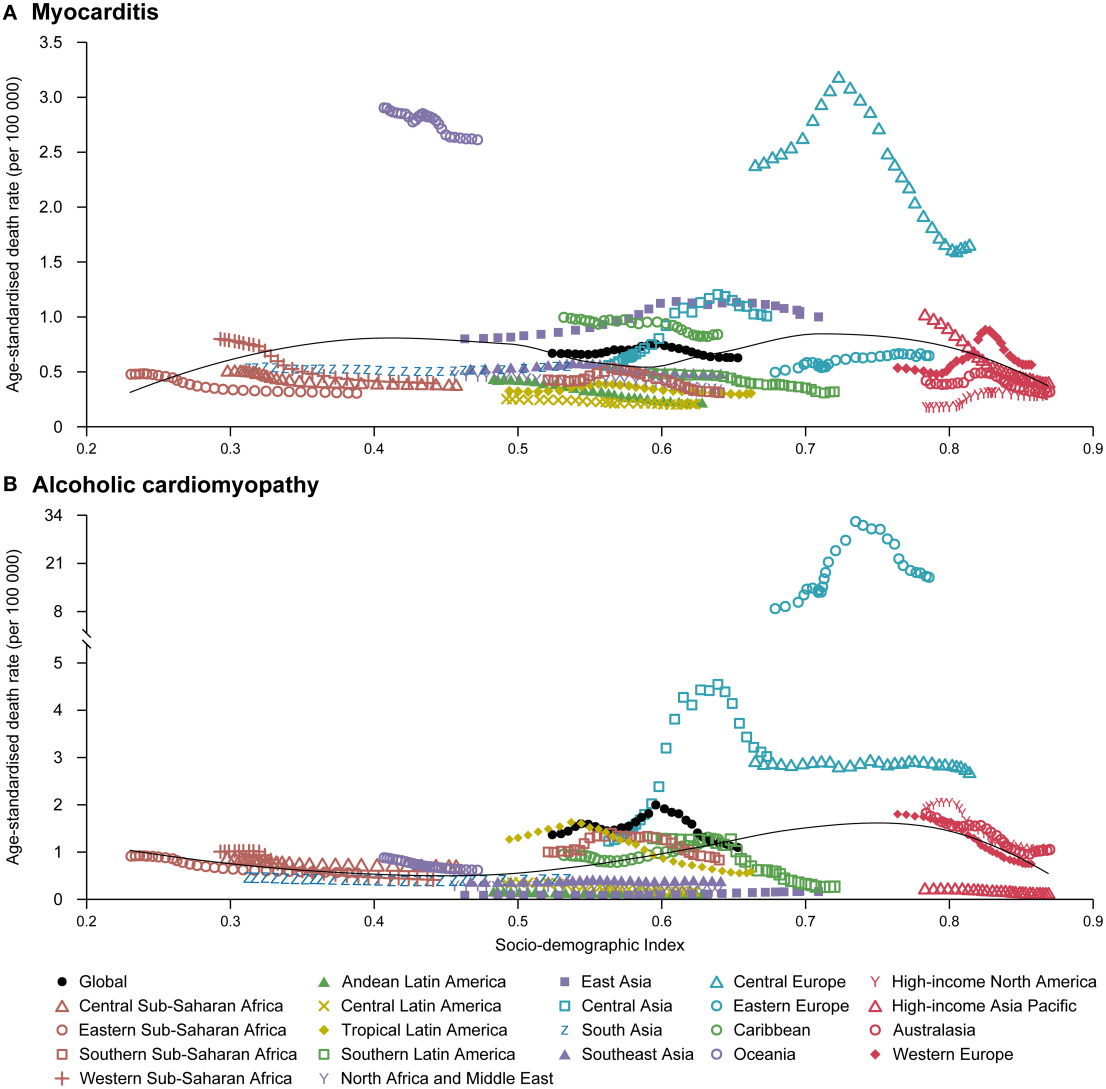
Temporal trends in age-standardized death rates of myocarditis **(A)** and alcoholic cardiomyopathy **(B)** for 21 world regions by SDI, both sexes, 1990–2017. For each region, points from left to right depict estimates from each year from 1990 to 2017. SDI, Socio-demographic Index.

Similarly, the global numbers of deaths attributable to MC, AC and OC have increased by 71.4% (95% UI 54.9–90.5), 59.6% (95% UI 33.9–76.3), and 49.7% (95% UI 39.5–73.4), respectively from 1990 to 2017, despite the decreases in age-standardized death rates (Table 1). The decreases in age-standardized death rates were also greater in females than males for MC, AC, and OC (Table 1). However, the change patterns in age-standardized death rates across SDI quintiles were not similar to age-standardized prevalence rates for all the diseases (Table 1). Furthermore, there were no clear patterns between age-standardized death rates for the 21 world regions and SDI over the 1990–2017 period for all the diseases (Figure 4 and Supplementary Figure 6). These results indicated that the SDI level of the country is not a major determinant of size of deaths for these diseases. More information about the temporal changes in the burden of MC, AC, and OC from 1990 to 2017 was available in Table 1 and Supplementary Tables 5–7.

## Discussion

In this study, we utilized the GBD 2017 modeling framework to estimate the global burden generated by MC, AC, and OC, stratifying findings by age, sex, year (1990–2017), SDI, and geographic location. Most recently, a modeling study has estimated the death rate of AC in 2015 at the global, regional, and national levels, but without examining the prevalence, YLDs, and YLLs or exploring temporal trends of the burden over time (15). Additionally, to the best of our knowledge, no prior study has systematically reported the burden of MC and OC at the global, regional, and national levels. In the present study, we found that there were an estimated 1.80 million cases of MC, 1.62 million cases of AC, and 4.21 million cases of OC globally in 2017, contributing to 46,486, 88,890, and 233,159 deaths, respectively. Furthermore, globally, there were 131,376 YLDs and 1.26 million YLLs attributable to MC, 139,087 YLDs and 2.84 million YLLs attributable to AC, and 353,325 YLDs and 5.51 million YLLs attributable to OC in 2017. This study provides, indeed, health decision- and policy-makers with an updated synthesis of the epidemiology of MC, AC, and OC to inform the strategic development, planning of prevention and treatment programs and optimization of health system resource allocation.

As expected, the age-standardized prevalence and death rates of AC were found to be significantly higher in males than females, and the burden of AC mainly concentered in adults aged 40–70 years (16). Alcohol consumption represents a major risk factor for global disease burden and generates substantial health losses (17). The higher frequency of alcohol use, as the risk factor for AC, in males compared with females can explain the higher prevalence rate of AC in males (18). Considering the significant impact of alcohol intake as etiopathogenetic driver of AC, the analysis of national trends and projections of alcohol consumption remain essential for AC surveillance and the development of targeted prevention strategies. In addition, it is noteworthy that although the age-standardized prevalence rates of MC and OC were lowest in children aged <5 years, their corresponding numbers of deaths were much higher than in other young people. Actually, pediatric cardiomyopathies have a poor prognosis despite optimal medical management, with 40% of children with symptomatic cardiomyopathy dying or undergoing cardiac transplantation within 2 years (19). Further studies are warranted in order to fully understand the risk factors of pediatric cardiomyopathies to improve prognosis.

The burden of MC in 2017 varied significantly across geographic locations. Regionally, High-income Asia Pacific had the highest age-standardized prevalence rate of MC, which is probably because of the infection caused by hepatitis C virus (HCV) (5). According to a study by Matsumori et al. (20), anti-HCV antibodies are frequently detected in MC patients with a higher rate than in the general population. While among all the industrialized countries, Japan has the highest prevalence rate of HCV, with more than 1 million cases of infections (21). However, despite High-income Asia Pacific reported the highest age-standardized prevalence rate of MC, the highest age-standardized death rate due to MC was seen in Oceania, which is probably due to the lack of adequate healthcare resources, especially in remote underserved areas.

In addition, large geographic differences in the burden of AC and OC were also observed in the present study. The highest age-standardized prevalence rates of AC in Eastern Europe may be explained taking into account the high level of alcohol consumption, in terms of drinking pattern, frequency, quality and type of alcoholic beverage (22). It has been reported that the detrimental effects of drinking patterns, such as alcohol binge drinking are a major public health issue, in Europe and especially in Eastern Europe (23). Even though these countries have enacted restrictive policies on alcohol consumption which have contributed to a decline in alcohol-related mortality rate, the burden remains dramatically high (24). The highest age-standardized prevalence rate of OC in Southern Sub-Saharan Africa can be partly explained by the high prevalence of communicable disease, in particular infectious agents like *Toxoplasma gondii* and *Schistosoma* (25). Also, peripartum cardiomyopathy is quite widespread in the African continent (25, 26). On the other hand, in resource-limited regions like Sub-Saharan Africa, the true prevalence of OC could even be under-estimated, due to the lack of detection systems (27, 28).

Concerning the temporal trends of burden from 1990 to 2017, the global numbers of prevalent cases and deaths for MC, AC, and OC have significant increased, contrasting with the decreases in age-standardized prevalence and death rates. The exact causes for the trends are unknown but may be in part due to population growth and aging (29). In terms of sex, females had greater decreases in age-standardized prevalence and death rates than males for all the diseases during 1990–2017, suggesting that preventive policies and intervention treatments may be more effective in females in the same timeframe. To our best knowledge, the associations of the burden of MC, AC, and OC with developing status of regions and countries haven't been explored in previous studies. In the present study, we found that there were no clear patterns between age-standardized death rates for the 21 world regions and SDI over the 1990–2017 period for all the diseases, suggesting that such associations are complex and non-linear. Indeed, the burden of MC, AC, and OC is not limited to developed or less developed countries and a high burden of these diseases was seen in countries with various SDI.

As YLDs were calculated on the basis of prevalence (in number of cases), the patterns of YLDs by SDI and geographic location for MC, AC, and OC were similar to prevalence. The same relationship existed between YLLs and deaths. Globally, although the absolute numbers of YLDs were lower than YLLs for MC, the reductions in age-standardized YLD rates (−7.2%) during 1990–2017 have been much smaller than age-standardized YLL rates (−23.5%), suggesting that future MC treatment strategies should pay more attention to improve the quality of life of MC patients. Conversely, for males, the age-standardized YLD rates of AC have decreased by −25.7% during 1990–2017, while there was no significant change in age-standardized YLL rates of AC in the same timeframe, suggesting that future AC treatment strategies should still pay more attention to reduce the mortality of male patients with AC. However, for females, there was no difference between age-standardized YLD and YLL rates of AC.

### Limitations

Even though the GBD estimations fill a gap where data on the burden imposed by MC, OC, and AC are scarce or inaccessible, several limitations still exist and should be properly acknowledged. The first limitation is the low availability of data in some countries, although statistically robust approaches have been applied in order to overcome data scarcity and deal with uncertainty. In those countries for which data was missing or was deemed inaccurate and of low quality, results mainly relied on covariates known to be associated with these diseases, trends in neighboring countries, or a combination of both methods. Besides, differences in data collection and data sources quality across countries, delay and inaccuracy in reporting, misclassification and bias in coding, are unavoidable in this study, even though the GBD has attempted to enhance the reliability and comparability of related data. Moreover, diagnostic criteria may have changed over time and may reflect different regional use of coding. Various nomenclatures, classification schemes, and nosological taxonomies, indeed, exist, some of them being even confusing and contrasting, and have been differently employed in the literature (30).

## Conclusions

In summary, MC, AC and OC remain important global public health problems; however, large national and regional variations in the burden were seen for all these diseases. Public health policy- and decision-makers should devise and implement more effective and geo-specific strategies aimed at counteracting and mitigating the future burden of these diseases. Further research is also warranted to expand our knowledge of potential risk factors associated with MC, AC, and OC and to improve the prevention, early detection and treatment of these diseases.

## Data Availability Statement

The datasets presented in this study can be found in online repositories. The names of the repository/repositories and accession number(s) can be found in the article/Supplementary Material.

## Ethics Statement

The studies involving human participants were reviewed and approved by University of Washington Institutional Review Board. Written informed consent from the participants' legal guardian/next of kin was not required to participate in this study in accordance with the national legislation and the institutional requirements.

## Author Contributions

HD involved in data collection, study design, statistical analysis, manuscript preparation, and supervision. DL and AM involved in study design, data interpretation, and manuscript review. AY involved in data interpretation and manuscript review. YL and NB involved in study design, statistical analysis, manuscript preparation, manuscript review, and supervision. JW involved in manuscript review and supervision. All authors have read and approved the final manuscript.

## Conflict of Interest

The authors declare that the research was conducted in the absence of any commercial or financial relationships that could be construed as a potential conflict of interest.
