# Supplementary material for: Global, Regional, and National Burden of Myocarditis and Cardiomyopathy, 1990–2017
Source: Front Cardiovasc Med. 2021 Feb 11;8:610989. doi: 10.3389/fcvm.2021.610989 (PMC7904878; doi:10.3389/fcvm.2021.610989)
Supplement: Supplementary file 1 [file Data_Sheet_1.PDF]

Supplementary Figures and Tables:

Supplementary Figure 1. Age-specific numbers and rates of prevalent cases (A) and deaths (B) for other cardiomyopathy by sex, 2017... **2**

Supplementary Figure 2. Age-standardised prevalence rates of other cardiomyopathy for 195 countries and territories, both sexes, 2017.......................... **3**

Supplementary Figure 3. Age-standardised death rates of myocarditis for 195 countries and territories, both sexes, 2017... **4**

Supplementary Figure 4. Age-standardised death rates of alcoholic cardiomyopathy for 195 countries and territories, both sexes, 2017.. **5**

Supplementary Figure 5. Age-standardised death rates of other cardiomyopathy for 195 countries and territories, both sexes, 2017.. **6**

Supplementary Figure 6. Temporal trends in age-standardised death rates of other cardiomyopathy for 21 world regions by SDI, both sexes, 1990-2017....................... **7**

Supplementary Table 1. Selected covariates for CODEm models for myocarditis, alcoholic cardiomyopathy, and other cardiomyopathy...**8**

Supplementary Table 2. Numbers of myocarditis prevalent cases, deaths, YLDs, and YLLs in 2017, by sex, SDI quintile, and location..**9**

Supplementary Table 3. Numbers of alcoholic cardiomyopathy prevalent cases, deaths, YLDs, and YLLs in 2017, by sex, SDI quintile, and location.........................**14**

Supplementary Table 4. Numbers of other cardiomyopathy prevalent cases, deaths, YLDs, and YLLs in 2017, by sex, SDI quintile, and location.....................................**19**

Supplementary Table 5. Age-standardised prevalence, death, YLD, and YLL rates of myocarditis for both sexes in 2017, and their percentage changes from 1990 to 2017, by location...**24**

Supplementary Table 6. Age-standardised prevalence, death, YLD, and YLL rates of alcoholic cardiomyopathy for both sexes in 2017, and their percentage changes from 1990 to 2017, by location..**29**

Supplementary Table 7. Age-standardised prevalence, death, YLD, and YLL rates of other cardiomyopathy for both sexes in 2017, and their percentage changes from 1990 to 2017, by location..**34**

Supplementary Figure 1. Age-specific numbers and rates of prevalent cases (A) and deaths (B) for other cardiomyopathy by sex, 2017.

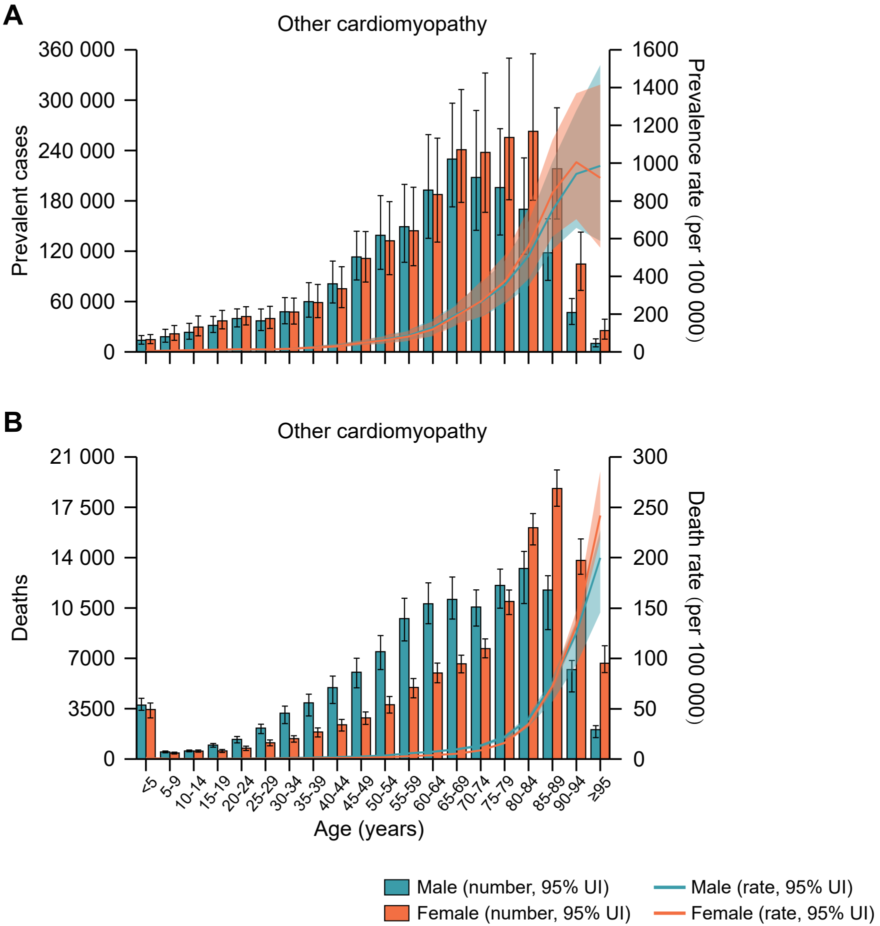

Supplementary Figure 2. Age-standardised prevalence rates of other cardiomyopathy for 195 countries and territories, both sexes, 2017.

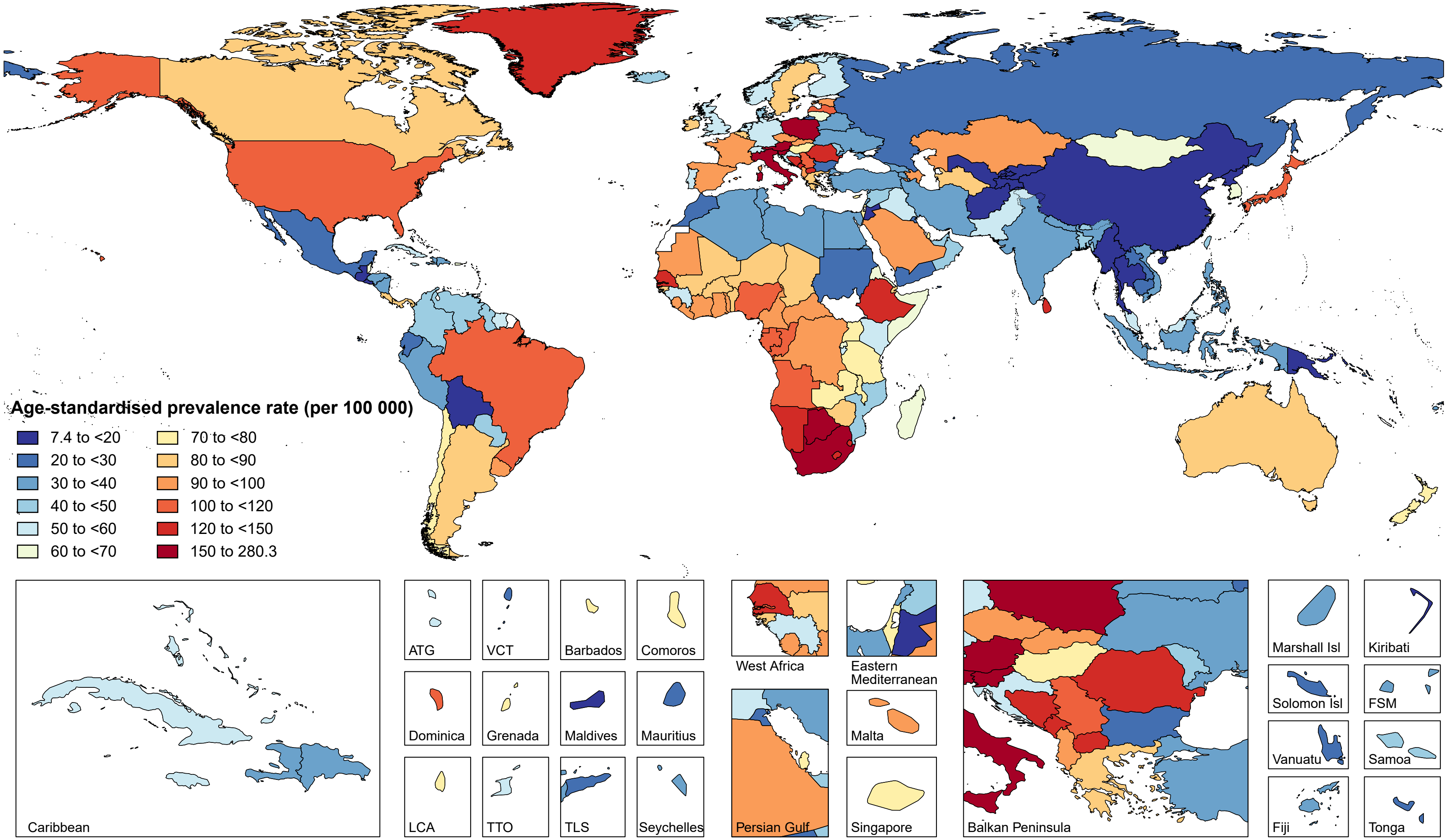

ATG, Antigua and Barbuda; Isl, Islands; FSM, Federated States of Micronesia; LCA, Saint Lucia; TLS, Timor-Leste; TTO, Trinidad and Tobago; VCT, Saint Vincent and the Grenadines.

Supplementary Figure 3. Age-standardised death rates of myocarditis for 195 countries and territories, both sexes, 2017.

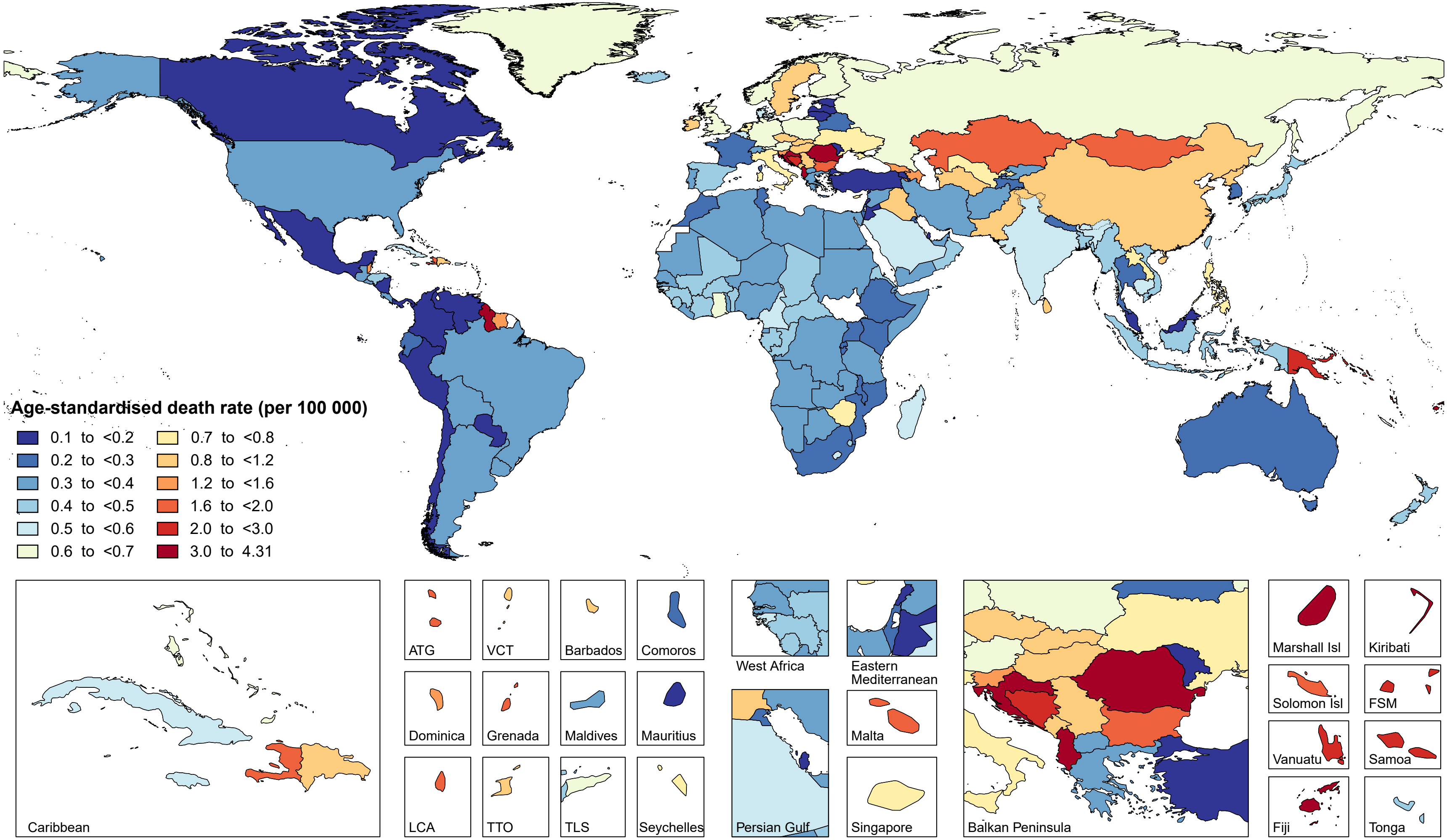

ATG, Antigua and Barbuda; Isl, Islands; FSM, Federated States of Micronesia; LCA, Saint Lucia; TLS, Timor-Leste; TTO, Trinidad and Tobago; VCT, Saint Vincent and the Grenadines.

Supplementary Figure 4. Age-standardised death rates of alcoholic cardiomyopathy for 195 countries and territories, both sexes, 2017

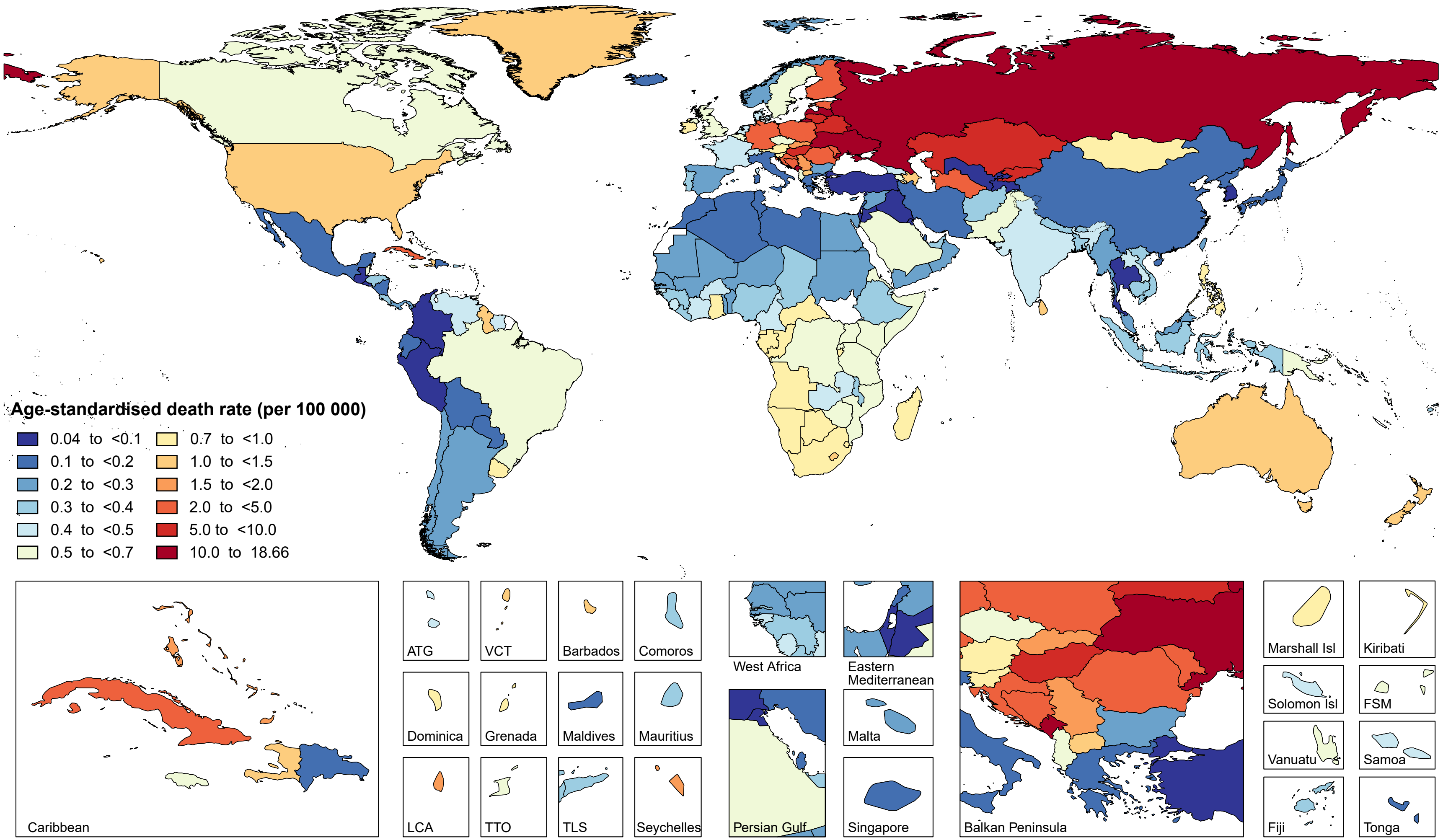

ATG, Antigua and Barbuda; Isl, Islands; FSM, Federated States of Micronesia; LCA, Saint Lucia; TLS, Timor-Leste; TTO, Trinidad and Tobago; VCT, Saint Vincent and the Grenadines.

Supplementary Figure 5. Age-standardised death rates of other cardiomyopathy for 195 countries and territories, both sexes, 2017.

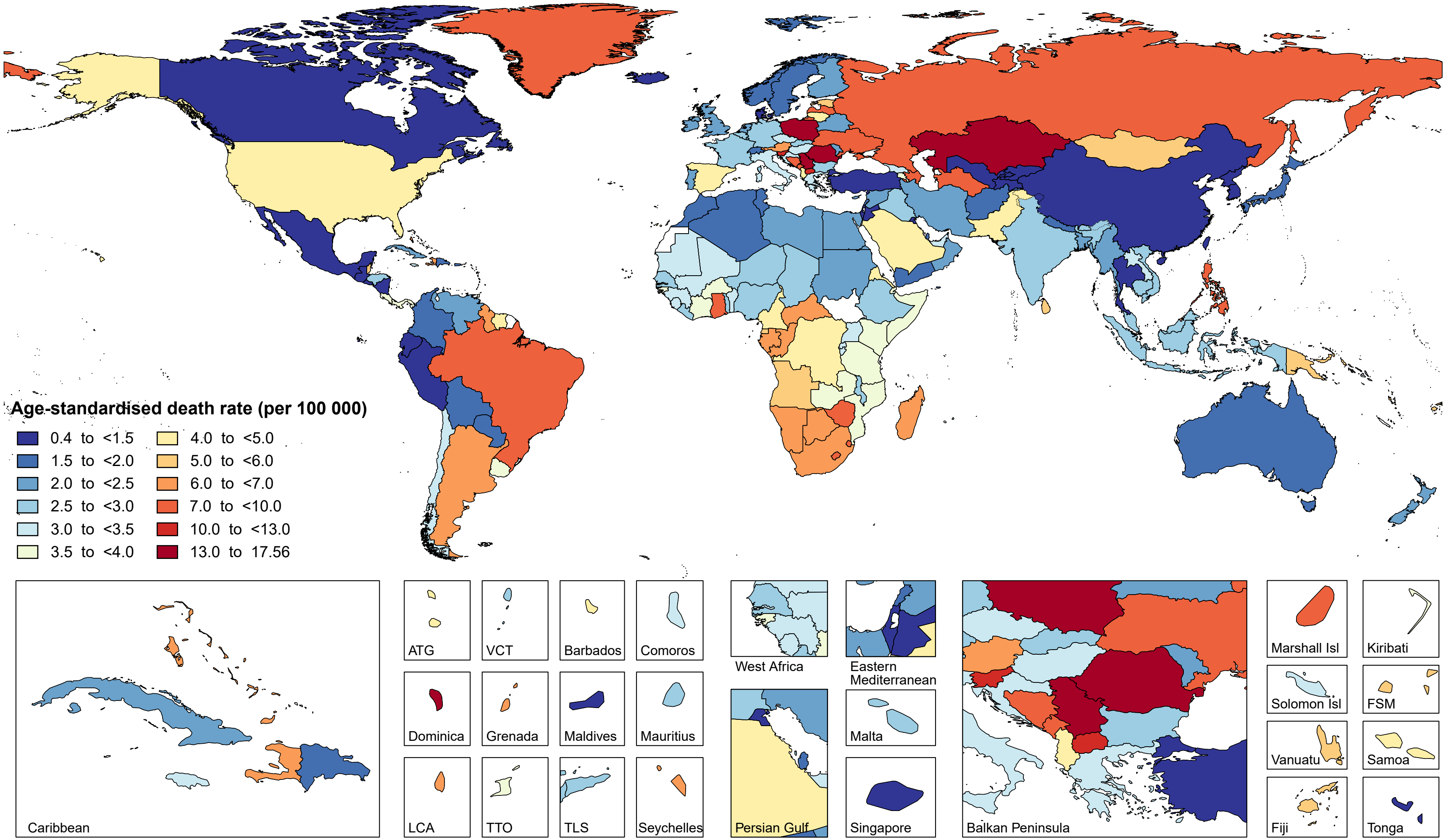

ATG, Antigua and Barbuda; Isl, Islands; FSM, Federated States of Micronesia; LCA, Saint Lucia; TLS, Timor-Leste; TTO, Trinidad and Tobago; VCT, Saint Vincent and the Grenadines.

Supplementary Figure 6. Temporal trends in age-standardised death rates of other cardiomyopathy for 21 world regions by SDI, both sexes, 1990-2017.

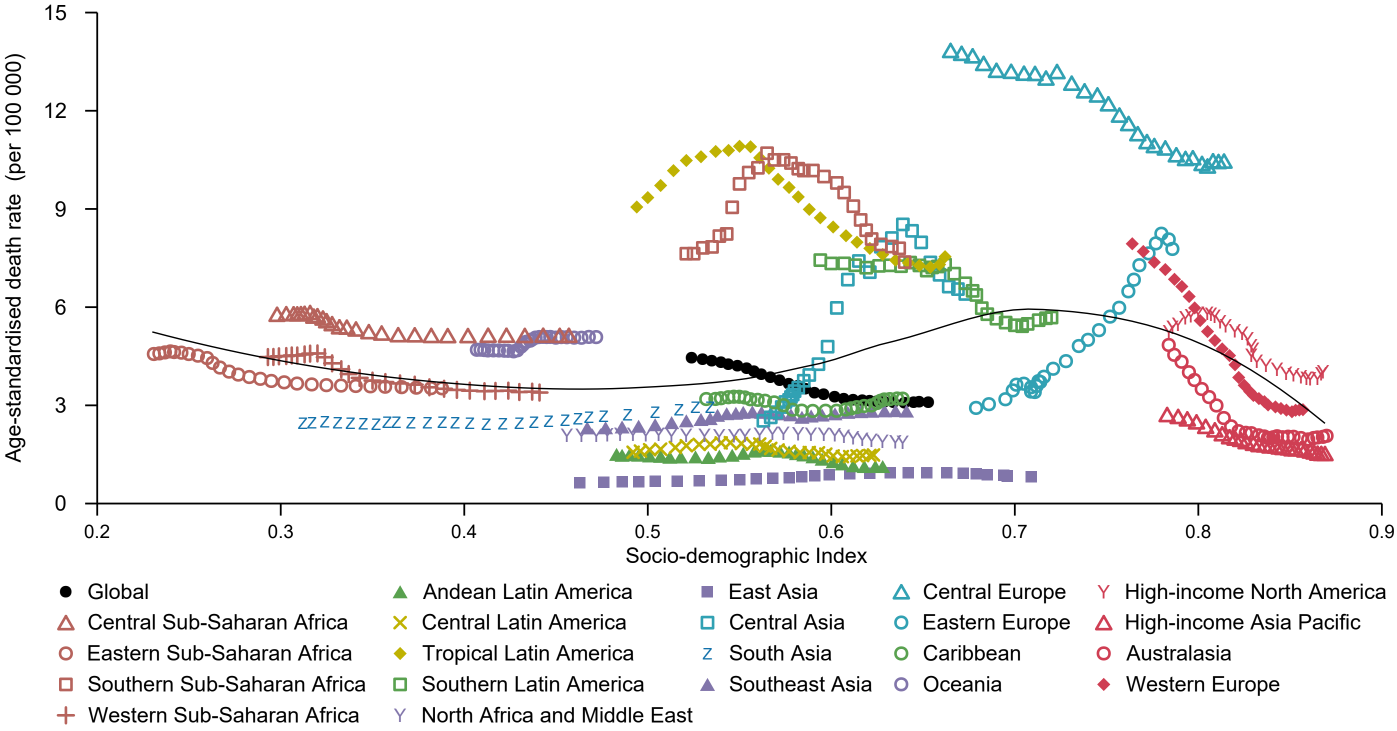

For each region, points from left to right depict estimates from each year from 1990 to 2017. SDI= Socio-demographic Index.

Supplementary Table 1. Selected covariates for CODEm models for myocarditis, alcoholic cardiomyopathy, and other cardiomyopathy.

	Covariate	Transformation	Level	Direction
Myocarditis	Summary exposure variable, CMP	None	1	1
	Systolic blood pressure (mm Hg)	None	1	1
	Healthcare access and quality index	None	2	-1
	Lag distributed income per capita (I$)	Log	3	0
	Socio-demographic Index	None	3	0
Alcoholic Cardiomyopathy	Summary exposure variable, CMP	None	1	1
	Smoking prevalence	None	1	1
	Alcohol (litres per capita)	None	1	1
	Healthcare access and quality index	None	2	-1
	Lag distributed income per capita (I$)	Log	3	0
	Socio-demographic Index	None	3	0
Other Cardiomyopathy	Summary exposure variable, CMP	None	1	1
	Systolic blood pressure (mmHg)	None	1	1
	Smoking prevalence	None	1	1
	Body mass index (kg/m^2)	None	2	1
	Healthcare access and quality index	None	2	-1
	Lag distributed income per capita (I$)	Log	3	0
	Socio-demographic Index	None	3	0

Supplementary Table 2. Numbers of myocarditis prevalent cases, deaths, YLDs, and YLLs in 2017, by sex, SDI quintile, and location.

	Prevalence		Deaths		YLDs		YLLs	
	Males	Females	Males	Females	Males	Females	Males	Females
Global	810167 (736794 to 890507)	994402 (897476 to 1092773)	21931 (17832 to 26594)	24555 (20123 to 27094)	57641 (39587 to 80137)	73735 (50776 to 102339)	686922 (565331 to 836085)	572369 (488933 to 643422)
SDI Quintile								
Low SDI	84717 (76064 to 94268)	91438 (81892 to 101324)	1822 (1090 to 2448)	1859 (1247 to 2599)	5899 (3978 to 8383)	6604 (4463 to 9276)	78727 (51254 to 102905)	83667 (55275 to 115615)
Low-middle SDI	132449 (119244 to 146444)	157888 (141444 to 174939)	3711 (2740 to 5295)	3621 (2691 to 4255)	9248 (6297 to 13023)	11591 (7883 to 16401)	145410 (111204 to 198192)	136630 (105058 to 162908)
Middle SDI	249038 (224194 to 274919)	294006 (262494 to 326335)	7735 (5641 to 10073)	6858 (5735 to 7633)	17743 (12147 to 24763)	21895 (14842 to 30653)	234949 (180480 to 311041)	167125 (144882 to 186630)
High-middle SDI	173901 (157316 to 191250)	209520 (188616 to 230333)	4848 (4163 to 5867)	5050 (4327 to 6352)	12295 (8373 to 17173)	15316 (10503 to 21415)	141320 (123417 to 165785)	97633 (88326 to 117041)
High SDI	165663 (151926 to 180106)	236303 (214622 to 258256)	3676 (2759 to 4503)	7021 (4873 to 8023)	12136 (8419 to 16742)	17926 (12459 to 24615)	82566 (64892 to 95880)	84090 (63295 to 93060)
Central Sub-Saharan Africa	7124 (6327 to 8022)	6667 (5936 to 7402)	135 (72 to 213)	120 (65 to 236)	488 (330 to 703)	455 (304 to 636)	6818 (3638 to 11802)	6552 (3286 to 13094)
Angola	1597 (1414 to 1800)	1580 (1404 to 1773)	31 (18 to 51)	26 (14 to 56)	110 (74 to 158)	109 (73 to 156)	1578 (825 to 2710)	1396 (725 to 3022)
Central African Republic	277 (245 to 310)	255 (226 to 285)	7 (3 to 13)	7 (4 to 14)	19 (12 to 27)	17 (12 to 25)	337 (120 to 755)	429 (194 to 824)
Congo	332 (296 to 371)	299 (269 to 333)	7 (3 to 14)	6 (3 to 14)	23 (15 to 32)	20 (14 to 29)	325 (163 to 640)	284 (140 to 630)
Democratic Republic of the Congo	4710 (4185 to 5335)	4334 (3853 to 4851)	86 (39 to 139)	78 (39 to 154)	323 (217 to 465)	295 (197 to 415)	4391 (2037 to 7663)	4341 (1939 to 8572)
Equatorial Guinea	83 (72 to 94)	72 (64 to 80)	1 (1 to 2)	1 (0 to 2)	6 (4 to 8)	5 (3 to 7)	61 (25 to 131)	42 (17 to 105)
Gabon	125 (111 to 140)	128 (115 to 141)	3 (2 to 6)	2 (1 to 4)	9 (6 to 12)	9 (6 to 12)	126 (72 to 254)	60 (31 to 145)
Eastern Sub-Saharan Africa	24101 (21534 to 26943)	21443 (19183 to 23783)	482 (196 to 867)	221 (121 to 499)	1669 (1127 to 2347)	1458 (989 to 2080)	24793 (11583 to 42626)	12245 (7022 to 27251)
Burundi	581 (515 to 650)	495 (440 to 555)	12 (5 to 26)	6 (3 to 13)	39 (26 to 55)	32 (21 to 46)	670 (243 to 1338)	398 (186 to 871)
Comoros	49 (44 to 55)	46 (41 to 51)	1 (0 to 2)	0 (0 to 1)	3 (2 to 5)	3 (2 to 4)	41 (14 to 80)	19 (10 to 43)
Djibouti	79 (70 to 88)	60 (53 to 67)	2 (1 to 4)	1 (0 to 1)	5 (4 to 8)	4 (3 to 6)	82 (28 to 175)	26 (11 to 65)
Eritrea	320 (281 to 361)	313 (278 to 350)	12 (3 to 25)	5 (2 to 12)	22 (15 to 31)	21 (14 to 30)	641 (160 to 1226)	263 (109 to 588)
Ethiopia	8338 (7371 to 9355)	6919 (6167 to 7650)	101 (37 to 182)	48 (26 to 110)	615 (413 to 857)	503 (339 to 715)	5137 (2209 to 8413)	2785 (1489 to 6229)
Kenya	2775 (2461 to 3123)	2474 (2215 to 2771)	59 (19 to 122)	20 (10 to 48)	184 (126 to 262)	160 (108 to 228)	2712 (972 to 5238)	929 (468 to 2081)
Madagascar	1460 (1296 to 1653)	1308 (1159 to 1468)	62 (24 to 117)	29 (13 to 67)	99 (66 to 143)	88 (59 to 126)	3235 (1371 to 5869)	1526 (675 to 3633)
Malawi	894 (790 to 1001)	912 (813 to 1017)	16 (8 to 31)	7 (3 to 17)	58 (39 to 83)	59 (40 to 84)	839 (424 to 1617)	383 (175 to 881)
Mozambique	1269 (1123 to 1431)	1301 (1151 to 1463)	34 (15 to 74)	14 (6 to 32)	78 (52 to 113)	80 (52 to 116)	1733 (740 to 3645)	733 (320 to 1754)
Rwanda	682 (602 to 768)	676 (601 to 755)	14 (4 to 28)	7 (3 to 17)	46 (30 to 66)	45 (30 to 63)	666 (232 to 1198)	342 (160 to 807)
Somalia	927 (817 to 1041)	791 (703 to 892)	22 (10 to 40)	12 (6 to 27)	63 (42 to 90)	53 (36 to 77)	1229 (511 to 2278)	729 (330 to 1598)
South Sudan	554 (489 to 628)	462 (411 to 521)	12 (5 to 23)	9 (4 to 21)	38 (25 to 54)	31 (21 to 45)	722 (208 to 1345)	583 (251 to 1361)
Tanzania	3151 (2783 to 3543)	2956 (2625 to 3310)	69 (25 to 130)	37 (18 to 86)	213 (144 to 304)	198 (133 to 284)	3632 (1558 to 6718)	2112 (972 to 4773)
Uganda	2039 (1793 to 2311)	1855 (1657 to 2066)	44 (15 to 84)	16 (7 to 39)	138 (91 to 199)	121 (82 to 173)	2324 (875 to 4240)	937 (417 to 2190)
Zambia	968 (854 to 1096)	860 (761 to 969)	22 (7 to 44)	8 (4 to 20)	66 (45 to 94)	58 (39 to 82)	1115 (408 to 2273)	471 (232 to 1094)
Southern Sub-Saharan Africa	5980 (5371 to 6657)	5748 (5194 to 6320)	124 (92 to 203)	76 (60 to 116)	416 (281 to 586)	382 (258 to 546)	6187 (4451 to 10081)	3216 (2454 to 5087)
Botswana	211 (186 to 236)	182 (163 to 202)	3 (2 to 5)	2 (1 to 3)	15 (10 to 22)	13 (9 to 18)	149 (73 to 246)	65 (46 to 103)
Lesotho	145 (129 to 164)	147 (131 to 163)	4 (2 to 7)	3 (2 to 5)	10 (7 to 15)	10 (7 to 14)	209 (105 to 355)	118 (75 to 174)
Namibia	201 (178 to 226)	189 (168 to 209)	4 (2 to 8)	2 (1 to 3)	15 (10 to 21)	13 (9 to 19)	212 (90 to 364)	72 (46 to 117)
South Africa	4279 (3844 to 4737)	4213 (3806 to 4652)	65 (52 to 95)	39 (31 to 59)	291 (196 to 410)	274 (186 to 391)	2912 (2234 to 4495)	1500 (1165 to 2361)
Swaziland	78 (70 to 88)	78 (70 to 87)	2 (1 to 4)	1 (1 to 2)	6 (4 to 8)	5 (4 to 8)	120 (67 to 187)	51 (35 to 74)
Zimbabwe	1066 (921 to 1223)	940 (832 to 1054)	45 (22 to 99)	29 (18 to 49)	79 (53 to 113)	66 (44 to 95)	2585 (1359 to 5413)	1410 (875 to 2436)
Western Sub-Saharan Africa	29767 (26294 to 33656)	30119 (26824 to 33710)	475 (344 to 609)	399 (291 to 499)	2154 (1443 to 3072)	2179 (1462 to 3078)	23428 (17669 to 29860)	19619 (13086 to 25497)
Benin	765 (665 to 869)	776 (685 to 870)	12 (6 to 21)	13 (8 to 18)	55 (37 to 80)	56 (37 to 80)	600 (313 to 1030)	608 (338 to 925)
Burkina Faso	1330 (1168 to 1517)	1373 (1224 to 1548)	22 (11 to 35)	26 (14 to 38)	95 (63 to 136)	98 (65 to 138)	1097 (492 to 1778)	1369 (635 to 2100)
Cameroon	1847 (1621 to 2096)	1847 (1627 to 2078)	39 (26 to 57)	30 (19 to 46)	132 (88 to 190)	133 (88 to 190)	2003 (1293 to 2976)	1481 (905 to 2311)
Cape Verde	63 (55 to 72)	85 (73 to 99)	1 (1 to 1)	1 (0 to 1)	5 (3 to 7)	6 (4 to 9)	32 (24 to 41)	12 (9 to 15)
Chad	962 (837 to 1099)	907 (798 to 1029)	14 (8 to 23)	19 (9 to 31)	69 (46 to 99)	66 (44 to 94)	751 (422 to 1179)	1121 (458 to 1913)

Cote d'Ivoire	1773 (1562 to 2032)	1581 (1400 to 1772)	39 (23 to 62)	24 (16 to 34)	127 (84 to 179)	113 (76 to 159)	1982 (1244 to 3117)	1309 (828 to 1881)
The Gambia	153 (133 to 175)	154 (136 to 173)	3 (2 to 5)	2 (1 to 2)	11 (7 to 16)	11 (7 to 16)	139 (76 to 238)	73 (49 to 100)
Ghana	1867 (1646 to 2101)	2018 (1800 to 2265)	41 (27 to 64)	49 (35 to 70)	128 (85 to 185)	139 (93 to 199)	2113 (1385 to 3152)	1840 (1268 to 2759)
Guinea	716 (634 to 810)	696 (613 to 782)	14 (8 to 23)	16 (9 to 25)	50 (33 to 71)	48 (32 to 68)	711 (412 to 1092)	778 (406 to 1202)
Guinea-Bissau	115 (101 to 132)	122 (108 to 137)	3 (1 to 4)	2 (1 to 3)	8 (6 to 12)	9 (6 to 12)	135 (70 to 217)	118 (73 to 172)
Liberia	328 (291 to 374)	300 (267 to 338)	5 (2 to 7)	4 (2 to 6)	23 (16 to 34)	21 (14 to 30)	231 (118 to 361)	188 (108 to 275)
Mali	1389 (1213 to 1582)	1187 (1046 to 1338)	23 (10 to 42)	27 (12 to 42)	102 (67 to 147)	85 (57 to 121)	1175 (507 to 2112)	1665 (643 to 2612)
Mauritania	306 (266 to 346)	286 (253 to 322)	5 (3 to 8)	3 (2 to 5)	22 (15 to 33)	21 (14 to 29)	209 (114 to 345)	123 (80 to 166)
Niger	1335 (1167 to 1532)	1236 (1096 to 1400)	15 (6 to 30)	20 (8 to 36)	98 (65 to 140)	89 (60 to 126)	799 (324 to 1570)	1248 (416 to 2186)
Nigeria	14553 (12745 to 16445)	15325 (13622 to 17202)	207 (114 to 338)	136 (88 to 196)	1064 (706 to 1537)	1125 (751 to 1585)	9831 (5812 to 15779)	6503 (4045 to 9884)
Sao Tome and Principe	15 (13 to 17)	15 (13 to 17)	0 (0 to 1)	0 (0 to 0)	1 (1 to 2)	1 (1 to 1)	18 (11 to 29)	7 (5 to 11)
Senegal	1205 (1056 to 1375)	1170 (1036 to 1314)	14 (7 to 23)	11 (8 to 15)	89 (59 to 125)	85 (57 to 122)	686 (368 to 1077)	444 (296 to 628)
Sierra Leone	524 (462 to 597)	504 (449 to 567)	10 (6 to 14)	8 (5 to 12)	37 (25 to 53)	35 (24 to 51)	497 (318 to 700)	456 (251 to 657)
Togo	521 (458 to 590)	533 (473 to 599)	8 (5 to 13)	6 (4 to 8)	38 (25 to 54)	38 (25 to 54)	418 (257 to 639)	278 (174 to 397)
Andean Latin America	4572 (4109 to 5062)	5394 (4860 to 5960)	58 (47 to 74)	58 (49 to 72)	293 (196 to 414)	375 (249 to 527)	1998 (1594 to 2594)	1683 (1413 to 2116)
Bolivia	614 (538 to 700)	733 (617 to 866)	14 (9 to 22)	17 (12 to 25)	35 (23 to 52)	47 (29 to 69)	495 (304 to 836)	555 (367 to 786)
Ecuador	1186 (1066 to 1313)	1352 (1211 to 1509)	17 (12 to 28)	17 (13 to 24)	75 (50 to 106)	92 (60 to 132)	620 (412 to 1036)	506 (388 to 716)
Peru	2773 (2486 to 3062)	3309 (2974 to 3659)	27 (18 to 37)	24 (17 to 31)	183 (123 to 265)	236 (159 to 334)	883 (609 to 1217)	621 (472 to 838)
Tropical Latin America	16618 (14941 to 18408)	19659 (17801 to 21588)	363 (288 to 613)	294 (247 to 455)	1009 (670 to 1426)	1195 (806 to 1699)	12430 (9618 to 20698)	7849 (6550 to 11799)
Brazil	16078 (14444 to 17822)	19129 (17317 to 21008)	357 (282 to 607)	290 (244 to 452)	973 (647 to 1382)	1160 (781 to 1652)	12207 (9382 to 20503)	7735 (6409 to 11709)
Paraguay	540 (482 to 605)	531 (478 to 588)	6 (4 to 8)	3 (2 to 5)	35 (24 to 50)	35 (23 to 50)	223 (152 to 302)	114 (73 to 158)
Central Latin America	21534 (19299 to 23842)	23433 (21113 to 25937)	301 (256 to 406)	196 (161 to 250)	1448 (986 to 2038)	1595 (1087 to 2270)	12328 (10640 to 16432)	7389 (5968 to 8953)
Colombia	4748 (4281 to 5236)	5081 (4572 to 5613)	60 (44 to 92)	41 (30 to 63)	323 (217 to 456)	349 (233 to 500)	2647 (1695 to 3810)	1802 (1138 to 2400)
Costa Rica	517 (461 to 573)	612 (542 to 680)	10 (7 to 17)	5 (4 to 9)	37 (25 to 51)	45 (30 to 64)	396 (248 to 522)	197 (136 to 246)
El Salvador	609 (543 to 685)	581 (525 to 641)	17 (10 to 23)	7 (4 to 9)	43 (29 to 62)	40 (27 to 57)	558 (367 to 763)	169 (108 to 231)
Guatemala	1396 (1243 to 1573)	1310 (1168 to 1460)	28 (21 to 37)	14 (10 to 18)	100 (67 to 141)	91 (61 to 129)	1188 (923 to 1739)	581 (430 to 772)
Honduras	707 (631 to 788)	677 (604 to 751)	17 (10 to 29)	12 (8 to 19)	48 (32 to 70)	46 (30 to 65)	657 (360 to 1129)	361 (222 to 576)
Mexico	10308 (9249 to 11504)	12050 (10829 to 13357)	129 (107 to 188)	101 (74 to 125)	688 (465 to 978)	829 (563 to 1181)	5294 (4333 to 8006)	3711 (2985 to 5071)
Nicaragua	437 (395 to 484)	457 (411 to 512)	3 (3 to 4)	3 (2 to 3)	28 (19 to 40)	29 (19 to 42)	140 (100 to 188)	95 (70 to 125)
Panama	286 (259 to 316)	326 (295 to 356)	3 (2 to 5)	3 (2 to 5)	17 (11 to 25)	21 (14 to 30)	121 (83 to 172)	88 (66 to 137)
Venezuela	2526 (2211 to 2841)	2340 (2090 to 2597)	33 (23 to 54)	11 (7 to 19)	163 (107 to 239)	145 (98 to 206)	1325 (950 to 2039)	384 (277 to 546)
Southern Latin America	3831 (3311 to 4354)	4489 (3860 to 5123)	132 (105 to 183)	116 (88 to 148)	250 (167 to 359)	326 (211 to 466)	4180 (3287 to 6124)	2795 (2228 to 3754)
Argentina	2601 (2206 to 3001)	3071 (2572 to 3560)	106 (82 to 149)	95 (71 to 125)	172 (113 to 252)	224 (144 to 326)	3381 (2551 to 5096)	2283 (1757 to 3192)
Chile	928 (828 to 1040)	1096 (966 to 1235)	15 (11 to 21)	14 (11 to 19)	57 (38 to 81)	77 (51 to 110)	459 (345 to 669)	370 (271 to 476)
Uruguay	302 (265 to 340)	321 (286 to 359)	11 (8 to 15)	7 (5 to 9)	22 (14 to 31)	24 (16 to 33)	339 (229 to 441)	142 (102 to 178)
Caribbean	4139 (3756 to 4533)	8628 (7763 to 9613)	90 (73 to 118)	330 (279 to 403)	268 (182 to 378)	634 (434 to 878)	3672 (2953 to 4833)	6857 (5426 to 8665)
Antigua and Barbuda	12 (11 to 13)	20 (17 to 22)	0 (0 to 1)	1 (1 to 2)	1 (1 to 1)	2 (1 to 2)	16 (12 to 21)	17 (14 to 23)
The Bahamas	26 (23 to 29)	36 (33 to 40)	1 (1 to 1)	1 (1 to 2)	2 (1 to 2)	2 (2 to 3)	32 (22 to 42)	25 (19 to 33)
Barbados	34 (31 to 37)	74 (65 to 84)	1 (1 to 1)	3 (2 to 4)	2 (1 to 3)	5 (4 to 8)	27 (20 to 36)	42 (33 to 55)
Belize	33 (30 to 37)	55 (49 to 61)	1 (1 to 2)	2 (2 to 3)	2 (2 to 3)	4 (3 to 6)	48 (37 to 66)	44 (35 to 62)
Bermuda	9 (8 to 10)	13 (11 to 14)	0 (0 to 0)	0 (0 to 0)	1 (0 to 1)	1 (1 to 1)	6 (4 to 8)	3 (2 to 4)
Cuba	1255 (1130 to 1388)	3094 (2729 to 3493)	13 (9 to 22)	84 (60 to 151)	76 (50 to 111)	227 (151 to 320)	410 (305 to 602)	1202 (854 to 2152)
Dominica	7 (6 to 7)	10 (9 to 11)	0 (0 to 0)	1 (1 to 1)	0 (0 to 1)	1 (0 to 1)	10 (8 to 14)	10 (8 to 15)
Dominican Republic	775 (698 to 859)	1758 (1556 to 1982)	10 (6 to 15)	63 (48 to 80)	50 (34 to 70)	132 (88 to 185)	407 (265 to 663)	1108 (843 to 1388)
Grenada	15 (14 to 17)	33 (28 to 38)	1 (0 to 1)	3 (2 to 3)	1 (1 to 2)	2 (2 to 4)	18 (14 to 25)	34 (27 to 44)
Guyana	87 (77 to 98)	124 (110 to 140)	7 (4 to 9)	11 (8 to 13)	7 (4 to 9)	9 (6 to 13)	265 (169 to 349)	238 (185 to 305)
Haiti	811 (728 to 902)	1111 (1000 to 1232)	34 (20 to 54)	84 (47 to 133)	55 (37 to 78)	79 (54 to 111)	1606 (1014 to 2377)	2912 (1621 to 4782)
Jamaica	235 (212 to 260)	425 (380 to 472)	3 (2 to 6)	14 (10 to 20)	15 (10 to 22)	31 (21 to 43)	135 (84 to 234)	243 (181 to 333)
Puerto Rico	417 (376 to 461)	1094 (956 to 1245)	6 (5 to 10)	31 (24 to 41)	27 (18 to 39)	81 (55 to 116)	184 (142 to 285)	393 (308 to 521)
Saint Lucia	20 (19 to 22)	38 (33 to 43)	1 (1 to 1)	2 (2 to 3)	1 (1 to 2)	3 (2 to 4)	31 (23 to 41)	34 (27 to 43)

Saint Vincent and the Grenadines	14 (13 to 16)	17 (15 to 19)	1 (0 to 1)	1 (1 to 1)	1 (1 to 1)	1 (1 to 2)	20 (16 to 26)	13 (10 to 18)
Suriname	49 (44 to 54)	98 (87 to 111)	2 (1 to 2)	5 (4 to 7)	3 (2 to 5)	7 (5 to 10)	63 (47 to 81)	97 (77 to 129)
Trinidad and Tobago	175 (157 to 194)	293 (258 to 329)	6 (4 to 8)	10 (7 to 14)	12 (8 to 18)	22 (15 to 30)	237 (168 to 319)	176 (126 to 238)
Virgin Islands, U.S.	15 (13 to 17)	25 (22 to 29)	1 (1 to 1)	1 (1 to 2)	1 (1 to 1)	2 (1 to 3)	24 (16 to 33)	18 (13 to 23)
Central Europe	27394 (24547 to 30450)	41621 (36949 to 47055)	1553 (1215 to 1825)	1847 (1571 to 2592)	2005 (1371 to 2737)	3141 (2160 to 4371)	32063 (23306 to 37880)	23231 (19667 to 29626)
Albania	1881 (1578 to 2223)	2179 (1834 to 2545)	105 (72 to 140)	58 (41 to 78)	151 (101 to 218)	176 (117 to 248)	2067 (1532 to 2689)	870 (617 to 1180)
Bosnia and Herzegovina	1007 (882 to 1144)	1307 (1137 to 1496)	50 (24 to 79)	69 (37 to 102)	76 (50 to 108)	100 (67 to 141)	1002 (469 to 1545)	906 (497 to 1290)
Bulgaria	1845 (1612 to 2102)	2174 (1878 to 2528)	136 (109 to 194)	134 (102 to 178)	138 (90 to 196)	164 (109 to 233)	2873 (2372 to 3820)	1984 (1618 to 2446)
Croatia	1324 (1182 to 1484)	3196 (2685 to 3779)	76 (53 to 97)	202 (125 to 262)	99 (67 to 139)	245 (160 to 346)	1001 (803 to 1421)	1839 (1191 to 2301)
Czech Republic	2357 (2081 to 2673)	3965 (3442 to 4594)	77 (48 to 99)	112 (72 to 138)	172 (117 to 239)	301 (201 to 423)	1563 (976 to 2002)	1529 (1067 to 1842)
Hungary	1426 (1287 to 1575)	2714 (2412 to 3065)	48 (32 to 64)	155 (75 to 204)	88 (59 to 125)	187 (126 to 261)	809 (563 to 1036)	1698 (856 to 2169)
Macedonia	235 (213 to 258)	219 (198 to 242)	6 (3 to 10)	4 (3 to 7)	15 (10 to 22)	14 (9 to 20)	189 (72 to 332)	102 (67 to 130)
Montenegro	128 (116 to 142)	116 (104 to 129)	4 (2 to 6)	4 (2 to 6)	9 (6 to 12)	8 (5 to 11)	71 (40 to 102)	46 (29 to 65)
Poland	7020 (6302 to 7736)	12819 (11329 to 14479)	188 (102 to 401)	274 (120 to 863)	493 (335 to 689)	964 (654 to 1342)	3766 (2502 to 6671)	3318 (1918 to 8055)
Romania	6938 (5991 to 8005)	8882 (7543 to 10441)	769 (475 to 987)	674 (491 to 887)	531 (354 to 747)	682 (462 to 994)	16558 (8848 to 22547)	9018 (6390 to 11148)
Serbia	1452 (1315 to 1586)	1586 (1425 to 1752)	46 (26 to 88)	68 (26 to 192)	105 (72 to 147)	116 (77 to 162)	1185 (791 to 1627)	903 (478 to 2114)
Slovakia	1398 (1229 to 1577)	1937 (1703 to 2193)	30 (23 to 47)	37 (27 to 47)	103 (69 to 148)	146 (96 to 208)	723 (523 to 1109)	561 (439 to 765)
Slovenia	383 (345 to 422)	525 (468 to 585)	17 (10 to 25)	56 (15 to 93)	27 (19 to 37)	38 (26 to 53)	256 (155 to 340)	457 (145 to 717)
Eastern Europe	16562 (14904 to 18345)	29255 (26526 to 32417)	729 (565 to 1005)	1282 (868 to 2242)	975 (659 to 1379)	1706 (1174 to 2385)	19915 (15476 to 25637)	16007 (11871 to 24220)
Belarus	659 (589 to 733)	1046 (945 to 1158)	15 (5 to 35)	16 (10 to 29)	40 (26 to 58)	65 (43 to 93)	465 (189 to 889)	302 (142 to 480)
Estonia	97 (87 to 109)	145 (129 to 161)	2 (1 to 3)	2 (1 to 3)	6 (4 to 8)	8 (6 to 12)	46 (24 to 68)	32 (21 to 41)
Latvia	144 (129 to 161)	224 (198 to 253)	2 (1 to 3)	2 (1 to 5)	8 (5 to 11)	12 (8 to 18)	42 (26 to 70)	32 (23 to 52)
Lithuania	217 (195 to 243)	313 (282 to 351)	3 (2 to 7)	3 (2 to 5)	13 (8 to 18)	18 (12 to 26)	95 (65 to 150)	51 (39 to 64)
Moldova	273 (245 to 302)	383 (346 to 424)	3 (2 to 6)	2 (2 to 5)	17 (11 to 24)	24 (16 to 34)	115 (65 to 165)	63 (46 to 79)
Russian Federation	12228 (10989 to 13611)	21701 (19626 to 24135)	542 (387 to 745)	897 (611 to 1635)	716 (486 to 1017)	1252 (858 to 1743)	14547 (10352 to 18646)	11529 (8283 to 18570)
Ukraine	2943 (2631 to 3288)	5442 (4866 to 6075)	163 (125 to 228)	360 (226 to 560)	177 (117 to 254)	328 (221 to 475)	4607 (3394 to 6847)	3998 (3042 to 5413)
North Africa and Middle East	49473 (44554 to 54552)	52999 (47500 to 59114)	860 (697 to 1075)	827 (684 to 1014)	3448 (2327 to 4851)	3927 (2647 to 5526)	40749 (31373 to 51529)	45304 (37078 to 56840)
Afghanistan	1585 (1405 to 1792)	1738 (1526 to 1977)	22 (11 to 36)	46 (26 to 79)	100 (66 to 147)	121 (80 to 173)	986 (438 to 1649)	2566 (1413 to 4299)
Algeria	3701 (3323 to 4084)	3525 (3140 to 3939)	64 (32 to 118)	54 (40 to 74)	260 (175 to 372)	256 (171 to 361)	2467 (1272 to 4145)	2614 (1855 to 3771)
Bahrain	139 (122 to 157)	87 (77 to 97)	1 (0 to 1)	0 (0 to 0)	9 (6 to 13)	6 (4 to 9)	27 (15 to 40)	15 (11 to 21)
Egypt	6746 (6062 to 7503)	8198 (7263 to 9200)	105 (47 to 195)	125 (79 to 192)	453 (304 to 647)	614 (407 to 871)	5016 (2335 to 8726)	6680 (4121 to 10409)
Iran	6632 (5939 to 7325)	8379 (7368 to 9471)	120 (96 to 156)	111 (93 to 145)	446 (301 to 631)	627 (418 to 885)	4700 (3563 to 6303)	4679 (3975 to 6087)
Iraq	5207 (4610 to 5841)	4989 (4383 to 5622)	164 (125 to 211)	163 (128 to 211)	404 (273 to 562)	395 (263 to 557)	10337 (7008 to 13973)	11055 (8184 to 14766)
Jordan	911 (796 to 1030)	867 (750 to 986)	9 (7 to 11)	4 (3 to 5)	66 (44 to 95)	66 (44 to 95)	401 (303 to 506)	206 (156 to 268)
Kuwait	477 (419 to 545)	506 (432 to 586)	6 (4 to 8)	4 (2 to 5)	36 (24 to 50)	40 (25 to 59)	287 (220 to 392)	197 (124 to 246)
Lebanon	668 (597 to 738)	735 (653 to 823)	8 (5 to 13)	5 (3 to 7)	47 (31 to 66)	53 (36 to 75)	325 (217 to 518)	154 (99 to 237)
Libya	554 (496 to 617)	593 (523 to 667)	12 (7 to 20)	10 (6 to 16)	39 (26 to 55)	44 (30 to 64)	743 (401 to 1269)	567 (307 to 993)
Morocco	2874 (2592 to 3170)	2806 (2512 to 3114)	48 (31 to 73)	39 (27 to 60)	191 (125 to 271)	192 (127 to 273)	1893 (1208 to 2908)	1776 (1199 to 2736)
Palestine	338 (300 to 380)	361 (321 to 403)	6 (4 to 8)	5 (3 to 6)	24 (16 to 34)	27 (18 to 38)	264 (177 to 371)	217 (160 to 282)
Oman	615 (539 to 695)	314 (276 to 354)	9 (6 to 13)	5 (4 to 7)	47 (31 to 66)	24 (16 to 34)	558 (367 to 769)	352 (238 to 505)
Qatar	345 (302 to 390)	121 (105 to 139)	2 (1 to 4)	0 (0 to 1)	25 (16 to 36)	9 (6 to 13)	122 (65 to 240)	28 (20 to 38)
Saudi Arabia	4974 (4343 to 5617)	5094 (4416 to 5796)	69 (46 to 99)	55 (40 to 76)	388 (257 to 545)	414 (269 to 615)	2959 (1980 to 4295)	2512 (1795 to 3477)
Sudan	2392 (2138 to 2672)	2357 (2074 to 2669)	48 (32 to 71)	67 (40 to 104)	160 (107 to 228)	166 (112 to 236)	2461 (1414 to 4022)	4690 (2741 to 7626)
Syria	1537 (1372 to 1721)	1757 (1552 to 1975)	29 (19 to 41)	16 (12 to 22)	108 (72 to 153)	133 (90 to 189)	1264 (772 to 1847)	793 (557 to 1093)
Tunisia	1083 (978 to 1192)	1101 (981 to 1234)	18 (10 to 29)	10 (7 to 14)	75 (51 to 106)	78 (52 to 111)	555 (282 to 906)	295 (202 to 440)
Turkey	5501 (4942 to 6074)	6868 (6214 to 7555)	64 (49 to 84)	45 (31 to 57)	347 (230 to 503)	466 (312 to 676)	2675 (1915 to 3878)	1829 (1397 to 2579)
United Arab Emirates	1364 (1162 to 1614)	578 (494 to 670)	20 (11 to 39)	5 (3 to 9)	100 (65 to 146)	46 (29 to 68)	964 (557 to 1757)	249 (148 to 428)
Yemen	1784 (1589 to 2000)	1977 (1737 to 2231)	34 (21 to 50)	56 (33 to 92)	121 (80 to 172)	144 (95 to 204)	1707 (1074 to 2712)	3788 (2070 to 6636)
Central Asia	8838 (7830 to 9878)	11220 (10066 to 12455)	442 (364 to 534)	350 (284 to 418)	677 (458 to 940)	858 (583 to 1203)	17363 (14503 to 21229)	12291 (10551 to 15700)

Armenia	204 (183 to 227)	284 (255 to 313)	3 (2 to 5)	3 (2 to 7)	13 (9 to 18)	18 (12 to 26)	79 (49 to 169)	54 (38 to 101)
Azerbaijan	1265 (1108 to 1441)	1451 (1290 to 1620)	80 (54 to 132)	42 (29 to 62)	99 (64 to 145)	113 (76 to 157)	2884 (1900 to 4808)	1357 (956 to 2105)
Georgia	608 (529 to 702)	705 (621 to 796)	48 (14 to 72)	17 (6 to 23)	48 (31 to 68)	54 (35 to 78)	1624 (492 to 2432)	488 (182 to 670)
Kazakhstan	1420 (1268 to 1582)	2301 (2074 to 2536)	122 (49 to 170)	143 (108 to 188)	100 (68 to 140)	168 (115 to 233)	4476 (2106 to 6779)	4171 (3032 to 6590)
Kyrgyzstan	563 (500 to 636)	817 (728 to 918)	10 (5 to 14)	9 (5 to 12)	38 (26 to 55)	58 (38 to 85)	461 (269 to 631)	419 (233 to 578)
Mongolia	483 (415 to 550)	447 (394 to 504)	32 (20 to 43)	15 (9 to 20)	39 (26 to 54)	35 (23 to 50)	1206 (802 to 1671)	483 (340 to 622)
Tajikistan	476 (420 to 536)	624 (551 to 702)	7 (4 to 10)	11 (7 to 15)	32 (21 to 46)	44 (30 to 64)	362 (197 to 499)	518 (316 to 697)
Turkmenistan	389 (346 to 433)	451 (406 to 502)	21 (14 to 38)	16 (11 to 25)	29 (19 to 40)	33 (22 to 47)	930 (624 to 1657)	661 (484 to 1052)
Uzbekistan	3430 (2954 to 3935)	4140 (3584 to 4710)	119 (89 to 197)	94 (72 to 117)	280 (184 to 389)	334 (217 to 483)	5340 (4105 to 7753)	4139 (3152 to 5187)
South Asia	132690 (119158 to 146704)	166048 (148463 to 185580)	3569 (1852 to 6808)	3462 (2106 to 4634)	9281 (6301 to 13038)	12362 (8412 to 17585)	131959 (71876 to 246861)	128668 (79976 to 173027)
Bangladesh	13752 (12287 to 15272)	16693 (14792 to 18705)	267 (121 to 483)	266 (155 to 419)	1006 (675 to 1407)	1296 (872 to 1825)	10265 (4332 to 19389)	10784 (6515 to 16670)
Bhutan	83 (75 to 93)	87 (77 to 97)	2 (1 to 4)	1 (1 to 2)	6 (4 to 9)	7 (4 to 9)	68 (32 to 146)	49 (32 to 71)
India	101143 (90645 to 112131)	127512 (113933 to 142615)	2759 (1356 to 5367)	2642 (1579 to 3637)	6991 (4767 to 9876)	9377 (6380 to 13300)	100775 (51731 to 190925)	93496 (57235 to 129517)
Nepal	2227 (1992 to 2488)	2510 (2208 to 2822)	39 (18 to 69)	22 (14 to 31)	160 (109 to 225)	185 (125 to 266)	1355 (646 to 2366)	768 (488 to 1093)
Pakistan	15485 (13912 to 17186)	19245 (16793 to 21767)	502 (238 to 1120)	530 (315 to 792)	1118 (753 to 1562)	1497 (996 to 2147)	19496 (9973 to 42240)	23571 (14038 to 34632)
Southeast Asia	68656 (61777 to 75965)	79290 (71342 to 87902)	1537 (1170 to 2219)	1075 (840 to 1256)	4708 (3204 to 6587)	5753 (3881 to 8082)	58617 (47028 to 78712)	40585 (33991 to 47226)
Cambodia	1250 (1117 to 1389)	1528 (1361 to 1705)	33 (23 to 45)	32 (19 to 48)	83 (56 to 119)	108 (73 to 156)	1520 (1015 to 2133)	1161 (706 to 1746)
Indonesia	27848 (24908 to 31068)	31099 (27607 to 34777)	570 (314 to 1120)	413 (274 to 537)	1914 (1300 to 2699)	2274 (1519 to 3233)	19467 (11563 to 36002)	16160 (11040 to 20735)
Laos	557 (498 to 625)	624 (550 to 701)	19 (13 to 31)	23 (14 to 35)	37 (25 to 54)	45 (30 to 65)	873 (563 to 1254)	1371 (802 to 2161)
Malaysia	3114 (2780 to 3468)	3331 (2991 to 3726)	26 (19 to 38)	20 (15 to 27)	210 (140 to 291)	243 (162 to 350)	1261 (891 to 1750)	988 (740 to 1319)
Maldives	56 (50 to 63)	29 (26 to 32)	1 (1 to 1)	0 (0 to 0)	4 (3 to 6)	2 (1 to 3)	29 (23 to 36)	6 (5 to 8)
Mauritius	109 (97 to 122)	130 (117 to 144)	1 (1 to 3)	1 (1 to 2)	6 (4 to 9)	8 (6 to 12)	53 (36 to 99)	37 (28 to 59)
Myanmar	4433 (3978 to 4927)	4779 (4289 to 5321)	148 (97 to 225)	55 (33 to 78)	289 (192 to 408)	318 (215 to 452)	6772 (4343 to 9725)	2038 (1283 to 2843)
Philippines	8145 (7232 to 9191)	9051 (8065 to 10116)	246 (182 to 383)	248 (183 to 391)	534 (359 to 759)	637 (427 to 911)	11180 (8154 to 17675)	10716 (8050 to 16616)
Sri Lanka	3247 (2922 to 3600)	4295 (3843 to 4756)	128 (81 to 180)	64 (44 to 88)	238 (160 to 335)	329 (223 to 466)	4408 (2686 to 6100)	2134 (1486 to 2902)
Seychelles	11 (10 to 12)	11 (10 to 12)	0 (0 to 0)	0 (0 to 0)	1 (0 to 1)	1 (1 to 1)	13 (10 to 17)	8 (7 to 11)
Thailand	9890 (8831 to 11039)	13916 (12247 to 15605)	122 (97 to 163)	84 (69 to 104)	708 (471 to 1003)	1064 (704 to 1501)	3924 (3096 to 5509)	2422 (1932 to 3232)
Timor-Leste	120 (107 to 134)	120 (106 to 135)	3 (1 to 7)	3 (1 to 4)	8 (6 to 12)	9 (6 to 13)	122 (56 to 235)	141 (75 to 213)
Vietnam	9785 (8793 to 10777)	10273 (9226 to 11428)	237 (139 to 341)	131 (83 to 191)	668 (447 to 940)	706 (472 to 993)	8917 (5152 to 13015)	3349 (2253 to 4806)
East Asia	246735 (220954 to 273886)	286177 (253413 to 319928)	7873 (5888 to 8894)	7495 (6267 to 8279)	18120 (12485 to 25319)	22053 (15076 to 30914)	216948 (167507 to 243470)	158723 (135817 to 174654)
China	235421 (210568 to 261792)	272513 (241068 to 305338)	7584 (5679 to 8581)	7183 (6031 to 7951)	17282 (11854 to 24216)	21001 (14343 to 29479)	207766 (160418 to 233849)	150674 (129262 to 166770)
North Korea	3008 (2679 to 3347)	4131 (3649 to 4603)	84 (55 to 118)	131 (88 to 178)	220 (150 to 312)	316 (210 to 441)	3165 (2010 to 4372)	3916 (2573 to 5473)
Taiwan (Province of China)	4331 (3983 to 4689)	4922 (4530 to 5367)	77 (41 to 102)	62 (31 to 80)	325 (213 to 459)	380 (254 to 538)	2522 (1339 to 3308)	1577 (903 to 1984)
Oceania	1025 (922 to 1136)	1255 (1116 to 1404)	79 (59 to 104)	110 (72 to 163)	70 (47 to 99)	94 (63 to 132)	2888 (2156 to 3868)	5047 (3186 to 7756)
American Samoa	4 (4 to 5)	6 (5 to 6)	0 (0 to 0)	0 (0 to 0)	0 (0 to 0)	0 (0 to 1)	4 (3 to 5)	5 (3 to 6)
Federated States of Micronesia	10 (9 to 12)	16 (14 to 18)	1 (0 to 1)	1 (1 to 2)	1 (1 to 1)	1 (1 to 2)	23 (14 to 32)	34 (20 to 54)
Fiji	144 (126 to 163)	170 (148 to 193)	14 (10 to 19)	12 (9 to 15)	11 (7 to 15)	13 (9 to 18)	439 (329 to 597)	384 (303 to 497)
Guam	20 (18 to 22)	29 (26 to 33)	1 (1 to 1)	1 (1 to 1)	1 (1 to 2)	2 (1 to 3)	26 (20 to 33)	27 (21 to 33)
Kiribati	14 (12 to 16)	15 (13 to 17)	2 (1 to 3)	1 (1 to 1)	1 (1 to 1)	1 (1 to 2)	82 (52 to 128)	36 (24 to 56)
Marshall Islands	5 (4 to 5)	8 (7 to 9)	0 (0 to 1)	1 (0 to 1)	0 (0 to 0)	1 (0 to 1)	13 (8 to 20)	29 (17 to 44)
Northern Mariana Islands	7 (6 to 7)	10 (9 to 11)	0 (0 to 0)	0 (0 to 0)	0 (0 to 1)	1 (1 to 1)	5 (4 to 7)	5 (4 to 6)
Papua New Guinea	651 (580 to 728)	801 (707 to 899)	50 (33 to 74)	81 (49 to 132)	43 (29 to 61)	60 (40 to 85)	1930 (1267 to 2824)	4022 (2333 to 6555)
Samoa	23 (21 to 26)	31 (28 to 35)	1 (1 to 2)	1 (1 to 2)	2 (1 to 2)	2 (2 to 3)	34 (20 to 56)	41 (26 to 59)
Solomon Islands	48 (43 to 53)	62 (55 to 69)	2 (1 to 3)	3 (2 to 5)	3 (2 to 5)	5 (3 to 6)	69 (42 to 103)	114 (64 to 179)
Tonga	10 (9 to 11)	8 (8 to 9)	0 (0 to 0)	0 (0 to 0)	1 (0 to 1)	1 (0 to 1)	9 (6 to 12)	2 (1 to 2)
Vanuatu	32 (28 to 36)	30 (27 to 34)	3 (1 to 8)	2 (1 to 3)	2 (2 to 3)	2 (1 to 3)	97 (34 to 240)	70 (44 to 115)
High-income Asia Pacific	47235 (42502 to 52287)	77588 (69157 to 86253)	460 (338 to 737)	1319 (1055 to 2106)	3596 (2476 to 4993)	6027 (4187 to 8328)	12206 (9477 to 18315)	14206 (11466 to 22152)

Brunei	68 (60 to 77)	65 (57 to 73)	2 (1 to 2)	1 (1 to 2)	5 (3 to 8)	5 (3 to 7)	99 (71 to 126)	53 (39 to 66)
Japan	37559 (33821 to 41512)	62830 (55783 to 70412)	389 (285 to 626)	1142 (891 to 1869)	2860 (1972 to 3961)	4871 (3355 to 6683)	9509 (7300 to 14432)	11068 (8565 to 18258)
Singapore	1911 (1647 to 2219)	2327 (2041 to 2627)	26 (19 to 44)	20 (15 to 31)	158 (101 to 228)	192 (123 to 275)	1168 (830 to 1554)	705 (560 to 906)
South Korea	7697 (6803 to 8702)	12365 (10863 to 13931)	42 (29 to 69)	156 (116 to 207)	573 (370 to 834)	958 (625 to 1383)	1431 (971 to 2287)	2380 (1777 to 3018)
High-income North America	26364 (24158 to 28529)	31760 (29094 to 34468)	646 (389 to 770)	589 (358 to 694)	1948 (1333 to 2672)	2547 (1745 to 3533)	25765 (16313 to 30431)	21219 (13944 to 24346)
Canada	3353 (2991 to 3706)	5491 (4795 to 6168)	45 (22 to 62)	44 (21 to 58)	257 (174 to 353)	452 (290 to 659)	1676 (892 to 2259)	1422 (798 to 1824)
Greenland	4 (4 to 5)	7 (6 to 8)	0 (0 to 0)	0 (0 to 0)	0 (0 to 0)	1 (0 to 1)	6 (3 to 7)	10 (6 to 13)
United States	23006 (21117 to 24952)	26261 (24149 to 28538)	601 (364 to 717)	545 (335 to 643)	1690 (1151 to 2332)	2095 (1438 to 2898)	24083 (15365 to 28343)	19787 (13119 to 22720)
Western Europe	63867 (58273 to 69966)	85851 (77763 to 94891)	1959 (1197 to 2388)	4332 (2174 to 5387)	4547 (3129 to 6276)	6224 (4270 to 8584)	30439 (21546 to 34575)	37310 (23074 to 43815)
Andorra	13 (12 to 14)	15 (13 to 17)	0 (0 to 1)	0 (0 to 1)	1 (1 to 1)	1 (1 to 2)	7 (4 to 11)	5 (3 to 7)
Austria	2200 (2005 to 2409)	2202 (1999 to 2414)	51 (33 to 120)	82 (51 to 216)	164 (111 to 232)	162 (107 to 229)	772 (566 to 1234)	835 (579 to 1485)
Belgium	1592 (1425 to 1760)	2234 (1974 to 2516)	56 (27 to 79)	109 (52 to 156)	116 (79 to 164)	167 (113 to 236)	740 (469 to 948)	958 (574 to 1256)
Cyprus	210 (187 to 233)	269 (239 to 301)	6 (3 to 8)	9 (5 to 12)	16 (10 to 22)	20 (14 to 29)	118 (84 to 150)	88 (59 to 113)
Denmark	944 (842 to 1049)	1182 (1044 to 1323)	29 (20 to 37)	41 (28 to 50)	71 (47 to 101)	90 (60 to 127)	482 (358 to 599)	396 (313 to 484)
Finland	1248 (1108 to 1406)	2132 (1878 to 2380)	36 (26 to 59)	32 (23 to 52)	94 (64 to 132)	163 (109 to 229)	840 (637 to 1079)	563 (457 to 713)
France	6660 (6038 to 7319)	8885 (8030 to 9868)	141 (71 to 201)	283 (132 to 412)	435 (296 to 618)	604 (407 to 856)	2253 (1160 to 3019)	2670 (1367 to 3607)
Germany	13808 (12346 to 15257)	19399 (17265 to 21796)	505 (362 to 864)	911 (631 to 1703)	1009 (689 to 1405)	1406 (935 to 1975)	8800 (6816 to 12479)	8559 (6429 to 14303)
Greece	1230 (1110 to 1361)	1747 (1571 to 1952)	28 (16 to 38)	80 (45 to 115)	82 (54 to 115)	124 (83 to 175)	388 (280 to 538)	738 (457 to 981)
Iceland	61 (54 to 67)	63 (56 to 70)	1 (1 to 2)	2 (1 to 2)	5 (3 to 7)	5 (3 to 7)	20 (16 to 26)	14 (11 to 17)
Ireland	693 (620 to 766)	798 (718 to 885)	23 (9 to 34)	48 (16 to 72)	51 (34 to 73)	60 (40 to 83)	416 (157 to 618)	470 (147 to 677)
Israel	1225 (1097 to 1357)	1205 (1069 to 1334)	15 (11 to 24)	16 (11 to 29)	94 (64 to 132)	91 (60 to 129)	406 (264 to 548)	273 (196 to 381)
Italy	11716 (10446 to 13083)	13516 (11939 to 15345)	396 (92 to 668)	1224 (240 to 1981)	836 (554 to 1190)	992 (663 to 1408)	3692 (1382 to 5547)	7439 (1831 to 11654)
Luxembourg	133 (118 to 149)	172 (152 to 194)	4 (2 to 5)	9 (6 to 12)	10 (7 to 14)	13 (9 to 19)	54 (33 to 77)	79 (50 to 103)
Malta	154 (135 to 176)	196 (171 to 223)	5 (3 to 6)	9 (6 to 12)	12 (8 to 17)	15 (10 to 22)	88 (64 to 109)	103 (67 to 129)
Netherlands	2687 (2404 to 2983)	3635 (3251 to 4090)	90 (63 to 114)	188 (110 to 246)	194 (133 to 276)	268 (184 to 376)	1259 (945 to 1741)	1674 (1118 to 2055)
Norway	989 (896 to 1080)	1266 (1128 to 1413)	22 (17 to 26)	47 (35 to 54)	71 (49 to 96)	95 (66 to 130)	373 (289 to 456)	426 (339 to 490)
Portugal	1112 (1003 to 1232)	2121 (1895 to 2367)	31 (17 to 43)	82 (48 to 112)	74 (50 to 104)	151 (103 to 213)	439 (295 to 569)	740 (482 to 949)
Spain	4737 (4284 to 5283)	6366 (5746 to 7030)	156 (75 to 226)	440 (139 to 698)	309 (208 to 436)	431 (289 to 601)	2065 (1220 to 2664)	3164 (1329 to 4611)
Sweden	2938 (2571 to 3325)	3602 (3164 to 4081)	74 (46 to 94)	98 (72 to 119)	227 (153 to 317)	272 (182 to 386)	1526 (780 to 2069)	1131 (747 to 1362)
Switzerland	1075 (981 to 1176)	1611 (1464 to 1770)	22 (15 to 38)	48 (35 to 73)	78 (54 to 108)	122 (82 to 172)	370 (273 to 559)	451 (363 to 614)
United Kingdom	8379 (7586 to 9187)	13148 (11844 to 14498)	262 (182 to 300)	568 (341 to 667)	595 (411 to 827)	966 (673 to 1328)	5300 (3562 to 5986)	6495 (4143 to 7450)
Australasia	3663 (3309 to 4023)	5758 (5150 to 6366)	65 (51 to 91)	57 (43 to 78)	272 (185 to 378)	445 (298 to 632)	2174 (1695 to 3091)	1572 (1179 to 2144)
Australia	2915 (2621 to 3206)	4637 (4134 to 5147)	53 (40 to 76)	46 (34 to 63)	218 (147 to 305)	358 (236 to 514)	1729 (1286 to 2569)	1212 (883 to 1705)
New Zealand	748 (679 to 821)	1121 (996 to 1245)	12 (10 to 16)	11 (8 to 15)	54 (37 to 77)	87 (56 to 123)	445 (344 to 550)	361 (288 to 450)

Data in parentheses are 95% uncertainty intervals. YLDs= years lived with disability; YLLs= years of life lost; SDI= Socio-demographic Index.

Supplementary Table 3. Numbers of alcoholic cardiomyopathy prevalent cases, deaths, YLDs, and YLLs in 2017, by sex, SDI quintile, and location.

	Prevalence		Deaths		YLDs		YLLs	
	Males	Females	Males	Females	Males	Females	Males	Females
Global	1091128 (916345 to 1283362)	530648 (449265 to 621133)	68588 (57478 to 75218)	20302 (18716 to 24216)	93466 (63876 to 132144)	45621 (31122 to 63667)	2235046 (1902122 to 2440826)	614148 (566054 to 703031)
SDI Quintile								
Low SDI	53979 (43861 to 65247)	38085 (31434 to 45756)	2070 (1085 to 3663)	1172 (630 to 1701)	4712 (3149 to 6661)	3356 (2248 to 4828)	65698 (33973 to 116707)	36482 (17947 to 52711)
Low-middle SDI	79790 (65238 to 95740)	44151 (36875 to 52681)	3670 (2822 to 4802)	1444 (1025 to 1989)	7039 (4679 to 10125)	3958 (2632 to 5641)	116506 (90076 to 151610)	42769 (28262 to 58585)
Middle SDI	115336 (94980 to 137051)	50754 (42217 to 60491)	5900 (4658 to 7568)	1341 (1141 to 1665)	10318 (6885 to 14862)	4579 (3056 to 6572)	181823 (143763 to 233054)	32761 (28255 to 39026)
High-middle SDI	274146 (226717 to 326702)	223174 (186736 to 264584)	42859 (36598 to 48989)	13885 (13004 to 16828)	23448 (16015 to 33123)	18842 (12833 to 26265)	1518530 (1315559 to 1731645)	446743 (421536 to 524707)
High SDI	566003 (481127 to 659209)	173887 (149946 to 199906)	14016 (10671 to 15520)	2446 (2223 to 2871)	47781 (32825 to 66728)	14831 (10105 to 20642)	350248 (276755 to 381519)	55016 (50401 to 60790)
Central Sub-Saharan Africa	6694 (5259 to 8302)	4712 (3741 to 5767)	236 (109 to 396)	139 (72 to 223)	569 (371 to 829)	406 (257 to 590)	7968 (3736 to 13575)	4706 (2360 to 7437)
Angola	1581 (1235 to 1992)	1168 (911 to 1448)	62 (30 to 113)	31 (17 to 50)	134 (82 to 198)	100 (59 to 150)	2114 (1040 to 3912)	1063 (573 to 1726)
Central African Republic	277 (214 to 347)	218 (169 to 269)	11 (4 to 27)	11 (4 to 20)	23 (14 to 35)	18 (11 to 29)	397 (133 to 1001)	390 (134 to 747)
Congo	366 (283 to 455)	232 (182 to 285)	14 (8 to 26)	9 (4 to 16)	31 (19 to 47)	20 (12 to 29)	478 (261 to 850)	343 (137 to 601)
Democratic Republic of the Congo	4220 (3269 to 5299)	2927 (2279 to 3624)	138 (51 to 260)	85 (37 to 155)	359 (230 to 538)	253 (158 to 375)	4659 (1723 to 8776)	2808 (1158 to 4933)
Equatorial Guinea	76 (59 to 94)	63 (50 to 78)	3 (1 to 4)	1 (1 to 3)	7 (4 to 9)	5 (3 to 8)	88 (44 to 152)	45 (19 to 104)
Gabon	175 (134 to 218)	103 (82 to 126)	7 (5 to 11)	2 (1 to 3)	15 (9 to 22)	9 (5 to 13)	231 (154 to 343)	57 (34 to 96)
Eastern Sub-Saharan Africa	21982 (17357 to 27241)	15470 (12361 to 18988)	741 (433 to 1575)	234 (119 to 428)	1878 (1240 to 2678)	1334 (880 to 1917)	25227 (15128 to 54037)	7455 (3946 to 12853)
Burundi	646 (487 to 823)	647 (496 to 808)	25 (11 to 58)	14 (6 to 27)	55 (32 to 85)	56 (34 to 82)	890 (386 to 2062)	461 (190 to 908)
Comoros	44 (33 to 56)	27 (21 to 34)	1 (0 to 3)	0 (0 to 1)	4 (2 to 6)	2 (1 to 4)	45 (15 to 107)	14 (5 to 29)
Djibouti	70 (53 to 90)	28 (22 to 34)	3 (1 to 7)	0 (0 to 1)	6 (4 to 9)	2 (2 to 4)	94 (30 to 236)	16 (6 to 35)
Eritrea	180 (137 to 228)	140 (108 to 177)	13 (4 to 29)	5 (2 to 9)	15 (10 to 23)	12 (8 to 19)	502 (166 to 1109)	151 (52 to 299)
Ethiopia	9403 (7473 to 11408)	5789 (4747 to 6959)	134 (46 to 324)	35 (15 to 72)	799 (522 to 1136)	493 (321 to 709)	4230 (1510 to 10357)	1107 (499 to 2195)
Kenya	1752 (1321 to 2217)	1100 (847 to 1370)	111 (64 to 180)	28 (13 to 50)	152 (98 to 223)	97 (63 to 142)	3765 (2194 to 6102)	891 (410 to 1607)
Madagascar	1106 (847 to 1419)	701 (536 to 880)	86 (36 to 202)	34 (13 to 66)	95 (59 to 143)	62 (39 to 93)	3224 (1368 to 7734)	1194 (461 to 2229)
Malawi	718 (549 to 914)	546 (423 to 689)	25 (12 to 58)	7 (2 to 15)	62 (38 to 91)	47 (30 to 71)	851 (418 to 1960)	183 (63 to 406)
Mozambique	660 (494 to 862)	622 (477 to 792)	60 (22 to 129)	20 (7 to 41)	57 (34 to 88)	55 (34 to 83)	2149 (765 to 4640)	656 (236 to 1273)
Rwanda	635 (487 to 803)	730 (563 to 917)	24 (12 to 48)	9 (4 to 19)	54 (33 to 82)	63 (38 to 95)	782 (401 to 1562)	270 (118 to 548)
Somalia	649 (496 to 832)	472 (364 to 599)	25 (6 to 67)	13 (5 to 31)	56 (34 to 83)	41 (25 to 62)	867 (199 to 2335)	441 (151 to 1001)
South Sudan	417 (319 to 531)	227 (176 to 286)	14 (3 to 37)	6 (2 to 15)	35 (22 to 53)	20 (12 to 30)	474 (116 to 1269)	208 (66 to 483)
Tanzania	3156 (2412 to 4020)	2013 (1566 to 2542)	120 (52 to 285)	34 (16 to 67)	269 (165 to 399)	175 (110 to 255)	3869 (1789 to 9309)	987 (471 to 1841)
Uganda	1879 (1436 to 2373)	1984 (1550 to 2462)	71 (37 to 152)	21 (9 to 42)	160 (97 to 241)	170 (105 to 254)	2448 (1274 to 5343)	641 (274 to 1298)
Zambia	656 (502 to 841)	434 (335 to 549)	30 (15 to 54)	7 (3 to 14)	57 (35 to 86)	38 (23 to 57)	1022 (514 to 1875)	231 (105 to 432)
Southern Sub-Saharan Africa	6464 (5197 to 7765)	6704 (5381 to 8243)	217 (174 to 263)	182 (154 to 220)	539 (358 to 767)	559 (366 to 811)	5146 (4092 to 6316)	3576 (2924 to 4223)
Botswana	179 (141 to 221)	102 (81 to 126)	5 (2 to 9)	3 (2 to 5)	15 (10 to 22)	9 (5 to 13)	107 (50 to 214)	50 (28 to 83)
Lesotho	114 (89 to 144)	99 (76 to 125)	5 (3 to 8)	5 (2 to 10)	9 (6 to 14)	8 (5 to 12)	129 (65 to 203)	98 (37 to 211)
Namibia	165 (129 to 204)	109 (86 to 135)	7 (3 to 11)	2 (1 to 5)	14 (8 to 20)	9 (6 to 14)	154 (74 to 285)	36 (16 to 71)
South Africa	5701 (4589 to 6842)	5955 (4742 to 7344)	181 (141 to 224)	145 (122 to 182)	475 (315 to 677)	495 (323 to 716)	4168 (3227 to 5294)	2586 (2129 to 3153)
Swaziland	57 (44 to 71)	46 (35 to 58)	2 (1 to 3)	1 (1 to 3)	5 (3 to 7)	4 (2 to 6)	60 (30 to 95)	28 (11 to 57)
Zimbabwe	249 (193 to 312)	393 (307 to 487)	18 (10 to 30)	26 (15 to 39)	22 (13 to 34)	35 (22 to 51)	529 (306 to 852)	778 (423 to 1197)
Western Sub-Saharan Africa	36217 (28661 to 44732)	10447 (8256 to 12954)	667 (409 to 1080)	139 (106 to 183)	3075 (2007 to 4499)	903 (579 to 1324)	21575 (13340 to 35438)	4181 (3155 to 5454)
Benin	539 (416 to 679)	239 (186 to 298)	10 (3 to 22)	4 (2 to 6)	46 (28 to 70)	21 (13 to 31)	315 (98 to 703)	113 (52 to 187)
Burkina Faso	1309 (1005 to 1645)	751 (584 to 947)	29 (12 to 62)	13 (5 to 19)	112 (68 to 166)	64 (38 to 98)	906 (375 to 1892)	362 (127 to 541)
Cameroon	1760 (1356 to 2215)	816 (641 to 1018)	49 (29 to 78)	13 (7 to 22)	150 (90 to 227)	70 (43 to 106)	1677 (1017 to 2718)	416 (224 to 691)
Cape Verde	38 (30 to 48)	20 (16 to 25)	0 (0 to 1)	0 (0 to 0)	3 (2 to 5)	2 (1 to 3)	14 (10 to 20)	2 (2 to 3)
Chad	864 (665 to 1087)	299 (233 to 375)	15 (5 to 32)	5 (2 to 8)	73 (45 to 109)	26 (16 to 38)	482 (172 to 984)	161 (55 to 267)

Cote d'Ivoire	1803 (1398 to 2257)	577 (445 to 722)	43 (18 to 75)	8 (4 to 14)	154 (94 to 234)	50 (31 to 75)	1504 (629 to 2644)	255 (124 to 454)
The Gambia	137 (107 to 172)	55 (43 to 68)	3 (1 to 5)	1 (0 to 1)	12 (7 to 17)	5 (3 to 7)	92 (34 to 166)	20 (11 to 32)
Ghana	2451 (1892 to 3123)	783 (604 to 993)	103 (62 to 182)	37 (25 to 53)	209 (127 to 314)	68 (41 to 105)	3327 (2060 to 5568)	1018 (702 to 1499)
Guinea	503 (380 to 652)	172 (131 to 221)	13 (4 to 25)	5 (2 to 9)	43 (26 to 65)	15 (9 to 23)	394 (137 to 793)	155 (54 to 304)
Guinea-Bissau	96 (74 to 119)	53 (41 to 67)	2 (1 to 5)	1 (0 to 2)	8 (5 to 12)	5 (3 to 7)	83 (35 to 188)	33 (14 to 56)
Liberia	364 (282 to 458)	168 (129 to 211)	5 (1 to 11)	2 (1 to 3)	31 (19 to 45)	14 (9 to 22)	177 (50 to 363)	56 (22 to 91)
Mali	1095 (839 to 1361)	409 (318 to 510)	17 (3 to 39)	7 (2 to 13)	93 (57 to 140)	35 (22 to 54)	514 (92 to 1181)	252 (82 to 467)
Mauritania	273 (212 to 346)	83 (65 to 105)	4 (1 to 10)	1 (0 to 2)	23 (15 to 35)	7 (4 to 11)	129 (33 to 297)	28 (12 to 60)
Niger	945 (739 to 1198)	351 (275 to 434)	13 (2 to 32)	5 (2 to 12)	82 (51 to 123)	31 (19 to 48)	404 (79 to 1018)	178 (52 to 373)
Nigeria	21731 (17128 to 27109)	4629 (3648 to 5785)	324 (132 to 741)	27 (15 to 45)	1839 (1173 to 2770)	403 (249 to 602)	10405 (4208 to 24120)	789 (442 to 1289)
Sao Tome and Principe	20 (15 to 25)	11 (8 to 13)	0 (0 to 1)	0 (0 to 0)	2 (1 to 3)	1 (1 to 1)	13 (7 to 23)	3 (2 to 5)
Senegal	1170 (921 to 1463)	422 (332 to 524)	14 (4 to 28)	4 (2 to 7)	99 (62 to 147)	36 (23 to 53)	421 (114 to 856)	103 (46 to 204)
Sierra Leone	663 (515 to 818)	403 (315 to 507)	13 (6 to 21)	5 (2 to 8)	57 (35 to 83)	34 (21 to 50)	418 (201 to 710)	166 (65 to 258)
Togo	456 (356 to 573)	208 (162 to 265)	9 (5 to 15)	2 (1 to 4)	39 (25 to 59)	18 (11 to 27)	301 (152 to 520)	69 (34 to 122)
Andean Latin America	1080 (859 to 1352)	808 (653 to 994)	27 (21 to 33)	18 (14 to 22)	100 (64 to 148)	74 (47 to 110)	719 (542 to 910)	389 (318 to 467)
Bolivia	71 (44 to 110)	124 (82 to 168)	7 (4 to 11)	6 (4 to 9)	7 (3 to 12)	11 (6 to 18)	183 (97 to 299)	139 (89 to 202)
Ecuador	184 (148 to 229)	168 (135 to 210)	9 (6 to 12)	7 (5 to 9)	17 (11 to 25)	16 (10 to 23)	237 (166 to 316)	143 (111 to 179)
Peru	824 (665 to 1021)	515 (419 to 627)	11 (7 to 16)	5 (3 to 6)	76 (49 to 113)	48 (31 to 70)	299 (182 to 420)	107 (71 to 146)
Tropical Latin America	27690 (21187 to 34832)	7336 (5975 to 8788)	1241 (975 to 1797)	167 (128 to 190)	2397 (1496 to 3498)	668 (437 to 972)	40461 (32668 to 58122)	4457 (3443 to 5102)
Brazil	27471 (20999 to 34580)	7218 (5872 to 8666)	1235 (970 to 1789)	164 (126 to 187)	2377 (1484 to 3473)	658 (429 to 958)	40301 (32499 to 58012)	4414 (3409 to 5062)
Paraguay	219 (177 to 271)	118 (97 to 143)	6 (4 to 9)	2 (1 to 3)	19 (12 to 28)	11 (7 to 15)	160 (110 to 222)	43 (30 to 60)
Central Latin America	11032 (8746 to 13561)	5123 (4277 to 6051)	301 (217 to 377)	90 (80 to 121)	991 (638 to 1444)	471 (310 to 677)	9165 (6683 to 10875)	2173 (1961 to 2608)
Colombia	1581 (1308 to 1875)	1127 (936 to 1327)	19 (12 to 44)	7 (5 to 9)	146 (96 to 206)	104 (70 to 150)	532 (320 to 1359)	161 (116 to 206)
Costa Rica	455 (376 to 547)	150 (125 to 178)	13 (8 to 19)	3 (2 to 5)	40 (25 to 58)	13 (9 to 20)	373 (230 to 523)	54 (42 to 75)
El Salvador	50 (40 to 60)	47 (39 to 57)	2 (1 to 3)	1 (1 to 1)	5 (3 to 7)	4 (3 to 6)	49 (30 to 85)	25 (17 to 33)
Guatemala	185 (150 to 227)	162 (134 to 197)	5 (3 to 8)	4 (3 to 5)	17 (11 to 25)	15 (10 to 22)	166 (105 to 231)	105 (81 to 137)
Honduras	318 (256 to 390)	185 (151 to 225)	16 (9 to 25)	8 (5 to 12)	28 (18 to 43)	17 (11 to 25)	516 (278 to 867)	207 (124 to 308)
Mexico	5305 (4184 to 6459)	2476 (2031 to 2983)	134 (86 to 158)	41 (36 to 48)	482 (312 to 710)	229 (150 to 329)	4336 (2751 to 5096)	1096 (986 to 1296)
Nicaragua	264 (219 to 316)	147 (121 to 178)	5 (4 to 7)	4 (2 to 5)	24 (15 to 35)	14 (9 to 20)	147 (106 to 208)	89 (60 to 141)
Panama	171 (141 to 202)	148 (122 to 177)	5 (4 to 11)	4 (3 to 8)	15 (10 to 22)	13 (8 to 19)	145 (99 to 271)	72 (54 to 126)
Venezuela	2703 (1906 to 3619)	680 (555 to 823)	102 (66 to 149)	19 (13 to 34)	233 (131 to 365)	62 (40 to 90)	2901 (1941 to 3930)	364 (266 to 496)
Southern Latin America	7873 (5737 to 10185)	1540 (1221 to 1897)	194 (153 to 317)	21 (17 to 25)	671 (415 to 1020)	139 (88 to 203)	5216 (4058 to 8513)	443 (346 to 516)
Argentina	4551 (3190 to 5956)	475 (342 to 612)	104 (75 to 183)	6 (5 to 8)	389 (229 to 605)	44 (27 to 67)	2864 (2055 to 5024)	159 (105 to 205)
Chile	2308 (1649 to 3016)	601 (474 to 739)	52 (37 to 94)	6 (5 to 8)	197 (112 to 302)	56 (35 to 82)	1504 (1099 to 2562)	153 (116 to 195)
Uruguay	1013 (805 to 1277)	464 (370 to 577)	39 (28 to 50)	8 (6 to 11)	85 (54 to 126)	39 (25 to 60)	847 (609 to 1124)	130 (96 to 167)
Caribbean	10257 (8484 to 12321)	2688 (2263 to 3195)	543 (342 to 643)	99 (73 to 135)	884 (586 to 1289)	236 (154 to 338)	15085 (9430 to 18014)	2898 (2026 to 4180)
Antigua and Barbuda	5 (4 to 6)	2 (2 to 3)	0 (0 to 0)	0 (0 to 0)	0 (0 to 1)	0 (0 to 0)	10 (6 to 13)	3 (2 to 4)
The Bahamas	42 (34 to 51)	14 (11 to 17)	5 (4 to 7)	1 (1 to 1)	4 (2 to 6)	1 (1 to 2)	179 (123 to 230)	23 (17 to 29)
Barbados	89 (72 to 108)	33 (28 to 40)	5 (4 to 6)	1 (1 to 1)	8 (5 to 11)	3 (2 to 4)	132 (100 to 165)	22 (17 to 27)
Belize	14 (12 to 17)	12 (10 to 14)	1 (1 to 1)	0 (0 to 1)	1 (1 to 2)	1 (1 to 2)	36 (22 to 46)	15 (12 to 19)
Bermuda	8 (6 to 9)	8 (7 to 10)	0 (0 to 0)	0 (0 to 0)	1 (0 to 1)	1 (0 to 1)	9 (5 to 12)	2 (1 to 2)
Cuba	7474 (6111 to 9044)	1263 (1031 to 1536)	380 (207 to 469)	31 (24 to 39)	643 (415 to 946)	110 (67 to 169)	10367 (5497 to 12950)	844 (650 to 1075)
Dominica	5 (4 to 6)	2 (2 to 3)	1 (0 to 1)	0 (0 to 0)	0 (0 to 1)	0 (0 to 0)	19 (12 to 25)	4 (3 to 5)
Dominican Republic	282 (235 to 331)	132 (111 to 157)	13 (9 to 19)	5 (4 to 7)	26 (17 to 37)	12 (8 to 18)	411 (260 to 614)	124 (91 to 169)
Grenada	18 (15 to 22)	7 (6 to 8)	1 (1 to 1)	0 (0 to 1)	2 (1 to 2)	1 (0 to 1)	27 (21 to 35)	10 (8 to 12)
Guyana	55 (45 to 68)	35 (28 to 41)	6 (3 to 8)	2 (2 to 3)	5 (3 to 7)	3 (2 to 5)	191 (105 to 271)	64 (49 to 84)
Haiti	518 (425 to 626)	435 (359 to 527)	53 (25 to 90)	43 (19 to 75)	45 (28 to 66)	39 (25 to 57)	1631 (817 to 2754)	1446 (594 to 2601)
Jamaica	284 (233 to 350)	89 (74 to 107)	15 (9 to 27)	3 (2 to 4)	25 (15 to 36)	8 (5 to 12)	412 (234 to 809)	71 (51 to 101)
Puerto Rico	802 (652 to 981)	456 (374 to 551)	22 (16 to 33)	4 (3 to 5)	68 (44 to 100)	39 (25 to 58)	512 (361 to 879)	72 (44 to 92)
Saint Lucia	43 (36 to 52)	17 (14 to 20)	4 (2 to 4)	1 (1 to 1)	4 (2 to 5)	1 (1 to 2)	106 (66 to 133)	18 (14 to 22)
Saint Vincent and the	16 (13 to 19)	3 (2 to 3)	2 (1 to 2)	0 (0 to 0)	1 (1 to 2)	0 (0 to 0)	44 (30 to 55)	4 (3 to 5)

Grenadines								
Suriname	26 (21 to 32)	18 (15 to 23)	2 (1 to 3)	1 (1 to 1)	2 (2 to 3)	2 (1 to 3)	65 (43 to 86)	21 (16 to 27)
Trinidad and Tobago	164 (132 to 201)	47 (38 to 56)	10 (7 to 15)	2 (1 to 2)	14 (9 to 21)	4 (3 to 6)	265 (182 to 401)	38 (26 to 52)
Virgin Islands, U.S.	45 (37 to 56)	19 (16 to 22)	5 (3 to 6)	1 (0 to 1)	4 (2 to 6)	2 (1 to 2)	125 (89 to 162)	13 (9 to 16)
Central Europe	75176 (62861 to 88875)	33718 (28526 to 39774)	4286 (2832 to 5055)	912 (780 to 1193)	6252 (4222 to 8774)	2820 (1910 to 3974)	115507 (72720 to 132397)	19468 (17248 to 22902)
Albania	494 (405 to 593)	400 (330 to 474)	19 (12 to 28)	6 (2 to 9)	42 (27 to 61)	34 (21 to 49)	363 (214 to 559)	97 (40 to 155)
Bosnia and Herzegovina	2191 (1790 to 2644)	1044 (857 to 1262)	89 (40 to 151)	27 (17 to 40)	181 (118 to 261)	86 (55 to 125)	2201 (961 to 3761)	497 (311 to 720)
Bulgaria	427 (353 to 511)	288 (234 to 357)	26 (20 to 40)	8 (5 to 10)	39 (25 to 57)	27 (17 to 39)	702 (530 to 1046)	142 (110 to 176)
Croatia	3787 (3274 to 4361)	2189 (1768 to 2672)	172 (108 to 206)	39 (29 to 55)	310 (210 to 440)	178 (114 to 256)	3581 (2480 to 4181)	496 (359 to 644)
Czech Republic	3013 (2448 to 3669)	808 (654 to 984)	92 (30 to 123)	11 (9 to 15)	254 (161 to 379)	71 (44 to 115)	2773 (766 to 3752)	331 (261 to 471)
Hungary	14678 (12134 to 17759)	8623 (7079 to 10461)	912 (594 to 1051)	255 (184 to 298)	1213 (810 to 1695)	713 (467 to 1029)	23542 (14937 to 27127)	5812 (4386 to 6740)
Macedonia	577 (479 to 691)	535 (438 to 649)	26 (13 to 41)	16 (9 to 30)	48 (31 to 69)	44 (29 to 64)	533 (253 to 880)	250 (167 to 405)
Montenegro	1678 (1402 to 2017)	1826 (1510 to 2211)	60 (41 to 81)	53 (37 to 69)	138 (91 to 196)	148 (99 to 211)	1305 (894 to 1806)	767 (539 to 976)
Poland	28794 (23500 to 34550)	7729 (6228 to 9482)	1736 (815 to 2123)	208 (164 to 256)	2401 (1582 to 3496)	664 (400 to 988)	49835 (22798 to 60139)	5947 (4683 to 7266)
Romania	13650 (11269 to 16520)	6067 (4865 to 7428)	879 (667 to 1262)	130 (106 to 181)	1138 (748 to 1638)	510 (331 to 750)	24010 (18340 to 34602)	3149 (2558 to 4303)
Serbia	2077 (1758 to 2448)	2089 (1673 to 2591)	135 (70 to 287)	122 (44 to 351)	173 (114 to 251)	170 (107 to 250)	2821 (1633 to 4883)	1367 (586 to 3614)
Slovakia	2001 (1620 to 2431)	602 (504 to 711)	119 (76 to 148)	14 (11 to 18)	167 (107 to 245)	52 (33 to 78)	3503 (2244 to 4288)	400 (301 to 506)
Slovenia	1810 (1522 to 2140)	1518 (1260 to 1807)	21 (14 to 39)	24 (16 to 48)	148 (99 to 209)	122 (82 to 176)	338 (233 to 817)	213 (150 to 375)
Eastern Europe	171769 (142378 to 207991)	182455 (151678 to 217882)	37925 (32876 to 43410)	12911 (12079 to 15773)	14528 (9905 to 20478)	15300 (10364 to 21560)	1369669 (1207303 to 1569010)	424363 (399811 to 501888)
Belarus	7324 (5935 to 8878)	6569 (5448 to 7813)	671 (523 to 924)	231 (162 to 296)	619 (419 to 888)	551 (362 to 784)	23995 (18610 to 32162)	7005 (4802 to 9335)
Estonia	619 (501 to 753)	292 (239 to 349)	81 (57 to 123)	11 (8 to 21)	52 (34 to 74)	25 (16 to 38)	2355 (1674 to 3671)	313 (218 to 591)
Latvia	1103 (926 to 1319)	650 (537 to 771)	357 (183 to 451)	86 (66 to 127)	92 (59 to 132)	55 (35 to 81)	12370 (6433 to 15457)	2744 (2129 to 3942)
Lithuania	2151 (1765 to 2585)	1789 (1475 to 2160)	197 (156 to 286)	42 (33 to 65)	181 (121 to 260)	151 (97 to 214)	6700 (5316 to 9554)	1295 (1019 to 1942)
Moldova	1356 (1091 to 1663)	726 (583 to 901)	163 (53 to 194)	47 (40 to 55)	116 (76 to 168)	62 (38 to 92)	5348 (1832 to 6399)	1391 (1188 to 1631)
Russian Federation	120861 (99329 to 145468)	129688 (107214 to 154725)	28021 (22567 to 33563)	10282 (9524 to 13082)	10222 (6903 to 14335)	10871 (7416 to 15323)	1004736 (814082 to 1205108)	339220 (315818 to 418774)
Ukraine	38356 (31315 to 46377)	42741 (35440 to 52427)	8435 (7262 to 11379)	2211 (1947 to 2493)	3247 (2182 to 4694)	3585 (2401 to 5165)	314165 (267273 to 431415)	72395 (61543 to 82455)
North Africa and Middle East	14532 (12348 to 16889)	10230 (8664 to 11993)	450 (374 to 583)	246 (187 to 312)	1320 (883 to 1874)	941 (627 to 1345)	12421 (10001 to 16873)	8097 (5277 to 10859)
Afghanistan	282 (233 to 333)	545 (437 to 668)	14 (4 to 47)	42 (10 to 71)	26 (17 to 37)	50 (33 to 72)	455 (123 to 1637)	1703 (330 to 3109)
Algeria	1444 (1202 to 1702)	610 (504 to 726)	41 (18 to 84)	16 (11 to 23)	128 (82 to 184)	56 (37 to 82)	993 (432 to 1960)	408 (268 to 567)
Bahrain	118 (97 to 142)	48 (39 to 57)	1 (1 to 2)	0 (0 to 1)	11 (7 to 16)	4 (3 to 6)	39 (24 to 57)	13 (10 to 16)
Egypt	1777 (1475 to 2114)	2128 (1739 to 2576)	76 (26 to 177)	56 (29 to 87)	164 (107 to 234)	196 (131 to 285)	2271 (796 to 5228)	1955 (878 to 3261)
Iran	1811 (1500 to 2159)	1141 (942 to 1356)	67 (50 to 88)	25 (20 to 31)	166 (109 to 239)	105 (69 to 151)	1674 (1234 to 2302)	605 (494 to 732)
Iraq	567 (472 to 670)	808 (658 to 979)	7 (5 to 10)	5 (3 to 7)	53 (34 to 74)	75 (49 to 107)	262 (182 to 362)	181 (127 to 243)
Jordan	149 (122 to 176)	61 (49 to 73)	2 (2 to 3)	0 (0 to 1)	14 (9 to 20)	6 (4 to 8)	85 (60 to 115)	19 (13 to 27)
Kuwait	83 (68 to 98)	38 (31 to 46)	2 (1 to 2)	0 (0 to 0)	8 (5 to 11)	4 (2 to 5)	49 (32 to 71)	11 (7 to 14)
Lebanon	247 (207 to 290)	142 (118 to 169)	7 (4 to 12)	2 (1 to 3)	22 (15 to 32)	13 (9 to 19)	188 (106 to 333)	40 (25 to 63)
Libya	201 (167 to 238)	100 (82 to 120)	5 (3 to 11)	2 (1 to 4)	18 (12 to 26)	9 (6 to 13)	164 (78 to 349)	81 (45 to 137)
Morocco	1092 (914 to 1303)	797 (658 to 961)	37 (23 to 58)	22 (15 to 32)	99 (65 to 142)	74 (48 to 107)	939 (607 to 1439)	647 (396 to 1006)
Palestine	172 (144 to 203)	157 (132 to 186)	3 (3 to 5)	2 (2 to 4)	15 (10 to 22)	14 (9 to 20)	109 (79 to 156)	51 (36 to 79)
Oman	427 (350 to 505)	78 (66 to 93)	6 (3 to 10)	1 (1 to 1)	38 (24 to 56)	7 (5 to 10)	216 (118 to 368)	29 (20 to 43)
Qatar	221 (182 to 265)	31 (25 to 37)	2 (1 to 2)	0 (0 to 0)	20 (13 to 29)	3 (2 to 4)	62 (37 to 107)	4 (3 to 5)
Saudi Arabia	2112 (1774 to 2486)	967 (813 to 1133)	56 (23 to 117)	16 (10 to 25)	187 (124 to 267)	86 (57 to 125)	1477 (593 to 3052)	474 (300 to 746)
Sudan	684 (572 to 814)	719 (589 to 858)	30 (14 to 60)	23 (14 to 38)	62 (41 to 90)	66 (43 to 96)	826 (431 to 1636)	817 (437 to 1357)
Syria	604 (504 to 717)	410 (344 to 490)	21 (13 to 31)	6 (4 to 9)	54 (35 to 79)	38 (25 to 54)	542 (347 to 798)	166 (98 to 251)
Tunisia	456 (379 to 545)	193 (160 to 228)	15 (7 to 26)	4 (3 to 6)	41 (27 to 59)	18 (12 to 26)	324 (155 to 557)	78 (50 to 121)
Turkey	1063 (894 to 1247)	680 (582 to 789)	25 (18 to 33)	6 (4 to 7)	98 (66 to 140)	63 (42 to 90)	708 (533 to 929)	185 (145 to 240)
United Arab Emirates	636 (506 to 792)	138 (109 to 171)	14 (5 to 43)	2 (1 to 4)	59 (38 to 86)	13 (8 to 18)	548 (205 to 1590)	66 (26 to 131)
Yemen	372 (314 to 436)	429 (348 to 520)	17 (9 to 29)	16 (9 to 24)	34 (22 to 49)	40 (26 to 57)	480 (280 to 834)	558 (262 to 898)

Central Asia	17172 (14206 to 20411)	8304 (6913 to 9879)	2244 (876 to 2849)	387 (325 to 473)	1474 (956 to 2073)	712 (475 to 1004)	80789 (32971 to 103836)	13437 (11122 to 16510)
Armenia	1398 (1162 to 1670)	1538 (1285 to 1818)	20 (13 to 38)	5 (4 to 14)	118 (79 to 170)	129 (87 to 182)	464 (323 to 1172)	97 (75 to 163)
Azerbaijan	2069 (1695 to 2507)	1138 (931 to 1379)	115 (66 to 203)	28 (16 to 41)	178 (113 to 259)	98 (60 to 147)	3868 (2069 to 6943)	879 (458 to 1308)
Georgia	156 (131 to 185)	106 (87 to 125)	18 (11 to 37)	6 (4 to 12)	14 (9 to 21)	10 (6 to 14)	551 (317 to 1158)	93 (64 to 195)
Kazakhstan	8865 (7234 to 10637)	2756 (2233 to 3367)	1670 (439 to 2239)	224 (166 to 306)	756 (487 to 1086)	236 (147 to 350)	59957 (16734 to 81542)	7808 (5797 to 10818)
Kyrgyzstan	2634 (2181 to 3157)	1552 (1248 to 1881)	253 (107 to 307)	66 (57 to 74)	228 (150 to 332)	133 (85 to 195)	9576 (4092 to 11631)	2402 (2069 to 2714)
Mongolia	173 (140 to 212)	115 (94 to 142)	14 (10 to 22)	5 (3 to 8)	15 (9 to 23)	10 (6 to 16)	555 (380 to 862)	127 (76 to 215)
Tajikistan	64 (52 to 77)	43 (34 to 52)	3 (2 to 3)	2 (1 to 4)	6 (4 to 9)	4 (3 to 6)	97 (73 to 137)	86 (47 to 181)
Turkmenistan	1488 (1231 to 1784)	921 (752 to 1123)	134 (97 to 173)	46 (33 to 56)	128 (81 to 184)	79 (51 to 116)	4965 (3546 to 6485)	1689 (1242 to 2055)
Uzbekistan	325 (263 to 387)	135 (110 to 160)	18 (12 to 35)	6 (5 to 9)	30 (19 to 43)	12 (8 to 18)	755 (505 to 1373)	256 (190 to 365)
South Asia	65657 (54064 to 78655)	46814 (38855 to 55490)	3844 (2054 to 6370)	1813 (1068 to 2946)	5941 (3984 to 8552)	4237 (2817 to 6109)	116406 (60861 to 194257)	51722 (27847 to 84956)
Bangladesh	7805 (6496 to 9280)	5530 (4607 to 6619)	245 (75 to 494)	130 (64 to 203)	696 (452 to 1014)	490 (316 to 710)	7430 (1940 to 16134)	3602 (1735 to 5924)
Bhutan	52 (44 to 63)	23 (20 to 28)	2 (1 to 4)	1 (0 to 1)	5 (3 to 7)	2 (1 to 3)	49 (17 to 104)	14 (8 to 24)
India	48576 (39677 to 58833)	35424 (29093 to 42378)	3102 (1792 to 5105)	1469 (828 to 2356)	4423 (2920 to 6442)	3224 (2130 to 4662)	93997 (53548 to 153764)	41293 (21643 to 66537)
Nepal	1309 (1079 to 1574)	712 (594 to 848)	53 (15 to 106)	15 (7 to 29)	115 (73 to 173)	66 (43 to 96)	1451 (420 to 2884)	414 (200 to 754)
Pakistan	7914 (6607 to 9429)	5125 (4299 to 6088)	443 (123 to 921)	198 (108 to 368)	702 (462 to 1016)	456 (294 to 659)	13479 (3437 to 28485)	6399 (3455 to 11859)
Southeast Asia	27917 (23220 to 33132)	15519 (12917 to 18541)	1655 (1225 to 2318)	457 (399 to 534)	2533 (1682 to 3607)	1414 (934 to 2015)	52146 (38974 to 72650)	12364 (10654 to 14509)
Cambodia	414 (337 to 496)	412 (337 to 504)	23 (14 to 38)	17 (9 to 28)	38 (25 to 55)	38 (24 to 55)	742 (441 to 1249)	499 (249 to 833)
Indonesia	9897 (7970 to 12086)	7577 (6207 to 9219)	466 (191 to 1003)	223 (182 to 274)	907 (594 to 1296)	693 (451 to 997)	14480 (5801 to 31316)	6420 (5004 to 8087)
Laos	178 (146 to 214)	200 (164 to 240)	14 (8 to 25)	9 (5 to 14)	16 (11 to 24)	18 (12 to 26)	463 (248 to 836)	292 (156 to 469)
Malaysia	1422 (1174 to 1696)	1108 (919 to 1312)	53 (36 to 83)	22 (17 to 30)	129 (84 to 187)	99 (64 to 144)	1672 (1103 to 2740)	608 (455 to 845)
Maldives	7 (5 to 8)	10 (8 to 12)	0 (0 to 0)	0 (0 to 0)	1 (0 to 1)	1 (1 to 1)	4 (3 to 6)	4 (3 to 5)
Mauritius	89 (74 to 106)	48 (40 to 58)	4 (3 to 8)	2 (1 to 2)	8 (5 to 12)	4 (3 to 6)	156 (105 to 301)	40 (32 to 55)
Myanmar	1213 (989 to 1470)	987 (804 to 1194)	85 (45 to 141)	24 (16 to 37)	112 (74 to 161)	91 (60 to 134)	2657 (1385 to 4374)	673 (439 to 995)
Philippines	4194 (3427 to 5074)	893 (743 to 1071)	409 (295 to 546)	43 (30 to 79)	384 (244 to 557)	83 (54 to 120)	13628 (10332 to 17994)	1103 (819 to 1605)
Sri Lanka	3867 (3143 to 4616)	1741 (1411 to 2109)	292 (161 to 418)	46 (30 to 67)	330 (208 to 486)	151 (93 to 226)	8355 (4834 to 11950)	1250 (806 to 1825)
Seychelles	16 (13 to 19)	3 (2 to 4)	2 (2 to 2)	0 (0 to 0)	1 (1 to 2)	0 (0 to 0)	57 (44 to 71)	5 (4 to 6)
Thailand	1117 (907 to 1363)	700 (578 to 837)	27 (21 to 37)	10 (8 to 13)	104 (67 to 153)	65 (42 to 94)	856 (660 to 1150)	256 (199 to 320)
Timor-Leste	40 (33 to 47)	21 (18 to 26)	2 (0 to 5)	1 (0 to 1)	4 (2 to 5)	2 (1 to 3)	65 (11 to 170)	22 (13 to 32)
Vietnam	5428 (4434 to 6601)	1798 (1504 to 2136)	275 (155 to 463)	58 (37 to 89)	495 (319 to 713)	167 (108 to 240)	8943 (4953 to 15253)	1178 (738 to 1723)
East Asia	71620 (57444 to 88203)	23031 (19080 to 27910)	2778 (2300 to 3601)	463 (355 to 770)	6579 (4310 to 9549)	2124 (1404 to 3074)	89388 (73932 to 113377)	10351 (8278 to 15888)
China	63835 (50662 to 79384)	19913 (16258 to 24410)	2635 (2149 to 3431)	426 (321 to 722)	5885 (3846 to 8609)	1844 (1211 to 2657)	84599 (69164 to 107589)	9384 (7388 to 14531)
North Korea	1143 (935 to 1375)	576 (474 to 702)	37 (21 to 54)	19 (11 to 30)	106 (69 to 152)	53 (35 to 77)	1287 (692 to 1940)	561 (286 to 864)
Taiwan (Province of China)	5489 (4750 to 6219)	2171 (1911 to 2461)	62 (44 to 78)	11 (8 to 14)	483 (311 to 703)	192 (123 to 283)	2062 (1433 to 2686)	240 (186 to 330)
Oceania	119 (99 to 143)	169 (139 to 203)	20 (12 to 38)	26 (11 to 41)	11 (7 to 16)	15 (10 to 22)	645 (388 to 1258)	1067 (395 to 1732)
American Samoa	2 (1 to 2)	2 (2 to 3)	0 (0 to 0)	0 (0 to 0)	0 (0 to 0)	0 (0 to 0)	3 (2 to 5)	2 (1 to 2)
Federated States of Micronesia	2 (1 to 2)	2 (2 to 3)	0 (0 to 0)	0 (0 to 0)	0 (0 to 0)	0 (0 to 0)	6 (3 to 9)	6 (3 to 11)
Fiji	11 (9 to 13)	10 (8 to 12)	2 (1 to 2)	1 (1 to 1)	1 (1 to 1)	1 (1 to 1)	45 (33 to 60)	27 (19 to 37)
Guam	8 (6 to 9)	7 (6 to 8)	0 (0 to 1)	0 (0 to 0)	1 (0 to 1)	1 (0 to 1)	14 (11 to 20)	5 (4 to 7)
Kiribati	2 (1 to 2)	2 (2 to 3)	0 (0 to 1)	0 (0 to 0)	0 (0 to 0)	0 (0 to 0)	14 (6 to 24)	7 (5 to 10)
Marshall Islands	1 (1 to 1)	1 (1 to 2)	0 (0 to 0)	0 (0 to 0)	0 (0 to 0)	0 (0 to 0)	4 (1 to 8)	7 (2 to 11)
Northern Mariana Islands	5 (4 to 6)	2 (2 to 2)	0 (0 to 0)	0 (0 to 0)	0 (0 to 1)	0 (0 to 0)	5 (3 to 6)	1 (0 to 1)
Papua New Guinea	67 (55 to 81)	117 (95 to 141)	14 (7 to 30)	21 (7 to 35)	6 (4 to 9)	11 (7 to 15)	467 (235 to 1000)	908 (292 to 1531)
Samoa	4 (3 to 5)	4 (3 to 5)	0 (0 to 1)	0 (0 to 0)	0 (0 to 1)	0 (0 to 1)	9 (3 to 18)	6 (4 to 10)
Solomon Islands	5 (4 to 6)	9 (7 to 10)	1 (0 to 1)	1 (0 to 1)	0 (0 to 1)	1 (1 to 1)	17 (7 to 44)	29 (10 to 48)
Tonga	1 (1 to 2)	1 (0 to 1)	0 (0 to 0)	0 (0 to 0)	0 (0 to 0)	0 (0 to 0)	2 (1 to 3)	0 (0 to 0)
Vanuatu	5 (4 to 6)	3 (2 to 4)	1 (0 to 2)	0 (0 to 1)	0 (0 to 1)	0 (0 to 0)	24 (4 to 59)	10 (4 to 20)
High-income Asia Pacific	39814 (33694 to	7728 (6632 to 8906)	290 (228 to 371)	61 (45 to 74)	3476 (2385 to 4873)	709 (472 to 999)	7260 (5616 to 9624)	1042 (763 to 1239)

	46122)							
Brunei	173 (145 to 207)	58 (49 to 68)	4 (3 to 5)	1 (1 to 2)	15 (10 to 21)	5 (3 to 7)	150 (117 to 196)	40 (30 to 50)
Japan	33619 (28466 to 39097)	4668 (4033 to 5319)	230 (181 to 287)	31 (21 to 36)	2929 (2001 to 4132)	432 (292 to 609)	5800 (4497 to 7341)	529 (355 to 631)
Singapore	1507 (1258 to 1796)	671 (564 to 788)	10 (6 to 26)	2 (1 to 3)	132 (85 to 190)	59 (36 to 87)	321 (198 to 783)	59 (43 to 76)
South Korea	4515 (3703 to 5472)	2331 (1939 to 2762)	46 (32 to 63)	27 (20 to 36)	400 (244 to 585)	213 (140 to 310)	989 (702 to 1461)	414 (304 to 536)
High-income North America	191533 (162000 to 223127)	65632 (56539 to 75632)	5339 (4038 to 6409)	977 (809 to 1065)	16024 (11014 to 22263)	5540 (3790 to 7715)	130245 (99662 to 158754)	23000 (19607 to 25271)
Canada	24233 (19510 to 29463)	8450 (6913 to 10182)	323 (230 to 419)	46 (31 to 57)	2063 (1357 to 2999)	724 (462 to 1054)	7868 (5703 to 10186)	1081 (739 to 1349)
Greenland	17 (14 to 21)	2 (2 to 2)	1 (1 to 1)	0 (0 to 0)	1 (1 to 2)	0 (0 to 0)	22 (16 to 29)	1 (1 to 1)
United States	167280 (142006 to 196398)	57179 (49212 to 65938)	5015 (3797 to 5973)	931 (778 to 1013)	13960 (9612 to 19442)	4816 (3291 to 6703)	122353 (93322 to 148752)	21918 (18855 to 24134)
Western Europe	265641 (224137 to 313211)	76670 (64908 to 89510)	5171 (3429 to 6275)	918 (762 to 1297)	22465 (15015 to 31963)	6543 (4366 to 9250)	119288 (89412 to 135633)	17947 (15447 to 21835)
Andorra	44 (36 to 53)	8 (6 to 9)	1 (0 to 1)	0 (0 to 0)	4 (2 to 5)	1 (0 to 1)	20 (9 to 31)	1 (1 to 2)
Austria	5190 (4493 to 5977)	678 (569 to 788)	117 (37 to 162)	26 (19 to 52)	436 (295 to 630)	59 (37 to 87)	2469 (1012 to 3070)	352 (277 to 506)
Belgium	6624 (5369 to 7942)	1944 (1606 to 2327)	107 (62 to 148)	17 (13 to 26)	560 (359 to 809)	164 (105 to 240)	2372 (1676 to 2953)	306 (235 to 388)
Cyprus	130 (107 to 158)	55 (46 to 64)	3 (2 to 6)	1 (1 to 2)	11 (7 to 17)	5 (3 to 7)	77 (51 to 132)	17 (12 to 27)
Denmark	2654 (2126 to 3249)	329 (272 to 391)	38 (24 to 46)	6 (5 to 8)	224 (144 to 331)	30 (20 to 43)	967 (628 to 1196)	134 (103 to 171)
Finland	7396 (5967 to 9182)	1653 (1340 to 2011)	224 (111 to 276)	27 (21 to 35)	623 (410 to 893)	141 (87 to 203)	6049 (3181 to 7335)	722 (584 to 936)
France	32638 (27128 to 39438)	9166 (7650 to 10836)	425 (288 to 551)	41 (32 to 55)	2777 (1807 to 4032)	789 (498 to 1166)	10321 (7102 to 13138)	835 (572 to 1047)
Germany	116688 (96105 to 142138)	37046 (30455 to 44083)	2985 (1745 to 3781)	642 (500 to 930)	9805 (6339 to 14190)	3108 (2024 to 4454)	65153 (43090 to 78504)	12062 (9737 to 15223)
Greece	946 (780 to 1137)	70 (59 to 82)	20 (15 to 32)	2 (1 to 2)	83 (53 to 130)	7 (4 to 9)	462 (338 to 789)	30 (25 to 38)
Iceland	70 (58 to 84)	20 (16 to 23)	1 (1 to 1)	0 (0 to 0)	6 (4 to 8)	2 (1 to 3)	16 (12 to 24)	3 (2 to 3)
Ireland	2252 (1856 to 2685)	577 (488 to 678)	45 (33 to 61)	8 (6 to 12)	192 (127 to 280)	50 (32 to 75)	1127 (758 to 1693)	141 (100 to 200)
Israel	540 (444 to 654)	200 (167 to 237)	6 (3 to 8)	1 (1 to 1)	48 (30 to 71)	19 (12 to 27)	149 (87 to 208)	21 (14 to 26)
Italy	31251 (25366 to 36247)	10612 (8960 to 12654)	105 (73 to 224)	17 (13 to 26)	2652 (1730 to 3742)	901 (573 to 1316)	2546 (1571 to 5346)	278 (194 to 357)
Luxembourg	302 (248 to 366)	109 (91 to 130)	4 (3 to 8)	1 (1 to 2)	25 (16 to 37)	9 (6 to 13)	87 (55 to 192)	16 (12 to 21)
Malta	104 (85 to 128)	24 (20 to 28)	2 (1 to 2)	0 (0 to 0)	9 (6 to 13)	2 (1 to 3)	36 (26 to 47)	5 (4 to 7)
Netherlands	11037 (9019 to 13420)	2784 (2308 to 3320)	136 (101 to 197)	18 (15 to 23)	934 (615 to 1352)	238 (149 to 349)	3184 (2029 to 5062)	374 (262 to 468)
Norway	1089 (924 to 1269)	299 (259 to 339)	18 (15 to 25)	5 (4 to 6)	93 (63 to 132)	27 (18 to 38)	459 (378 to 663)	88 (74 to 99)
Portugal	3587 (2928 to 4300)	563 (475 to 664)	71 (46 to 99)	5 (4 to 7)	305 (188 to 450)	51 (33 to 75)	1812 (1284 to 2325)	75 (58 to 107)
Spain	13175 (10605 to 16002)	2583 (2152 to 3085)	224 (168 to 354)	12 (9 to 17)	1128 (728 to 1641)	233 (147 to 349)	5369 (4047 to 8198)	200 (161 to 256)
Sweden	6672 (5290 to 8184)	1138 (926 to 1402)	107 (63 to 133)	8 (6 to 10)	568 (377 to 824)	100 (64 to 154)	2652 (1546 to 3269)	199 (160 to 254)
Switzerland	5965 (5121 to 7002)	1108 (941 to 1279)	71 (40 to 90)	5 (4 to 6)	501 (337 to 713)	95 (61 to 137)	1489 (987 to 1865)	92 (71 to 119)
United Kingdom	17012 (14377 to 20059)	5626 (4848 to 6481)	456 (378 to 546)	77 (69 to 107)	1456 (996 to 2042)	507 (343 to 715)	12349 (10282 to 14573)	1976 (1773 to 2596)
Australasia	20887 (17339 to 24991)	5550 (4653 to 6536)	418 (319 to 541)	41 (26 to 51)	1759 (1152 to 2548)	474 (305 to 693)	10719 (8033 to 13912)	1011 (644 to 1250)
Australia	17652 (14573 to 21172)	4820 (4027 to 5713)	342 (252 to 449)	32 (20 to 41)	1488 (971 to 2174)	412 (263 to 610)	8730 (6182 to 11356)	779 (467 to 1010)
New Zealand	3235 (2661 to 3920)	730 (608 to 871)	75 (60 to 101)	10 (6 to 12)	271 (182 to 384)	62 (40 to 93)	1988 (1648 to 2589)	232 (177 to 271)

Data in parentheses are 95% uncertainty intervals. YLDs= years lived with disability; YLLs= years of life lost; SDI= Socio-demographic Index.

Supplementary Table 4. Numbers of other cardiomyopathy prevalent cases, deaths, YLDs, and YLLs in 2017, by sex, SDI quintile, and location.

	Prevalence		Deaths		YLDs		YLLs	
	Males	Females	Males	Females	Males	Females	Males	Females
Global	1925364 (1666236 to 2212439)	2287296 (1971579 to 2636031)	122410 (107544 to 133487)	110749 (103499 to 117302)	162537 (110484 to 227578)	190788 (130356 to 265376)	3290313 (2895143 to 3613749)	2224494 (1979275 to 2424578)
SDI Quintile								
Low SDI	176427 (146219 to 208166)	189080 (159748 to 221636)	11980 (8839 to 16067)	9595 (7025 to 12889)	15106 (10123 to 21204)	16081 (10864 to 22890)	436960 (334873 to 568242)	368970 (262507 to 485807)
Low-middle SDI	239200 (203054 to 280182)	279506 (237447 to 326015)	19757 (16616 to 23057)	14262 (12126 to 16669)	20487 (13818 to 28971)	23761 (16064 to 33292)	633924 (550521 to 731059)	446735 (373503 to 521909)
Middle SDI	305144 (257919 to 357326)	382800 (326172 to 445270)	23127 (19158 to 25971)	17513 (16257 to 19516)	26362 (17773 to 37126)	32609 (22052 to 45559)	656874 (544596 to 743888)	404191 (374450 to 447406)
High-middle SDI	270403 (229730 to 314947)	363119 (311649 to 422535)	31413 (24372 to 34114)	28747 (24675 to 30278)	23101 (15498 to 32183)	30455 (20413 to 42825)	902754 (672300 to 995679)	518750 (434154 to 546526)
High SDI	930840 (803473 to 1076723)	1069137 (914020 to 1245260)	35938 (32069 to 39206)	40469 (38204 to 44087)	77188 (52503 to 106778)	87565 (59449 to 120939)	654060 (615938 to 799746)	481797 (459922 to 515147)
Central Sub-Saharan Africa	24105 (19561 to 29091)	25840 (21021 to 30954)	1482 (921 to 2304)	1366 (798 to 2079)	2028 (1367 to 2888)	2165 (1448 to 3085)	60609 (40249 to 87647)	59841 (34617 to 90242)
Angola	5523 (4447 to 6726)	5912 (4772 to 7170)	367 (218 to 535)	282 (187 to 381)	466 (308 to 660)	499 (331 to 713)	14956 (9390 to 21146)	12540 (8130 to 17125)
Central African Republic	951 (760 to 1170)	908 (724 to 1104)	71 (40 to 111)	75 (34 to 137)	80 (52 to 115)	76 (49 to 110)	3033 (1649 to 4685)	3609 (1649 to 6417)
Congo	1292 (1028 to 1591)	1225 (984 to 1496)	84 (54 to 127)	76 (43 to 111)	109 (71 to 158)	103 (68 to 150)	2987 (1976 to 4376)	2683 (1458 to 3963)
Democratic Republic of the Congo	15501 (12459 to 18836)	16902 (13628 to 20424)	905 (522 to 1559)	899 (472 to 1481)	1303 (859 to 1892)	1412 (932 to 2053)	37709 (23301 to 59612)	39959 (21071 to 64498)
Equatorial Guinea	266 (216 to 322)	282 (229 to 340)	15 (9 to 23)	12 (7 to 18)	23 (15 to 33)	24 (16 to 34)	594 (366 to 899)	417 (261 to 620)
Gabon	572 (456 to 699)	611 (495 to 745)	39 (28 to 55)	22 (16 to 32)	48 (32 to 69)	51 (33 to 72)	1330 (951 to 1840)	633 (430 to 865)
Eastern Sub-Saharan Africa	70153 (57089 to 84498)	71220 (58728 to 85244)	4433 (2736 to 5401)	2438 (1982 to 3231)	5979 (3950 to 8543)	6068 (4028 to 8627)	197992 (132877 to 243469)	114913 (84674 to 150868)
Burundi	1431 (1122 to 1768)	1146 (922 to 1398)	116 (66 to 162)	66 (46 to 96)	123 (79 to 179)	99 (64 to 144)	5547 (3416 to 7477)	3623 (2340 to 5282)
Comoros	167 (131 to 208)	191 (151 to 238)	9 (5 to 14)	6 (4 to 9)	14 (9 to 21)	16 (10 to 23)	341 (203 to 514)	203 (137 to 306)
Djibouti	255 (201 to 319)	212 (168 to 260)	17 (10 to 27)	7 (4 to 11)	22 (14 to 33)	18 (12 to 26)	669 (381 to 1038)	266 (161 to 429)
Eritrea	657 (523 to 808)	927 (732 to 1144)	84 (37 to 127)	53 (31 to 82)	56 (36 to 81)	79 (52 to 116)	4005 (1896 to 5967)	2239 (1272 to 3356)
Ethiopia	30679 (24985 to 36514)	28564 (23794 to 33875)	843 (516 to 1136)	481 (369 to 624)	2604 (1707 to 3704)	2421 (1598 to 3416)	37247 (25150 to 49534)	23956 (17160 to 30508)
Kenya	5848 (4657 to 7238)	6750 (5449 to 8194)	580 (299 to 708)	258 (214 to 333)	503 (334 to 727)	580 (383 to 827)	22513 (12457 to 26834)	9889 (7985 to 12767)
Madagascar	3856 (3065 to 4812)	3912 (3096 to 4790)	531 (311 to 756)	319 (199 to 455)	331 (212 to 489)	337 (214 to 501)	24887 (15526 to 35062)	14589 (8751 to 20496)
Malawi	2491 (1953 to 3096)	3215 (2541 to 3975)	172 (118 to 222)	90 (64 to 126)	212 (137 to 308)	272 (176 to 393)	7708 (5330 to 10475)	4010 (2663 to 5656)
Mozambique	2314 (1818 to 2928)	2734 (2180 to 3393)	369 (250 to 506)	171 (126 to 232)	198 (124 to 292)	235 (150 to 344)	16453 (11280 to 21689)	7925 (5393 to 11441)
Rwanda	1799 (1425 to 2212)	2111 (1678 to 2613)	128 (64 to 180)	82 (60 to 118)	154 (101 to 224)	180 (116 to 266)	5215 (2752 to 7340)	3299 (2492 to 4691)
Somalia	2413 (1908 to 2998)	2399 (1903 to 2986)	187 (110 to 291)	132 (81 to 200)	207 (137 to 300)	206 (136 to 305)	9344 (5512 to 14461)	6623 (3799 to 10159)
South Sudan	1572 (1238 to 1952)	1331 (1068 to 1628)	118 (78 to 176)	91 (58 to 124)	133 (87 to 196)	113 (73 to 165)	6050 (3872 to 9059)	5291 (3145 to 7362)
Tanzania	9257 (7266 to 11542)	9918 (7934 to 12331)	696 (380 to 1011)	412 (325 to 574)	789 (509 to 1154)	845 (550 to 1222)	31596 (18709 to 45732)	19977 (14489 to 28638)
Uganda	5033 (3990 to 6181)	5257 (4229 to 6436)	389 (210 to 540)	179 (136 to 289)	429 (277 to 621)	449 (293 to 655)	17995 (10625 to 24592)	8785 (6483 to 13853)
Zambia	2338 (1867 to 2857)	2508 (1995 to 3110)	190 (111 to 265)	90 (62 to 141)	200 (128 to 287)	215 (142 to 314)	8299 (5046 to 11527)	4167 (2744 to 6465)
Southern Sub-Saharan Africa	35813 (29233 to 42936)	44235 (35514 to 54061)	1991 (1855 to 2148)	1694 (1548 to 1841)	2972 (1992 to 4176)	3636 (2379 to 5153)	56302 (51948 to 61263)	40280 (36740 to 44225)
Botswana	943 (758 to 1151)	1149 (927 to 1403)	42 (30 to 55)	37 (29 to 47)	78 (51 to 112)	95 (61 to 138)	1083 (757 to 1433)	834 (668 to 1035)
Lesotho	610 (488 to 760)	863 (675 to 1077)	49 (36 to 62)	54 (33 to 77)	50 (33 to 73)	70 (45 to 102)	1531 (1112 to 2018)	1308 (825 to 1823)
Namibia	864 (693 to 1052)	1148 (907 to 1390)	54 (35 to 68)	36 (25 to 52)	71 (47 to 103)	94 (61 to 133)	1474 (921 to 2001)	790 (572 to 1078)
South Africa	30460 (24855 to 36709)	37502 (29911 to 46131)	1499 (1355 to 1636)	1268 (1143 to 1401)	2525 (1697 to 3561)	3079 (2011 to 4373)	40903 (36406 to 45099)	27817 (25109 to 30823)
Swaziland	304 (243 to 373)	407 (322 to 504)	25 (18 to 35)	18 (12 to 25)	25 (16 to 36)	33 (22 to 49)	857 (637 to 1181)	515 (355 to 706)
Zimbabwe	2630 (2084 to 3277)	3166 (2521 to 3880)	323 (230 to 436)	280 (205 to 358)	221 (144 to 326)	264 (172 to 389)	10455 (7366 to 14159)	9015 (6446 to 11490)
Western Sub-Saharan Africa	91371 (73969 to 110975)	91995 (74971 to 110100)	3951 (3106 to 4897)	2362 (1822 to 2844)	7687 (5122 to 11111)	7789 (5191 to 11196)	126413 (104553 to 151431)	98206 (70917 to 121318)
Benin	2173 (1719 to 2699)	2183 (1764 to 2639)	89 (50 to 149)	66 (41 to 93)	183 (123 to 269)	185 (120 to 267)	2939 (1738 to 5030)	2664 (1591 to 3820)
Burkina Faso	3753 (2957 to 4632)	3788 (3041 to 4704)	187 (127 to 270)	149 (88 to 207)	317 (207 to 464)	321 (211 to 477)	6177 (4255 to 9401)	6126 (3332 to 8850)
Cameroon	5777 (4598 to 7098)	5117 (4178 to 6306)	309 (218 to 430)	160 (108 to 217)	489 (324 to 707)	433 (281 to 626)	10165 (7212 to 14322)	6384 (4101 to 8725)
Cape Verde	226 (179 to 280)	258 (206 to 323)	4 (4 to 5)	1 (1 to 2)	19 (12 to 27)	22 (14 to 31)	115 (96 to 139)	36 (31 to 43)
Chad	2782 (2203 to 3435)	2149 (1742 to 2600)	101 (59 to 157)	85 (47 to 135)	233 (149 to 338)	182 (120 to 263)	3411 (2185 to 5279)	4374 (2104 to 6974)
Cote d'Ivoire	5343 (4238 to 6554)	4455 (3557 to 5398)	262 (163 to 367)	128 (84 to 176)	452 (298 to 648)	377 (244 to 541)	9141 (5987 to 12494)	5656 (3628 to 7815)

The Gambia	494 (398 to 612)	483 (390 to 592)	22 (14 to 32)	11 (8 to 15)	42 (27 to 61)	40 (26 to 58)	687 (431 to 1022)	378 (263 to 505)
Ghana	6527 (5130 to 8171)	7657 (6111 to 9496)	581 (421 to 712)	473 (379 to 578)	552 (362 to 795)	650 (421 to 951)	17609 (13228 to 21772)	16256 (13160 to 19796)
Guinea	1875 (1449 to 2375)	1527 (1222 to 1897)	111 (67 to 171)	83 (48 to 123)	158 (102 to 234)	130 (85 to 192)	3472 (2233 to 5088)	3402 (1906 to 5089)
Guinea-Bissau	320 (253 to 395)	321 (259 to 393)	17 (11 to 28)	12 (8 to 17)	27 (18 to 40)	27 (18 to 40)	618 (406 to 1032)	523 (326 to 710)
Liberia	1019 (810 to 1254)	837 (680 to 1021)	36 (20 to 56)	22 (13 to 30)	85 (56 to 121)	70 (46 to 102)	1145 (652 to 1809)	908 (517 to 1248)
Mali	4088 (3218 to 5122)	3240 (2602 to 3969)	166 (81 to 299)	143 (71 to 204)	343 (220 to 502)	274 (179 to 397)	5663 (3034 to 9743)	7494 (3388 to 11033)
Mauritania	1102 (872 to 1385)	943 (760 to 1166)	41 (22 to 65)	22 (14 to 31)	92 (61 to 135)	79 (52 to 116)	1114 (618 to 1737)	749 (471 to 1012)
Niger	3549 (2818 to 4443)	3198 (2577 to 3853)	104 (47 to 193)	103 (46 to 164)	301 (200 to 442)	272 (180 to 398)	3652 (1693 to 6732)	5004 (2080 to 8037)
Nigeria	44458 (35902 to 54512)	48290 (39192 to 58622)	1670 (1113 to 2574)	751 (513 to 1043)	3732 (2447 to 5517)	4093 (2721 to 5909)	52609 (37021 to 77808)	32383 (21397 to 45140)
Sao Tome and Principe	51 (40 to 62)	50 (40 to 61)	2 (2 to 3)	1 (1 to 1)	4 (3 to 6)	4 (3 to 6)	80 (57 to 109)	36 (28 to 46)
Senegal	4610 (3680 to 5721)	4278 (3454 to 5216)	115 (60 to 185)	67 (43 to 87)	385 (255 to 560)	358 (233 to 513)	3380 (1857 to 5362)	2330 (1550 to 3019)
Sierra Leone	1714 (1349 to 2115)	1455 (1173 to 1788)	73 (51 to 100)	47 (31 to 63)	144 (92 to 211)	122 (80 to 179)	2423 (1768 to 3255)	2115 (1298 to 2966)
Togo	1508 (1189 to 1859)	1765 (1408 to 2196)	61 (41 to 89)	37 (23 to 53)	128 (84 to 185)	149 (98 to 218)	2012 (1342 to 2865)	1386 (859 to 1949)
Andean Latin America	7427 (6164 to 8850)	7470 (6304 to 8804)	350 (309 to 405)	246 (219 to 271)	637 (414 to 913)	643 (426 to 922)	12106 (10528 to 14136)	7908 (6968 to 8926)
Bolivia	633 (446 to 862)	860 (605 to 1136)	89 (65 to 118)	75 (56 to 93)	53 (32 to 85)	74 (43 to 112)	3339 (2398 to 4483)	2731 (2025 to 3503)
Ecuador	1927 (1589 to 2338)	1911 (1588 to 2287)	128 (108 to 150)	89 (76 to 102)	165 (107 to 241)	165 (107 to 243)	4118 (3438 to 4922)	2689 (2223 to 3196)
Peru	4866 (4046 to 5789)	4699 (3979 to 5527)	134 (107 to 173)	82 (66 to 101)	418 (269 to 596)	404 (264 to 578)	4649 (3629 to 5980)	2488 (1942 to 3134)
Tropical Latin America	91818 (74532 to 111653)	147247 (122248 to 175258)	9541 (8872 to 11742)	7314 (6896 to 7702)	7690 (5006 to 11073)	12273 (8002 to 17309)	250874 (230961 to 294711)	152335 (144197 to 159359)
Brazil	90581 (73444 to 110164)	145855 (121118 to 173710)	9486 (8815 to 11691)	7279 (6859 to 7666)	7584 (4923 to 10924)	12155 (7932 to 17153)	249473 (229499 to 293271)	151534 (143427 to 158586)
Paraguay	1236 (1016 to 1493)	1393 (1162 to 1667)	54 (42 to 68)	35 (27 to 44)	105 (68 to 152)	118 (76 to 171)	1401 (1100 to 1759)	801 (626 to 1027)
Central Latin America	36741 (30906 to 43135)	49578 (42232 to 57419)	1900 (1596 to 2071)	1516 (1406 to 1631)	3180 (2128 to 4467)	4270 (2865 to 6041)	61568 (56173 to 67038)	42114 (39491 to 45069)
Colombia	11983 (10013 to 14137)	14263 (11971 to 16920)	505 (418 to 599)	406 (348 to 470)	1030 (662 to 1478)	1219 (792 to 1744)	14195 (11789 to 19300)	9652 (8246 to 11241)
Costa Rica	1815 (1529 to 2156)	2371 (1999 to 2778)	104 (74 to 122)	72 (62 to 85)	155 (102 to 222)	202 (134 to 288)	2737 (2132 to 3214)	1562 (1350 to 1833)
El Salvador	402 (334 to 475)	446 (375 to 527)	22 (16 to 34)	15 (11 to 19)	35 (22 to 50)	38 (25 to 55)	709 (508 to 1081)	326 (249 to 440)
Guatemala	997 (830 to 1192)	1081 (914 to 1271)	62 (42 to 75)	47 (39 to 56)	87 (55 to 126)	95 (62 to 137)	2490 (1979 to 3090)	1763 (1433 to 2155)
Honduras	1194 (984 to 1414)	1284 (1075 to 1527)	91 (67 to 122)	84 (58 to 113)	103 (68 to 149)	110 (70 to 158)	3023 (2225 to 4119)	2167 (1502 to 2951)
Mexico	13896 (11448 to 16557)	19547 (16433 to 22982)	676 (578 to 757)	527 (497 to 565)	1217 (812 to 1733)	1704 (1139 to 2375)	26640 (21391 to 29184)	18887 (17489 to 20342)
Nicaragua	930 (784 to 1093)	1006 (853 to 1169)	36 (29 to 43)	30 (25 to 36)	81 (52 to 118)	87 (56 to 123)	1149 (958 to 1372)	833 (692 to 992)
Panama	1768 (1469 to 2093)	1527 (1285 to 1793)	103 (58 to 120)	56 (49 to 64)	150 (98 to 216)	130 (85 to 187)	2986 (1970 to 3478)	1338 (1165 to 1513)
Venezuela	3754 (2955 to 4617)	8053 (6694 to 9618)	301 (196 to 391)	278 (219 to 346)	321 (205 to 464)	686 (452 to 987)	7639 (5948 to 9817)	5585 (4469 to 6717)
Southern Latin America	29338 (22758 to 36240)	37958 (30662 to 46343)	2661 (2333 to 3350)	2036 (1782 to 2312)	2435 (1557 to 3505)	3147 (2020 to 4540)	59068 (51697 to 76683)	31709 (27962 to 35995)
Argentina	18677 (14250 to 23685)	26376 (20912 to 32448)	2084 (1768 to 2568)	1675 (1417 to 1945)	1546 (1015 to 2288)	2181 (1402 to 3184)	45578 (38706 to 59310)	25312 (21821 to 29368)
Chile	8451 (6646 to 10570)	8586 (6980 to 10415)	445 (371 to 618)	271 (227 to 318)	706 (452 to 1023)	720 (462 to 1048)	10879 (9172 to 13949)	5095 (4256 to 6013)
Uruguay	2208 (1751 to 2732)	2995 (2396 to 3696)	132 (107 to 198)	90 (74 to 108)	183 (117 to 262)	246 (162 to 349)	2608 (2089 to 3973)	1302 (1092 to 1546)
Caribbean	9273 (7956 to 10769)	15826 (13553 to 18557)	734 (649 to 826)	849 (687 to 1017)	792 (531 to 1117)	1334 (876 to 1890)	21406 (18860 to 24165)	23477 (18503 to 29953)
Antigua and Barbuda	25 (21 to 30)	27 (22 to 32)	2 (2 to 3)	2 (1 to 2)	2 (1 to 3)	2 (1 to 3)	57 (46 to 66)	39 (33 to 45)
The Bahamas	91 (77 to 107)	103 (86 to 123)	15 (13 to 18)	10 (8 to 11)	8 (5 to 11)	9 (6 to 13)	493 (414 to 587)	245 (207 to 285)
Barbados	169 (140 to 202)	193 (160 to 231)	12 (10 to 15)	9 (8 to 11)	14 (9 to 20)	16 (11 to 23)	289 (238 to 343)	172 (144 to 199)
Belize	86 (73 to 100)	105 (89 to 122)	8 (7 to 10)	6 (5 to 7)	7 (5 to 11)	9 (6 to 13)	280 (243 to 317)	178 (151 to 207)
Bermuda	44 (37 to 53)	74 (61 to 89)	3 (2 to 4)	2 (2 to 2)	4 (2 to 5)	6 (4 to 9)	66 (51 to 77)	26 (21 to 30)
Cuba	2578 (2169 to 3036)	6880 (5800 to 8204)	152 (120 to 200)	270 (210 to 323)	220 (144 to 319)	576 (378 to 820)	3038 (2370 to 4187)	4980 (4068 to 5919)
Dominica	57 (47 to 68)	46 (38 to 56)	10 (8 to 11)	6 (5 to 7)	5 (3 to 7)	4 (3 to 5)	254 (216 to 286)	108 (93 to 123)
Dominican Republic	1257 (1065 to 1473)	1804 (1527 to 2115)	94 (75 to 116)	92 (74 to 112)	109 (71 to 157)	155 (100 to 221)	3108 (2453 to 3794)	2552 (2082 to 3091)
Grenada	53 (44 to 64)	69 (57 to 83)	4 (4 to 5)	6 (5 to 7)	4 (3 to 6)	6 (4 to 8)	107 (93 to 131)	109 (92 to 126)
Guyana	125 (105 to 149)	147 (122 to 173)	21 (16 to 25)	18 (15 to 22)	11 (7 to 15)	12 (8 to 18)	663 (516 to 816)	537 (439 to 655)
Haiti	1232 (1042 to 1441)	1351 (1146 to 1578)	190 (131 to 267)	251 (156 to 396)	107 (69 to 151)	117 (77 to 166)	7109 (5196 to 9402)	10685 (6606 to 16822)
Jamaica	806 (692 to 947)	896 (757 to 1053)	57 (40 to 76)	42 (33 to 53)	69 (46 to 97)	76 (50 to 107)	1683 (1170 to 2307)	1003 (779 to 1271)
Puerto Rico	1697 (1396 to 2061)	2741 (2248 to 3324)	66 (55 to 93)	59 (48 to 70)	142 (92 to 203)	228 (145 to 326)	1364 (1122 to 2158)	910 (767 to 1055)
Saint Lucia	74 (63 to 86)	79 (67 to 94)	8 (7 to 10)	6 (5 to 7)	6 (4 to 9)	7 (4 to 10)	221 (183 to 258)	116 (99 to 133)
Saint Vincent and the Grenadines	16 (14 to 20)	23 (19 to 27)	2 (2 to 2)	2 (2 to 2)	1 (1 to 2)	2 (1 to 3)	54 (43 to 64)	44 (37 to 50)
Suriname	142 (119 to 168)	180 (150 to 215)	15 (13 to 18)	12 (11 to 15)	12 (8 to 18)	15 (10 to 21)	470 (391 to 559)	325 (278 to 381)

Trinidad and Tobago	430 (362 to 511)	460 (385 to 548)	40 (31 to 51)	21 (16 to 28)	36 (24 to 52)	39 (25 to 55)	1197 (887 to 1546)	526 (390 to 694)
Virgin Islands, U.S.	57 (47 to 70)	80 (66 to 97)	8 (6 to 10)	4 (4 to 5)	5 (3 to 7)	7 (4 to 10)	183 (138 to 234)	80 (66 to 96)
Central Europe	110428 (92309 to 131280)	195573 (160795 to 237364)	9738 (8347 to 10643)	12999 (11902 to 14049)	8885 (6013 to 12513)	15666 (10397 to 22226)	166709 (143932 to 183421)	143032 (132780 to 153484)
Albania	1624 (1339 to 1957)	2049 (1675 to 2451)	105 (75 to 149)	56 (39 to 80)	134 (88 to 188)	169 (113 to 241)	2071 (1564 to 2901)	947 (695 to 1272)
Bosnia and Herzegovina	2722 (2234 to 3289)	4074 (3289 to 4986)	204 (139 to 284)	239 (191 to 294)	221 (148 to 316)	328 (213 to 475)	3805 (2540 to 5464)	3209 (2613 to 3893)
Bulgaria	1751 (1440 to 2128)	1918 (1544 to 2395)	212 (173 to 316)	156 (116 to 193)	144 (92 to 212)	158 (101 to 229)	4548 (3798 to 5967)	2428 (2008 to 2867)
Croatia	1196 (1046 to 1375)	2699 (2213 to 3246)	88 (58 to 200)	190 (137 to 287)	98 (66 to 138)	218 (143 to 307)	1344 (928 to 3055)	1940 (1512 to 2827)
Czech Republic	7632 (6316 to 9306)	10362 (8450 to 12692)	343 (293 to 525)	285 (246 to 337)	621 (413 to 888)	845 (556 to 1238)	7223 (6225 to 9298)	4516 (3951 to 5201)
Hungary	4024 (3285 to 4922)	10265 (8186 to 12944)	284 (198 to 606)	414 (330 to 600)	324 (215 to 457)	816 (531 to 1209)	4763 (3361 to 9398)	4544 (3614 to 7210)
Macedonia	1833 (1502 to 2209)	1973 (1606 to 2403)	198 (104 to 308)	139 (100 to 176)	151 (97 to 213)	161 (105 to 232)	3934 (2004 to 6238)	2158 (1631 to 2637)
Montenegro	521 (434 to 626)	634 (521 to 767)	33 (23 to 47)	37 (26 to 49)	42 (29 to 60)	51 (34 to 72)	558 (379 to 799)	450 (322 to 608)
Poland	54857 (45263 to 66166)	112734 (91363 to 138441)	4028 (3230 to 4620)	6582 (5707 to 7488)	4383 (2969 to 6164)	8992 (5943 to 12829)	60954 (51179 to 73287)	64742 (56560 to 72710)
Romania	19196 (15446 to 23370)	29134 (22625 to 36848)	3009 (2351 to 3478)	3111 (2774 to 3483)	1539 (1002 to 2227)	2338 (1481 to 3471)	54963 (42216 to 64325)	38020 (33980 to 42404)
Serbia	6580 (5584 to 7767)	8855 (7236 to 10841)	881 (563 to 1211)	1365 (952 to 1763)	540 (366 to 757)	717 (469 to 1018)	15635 (10107 to 20787)	15392 (11303 to 19251)
Slovakia	3503 (2926 to 4174)	3629 (3103 to 4286)	143 (116 to 188)	101 (83 to 126)	291 (190 to 417)	300 (200 to 427)	3960 (3269 to 4722)	1981 (1722 to 2289)
Slovenia	4991 (4090 to 6039)	7248 (5900 to 8727)	210 (176 to 241)	324 (254 to 410)	398 (267 to 559)	572 (383 to 806)	2950 (2564 to 3453)	2705 (2170 to 3433)
Eastern Europe	27334 (23367 to 31644)	59479 (50113 to 71160)	11431 (6155 to 13626)	12285 (8590 to 13361)	2345 (1578 to 3259)	4899 (3285 to 6815)	386096 (198759 to 463096)	213780 (131873 to 235256)
Belarus	1876 (1566 to 2220)	3140 (2642 to 3696)	202 (76 to 430)	125 (75 to 190)	161 (103 to 236)	263 (170 to 375)	7547 (2955 to 13659)	3172 (1319 to 5351)
Estonia	918 (762 to 1096)	1034 (862 to 1221)	77 (48 to 108)	39 (32 to 49)	77 (50 to 112)	87 (57 to 125)	2195 (1351 to 3056)	866 (700 to 1059)
Latvia	1399 (1176 to 1664)	2069 (1712 to 2499)	167 (109 to 287)	146 (110 to 177)	115 (74 to 164)	171 (113 to 244)	4738 (2947 to 8408)	2847 (1843 to 3532)
Lithuania	1225 (1018 to 1459)	1722 (1448 to 2064)	161 (88 to 202)	72 (50 to 84)	102 (67 to 145)	143 (92 to 202)	4720 (2562 to 6036)	1668 (1075 to 1978)
Moldova	1261 (1046 to 1513)	1164 (982 to 1370)	74 (52 to 144)	45 (39 to 52)	108 (70 to 155)	99 (65 to 145)	2508 (1776 to 4754)	1125 (932 to 1310)
Russian Federation	15132 (12856 to 17597)	36544 (30496 to 44490)	8714 (3669 to 10773)	9068 (5976 to 9880)	1307 (869 to 1798)	3005 (1991 to 4248)	296218 (120725 to 370191)	168897 (94432 to 186638)
Ukraine	5523 (4682 to 6504)	13806 (11353 to 16849)	2036 (1650 to 2892)	2790 (2242 to 3263)	475 (314 to 690)	1132 (734 to 1610)	68170 (53635 to 102552)	35205 (28979 to 40778)
North Africa and Middle East	95538 (82613 to 108970)	80330 (69365 to 91690)	5265 (4734 to 5971)	2857 (2544 to 3242)	8289 (5599 to 11501)	6959 (4733 to 9612)	195628 (173828 to 225150)	112692 (96935 to 130198)
Afghanistan	1333 (1112 to 1556)	1141 (941 to 1360)	109 (70 to 164)	118 (68 to 190)	119 (77 to 171)	104 (67 to 148)	4290 (2853 to 6968)	5433 (3025 to 8785)
Algeria	7856 (6678 to 9159)	4804 (4063 to 5609)	399 (261 to 549)	217 (180 to 274)	671 (437 to 963)	415 (271 to 598)	12252 (8079 to 16695)	7341 (5951 to 9093)
Bahrain	643 (535 to 763)	352 (298 to 415)	13 (11 to 18)	6 (5 to 7)	56 (35 to 80)	30 (20 to 43)	420 (344 to 633)	184 (155 to 219)
Egypt	10805 (9178 to 12654)	10967 (9234 to 12840)	796 (558 to 1344)	480 (324 to 677)	947 (596 to 1353)	952 (612 to 1339)	30450 (21120 to 48399)	19604 (12376 to 28320)
Iran	13211 (11167 to 15462)	14046 (11827 to 16515)	853 (790 to 979)	514 (473 to 566)	1141 (757 to 1583)	1204 (811 to 1669)	26022 (23878 to 29504)	14667 (13365 to 16436)
Iraq	8420 (7191 to 9749)	6570 (5562 to 7691)	488 (418 to 571)	304 (257 to 362)	721 (474 to 1011)	562 (370 to 792)	26254 (21203 to 32722)	16678 (13400 to 21131)
Jordan	709 (594 to 827)	488 (407 to 573)	22 (18 to 28)	8 (6 to 10)	64 (42 to 91)	44 (29 to 63)	789 (644 to 969)	251 (197 to 318)
Kuwait	507 (430 to 592)	352 (296 to 414)	14 (11 to 20)	4 (4 to 5)	44 (28 to 64)	31 (20 to 45)	493 (391 to 719)	142 (114 to 174)
Lebanon	1437 (1221 to 1660)	1154 (980 to 1343)	65 (48 to 89)	29 (23 to 39)	124 (83 to 176)	99 (63 to 142)	2052 (1551 to 2741)	663 (503 to 915)
Libya	1253 (1067 to 1454)	908 (769 to 1060)	83 (56 to 115)	36 (24 to 54)	109 (71 to 154)	79 (50 to 113)	4255 (2770 to 6146)	1695 (1069 to 2603)
Morocco	5565 (4735 to 6512)	3499 (2987 to 4079)	351 (251 to 475)	188 (137 to 258)	480 (310 to 690)	306 (200 to 444)	10802 (7750 to 14652)	6124 (4430 to 8610)
Palestine	978 (831 to 1134)	1040 (891 to 1189)	38 (32 to 46)	28 (25 to 33)	84 (55 to 119)	89 (60 to 127)	1598 (1322 to 2015)	933 (791 to 1097)
Oman	1031 (853 to 1203)	352 (301 to 404)	35 (26 to 44)	12 (9 to 16)	90 (59 to 129)	30 (20 to 43)	1626 (1222 to 2052)	576 (416 to 777)
Qatar	1137 (941 to 1351)	250 (210 to 293)	14 (10 to 18)	2 (2 to 3)	100 (62 to 143)	22 (14 to 31)	585 (432 to 793)	99 (77 to 128)
Saudi Arabia	12774 (10869 to 14885)	7554 (6453 to 8703)	457 (338 to 632)	176 (141 to 227)	1102 (739 to 1562)	652 (434 to 932)	14643 (10516 to 20634)	5926 (4601 to 7857)
Sudan	3331 (2818 to 3854)	2048 (1704 to 2396)	315 (233 to 427)	180 (119 to 258)	294 (193 to 416)	185 (118 to 262)	14117 (9722 to 20524)	10551 (6289 to 16970)
Syria	3411 (2908 to 3976)	3045 (2605 to 3549)	213 (161 to 278)	77 (58 to 100)	293 (192 to 413)	264 (171 to 385)	6930 (5005 to 9275)	2718 (1945 to 3548)
Tunisia	2417 (2047 to 2847)	1715 (1446 to 1987)	137 (90 to 189)	58 (43 to 78)	206 (134 to 296)	147 (95 to 212)	3249 (2067 to 4437)	1243 (909 to 1705)
Turkey	13401 (11451 to 15416)	17429 (15294 to 19752)	557 (466 to 771)	255 (221 to 297)	1172 (750 to 1681)	1510 (976 to 2139)	21598 (18339 to 25788)	9121 (7815 to 10879)
United Arab Emirates	3179 (2533 to 3922)	791 (640 to 962)	124 (67 to 210)	17 (11 to 26)	281 (170 to 424)	70 (41 to 106)	5206 (2971 to 8526)	713 (475 to 1099)
Yemen	2049 (1747 to 2377)	1750 (1464 to 2047)	179 (128 to 239)	144 (95 to 223)	181 (119 to 255)	158 (102 to 220)	7815 (5509 to 11242)	7924 (4783 to 12997)
Central Asia	15963 (13630 to 18438)	22630 (19475 to 25986)	2546 (1734 to 2927)	2077 (1895 to 2298)	1374 (909 to 1914)	1910 (1284 to 2633)	87513 (61280 to 102181)	54621 (49505 to 60699)
Armenia	522 (446 to 613)	803 (680 to 943)	68 (46 to 79)	74 (65 to 85)	45 (29 to 65)	67 (44 to 97)	1460 (1171 to 1747)	979 (870 to 1094)
Azerbaijan	4174 (3493 to 4946)	5006 (4232 to 5904)	466 (365 to 808)	282 (229 to 352)	354 (235 to 520)	420 (277 to 593)	13622 (10377 to 26222)	6968 (5687 to 8661)
Georgia	749 (630 to 885)	1082 (899 to 1272)	102 (77 to 151)	77 (64 to 98)	64 (41 to 92)	91 (58 to 132)	2937 (2121 to 4497)	1351 (1098 to 1800)

Kazakhstan	5388 (4548 to 6254)	10971 (9307 to 12845)	1396 (434 to 1834)	1375 (1216 to 1573)	457 (303 to 654)	913 (599 to 1283)	48674 (17311 to 65030)	35452 (30429 to 41714)
Kyrgyzstan	151 (125 to 179)	245 (206 to 290)	12 (8 to 15)	9 (7 to 11)	14 (9 to 20)	22 (15 to 32)	530 (310 to 688)	344 (269 to 418)
Mongolia	761 (638 to 901)	779 (666 to 902)	83 (68 to 113)	42 (34 to 54)	65 (42 to 93)	66 (43 to 95)	2994 (2435 to 4365)	1249 (1004 to 1577)
Tajikistan	299 (246 to 351)	320 (264 to 377)	21 (18 to 25)	20 (16 to 24)	27 (18 to 38)	29 (19 to 42)	851 (716 to 1028)	751 (601 to 928)
Turkmenistan	1779 (1512 to 2070)	1831 (1540 to 2147)	213 (169 to 348)	115 (98 to 141)	153 (100 to 219)	155 (100 to 223)	8785 (7116 to 13742)	4153 (3489 to 5075)
Uzbekistan	2139 (1752 to 2512)	1594 (1336 to 1852)	185 (141 to 273)	82 (66 to 102)	197 (129 to 280)	147 (97 to 207)	7660 (5863 to 9876)	3375 (2634 to 4256)
South Asia	208943 (175841 to 244738)	271737 (229833 to 317677)	19834 (13370 to 26590)	15987 (11814 to 20939)	18100 (12222 to 25461)	23088 (15805 to 32642)	583630 (402228 to 785928)	454056 (330248 to 595568)
Bangladesh	26196 (21954 to 30429)	30580 (25861 to 35820)	1390 (858 to 2338)	1156 (793 to 1640)	2250 (1471 to 3276)	2589 (1680 to 3680)	39996 (22130 to 70309)	35683 (24490 to 49253)
Bhutan	170 (144 to 199)	153 (130 to 178)	10 (6 to 16)	7 (5 to 9)	15 (10 to 20)	13 (9 to 19)	286 (159 to 455)	177 (125 to 237)
India	149085 (124417 to 176109)	205324 (172512 to 242977)	15549 (9756 to 20372)	12774 (9247 to 17040)	12958 (8700 to 18461)	17434 (11926 to 24570)	455003 (294522 to 597779)	350512 (248468 to 469807)
Nepal	3998 (3354 to 4711)	3442 (2901 to 4014)	268 (148 to 417)	114 (80 to 159)	344 (224 to 497)	296 (191 to 420)	7238 (3994 to 11432)	3111 (2202 to 4281)
Pakistan	29494 (24935 to 34205)	32238 (27431 to 37290)	2616 (1549 to 4129)	1937 (1377 to 2683)	2532 (1646 to 3627)	2756 (1811 to 4005)	81106 (49386 to 129075)	64571 (45072 to 86896)
Southeast Asia	87439 (74284 to 102047)	101885 (86893 to 118216)	8061 (6696 to 9485)	5993 (5455 to 6707)	7550 (5032 to 10532)	8678 (5860 to 12102)	231657 (194778 to 271213)	133406 (121478 to 147282)
Cambodia	1215 (1015 to 1445)	1516 (1265 to 1820)	130 (101 to 161)	121 (86 to 169)	105 (67 to 153)	128 (81 to 188)	4321 (3377 to 5398)	2979 (2047 to 4241)
Indonesia	34269 (28417 to 40760)	37264 (31293 to 43974)	2885 (1978 to 4086)	1915 (1680 to 2383)	2962 (1954 to 4183)	3183 (2126 to 4457)	79187 (54726 to 111842)	47318 (41112 to 56251)
Laos	504 (426 to 599)	509 (426 to 593)	72 (52 to 98)	60 (45 to 76)	44 (29 to 64)	44 (29 to 63)	2522 (1843 to 3235)	2336 (1612 to 3197)
Malaysia	7525 (6330 to 8782)	7915 (6727 to 9180)	436 (358 to 528)	209 (178 to 243)	655 (423 to 945)	680 (462 to 957)	14830 (11977 to 17822)	6546 (5552 to 7592)
Maldives	25 (21 to 30)	38 (32 to 44)	1 (1 to 2)	2 (2 to 3)	2 (1 to 3)	3 (2 to 5)	29 (23 to 35)	35 (29 to 42)
Mauritius	212 (177 to 251)	216 (182 to 254)	30 (18 to 35)	14 (12 to 16)	18 (12 to 26)	18 (12 to 26)	897 (481 to 1084)	309 (267 to 352)
Myanmar	4431 (3716 to 5271)	3697 (3098 to 4382)	628 (473 to 860)	250 (187 to 327)	386 (248 to 557)	323 (212 to 475)	20826 (15188 to 27312)	5786 (4412 to 7424)
Philippines	6844 (5777 to 8064)	12279 (10365 to 14463)	1459 (1046 to 1763)	1968 (1630 to 2325)	596 (397 to 841)	1043 (690 to 1459)	41994 (33008 to 50559)	40232 (33042 to 47535)
Sri Lanka	11849 (9951 to 14040)	16515 (13799 to 19601)	849 (624 to 1156)	439 (316 to 576)	996 (651 to 1418)	1382 (912 to 1956)	22505 (16594 to 30143)	10028 (7202 to 13161)
Seychelles	17 (15 to 21)	17 (14 to 20)	4 (3 to 4)	3 (2 to 3)	1 (1 to 2)	1 (1 to 2)	91 (78 to 108)	41 (35 to 48)
Thailand	5649 (4735 to 6694)	6948 (5749 to 8291)	268 (224 to 347)	142 (119 to 170)	496 (316 to 723)	597 (384 to 862)	8020 (6481 to 9630)	3078 (2512 to 3688)
Timor-Leste	131 (111 to 155)	117 (97 to 140)	13 (6 to 23)	9 (7 to 11)	11 (7 to 16)	10 (6 to 14)	369 (182 to 664)	287 (198 to 386)
Vietnam	14651 (12250 to 17286)	14722 (12491 to 17270)	1277 (763 to 1741)	855 (644 to 1115)	1267 (831 to 1849)	1252 (817 to 1790)	35761 (21375 to 49702)	14255 (10929 to 18701)
East Asia	143358 (119435 to 171826)	147103 (122666 to 176353)	7800 (6847 to 9837)	5648 (5050 to 7136)	12801 (8588 to 18217)	12953 (8708 to 18229)	226828 (200555 to 280209)	132262 (119144 to 166081)
China	129203 (106409 to 155992)	134104 (111067 to 161700)	7272 (6351 to 9218)	5258 (4677 to 6698)	11571 (7672 to 16626)	11837 (7956 to 16696)	210709 (184954 to 262627)	122832 (109862 to 155689)
North Korea	2427 (2040 to 2850)	2768 (2311 to 3295)	164 (117 to 211)	163 (121 to 222)	217 (142 to 319)	242 (151 to 353)	6107 (4322 to 7998)	4933 (3540 to 6561)
Taiwan (Province of China)	9419 (8306 to 10626)	7861 (6896 to 8925)	239 (203 to 305)	136 (111 to 177)	807 (536 to 1156)	665 (451 to 929)	6357 (5444 to 7687)	2366 (1936 to 3177)
Oceania	802 (680 to 924)	850 (721 to 986)	205 (162 to 257)	191 (131 to 271)	71 (47 to 100)	75 (50 to 104)	8796 (6627 to 11200)	9859 (6163 to 14138)
American Samoa	19 (16 to 22)	22 (18 to 25)	2 (2 to 3)	1 (1 to 1)	2 (1 to 2)	2 (1 to 3)	74 (63 to 87)	36 (31 to 42)
Federated States of Micronesia	11 (9 to 13)	14 (12 to 16)	2 (1 to 3)	2 (1 to 2)	1 (1 to 1)	1 (1 to 2)	71 (45 to 98)	60 (34 to 87)
Fiji	102 (86 to 121)	111 (92 to 131)	21 (18 to 26)	14 (11 to 17)	9 (6 to 13)	9 (6 to 13)	767 (633 to 933)	519 (409 to 630)
Guam	35 (30 to 41)	44 (37 to 51)	4 (4 to 5)	2 (2 to 3)	3 (2 to 4)	4 (2 to 5)	139 (118 to 164)	72 (62 to 85)
Kiribati	6 (5 to 7)	8 (7 to 10)	2 (1 to 3)	1 (1 to 1)	1 (0 to 1)	1 (0 to 1)	84 (28 to 116)	42 (24 to 59)
Marshall Islands	5 (4 to 6)	7 (6 to 8)	1 (1 to 2)	1 (1 to 2)	0 (0 to 1)	1 (0 to 1)	47 (33 to 74)	51 (31 to 70)
Northern Mariana Islands	24 (20 to 28)	19 (16 to 23)	1 (1 to 2)	0 (0 to 1)	2 (1 to 3)	2 (1 to 2)	40 (32 to 47)	13 (10 to 15)
Papua New Guinea	446 (372 to 518)	479 (402 to 559)	142 (105 to 186)	148 (94 to 222)	40 (26 to 57)	43 (28 to 60)	6499 (4402 to 8751)	8114 (4651 to 12167)
Samoa	31 (26 to 36)	33 (28 to 38)	4 (2 to 5)	3 (2 to 4)	3 (2 to 4)	3 (2 to 4)	110 (69 to 152)	78 (55 to 105)
Solomon Islands	38 (32 to 45)	41 (35 to 48)	6 (4 to 9)	5 (3 to 7)	3 (2 to 5)	4 (2 to 5)	210 (137 to 315)	193 (119 to 282)
Tonga	13 (11 to 15)	5 (4 to 5)	1 (1 to 1)	0 (0 to 0)	1 (1 to 2)	0 (0 to 1)	33 (27 to 41)	3 (3 to 4)
Vanuatu	30 (25 to 35)	22 (18 to 25)	7 (3 to 12)	3 (2 to 5)	3 (2 to 4)	2 (1 to 3)	237 (104 to 404)	135 (94 to 203)
High-income Asia Pacific	159114 (135980 to 184549)	204838 (174210 to 239420)	2963 (2620 to 3609)	3877 (3052 to 4302)	13567 (9149 to 18992)	16951 (11448 to 23644)	59649 (51993 to 74984)	43181 (34042 to 48150)
Brunei	192 (163 to 227)	210 (178 to 244)	10 (8 to 12)	8 (7 to 10)	17 (11 to 24)	18 (12 to 25)	390 (327 to 476)	254 (219 to 290)
Japan	129343 (111165 to 151100)	177239 (149726 to 207368)	2487 (2204 to 3046)	3445 (2683 to 3856)	11009 (7407 to 15358)	14618 (9833 to 20478)	47662 (41649 to 59552)	36019 (27794 to 40568)
Singapore	2679 (2254 to 3169)	2622 (2222 to 3069)	61 (46 to 104)	31 (20 to 38)	232 (152 to 330)	224 (148 to 316)	1809 (1400 to 2840)	796 (582 to 973)
South Korea	26900 (22338 to 32427)	24767 (20676 to 29343)	405 (331 to 489)	393 (323 to 458)	2309 (1492 to 3432)	2091 (1365 to 3054)	9787 (8046 to 12446)	6112 (5129 to 7066)

High-income North America	270575 (233679 to 315339)	299531 (258561 to 344860)	13337 (12171 to 17097)	10939 (10373 to 11717)	22253 (15227 to 30850)	24549 (16815 to 33968)	281205 (251522 to 377740)	183469 (173017 to 199307)
Canada	22531 (18774 to 26748)	28824 (24249 to 33870)	509 (405 to 863)	404 (353 to 464)	1889 (1258 to 2642)	2406 (1627 to 3448)	10605 (8525 to 17063)	6636 (5841 to 7729)
Greenland	64 (52 to 78)	25 (21 to 30)	4 (4 to 5)	1 (1 to 1)	5 (4 to 8)	2 (1 to 3)	106 (91 to 123)	17 (14 to 23)
United States	247975 (212970 to 288619)	270676 (232242 to 312268)	12824 (11736 to 16262)	10534 (9978 to 11294)	20358 (13950 to 28161)	22140 (15246 to 30455)	270489 (241768 to 361922)	176812 (166533 to 192505)
Western Europe	394010 (333676 to 462170)	391192 (324768 to 467122)	13580 (9181 to 15004)	17659 (15793 to 20886)	32587 (21965 to 45347)	32020 (21152 to 44721)	204394 (182094 to 228109)	167183 (152757 to 187794)
Andorra	49 (42 to 59)	41 (35 to 49)	2 (1 to 2)	1 (1 to 2)	4 (3 to 6)	3 (2 to 5)	35 (21 to 51)	16 (12 to 22)
Austria	14368 (12274 to 16650)	16441 (13118 to 19829)	529 (257 to 639)	860 (741 to 988)	1172 (799 to 1653)	1331 (881 to 1901)	6076 (5122 to 7051)	6555 (5789 to 7425)
Belgium	8292 (6770 to 9764)	11506 (9225 to 14085)	312 (157 to 385)	550 (457 to 665)	676 (456 to 947)	928 (618 to 1335)	3885 (3160 to 4621)	4778 (4032 to 5587)
Cyprus	796 (668 to 953)	549 (469 to 640)	29 (21 to 34)	16 (12 to 23)	67 (44 to 96)	46 (31 to 66)	704 (524 to 828)	233 (195 to 289)
Denmark	1924 (1607 to 2278)	1859 (1561 to 2215)	80 (67 to 95)	66 (55 to 83)	162 (106 to 231)	156 (104 to 225)	1616 (1367 to 1931)	983 (835 to 1145)
Finland	2555 (2016 to 3223)	3760 (3020 to 4631)	150 (90 to 184)	111 (86 to 131)	210 (137 to 307)	310 (198 to 449)	2703 (1719 to 3254)	1667 (1399 to 1918)
France	67640 (54900 to 82790)	63921 (52081 to 78145)	2168 (1369 to 2552)	2884 (2441 to 3387)	5597 (3670 to 7851)	5249 (3479 to 7579)	29149 (25277 to 35810)	23922 (20773 to 27396)
Germany	38247 (31340 to 46558)	52303 (41988 to 65785)	2393 (1283 to 3071)	4086 (3307 to 4968)	3157 (2050 to 4646)	4265 (2803 to 6168)	33500 (24010 to 40539)	40212 (32645 to 48233)
Greece	8743 (7151 to 10575)	10566 (8475 to 13134)	346 (201 to 405)	502 (426 to 595)	724 (475 to 1031)	868 (565 to 1237)	6444 (4059 to 7450)	5403 (4712 to 6209)
Iceland	142 (119 to 169)	73 (60 to 87)	4 (2 to 5)	2 (1 to 3)	12 (8 to 17)	6 (4 to 9)	57 (49 to 68)	18 (15 to 22)
Ireland	3484 (2872 to 4171)	2548 (2106 to 3078)	102 (82 to 131)	86 (62 to 127)	289 (191 to 414)	209 (140 to 294)	1702 (1312 to 2754)	869 (654 to 1277)
Israel	4146 (3471 to 4993)	3350 (2823 to 3937)	83 (70 to 102)	84 (69 to 98)	349 (228 to 505)	280 (185 to 402)	1768 (1473 to 2508)	1207 (1047 to 1374)
Italy	145810 (117732 to 173500)	120642 (98786 to 148007)	2864 (1839 to 3442)	3191 (2302 to 4722)	12000 (7981 to 16793)	9823 (6616 to 13970)	37686 (32140 to 48119)	26680 (20993 to 35964)
Luxembourg	407 (336 to 489)	294 (238 to 364)	11 (8 to 15)	12 (10 to 16)	34 (23 to 48)	24 (16 to 35)	165 (126 to 310)	109 (85 to 138)
Malta	418 (337 to 507)	380 (312 to 457)	12 (10 to 14)	13 (10 to 17)	35 (22 to 50)	31 (20 to 43)	200 (165 to 289)	142 (114 to 187)
Netherlands	7350 (6076 to 8813)	10053 (8192 to 12304)	298 (253 to 361)	481 (395 to 592)	612 (401 to 875)	825 (538 to 1173)	4565 (3734 to 7189)	4940 (4252 to 5802)
Norway	1962 (1708 to 2265)	2017 (1709 to 2347)	75 (64 to 83)	86 (78 to 101)	164 (113 to 228)	166 (113 to 228)	1482 (1278 to 1706)	972 (888 to 1065)
Portugal	4167 (3465 to 5020)	6697 (5525 to 8087)	225 (131 to 268)	348 (289 to 416)	347 (229 to 494)	548 (365 to 779)	3758 (2654 to 4460)	3536 (3004 to 4069)
Spain	47616 (39095 to 57720)	42692 (34275 to 52215)	2348 (1393 to 2763)	2375 (1945 to 2943)	3968 (2562 to 5613)	3525 (2290 to 4920)	38352 (28443 to 43992)	22396 (19111 to 26400)
Sweden	7369 (6001 to 8993)	7006 (5788 to 8571)	195 (167 to 234)	218 (189 to 259)	618 (415 to 876)	582 (378 to 831)	3487 (2945 to 4438)	2618 (2311 to 3057)
Switzerland	3484 (2987 to 4050)	5265 (4396 to 6159)	150 (105 to 180)	196 (167 to 236)	290 (193 to 411)	432 (295 to 603)	2264 (1873 to 2727)	1976 (1721 to 2276)
United Kingdom	24633 (21092 to 28920)	28821 (24435 to 33634)	1192 (1105 to 1471)	1474 (1344 to 1756)	2068 (1407 to 2856)	2379 (1613 to 3281)	24584 (22746 to 31507)	17777 (16570 to 20581)
Australasia	15824 (13242 to 18934)	20778 (17286 to 24975)	606 (499 to 932)	417 (363 to 475)	1316 (887 to 1883)	1713 (1146 to 2418)	11872 (9645 to 19152)	6171 (5339 to 7112)
Australia	13210 (10967 to 15875)	18106 (14999 to 21923)	493 (398 to 746)	363 (311 to 421)	1100 (736 to 1583)	1492 (989 to 2132)	9162 (7163 to 15382)	5064 (4247 to 5959)
New Zealand	2614 (2195 to 3120)	2672 (2249 to 3171)	113 (94 to 188)	54 (47 to 62)	216 (146 to 308)	221 (147 to 318)	2710 (2319 to 4043)	1106 (980 to 1249)

Data in parentheses are 95% uncertainty intervals. YLDs= years lived with disability; YLLs= years of life lost; SDI= Socio-demographic Index.

Supplementary Table 5. Age-standardised prevalence, death, YLD, and YLL rates of myocarditis for both sexes in 2017, and their percentage changes from 1990 to 2017, by location.

	Prevalence		Deaths		YLDs		YLLs	
	2017 age-standardised rate per 100 000 people	Percentage change in age-standardised rates, 1990–2017	2017 age-standardised rate per 100 000 people	Percentage change in age-standardised rates, 1990–2017	2017 age-standardised rate per 100 000 people	Percentage change in age-standardised rates, 1990–2017	2017 age-standardised rate per 100 000 people	Percentage change in age-standardised rates, 1990–2017
Central Sub-Saharan Africa	16.86 (15.13 to 18.62)	1.3% (-1.4 to 3.8)	0.37 (0.20 to 0.59)	-26.2% (-46.2 to 2.2)	1.12 (0.76 to 1.57)	1.8% (-4.1 to 8.4)	12.14 (6.96 to 19.85)	-34.4% (-55.0 to -5.9)
Angola	17.56 (15.70 to 19.49)	3.8% (0.1 to 7.5)	0.38 (0.22 to 0.68)	-32.5% (-58.7 to 14.6)	1.17 (0.79 to 1.66)	4.4% (-5.8 to 15.8)	12.25 (7.37 to 21.68)	-44.9% (-67.4 to -0.9)
Central African Republic	16.66 (14.87 to 18.39)	2.6% (-0.7 to 6.0)	0.47 (0.25 to 0.81)	-22.0% (-46.2 to 17.0)	1.10 (0.74 to 1.56)	2.5% (-7.2 to 12.4)	17.83 (9.47 to 33.01)	-19.8% (-47.3 to 29.8)
Congo	17.73 (15.81 to 19.55)	0.7% (-3.0 to 4.3)	0.47 (0.22 to 0.90)	-23.5% (-53.5 to 21.1)	1.18 (0.81 to 1.66)	0.8% (-9.4 to 12.4)	14.55 (7.51 to 27.08)	-31.3% (-60.4 to 9.9)
Democratic Republic of the Congo	16.53 (14.81 to 18.26)	0.4% (-3.1 to 4.1)	0.35 (0.17 to 0.56)	-24.0% (-47.2 to 5.8)	1.10 (0.74 to 1.53)	1.0% (-7.1 to 9.8)	11.64 (6.14 to 18.99)	-31.5% (-55.9 to -1.0)
Equatorial Guinea	17.66 (15.87 to 19.47)	8.4% (4.5 to 13.0)	0.35 (0.17 to 0.76)	-38.0% (-73.1 to 20.8)	1.18 (0.81 to 1.65)	10.2% (-0.6 to 21.9)	10.01 (4.83 to 21.71)	-54.4% (-79.3 to -10.7)
Gabon	18.85 (16.81 to 20.82)	-0.7% (-4.5 to 3.2)	0.41 (0.23 to 0.86)	-26.9% (-52.9 to 8.5)	1.28 (0.87 to 1.79)	-0.6% (-9.8 to 10.3)	12.52 (7.25 to 25.20)	-30.5% (-56.4 to 6.7)
Eastern Sub-Saharan Africa	17.41 (15.67 to 19.15)	4.3% (2.9 to 5.7)	0.30 (0.13 to 0.60)	-36.3% (-58.4 to -10.7)	1.17 (0.80 to 1.62)	5.0% (1.6 to 8.2)	10.55 (4.97 to 19.03)	-45.0% (-66.4 to -18.0)
Burundi	14.85 (13.29 to 16.40)	-0.8% (-3.7 to 2.7)	0.28 (0.12 to 0.59)	-53.5% (-68.8 to -35.6)	0.95 (0.64 to 1.35)	-0.7% (-9.8 to 9.2)	10.17 (4.58 to 20.53)	-57.2% (-72.9 to -39.7)
Comoros	16.51 (14.70 to 18.30)	1.6% (-2.3 to 5.5)	0.27 (0.10 to 0.56)	-49.5% (-71.0 to -20.4)	1.10 (0.75 to 1.54)	1.5% (-7.3 to 11.4)	9.22 (3.73 to 17.52)	-57.3% (-76.6 to -29.6)
Djibouti	17.00 (15.14 to 18.77)	6.9% (3.1 to 10.8)	0.33 (0.12 to 0.76)	-44.1% (-68.2 to -13.7)	1.14 (0.77 to 1.60)	7.7% (-3.1 to 19.2)	10.78 (4.05 to 23.54)	-50.7% (-73.2 to -20.0)
Eritrea	16.14 (14.47 to 17.95)	1.9% (-1.5 to 5.4)	0.50 (0.15 to 1.05)	-39.7% (-67.5 to 3.0)	1.07 (0.72 to 1.49)	2.3% (-6.5 to 12.3)	18.14 (5.45 to 35.44)	-43.4% (-72.6 to 6.7)
Ethiopia	23.17 (20.69 to 25.75)	3.6% (0.2 to 7.1)	0.26 (0.10 to 0.50)	-47.4% (-71.9 to -7.9)	1.66 (1.12 to 2.28)	4.4% (-2.7 to 11.5)	8.59 (3.71 to 15.18)	-57.4% (-77.3 to -24.1)
Kenya	15.01 (13.51 to 16.60)	3.4% (2.6 to 4.2)	0.29 (0.11 to 0.63)	-21.2% (-42.3 to 6.3)	0.95 (0.65 to 1.33)	3.8% (1.9 to 5.8)	9.45 (3.80 to 18.55)	-27.2% (-50.0 to 3.3)
Madagascar	15.78 (14.06 to 17.45)	1.8% (-1.6 to 6.0)	0.55 (0.23 to 1.07)	-33.6% (-52.3 to -10.3)	1.04 (0.70 to 1.48)	1.8% (-6.3 to 10.9)	20.58 (9.13 to 39.12)	-42.4% (-60.1 to -19.1)
Malawi	15.22 (13.58 to 16.81)	8.1% (4.4 to 12.0)	0.21 (0.11 to 0.40)	-33.5% (-61.7 to 15.1)	0.97 (0.66 to 1.36)	9.3% (-0.1 to 20.5)	7.51 (4.10 to 14.12)	-46.5% (-72.6 to 11.9)
Mozambique	13.07 (11.77 to 14.48)	4.7% (1.7 to 8.0)	0.29 (0.14 to 0.64)	-23.8% (-55.3 to 18.1)	0.78 (0.51 to 1.12)	5.9% (-5.5 to 19.1)	10.09 (5.02 to 21.34)	-41.6% (-68.4 to -4.4)
Rwanda	15.35 (13.77 to 16.91)	-0.8% (-4.1 to 2.4)	0.29 (0.11 to 0.62)	-52.1% (-71.4 to -30.7)	1.00 (0.67 to 1.43)	-0.4% (-9.1 to 8.2)	9.13 (3.63 to 17.86)	-57.6% (-75.6 to -35.6)
Somalia	15.59 (13.98 to 17.30)	3.6% (0.2 to 7.4)	0.33 (0.16 to 0.61)	-41.7% (-66.3 to 3.8)	1.03 (0.70 to 1.46)	4.3% (-4.0 to 12.9)	12.14 (5.89 to 22.13)	-48.7% (-73.3 to 9.0)
South Sudan	15.67 (14.01 to 17.40)	4.0% (0.4 to 7.8)	0.32 (0.15 to 0.62)	-39.1% (-61.8 to -1.7)	1.03 (0.70 to 1.46)	4.4% (-4.5 to 14.5)	12.26 (5.72 to 23.25)	-38.7% (-62.8 to 7.4)
Tanzania	16.39 (14.58 to 18.16)	7.2% (3.6 to 11.1)	0.30 (0.12 to 0.63)	-34.6% (-61.8 to -1.0)	1.08 (0.73 to 1.54)	8.2% (-1.3 to 18.8)	10.62 (4.73 to 20.09)	-39.7% (-66.5 to 0.2)
Uganda	15.59 (14.02 to 17.25)	-1.5% (-4.8 to 1.8)	0.28 (0.10 to 0.57)	-17.0% (-52.7 to 32.5)	1.01 (0.69 to 1.44)	-1.0% (-11.2 to 9.4)	9.55 (3.69 to 18.10)	-26.7% (-62.9 to 22.8)
Zambia	16.03 (14.34 to 17.73)	9.8% (6.0 to 14.2)	0.31 (0.12 to 0.67)	-32.3% (-60.5 to 6.6)	1.06 (0.72 to 1.49)	11.4% (2.1 to 22.3)	10.65 (4.22 to 21.75)	-43.7% (-70.6 to -6.9)
Southern Sub-Saharan Africa	17.34 (15.71 to 19.13)	2.7% (1.3 to 3.9)	0.31 (0.25 to 0.47)	-27.3% (-43.3 to 0.0)	1.16 (0.79 to 1.64)	3.0% (0.1 to 6.1)	12.31 (9.35 to 19.12)	-30.0% (-48.0 to 5.2)
Botswana	20.52 (18.42 to 22.57)	4.7% (1.0 to 8.5)	0.33 (0.22 to 0.48)	-32.1% (-55.0 to 0.8)	1.44 (0.98 to 2.04)	5.5% (-2.2 to 14.3)	10.24 (6.62 to 14.82)	-38.2% (-60.9 to -3.4)
Lesotho	18.31 (16.52 to 20.19)	3.8% (0.1 to 7.7)	0.53 (0.36 to 0.74)	-2.4% (-40.5 to 54.0)	1.25 (0.85 to 1.77)	4.1% (-3.9 to 13.1)	18.16 (12.00 to 26.39)	-0.4% (-38.1 to 61.3)
Namibia	20.04 (18.02 to 22.10)	3.6% (-0.1 to 7.1)	0.37 (0.20 to 0.56)	-38.7% (-61.0 to 0.7)	1.41 (0.95 to 1.99)	4.2% (-4.3 to 13.0)	12.79 (6.46 to 19.74)	-37.5% (-61.2 to 5.6)
South Africa	16.92 (15.31 to 18.62)	4.1% (2.6 to 5.6)	0.22 (0.19 to 0.31)	-43.6% (-55.3 to -14.8)	1.11 (0.76 to 1.57)	4.9% (1.3 to 8.6)	8.10 (6.57 to 12.08)	-52.5% (-64.8 to -19.4)
Swaziland	18.08 (16.26 to 19.92)	1.9% (-1.2 to 5.4)	0.44 (0.31 to 0.63)	-27.0% (-49.1 to 1.8)	1.23 (0.84 to 1.73)	2.3% (-5.6 to 11.0)	16.24 (11.01 to 23.33)	-23.0% (-47.3 to 8.2)
Zimbabwe	17.80 (15.88 to 19.81)	-3.9% (-8.6 to 0.4)	0.72 (0.41 to 1.48)	27.4% (-5.5 to 83.8)	1.24 (0.85 to 1.73)	-4.4% (-12.5 to 3.8)	28.30 (16.97 to 52.15)	43.1% (7.1 to 102.5)
Western Sub-Saharan Africa	19.63 (17.53 to 21.76)	4.5% (2.0 to 7.0)	0.40 (0.31 to 0.49)	-50.5% (-62.7 to -30.1)	1.36 (0.93 to 1.89)	5.5% (0.3 to 10.7)	11.05 (8.72 to 13.42)	-54.0% (-65.2 to -35.2)
Benin	19.12 (17.03 to 21.31)	4.4% (0.1 to 8.9)	0.43 (0.29 to 0.62)	-43.8% (-61.2 to -19.9)	1.32 (0.89 to 1.89)	5.8% (-4.1 to 15.9)	11.77 (7.89 to 17.28)	-49.2% (-64.1 to -30.1)
Burkina Faso	18.62 (16.65 to 20.78)	4.7% (1.0 to 8.9)	0.46 (0.31 to 0.68)	-37.8% (-56.4 to -11.5)	1.27 (0.86 to 1.80)	6.2% (-2.4 to 16.1)	12.83 (8.72 to 18.56)	-46.5% (-61.4 to -25.1)
Cameroon	19.20 (17.19 to 21.44)	3.4% (-2.5 to 9.5)	0.51 (0.38 to 0.70)	-50.4% (-67.4 to -26.0)	1.32 (0.88 to 1.87)	4.2% (-6.5 to 15.9)	14.54 (10.89 to 19.71)	-50.8% (-66.2 to -28.3)
Cape Verde	28.89 (25.32 to 32.60)	6.8% (0.8 to 12.9)	0.25 (0.20 to 0.31)	1.3% (-30.5 to 46.1)	2.16 (1.46 to 3.02)	7.5% (-2.4 to 19.8)	7.73 (6.33 to 9.57)	-20.8% (-45.1 to 15.1)
Chad	18.63 (16.63 to 20.87)	1.5% (-2.9 to 6.0)	0.42 (0.27 to 0.64)	-43.1% (-59.6 to -17.3)	1.28 (0.86 to 1.80)	2.4% (-7.2 to 12.0)	12.75 (8.38 to 19.53)	-42.4% (-58.5 to -15.7)
Cote d'Ivoire	19.47 (17.30 to 21.73)	1.3% (-2.8 to 5.7)	0.47 (0.33 to 0.66)	-34.5% (-53.8 to -6.2)	1.33 (0.91 to 1.86)	2.6% (-6.5 to 12.3)	14.53 (10.20 to 20.81)	-35.2% (-54.5 to -7.4)
The Gambia	20.14 (17.98 to 22.53)	3.4% (-0.7 to 8.1)	0.40 (0.29 to 0.55)	-39.8% (-60.3 to -7.5)	1.39 (0.94 to 1.96)	4.4% (-5.1 to 14.4)	11.51 (8.14 to 16.92)	-38.9% (-58.9 to -7.3)
Ghana	17.21 (15.27 to 19.19)	5.0% (0.6 to 9.9)	0.68 (0.49 to 0.92)	-18.3% (-52.1 to 77.5)	1.14 (0.77 to 1.61)	6.5% (-3.0 to 16.4)	14.96 (11.37 to 19.97)	-35.9% (-61.7 to 40.7)
Guinea	16.26 (14.61 to 18.09)	6.6% (2.2 to 11.2)	0.46 (0.30 to 0.68)	-46.3% (-60.6 to -23.0)	1.07 (0.72 to 1.51)	8.1% (-1.1 to 18.6)	13.84 (9.16 to 20.10)	-52.5% (-64.9 to -32.7)
Guinea-Bissau	19.22 (17.21 to 21.50)	7.5% (3.2 to 12.2)	0.52 (0.36 to 0.70)	-53.2% (-67.2 to -33.0)	1.31 (0.89 to 1.85)	9.0% (-0.4 to 18.9)	15.67 (11.26 to 21.44)	-54.5% (-68.1 to -34.9)
Liberia	18.70 (16.71 to 20.77)	5.4% (1.5 to 9.4)	0.34 (0.21 to 0.47)	-53.1% (-66.2 to -34.8)	1.26 (0.85 to 1.79)	6.4% (-1.7 to 15.5)	9.83 (6.55 to 13.55)	-62.9% (-73.8 to -46.9)
Mali	18.51 (16.50 to 20.56)	2.2% (-1.9 to 6.0)	0.46 (0.27 to 0.76)	-54.4% (-66.6 to -34.4)	1.28 (0.87 to 1.79)	3.2% (-5.2 to 12.1)	14.30 (8.32 to 22.55)	-57.9% (-69.6 to -39.4)
Mauritania	19.93 (17.80 to 22.21)	-2.5% (-6.5 to 1.5)	0.39 (0.27 to 0.58)	-52.9% (-68.1 to -30.4)	1.39 (0.94 to 1.95)	-2.4% (-10.6 to 6.7)	9.95 (6.74 to 14.89)	-55.4% (-69.1 to -35.4)
Niger	18.63 (16.65 to 20.73)	1.7% (-2.2 to 5.6)	0.36 (0.20 to 0.64)	-56.5% (-69.3 to -35.6)	1.28 (0.87 to 1.79)	2.0% (-6.4 to 11.3)	10.39 (5.62 to 18.06)	-62.9% (-74.6 to -44.3)

Nigeria	20.50 (18.28 to 22.77)	5.5% (1.0 to 9.9)	0.33 (0.20 to 0.47)	-58.6% (-74.7 to -34.2)	1.43 (0.97 to 1.99)	6.6% (-2.8 to 16.5)	8.83 (5.50 to 13.35)	-61.2% (-76.4 to -37.8)
Sao Tome and Principe	19.78 (17.64 to 22.13)	7.9% (3.4 to 13.3)	0.40 (0.29 to 0.55)	-34.6% (-54.1 to -2.2)	1.36 (0.91 to 1.89)	8.8% (0.0 to 18.9)	13.35 (9.37 to 18.54)	-40.4% (-59.3 to -11.7)
Senegal	22.39 (19.79 to 25.16)	5.2% (0.3 to 10.5)	0.32 (0.22 to 0.45)	-45.2% (-62.0 to -18.8)	1.58 (1.08 to 2.21)	6.1% (-2.8 to 15.5)	8.79 (6.07 to 12.13)	-48.4% (-63.7 to -24.7)
Sierra Leone	18.54 (16.52 to 20.66)	6.3% (2.0 to 10.5)	0.40 (0.30 to 0.54)	-40.5% (-59.1 to -11.6)	1.25 (0.85 to 1.77)	7.5% (-1.6 to 16.6)	12.85 (9.56 to 16.66)	-48.5% (-64.7 to -22.8)
Togo	19.86 (17.68 to 22.23)	6.2% (2.0 to 10.7)	0.39 (0.28 to 0.54)	-48.7% (-64.4 to -28.9)	1.37 (0.92 to 1.93)	7.6% (-1.8 to 19.0)	10.71 (7.45 to 14.91)	-52.4% (-65.5 to -33.7)
Andean Latin America	17.22 (15.51 to 18.96)	10.6% (7.7 to 13.6)	0.21 (0.18 to 0.24)	-51.1% (-62.4 to -26.1)	1.15 (0.77 to 1.63)	12.7% (5.5 to 20.5)	6.11 (5.22 to 7.48)	-57.9% (-68.6 to -36.3)
Bolivia	13.71 (11.93 to 15.69)	19.8% (13.4 to 27.3)	0.37 (0.27 to 0.51)	-46.5% (-64.0 to -18.2)	0.84 (0.55 to 1.22)	29.3% (12.6 to 49.8)	9.96 (7.00 to 14.00)	-57.9% (-72.8 to -33.3)
Ecuador	15.89 (14.34 to 17.58)	8.5% (4.6 to 12.5)	0.23 (0.18 to 0.33)	-34.3% (-58.5 to 32.4)	1.03 (0.69 to 1.48)	10.0% (-0.4 to 21.2)	6.86 (5.29 to 9.95)	-39.7% (-61.6 to 21.4)
Peru	19.01 (17.07 to 20.83)	10.9% (6.6 to 15.0)	0.15 (0.12 to 0.19)	-59.6% (-70.1 to -42.2)	1.31 (0.87 to 1.85)	12.7% (2.4 to 23.4)	4.55 (3.57 to 5.65)	-65.7% (-74.5 to -50.7)
Tropical Latin America	16.28 (14.78 to 17.88)	2.9% (1.7 to 4.3)	0.31 (0.26 to 0.45)	-6.0% (-16.5 to 10.0)	1.00 (0.67 to 1.41)	4.1% (0.5 to 7.8)	9.95 (8.13 to 14.96)	-23.8% (-37.7 to 4.7)
Brazil	16.25 (14.76 to 17.85)	2.7% (1.5 to 4.1)	0.31 (0.26 to 0.46)	-6.5% (-17.3 to 9.8)	1.00 (0.67 to 1.41)	3.8% (0.2 to 7.5)	10.14 (8.24 to 15.31)	-24.0% (-38.2 to 5.4)
Paraguay	16.94 (15.29 to 18.65)	7.1% (3.6 to 10.8)	0.16 (0.12 to 0.20)	29.9% (-14.0 to 81.8)	1.09 (0.73 to 1.53)	9.0% (-1.0 to 20.2)	5.14 (3.81 to 6.43)	9.7% (-24.3 to 51.5)
Central Latin America	18.06 (16.28 to 19.88)	3.0% (1.6 to 4.6)	0.21 (0.19 to 0.26)	-18.1% (-30.5 to 3.8)	1.21 (0.83 to 1.73)	4.2% (1.3 to 7.0)	7.84 (7.04 to 9.54)	-22.6% (-35.2 to 3.9)
Colombia	18.89 (17.05 to 20.80)	2.1% (-1.2 to 5.7)	0.20 (0.16 to 0.27)	-24.0% (-42.1 to 7.5)	1.30 (0.88 to 1.84)	2.6% (-5.2 to 11.0)	9.23 (6.80 to 11.74)	-25.9% (-43.5 to 3.8)
Costa Rica	23.90 (21.32 to 26.60)	1.8% (-2.0 to 5.4)	0.31 (0.25 to 0.44)	-11.4% (-32.4 to 19.4)	1.74 (1.17 to 2.46)	1.9% (-4.7 to 8.8)	12.39 (8.95 to 15.05)	-16.4% (-36.1 to 19.6)
El Salvador	19.91 (17.91 to 22.10)	7.9% (3.6 to 12.4)	0.40 (0.26 to 0.53)	-0.1% (-27.0 to 36.9)	1.39 (0.94 to 1.96)	9.7% (-2.1 to 21.7)	12.33 (8.30 to 16.29)	-10.2% (-34.5 to 23.8)
Guatemala	18.78 (16.92 to 20.83)	5.6% (1.7 to 9.9)	0.33 (0.27 to 0.40)	4.5% (-17.1 to 51.4)	1.30 (0.88 to 1.85)	6.9% (-1.5 to 16.7)	11.29 (9.44 to 14.15)	-2.3% (-22.2 to 45.5)
Honduras	17.56 (15.76 to 19.35)	8.1% (4.5 to 12.0)	0.46 (0.31 to 0.69)	-31.0% (-52.0 to -6.6)	1.16 (0.79 to 1.66)	10.7% (1.3 to 22.2)	13.09 (8.57 to 19.70)	-44.4% (-63.8 to -18.5)
Mexico	18.17 (16.37 to 20.04)	3.6% (2.4 to 5.0)	0.20 (0.18 to 0.25)	-5.9% (-16.1 to 13.0)	1.22 (0.83 to 1.74)	5.2% (2.9 to 7.8)	7.26 (6.39 to 9.54)	-12.5% (-26.5 to 19.8)
Nicaragua	15.68 (14.16 to 17.35)	10.2% (6.5 to 14.3)	0.11 (0.09 to 0.14)	-43.5% (-59.0 to -16.9)	0.97 (0.66 to 1.39)	12.1% (0.7 to 24.9)	3.86 (3.08 to 4.73)	-50.8% (-64.7 to -27.1)
Panama	15.44 (14.04 to 16.89)	1.7% (-1.5 to 5.2)	0.14 (0.11 to 0.20)	-3.3% (-30.7 to 59.4)	0.97 (0.65 to 1.38)	2.0% (-8.0 to 12.6)	5.40 (4.35 to 6.92)	-4.2% (-31.7 to 53.8)
Venezuela	16.45 (14.68 to 18.29)	4.7% (0.8 to 8.9)	0.15 (0.11 to 0.24)	-46.2% (-68.6 to 5.3)	1.04 (0.69 to 1.48)	7.6% (-2.6 to 19.0)	5.52 (4.20 to 7.93)	-45.9% (-67.5 to 6.8)
Southern Latin America	11.42 (9.87 to 12.96)	-3.5% (-8.5 to 2.1)	0.32 (0.27 to 0.40)	-34.3% (-49.9 to -14.2)	0.80 (0.53 to 1.15)	-3.7% (-11.1 to 5.5)	10.26 (8.52 to 13.78)	-44.1% (-57.1 to -25.2)
Argentina	11.68 (9.91 to 13.45)	1.0% (-5.3 to 8.2)	0.39 (0.33 to 0.50)	-36.7% (-53.5 to -13.5)	0.82 (0.54 to 1.19)	2.1% (-8.4 to 15.2)	12.39 (10.01 to 17.19)	-47.6% (-61.5 to -27.5)
Chile	10.17 (9.02 to 11.37)	-6.9% (-12.0 to -1.0)	0.14 (0.11 to 0.16)	6.1% (-14.0 to 28.1)	0.69 (0.46 to 0.98)	-7.9% (-16.8 to 3.2)	4.51 (3.75 to 5.54)	-11.5% (-27.4 to 9.4)
Uruguay	15.31 (13.55 to 17.11)	-12.0% (-16.5 to -6.4)	0.37 (0.29 to 0.45)	-18.5% (-36.8 to 1.5)	1.15 (0.77 to 1.62)	-14.0% (-20.3 to -6.4)	13.01 (9.89 to 15.90)	-30.9% (-47.1 to -13.0)
Caribbean	26.27 (23.80 to 28.83)	4.8% (2.4 to 7.4)	0.84 (0.72 to 0.99)	-15.7% (-27.3 to -1.1)	1.86 (1.28 to 2.59)	4.6% (-0.8 to 10.2)	22.54 (18.70 to 27.23)	-23.5% (-37.7 to -7.7)
Antigua and Barbuda	33.66 (29.93 to 37.46)	0.1% (-5.2 to 5.3)	1.62 (1.33 to 2.05)	-2.0% (-26.3 to 35.7)	2.55 (1.72 to 3.53)	0.2% (-9.9 to 10.9)	34.55 (29.10 to 43.08)	0.1% (-20.4 to 28.2)
The Bahamas	16.97 (15.44 to 18.65)	-2.6% (-6.1 to 0.6)	0.65 (0.53 to 0.81)	-11.9% (-33.9 to 15.3)	1.07 (0.71 to 1.51)	-3.1% (-13.2 to 8.2)	15.19 (12.29 to 18.75)	-10.7% (-28.7 to 12.0)
Barbados	26.45 (23.80 to 29.08)	-3.4% (-7.7 to 1.6)	0.88 (0.71 to 1.11)	-11.2% (-32.1 to 12.0)	1.89 (1.27 to 2.65)	-4.0% (-14.1 to 8.0)	17.28 (14.40 to 20.96)	-12.8% (-27.7 to 3.1)
Belize	28.40 (25.49 to 31.48)	3.0% (-2.3 to 8.1)	1.23 (0.98 to 1.63)	3.7% (-26.0 to 54.2)	2.07 (1.42 to 2.85)	3.4% (-7.3 to 15.8)	28.47 (23.53 to 37.05)	6.7% (-19.6 to 64.7)
Bermuda	22.32 (20.31 to 24.45)	-3.7% (-7.2 to -0.3)	0.43 (0.35 to 0.56)	-26.7% (-45.3 to 12.8)	1.55 (1.07 to 2.17)	-4.8% (-13.8 to 5.3)	9.69 (7.78 to 12.48)	-29.0% (-52.1 to 8.9)
Cuba	29.19 (26.35 to 32.05)	10.4% (5.5 to 15.2)	0.51 (0.38 to 0.89)	-6.2% (-28.8 to 25.1)	2.07 (1.40 to 2.86)	10.0% (-1.5 to 22.0)	11.32 (9.12 to 17.03)	-20.7% (-37.2 to 3.6)
Dominica	19.85 (18.01 to 21.82)	-2.8% (-6.8 to 1.3)	1.21 (0.95 to 1.57)	-6.7% (-35.9 to 44.9)	1.32 (0.89 to 1.84)	-3.7% (-14.5 to 8.8)	26.06 (21.40 to 33.84)	3.9% (-21.6 to 51.9)
Dominican Republic	26.27 (23.47 to 29.17)	10.0% (3.8 to 15.9)	0.80 (0.63 to 1.00)	-27.9% (-49.6 to 2.9)	1.89 (1.28 to 2.63)	11.1% (-1.2 to 25.8)	15.70 (12.49 to 19.73)	-35.4% (-53.6 to -8.5)
Grenada	33.06 (29.61 to 36.81)	-4.5% (-10.0 to 1.1)	1.92 (1.56 to 2.43)	-40.9% (-55.5 to -12.2)	2.46 (1.68 to 3.40)	-5.0% (-14.9 to 5.7)	37.22 (31.95 to 46.72)	-41.2% (-54.7 to -17.1)
Guyana	33.02 (29.22 to 36.92)	0.5% (-4.7 to 5.5)	3.08 (2.51 to 3.63)	45.8% (-2.0 to 112.1)	2.47 (1.69 to 3.45)	0.6% (-9.4 to 11.1)	74.90 (59.09 to 89.30)	48.3% (-8.1 to 119.5)
Haiti	22.07 (20.01 to 24.37)	4.4% (0.2 to 8.8)	1.84 (1.16 to 2.75)	-22.6% (-42.8 to 5.0)	1.52 (1.04 to 2.15)	5.1% (-5.5 to 17.9)	46.57 (30.49 to 68.06)	-35.0% (-52.9 to -11.3)
Jamaica	23.18 (20.85 to 25.59)	6.1% (1.9 to 10.8)	0.56 (0.43 to 0.77)	-15.8% (-40.4 to 23.8)	1.63 (1.09 to 2.27)	6.7% (-4.0 to 18.1)	13.68 (10.85 to 18.24)	-16.6% (-39.1 to 25.0)
Puerto Rico	29.02 (26.11 to 31.99)	2.7% (-1.5 to 7.4)	0.51 (0.42 to 0.65)	-21.4% (-45.5 to 2.2)	2.14 (1.47 to 3.01)	2.7% (-8.1 to 16.0)	12.17 (10.39 to 14.95)	-23.1% (-40.8 to -2.9)
Saint Lucia	30.20 (27.10 to 33.51)	-3.1% (-7.4 to 2.0)	1.64 (1.34 to 2.00)	-9.9% (-29.0 to 14.4)	2.20 (1.49 to 3.02)	-3.7% (-12.8 to 8.1)	33.00 (27.79 to 40.14)	-7.0% (-25.4 to 16.6)
Saint Vincent and the Grenadines	24.63 (22.15 to 27.22)	-0.7% (-4.9 to 3.1)	1.00 (0.85 to 1.28)	-37.6% (-55.6 to 10.2)	1.75 (1.19 to 2.44)	-0.9% (-10.3 to 9.2)	26.80 (22.54 to 34.11)	-26.9% (-47.6 to 31.3)
Suriname	26.37 (23.68 to 29.36)	4.4% (-0.7 to 9.8)	1.27 (1.02 to 1.63)	4.6% (-17.1 to 31.4)	1.90 (1.29 to 2.67)	4.6% (-6.5 to 16.9)	28.60 (24.23 to 35.57)	-5.7% (-23.3 to 18.1)
Trinidad and Tobago	31.26 (27.77 to 34.82)	-0.8% (-5.1 to 3.7)	1.01 (0.81 to 1.28)	-36.3% (-53.1 to -7.3)	2.33 (1.59 to 3.28)	-1.0% (-11.1 to 10.9)	28.56 (22.76 to 35.56)	-28.6% (-44.7 to -1.1)
Virgin Islands, U.S.	28.52 (25.63 to 31.49)	5.2% (1.0 to 10.0)	1.42 (1.09 to 1.74)	41.2% (9.0 to 88.8)	2.05 (1.39 to 2.83)	5.6% (-2.9 to 15.5)	29.26 (22.80 to 36.03)	38.0% (4.6 to 79.4)
Central Europe	42.80 (38.39 to 47.25)	-1.5% (-3.7 to 0.8)	1.64 (1.42 to 1.98)	-30.6% (-46.5 to 3.4)	3.28 (2.25 to 4.45)	-1.0% (-5.5 to 4.4)	32.08 (26.66 to 35.39)	-29.9% (-42.6 to -4.0)
Albania	105.63 (90.78 to 120.85)	1.7% (-4.7 to 8.8)	4.31 (3.41 to 5.37)	-51.1% (-63.3 to -30.1)	8.54 (5.82 to 11.81)	2.1% (-9.2 to 14.0)	87.93 (70.65 to 110.10)	-47.2% (-60.2 to -25.2)
Bosnia and Herzegovina	50.26 (44.77 to 56.14)	-2.7% (-7.2 to 2.3)	2.40 (1.36 to 3.44)	54.8% (16.1 to 113.6)	3.90 (2.65 to 5.46)	-2.3% (-12.7 to 10.3)	38.84 (21.51 to 54.15)	31.5% (5.6 to 73.3)
Bulgaria	38.78 (34.34 to 43.18)	-5.9% (-10.1 to -1.6)	1.98 (1.71 to 2.42)	61.0% (13.0 to 130.8)	3.02 (2.05 to 4.22)	-5.3% (-15.2 to 5.6)	46.22 (40.28 to 56.61)	41.1% (9.3 to 102.8)
Croatia	61.86 (54.80 to 69.50)	-22.5% (-29.1 to -15.8)	3.21 (2.27 to 3.94)	24.7% (-5.3 to 54.6)	4.81 (3.28 to 6.63)	-22.3% (-32.4 to -12.6)	35.47 (30.08 to 41.98)	-9.3% (-32.6 to 12.3)
Czech Republic	42.49 (38.07 to 47.12)	-6.1% (-11.4 to -0.3)	0.97 (0.71 to 1.14)	85.1% (-22.3 to 253.7)	3.28 (2.25 to 4.54)	-5.7% (-17.8 to 7.8)	19.78 (14.42 to 23.49)	31.8% (-41.7 to 126.9)
Hungary	32.22 (29.11 to 35.62)	1.1% (-2.8 to 5.1)	1.02 (0.62 to 1.26)	13.8% (-53.9 to 63.4)	2.27 (1.54 to 3.15)	2.5% (-9.1 to 14.9)	16.65 (10.18 to 20.09)	-11.3% (-56.1 to 19.3)

Macedonia	17.46 (15.93 to 19.07)	-10.0% (-12.8 to -7.0)	0.36 (0.24 to 0.49)	-4.2% (-27.4 to 23.2)	1.15 (0.78 to 1.63)	-9.7% (-16.4 to -2.1)	12.07 (5.88 to 18.16)	-24.0% (-48.7 to 6.6)
Montenegro	30.83 (28.19 to 33.77)	-0.2% (-3.5 to 3.1)	0.92 (0.60 to 1.27)	14.9% (-20.1 to 59.2)	2.11 (1.45 to 2.92)	0.3% (-9.3 to 11.0)	14.17 (9.71 to 18.15)	-15.7% (-34.5 to 6.8)
Poland	39.08 (35.06 to 43.28)	6.9% (2.3 to 11.5)	0.66 (0.36 to 1.55)	-61.3% (-83.4 to 4.6)	2.96 (2.02 to 4.08)	7.5% (-4.1 to 20.7)	12.95 (9.19 to 22.93)	-58.7% (-75.9 to -10.0)
Romania	52.50 (46.24 to 59.49)	-1.0% (-6.0 to 5.0)	4.07 (3.02 to 4.84)	-39.7% (-56.0 to -6.4)	4.10 (2.82 to 5.72)	-0.3% (-10.4 to 11.3)	82.14 (55.23 to 102.87)	-22.3% (-41.2 to 8.1)
Serbia	29.42 (26.43 to 32.65)	-14.7% (-18.6 to -10.8)	0.86 (0.46 to 1.94)	-23.8% (-61.2 to 83.0)	2.23 (1.49 to 3.14)	-14.2% (-23.6 to -4.1)	18.20 (13.00 to 26.79)	-56.4% (-72.7 to 1.7)
Slovakia	46.22 (41.32 to 51.19)	9.4% (3.3 to 16.1)	0.82 (0.69 to 1.02)	-38.9% (-54.9 to -1.0)	3.51 (2.36 to 4.86)	9.8% (-2.9 to 23.3)	18.00 (13.90 to 26.55)	-51.1% (-65.6 to -18.8)
Slovenia	32.03 (28.95 to 35.39)	-11.5% (-15.3 to -7.2)	1.51 (0.59 to 2.23)	158.2% (-52.3 to 1026.2)	2.43 (1.65 to 3.34)	-11.4% (-22.4 to 1.7)	17.66 (8.54 to 24.08)	76.4% (-58.4 to 404.3)
Eastern Europe	18.44 (16.62 to 20.36)	-8.7% (-10.5 to -6.8)	0.64 (0.52 to 0.94)	30.4% (10.3 to 84.8)	1.15 (0.78 to 1.62)	-6.5% (-9.4 to -3.7)	14.00 (11.91 to 17.32)	40.2% (19.4 to 69.8)
Belarus	14.72 (13.35 to 16.20)	-0.9% (-4.5 to 2.9)	0.22 (0.12 to 0.38)	-32.9% (-58.3 to 20.5)	0.95 (0.64 to 1.34)	0.7% (-8.5 to 10.6)	6.52 (3.91 to 9.79)	-29.9% (-54.6 to 25.6)
Estonia	14.12 (12.76 to 15.56)	8.4% (3.6 to 13.3)	0.15 (0.11 to 0.19)	17.1% (-9.9 to 51.5)	0.89 (0.60 to 1.26)	9.3% (-0.6 to 19.7)	4.63 (2.37 to 6.37)	-0.1% (-31.4 to 37.0)
Latvia	13.92 (12.51 to 15.33)	17.8% (11.5 to 24.2)	0.10 (0.07 to 0.17)	-8.5% (-37.1 to 39.8)	0.80 (0.54 to 1.15)	17.5% (4.3 to 32.8)	3.28 (1.99 to 4.26)	-25.5% (-46.3 to 25.5)
Lithuania	14.03 (12.71 to 15.49)	6.4% (1.6 to 10.8)	0.13 (0.10 to 0.19)	17.6% (-12.3 to 48.3)	0.86 (0.59 to 1.23)	7.7% (-2.6 to 19.1)	4.34 (2.80 to 5.56)	9.8% (-14.1 to 34.2)
Moldova	15.44 (14.00 to 16.96)	17.4% (11.6 to 23.1)	0.12 (0.10 to 0.18)	-25.4% (-44.9 to 5.9)	1.01 (0.68 to 1.41)	18.3% (7.9 to 30.0)	4.35 (2.94 to 5.35)	-25.7% (-51.6 to 12.3)
Russian Federation	19.81 (17.89 to 21.88)	-13.5% (-15.7 to -11.4)	0.68 (0.53 to 1.02)	13.2% (-9.3 to 89.0)	1.22 (0.83 to 1.73)	-11.1% (-14.1 to -8.3)	14.63 (11.95 to 18.71)	28.9% (-1.8 to 73.0)
Ukraine	15.76 (14.08 to 17.59)	0.2% (-3.2 to 4.0)	0.77 (0.59 to 1.04)	107.0% (60.9 to 180.4)	1.02 (0.69 to 1.46)	1.6% (-6.7 to 10.8)	15.97 (12.69 to 20.67)	104.0% (57.4 to 170.8)
North Africa and Middle East	18.73 (16.93 to 20.57)	7.8% (6.5 to 9.2)	0.34 (0.29 to 0.39)	-27.8% (-40.1 to -13.3)	1.31 (0.89 to 1.84)	9.5% (6.3 to 12.5)	14.55 (12.29 to 17.11)	-39.9% (-52.1 to -23.4)
Afghanistan	14.13 (12.76 to 15.58)	-0.5% (-3.2 to 2.5)	0.35 (0.23 to 0.54)	-6.4% (-34.8 to 94.0)	0.86 (0.57 to 1.21)	-0.7% (-9.7 to 9.9)	13.09 (8.72 to 20.89)	-20.0% (-46.8 to 96.3)
Algeria	18.96 (17.07 to 20.79)	5.0% (1.6 to 8.5)	0.33 (0.21 to 0.52)	-21.2% (-39.3 to 0.8)	1.33 (0.90 to 1.88)	5.4% (-2.7 to 13.9)	12.39 (8.11 to 17.78)	-35.8% (-54.6 to -12.2)
Bahrain	15.93 (14.42 to 17.46)	-3.3% (-6.2 to 0.0)	0.10 (0.08 to 0.12)	-55.6% (-67.6 to -35.3)	1.05 (0.71 to 1.49)	-4.1% (-12.3 to 5.3)	3.14 (2.46 to 3.95)	-60.4% (-71.7 to -43.1)
Egypt	17.95 (16.25 to 19.77)	13.4% (9.5 to 17.6)	0.33 (0.22 to 0.50)	-38.2% (-60.8 to -5.7)	1.23 (0.84 to 1.75)	16.0% (7.5 to 25.9)	12.81 (8.79 to 18.88)	-56.8% (-74.0 to -31.7)
Iran	18.59 (16.67 to 20.66)	3.8% (1.6 to 5.8)	0.33 (0.28 to 0.41)	-11.4% (-25.1 to 8.4)	1.31 (0.88 to 1.84)	4.5% (1.4 to 7.6)	12.21 (10.47 to 15.25)	-32.5% (-44.8 to -11.6)
Iraq	28.82 (25.83 to 31.98)	7.5% (3.5 to 11.6)	0.88 (0.73 to 1.02)	-47.4% (-61.9 to -25.6)	2.18 (1.49 to 3.04)	8.3% (-2.6 to 19.5)	42.81 (34.11 to 53.02)	-49.6% (-66.1 to -23.7)
Jordan	18.71 (16.67 to 20.79)	1.9% (-1.9 to 5.9)	0.18 (0.15 to 0.22)	-48.7% (-60.3 to -32.7)	1.33 (0.90 to 1.91)	2.0% (-4.9 to 8.6)	6.12 (5.12 to 7.28)	-61.2% (-70.6 to -48.2)
Kuwait	23.83 (21.14 to 26.71)	1.5% (-2.3 to 5.7)	0.24 (0.20 to 0.28)	-38.3% (-50.2 to -24.5)	1.79 (1.18 to 2.50)	1.8% (-10.6 to 16.1)	10.46 (8.75 to 12.33)	-46.4% (-57.2 to -35.0)
Lebanon	17.86 (16.08 to 19.68)	0.2% (-3.3 to 3.5)	0.18 (0.12 to 0.27)	-37.3% (-58.5 to -14.1)	1.24 (0.84 to 1.75)	-0.2% (-7.3 to 7.9)	5.85 (4.10 to 8.62)	-50.0% (-66.0 to -30.4)
Libya	18.03 (16.22 to 19.96)	12.7% (8.7 to 16.7)	0.37 (0.23 to 0.55)	-53.5% (-71.3 to -30.0)	1.27 (0.86 to 1.79)	14.5% (6.3 to 23.0)	20.02 (11.89 to 32.14)	-63.5% (-79.8 to -37.9)
Morocco	16.49 (14.94 to 18.12)	8.7% (5.2 to 12.6)	0.28 (0.21 to 0.38)	-18.5% (-42.3 to 10.7)	1.10 (0.74 to 1.57)	10.2% (1.0 to 19.9)	10.84 (8.08 to 15.11)	-34.6% (-56.5 to -4.8)
Palestine	17.88 (16.16 to 19.76)	6.0% (2.7 to 9.8)	0.33 (0.22 to 0.42)	0.0% (-41.6 to 75.2)	1.25 (0.84 to 1.74)	6.9% (-0.5 to 15.3)	10.73 (8.01 to 13.28)	-30.1% (-57.9 to 28.1)
Oman	24.70 (22.11 to 27.31)	20.2% (14.7 to 25.3)	0.45 (0.35 to 0.58)	-38.8% (-63.3 to -0.7)	1.85 (1.24 to 2.56)	21.9% (13.7 to 31.0)	22.59 (16.80 to 29.35)	-52.8% (-72.8 to -18.5)
Qatar	19.06 (17.30 to 20.84)	-1.8% (-5.5 to 1.8)	0.17 (0.12 to 0.23)	-45.9% (-69.0 to -6.6)	1.34 (0.90 to 1.89)	-1.7% (-9.4 to 5.9)	6.26 (4.16 to 9.84)	-49.1% (-76.0 to 7.9)
Saudi Arabia	32.28 (29.10 to 35.72)	5.8% (1.3 to 10.3)	0.59 (0.45 to 0.79)	-14.1% (-43.1 to 41.1)	2.48 (1.68 to 3.45)	5.9% (-4.9 to 17.3)	17.43 (13.79 to 22.07)	-41.1% (-62.2 to -0.9)
Sudan	15.01 (13.60 to 16.53)	4.2% (1.0 to 7.5)	0.36 (0.26 to 0.48)	-21.0% (-48.5 to 15.8)	0.97 (0.66 to 1.38)	5.4% (-3.2 to 15.3)	15.82 (11.13 to 21.60)	-35.4% (-60.5 to 7.5)
Syria	19.93 (17.90 to 22.10)	10.8% (7.2 to 14.7)	0.33 (0.25 to 0.43)	-34.7% (-53.8 to -7.0)	1.42 (0.96 to 1.99)	12.0% (4.1 to 20.3)	12.54 (8.99 to 16.74)	-52.4% (-69.3 to -24.4)
Tunisia	18.48 (16.59 to 20.45)	8.1% (4.4 to 11.9)	0.25 (0.16 to 0.36)	-30.7% (-52.6 to -1.2)	1.30 (0.88 to 1.85)	9.3% (1.8 to 17.5)	7.51 (4.61 to 10.94)	-53.7% (-71.2 to -28.8)
Turkey	14.58 (13.19 to 15.92)	-0.9% (-4.4 to 2.8)	0.14 (0.11 to 0.17)	-29.0% (-46.5 to -5.6)	0.96 (0.65 to 1.40)	-0.5% (-8.8 to 8.4)	6.28 (4.85 to 8.45)	-32.6% (-53.3 to 1.7)
United Arab Emirates	20.40 (18.32 to 22.52)	-6.4% (-10.0 to -2.7)	0.37 (0.23 to 0.65)	3.4% (-39.5 to 55.4)	1.45 (0.98 to 2.02)	-6.8% (-14.3 to 1.9)	13.29 (8.90 to 20.73)	-6.7% (-43.7 to 43.5)
Yemen	15.86 (14.35 to 17.49)	7.6% (4.1 to 11.2)	0.40 (0.26 to 0.58)	-23.4% (-54.3 to 34.6)	1.05 (0.70 to 1.49)	9.3% (0.4 to 19.0)	16.58 (10.76 to 25.51)	-42.2% (-68.2 to 20.6)
Central Asia	23.55 (21.13 to 25.95)	1.3% (-0.7 to 3.4)	1.01 (0.85 to 1.14)	80.3% (53.4 to 106.6)	1.78 (1.20 to 2.49)	2.4% (-2.6 to 7.7)	32.92 (29.79 to 38.87)	60.5% (41.4 to 89.4)
Armenia	15.13 (13.61 to 16.79)	-5.1% (-8.6 to -1.7)	0.15 (0.11 to 0.30)	-16.2% (-56.4 to 226.3)	1.02 (0.69 to 1.45)	-4.2% (-11.6 to 3.6)	3.89 (2.77 to 7.04)	-16.4% (-51.6 to 111.7)
Azerbaijan	26.95 (23.96 to 29.98)	-4.6% (-8.8 to -0.4)	1.38 (1.06 to 1.88)	-5.5% (-31.3 to 34.3)	2.08 (1.40 to 2.87)	-3.8% (-14.7 to 8.8)	40.33 (30.67 to 58.77)	-16.2% (-37.4 to 13.1)
Georgia	29.87 (26.43 to 33.36)	-1.6% (-6.3 to 3.5)	1.25 (0.44 to 1.77)	32.1% (-50.9 to 81.1)	2.36 (1.57 to 3.35)	-1.8% (-15.0 to 14.3)	44.18 (16.47 to 62.42)	17.1% (-52.6 to 58.1)
Kazakhstan	22.05 (19.88 to 24.17)	0.1% (-3.5 to 3.9)	1.67 (1.11 to 2.03)	1096.7% (362.8 to 1611.4)	1.58 (1.09 to 2.19)	1.0% (-8.1 to 10.6)	47.43 (37.69 to 65.78)	901.1% (279.9 to 1336.8)
Kyrgyzstan	23.69 (21.43 to 26.21)	10.0% (5.9 to 14.1)	0.34 (0.21 to 0.44)	-17.5% (-34.6 to 7.3)	1.61 (1.08 to 2.28)	10.8% (-3.0 to 26.4)	14.07 (8.65 to 18.46)	-17.2% (-35.4 to 11.4)
Mongolia	33.34 (29.47 to 37.24)	11.6% (6.5 to 17.3)	1.97 (1.21 to 2.58)	-12.2% (-31.0 to 13.0)	2.60 (1.74 to 3.59)	13.3% (-0.8 to 29.4)	54.92 (38.05 to 69.50)	-17.5% (-34.6 to 3.1)
Tajikistan	13.83 (12.47 to 15.33)	-3.5% (-7.0 to 0.1)	0.23 (0.17 to 0.29)	9.9% (-11.7 to 37.7)	0.92 (0.61 to 1.30)	-3.4% (-11.1 to 4.6)	10.03 (6.41 to 12.93)	8.9% (-13.0 to 37.8)
Turkmenistan	19.04 (17.17 to 20.96)	-1.3% (-4.5 to 2.3)	0.83 (0.61 to 1.22)	-17.1% (-39.3 to 14.6)	1.38 (0.94 to 1.93)	-0.5% (-8.6 to 9.2)	32.18 (23.81 to 49.87)	-15.1% (-36.1 to 16.6)
Uzbekistan	24.84 (21.78 to 27.92)	5.6% (0.9 to 10.4)	0.76 (0.63 to 1.08)	82.8% (38.6 to 158.8)	1.97 (1.32 to 2.77)	6.3% (-3.1 to 17.0)	29.49 (24.44 to 39.86)	71.4% (32.7 to 144.3)
South Asia	18.36 (16.61 to 20.24)	2.2% (1.2 to 3.2)	0.55 (0.35 to 0.81)	2.7% (-18.6 to 29.9)	1.30 (0.89 to 1.82)	3.8% (1.9 to 5.8)	16.00 (10.40 to 23.34)	-10.8% (-30.8 to 15.9)
Bangladesh	20.95 (18.86 to 23.13)	5.4% (1.7 to 9.7)	0.44 (0.26 to 0.69)	-27.0% (-44.6 to -2.0)	1.56 (1.06 to 2.18)	7.4% (0.5 to 15.1)	14.08 (8.09 to 22.09)	-37.8% (-53.6 to -9.3)
Bhutan	20.54 (18.49 to 22.66)	5.9% (2.0 to 9.9)	0.50 (0.30 to 0.86)	-25.5% (-50.0 to 8.8)	1.52 (1.02 to 2.11)	7.5% (0.1 to 15.5)	13.92 (8.45 to 23.72)	-39.0% (-61.7 to -7.6)
India	17.85 (16.15 to 19.72)	2.2% (1.2 to 3.2)	0.54 (0.33 to 0.79)	7.1% (-16.5 to 39.2)	1.25 (0.85 to 1.76)	3.8% (1.9 to 5.6)	15.37 (9.78 to 22.44)	-9.6% (-30.8 to 22.3)

Nepal	17.38 (15.65 to 19.15)	5.2% (1.3 to 9.0)	0.30 (0.17 to 0.44)	-5.7% (-39.8 to 35.9)	1.23 (0.84 to 1.73)	6.9% (-0.8 to 14.3)	8.16 (4.80 to 12.19)	-24.4% (-54.3 to 17.2)
Pakistan	20.82 (18.76 to 22.84)	3.6% (-0.1 to 7.3)	0.84 (0.48 to 1.47)	15.3% (-18.0 to 57.7)	1.52 (1.03 to 2.13)	4.4% (-3.0 to 12.6)	23.93 (14.30 to 41.19)	6.0% (-23.4 to 46.0)
Southeast Asia	23.38 (21.09 to 25.72)	7.7% (6.5 to 9.0)	0.47 (0.37 to 0.60)	-9.1% (-23.9 to 9.4)	1.64 (1.11 to 2.28)	8.7% (5.9 to 11.8)	15.78 (13.46 to 19.08)	-22.1% (-39.0 to -0.4)
Cambodia	19.64 (17.78 to 21.66)	3.9% (0.6 to 7.7)	0.56 (0.39 to 0.78)	1.0% (-33.0 to 45.0)	1.32 (0.90 to 1.87)	4.7% (-3.4 to 13.3)	18.07 (12.98 to 24.36)	-19.8% (-54.4 to 25.2)
Indonesia	24.25 (21.78 to 26.94)	9.9% (8.5 to 11.4)	0.49 (0.32 to 0.79)	9.5% (-11.7 to 35.0)	1.69 (1.15 to 2.38)	11.4% (8.5 to 14.4)	14.63 (9.90 to 22.39)	-19.9% (-36.1 to 4.4)
Laos	19.69 (17.76 to 21.70)	4.9% (1.6 to 8.2)	0.79 (0.53 to 1.21)	-16.2% (-44.3 to 19.6)	1.32 (0.89 to 1.88)	6.4% (-2.5 to 15.1)	32.39 (22.49 to 45.27)	-37.2% (-59.4 to 2.8)
Malaysia	22.27 (20.00 to 24.78)	6.1% (2.5 to 9.8)	0.17 (0.13 to 0.22)	-48.7% (-65.7 to -8.6)	1.54 (1.05 to 2.17)	6.6% (-1.4 to 15.6)	7.52 (6.03 to 9.65)	-53.3% (-68.0 to -21.0)
Maldives	21.03 (19.00 to 23.27)	12.9% (8.9 to 17.2)	0.38 (0.30 to 0.46)	-42.8% (-60.0 to -20.7)	1.45 (0.98 to 2.06)	15.4% (5.5 to 24.9)	9.13 (7.58 to 10.83)	-56.7% (-70.6 to -36.8)
Mauritius	17.59 (15.81 to 19.51)	-0.4% (-3.8 to 3.1)	0.18 (0.14 to 0.28)	-6.5% (-34.4 to 71.7)	1.12 (0.76 to 1.57)	-0.8% (-9.2 to 8.5)	6.96 (5.44 to 10.48)	-9.4% (-36.1 to 69.8)
Myanmar	18.40 (16.64 to 20.28)	4.7% (1.4 to 7.9)	0.47 (0.33 to 0.68)	-8.9% (-45.6 to 47.7)	1.19 (0.80 to 1.69)	6.4% (-2.4 to 15.3)	18.60 (12.61 to 25.79)	-26.4% (-59.9 to 33.6)
Philippines	18.69 (16.81 to 20.69)	2.0% (-1.3 to 5.6)	0.77 (0.54 to 1.00)	52.8% (10.4 to 124.3)	1.23 (0.84 to 1.75)	2.7% (-5.6 to 11.3)	22.48 (18.07 to 30.76)	18.7% (-8.7 to 71.0)
Sri Lanka	33.50 (30.11 to 36.95)	13.1% (8.9 to 18.0)	0.85 (0.61 to 1.09)	-33.3% (-60.1 to -3.9)	2.53 (1.71 to 3.51)	14.2% (3.6 to 26.6)	29.27 (20.59 to 37.29)	-33.9% (-60.4 to -6.4)
Seychelles	22.78 (20.43 to 25.38)	-5.8% (-9.3 to -2.1)	0.73 (0.61 to 0.90)	-13.9% (-40.3 to 22.4)	1.53 (1.03 to 2.15)	-6.1% (-14.3 to 2.0)	21.54 (17.99 to 25.63)	-11.3% (-36.4 to 20.7)
Thailand	29.35 (26.16 to 32.77)	7.7% (3.3 to 12.4)	0.25 (0.21 to 0.31)	-54.6% (-67.2 to -15.7)	2.21 (1.51 to 3.09)	7.8% (-1.7 to 18.1)	9.28 (7.52 to 12.52)	-52.2% (-65.9 to -10.9)
Timor-Leste	21.50 (19.51 to 23.62)	10.2% (6.8 to 14.1)	0.63 (0.33 to 1.13)	-8.3% (-41.2 to 42.4)	1.47 (1.01 to 2.08)	12.4% (3.9 to 20.8)	21.46 (11.37 to 35.60)	-38.3% (-64.3 to 9.1)
Vietnam	21.23 (19.16 to 23.34)	7.9% (4.2 to 11.7)	0.42 (0.29 to 0.53)	-5.1% (-33.6 to 40.0)	1.45 (0.97 to 2.04)	8.7% (0.8 to 17.6)	12.96 (8.72 to 17.10)	-19.6% (-47.4 to 19.6)
East Asia	33.58 (29.80 to 37.53)	9.4% (7.8 to 10.9)	1.00 (0.84 to 1.09)	24.9% (9.3 to 41.3)	2.57 (1.74 to 3.57)	12.0% (8.9 to 15.3)	26.65 (22.66 to 28.70)	-15.7% (-26.6 to -1.6)
China	33.71 (29.88 to 37.73)	10.0% (8.4 to 11.7)	1.02 (0.86 to 1.11)	25.1% (9.8 to 42.4)	2.58 (1.75 to 3.59)	12.8% (9.5 to 16.5)	26.74 (22.78 to 28.79)	-16.7% (-28.0 to -2.1)
North Korea	26.75 (23.76 to 29.85)	-2.1% (-5.9 to 1.6)	0.89 (0.64 to 1.16)	35.8% (-2.3 to 80.2)	2.03 (1.38 to 2.82)	-2.8% (-9.1 to 4.1)	31.49 (22.39 to 41.20)	38.5% (2.2 to 82.1)
Taiwan (Province of China)	33.79 (31.47 to 36.32)	-8.4% (-15.8 to 0.3)	0.44 (0.25 to 0.54)	91.1% (-6.8 to 172.6)	2.64 (1.79 to 3.68)	-9.2% (-23.3 to 8.7)	16.14 (9.95 to 19.74)	71.3% (-7.3 to 133.4)
Oceania	25.40 (22.87 to 28.07)	1.2% (-1.1 to 3.6)	2.61 (1.98 to 3.39)	-10.0% (-23.8 to 5.4)	1.79 (1.23 to 2.47)	0.7% (-4.8 to 6.6)	76.98 (58.02 to 104.53)	-8.5% (-24.5 to 10.4)
American Samoa	20.94 (19.03 to 22.98)	2.7% (-0.6 to 6.4)	0.69 (0.54 to 0.84)	-6.1% (-31.5 to 29.8)	1.38 (0.93 to 1.95)	3.1% (-7.1 to 15.3)	17.30 (13.85 to 20.51)	-4.2% (-26.6 to 25.5)
Federated States of Micronesia	31.82 (28.33 to 35.59)	10.3% (5.4 to 15.5)	2.54 (1.76 to 3.59)	-10.1% (-31.7 to 17.2)	2.31 (1.58 to 3.27)	10.2% (-1.7 to 22.9)	66.30 (44.60 to 94.67)	-14.9% (-37.4 to 13.7)
Fiji	40.77 (35.68 to 46.31)	2.6% (-2.5 to 7.7)	3.85 (3.14 to 4.81)	-23.0% (-43.2 to 5.1)	3.08 (2.12 to 4.26)	2.3% (-8.7 to 15.1)	100.05 (81.95 to 127.19)	-21.5% (-41.3 to 4.7)
Guam	28.71 (25.77 to 31.85)	1.8% (-2.5 to 5.8)	1.07 (0.90 to 1.26)	-13.1% (-33.9 to 19.2)	2.07 (1.39 to 2.86)	1.6% (-9.5 to 14.1)	30.18 (25.11 to 35.27)	-3.3% (-26.5 to 29.9)
Kiribati	34.86 (30.65 to 39.37)	0.5% (-4.1 to 5.6)	3.97 (2.97 to 5.84)	-4.3% (-25.2 to 19.8)	2.58 (1.76 to 3.59)	-0.5% (-11.1 to 11.2)	120.45 (89.89 to 177.61)	-6.1% (-26.3 to 18.3)
Marshall Islands	30.28 (26.98 to 33.77)	6.0% (1.2 to 11.2)	3.40 (2.38 to 4.41)	16.1% (-7.3 to 47.3)	2.20 (1.50 to 3.02)	6.4% (-6.1 to 21.3)	94.16 (65.95 to 124.08)	19.6% (-5.0 to 52.7)
Northern Mariana Islands	34.32 (30.60 to 38.18)	3.1% (-1.4 to 7.9)	0.81 (0.67 to 0.97)	-24.8% (-44.1 to 1.6)	2.53 (1.70 to 3.56)	2.6% (-9.8 to 17.8)	18.98 (15.77 to 22.39)	-26.0% (-43.4 to -2.4)
Papua New Guinea	21.86 (19.73 to 24.23)	5.9% (2.2 to 9.8)	2.54 (1.69 to 3.72)	-2.5% (-22.4 to 24.3)	1.50 (1.01 to 2.10)	6.5% (-3.0 to 16.9)	78.58 (54.28 to 115.34)	-6.6% (-27.9 to 20.4)
Samoa	33.86 (29.88 to 37.84)	6.8% (1.8 to 11.4)	2.02 (1.35 to 2.76)	3.1% (-23.3 to 39.8)	2.50 (1.70 to 3.45)	6.4% (-5.2 to 20.3)	47.68 (31.71 to 67.67)	-7.2% (-29.3 to 21.1)
Solomon Islands	23.99 (21.60 to 26.52)	4.1% (0.1 to 8.2)	1.65 (1.07 to 2.38)	6.8% (-17.4 to 43.9)	1.67 (1.12 to 2.37)	4.7% (-5.9 to 16.4)	41.63 (27.01 to 61.48)	1.2% (-21.9 to 35.8)
Tonga	20.02 (18.18 to 21.98)	3.9% (0.7 to 7.4)	0.46 (0.34 to 0.61)	-11.6% (-35.6 to 20.2)	1.35 (0.91 to 1.91)	4.3% (-5.4 to 14.3)	11.76 (8.79 to 15.74)	-3.5% (-27.5 to 26.0)
Vanuatu	28.51 (25.51 to 31.90)	2.1% (-2.2 to 6.6)	2.77 (1.36 to 5.50)	21.0% (-12.8 to 71.2)	2.04 (1.40 to 2.87)	1.9% (-7.9 to 13.7)	76.15 (37.80 to 152.43)	16.6% (-18.2 to 67.3)
High-income Asia Pacific	45.57 (41.11 to 50.11)	-15.8% (-18.7 to -12.8)	0.39 (0.32 to 0.56)	-61.3% (-70.3 to -31.4)	3.61 (2.48 to 5.01)	-16.7% (-20.6 to -12.5)	10.93 (9.37 to 14.41)	-56.1% (-64.7 to -31.5)
Brunei	31.38 (28.21 to 34.62)	-17.8% (-21.1 to -14.3)	0.93 (0.78 to 1.11)	-19.4% (-37.4 to 6.0)	2.39 (1.60 to 3.35)	-19.0% (-30.4 to -6.1)	34.37 (27.39 to 41.19)	-24.0% (-41.0 to 5.7)
Japan	51.43 (46.27 to 56.70)	-12.9% (-16.1 to -9.3)	0.41 (0.33 to 0.60)	-60.5% (-70.3 to -29.7)	4.10 (2.81 to 5.64)	-13.6% (-17.5 to -8.9)	11.51 (9.39 to 16.03)	-54.2% (-63.8 to -29.7)
Singapore	66.96 (58.61 to 75.80)	-11.6% (-16.3 to -7.1)	0.72 (0.57 to 1.01)	-65.0% (-75.6 to -35.7)	5.56 (3.67 to 7.77)	-11.9% (-24.3 to 2.4)	30.43 (25.88 to 36.34)	-68.0% (-76.6 to -42.4)
South Korea	29.66 (26.44 to 32.79)	-15.0% (-18.9 to -10.9)	0.29 (0.24 to 0.37)	-57.0% (-69.0 to -27.5)	2.28 (1.54 to 3.17)	-16.6% (-28.1 to -2.8)	7.03 (5.66 to 8.22)	-58.6% (-67.6 to -33.5)
High-income North America	14.89 (13.60 to 16.37)	-19.8% (-24.1 to -14.8)	0.29 (0.20 to 0.33)	64.2% (-8.0 to 130.1)	1.20 (0.81 to 1.66)	-21.1% (-26.2 to -15.4)	14.26 (10.34 to 16.20)	46.6% (-13.3 to 98.9)
Canada	21.47 (18.92 to 24.06)	-11.9% (-16.3 to -7.3)	0.19 (0.11 to 0.25)	70.2% (-26.0 to 183.6)	1.77 (1.16 to 2.53)	-12.1% (-24.7 to 3.5)	9.24 (5.91 to 11.52)	53.0% (-31.5 to 163.8)
Greenland	18.98 (16.79 to 21.42)	-11.0% (-15.0 to -6.6)	0.63 (0.39 to 0.81)	-28.0% (-57.3 to 0.9)	1.54 (1.02 to 2.17)	-11.1% (-21.5 to -0.3)	29.38 (19.96 to 38.44)	-41.5% (-63.2 to -15.5)
United States	14.14 (12.91 to 15.53)	-21.1% (-26.1 to -15.5)	0.30 (0.21 to 0.35)	64.2% (-6.6 to 128.8)	1.13 (0.77 to 1.56)	-22.6% (-28.1 to -16.4)	14.79 (10.80 to 16.77)	46.4% (-12.0 to 97.4)
Western Europe	25.28 (23.07 to 27.74)	-20.6% (-22.4 to -18.8)	0.56 (0.37 to 0.66)	5.3% (-44.3 to 42.2)	1.90 (1.31 to 2.62)	-22.3% (-25.7 to -19.0)	10.11 (7.32 to 11.25)	-12.0% (-45.7 to 8.2)
Andorra	27.71 (25.11 to 30.74)	-22.7% (-25.9 to -19.4)	0.55 (0.39 to 0.72)	-7.1% (-36.8 to 41.7)	2.11 (1.45 to 2.95)	-24.7% (-32.9 to -15.1)	11.81 (8.48 to 15.60)	-21.6% (-46.5 to 12.1)
Austria	36.72 (33.46 to 40.40)	-13.9% (-18.4 to -9.2)	0.65 (0.48 to 1.17)	-59.3% (-80.2 to 0.7)	2.82 (1.91 to 3.92)	-15.2% (-25.2 to -4.0)	10.90 (8.89 to 15.12)	-63.0% (-76.1 to -25.3)
Belgium	24.17 (21.86 to 26.54)	-15.9% (-19.9 to -11.4)	0.58 (0.37 to 0.73)	36.9% (-40.8 to 144.4)	1.85 (1.27 to 2.58)	-17.1% (-24.5 to -8.5)	8.77 (6.28 to 10.27)	0.3% (-49.8 to 59.9)
Cyprus	30.98 (27.89 to 34.25)	-19.2% (-23.0 to -15.0)	0.80 (0.50 to 1.04)	-42.1% (-55.8 to -23.8)	2.38 (1.61 to 3.29)	-20.4% (-28.4 to -11.7)	12.89 (10.30 to 15.18)	-46.1% (-57.2 to -33.1)
Denmark	28.85 (25.74 to 31.94)	-21.9% (-25.1 to -18.4)	0.58 (0.46 to 0.67)	-31.8% (-45.0 to -10.4)	2.26 (1.52 to 3.22)	-23.4% (-32.9 to -12.5)	10.06 (8.68 to 11.64)	-44.1% (-54.1 to -23.3)
Finland	45.78 (40.99 to 50.88)	-21.0% (-24.9 to -16.3)	0.66 (0.54 to 0.91)	-44.5% (-60.4 to -10.6)	3.63 (2.45 to 5.10)	-22.3% (-32.7 to -9.5)	19.48 (16.19 to 22.86)	-47.6% (-60.0 to -21.6)
France	17.64 (16.02 to 19.34)	-17.9% (-20.9 to -15.0)	0.25 (0.15 to 0.32)	88.7% (-46.2 to 339.3)	1.24 (0.84 to 1.72)	-20.7% (-26.2 to -15.1)	5.65 (2.81 to 7.51)	52.4% (-48.9 to 159.9)
Germany	29.43 (26.36 to 32.75)	-27.3% (-30.4 to -24.0)	0.67 (0.53 to 1.03)	-32.8% (-53.1 to -2.3)	2.26 (1.54 to 3.15)	-28.8% (-37.8 to -19.2)	13.56 (11.32 to 17.86)	-29.7% (-41.0 to -12.0)

Greece	19.73 (17.91 to 21.73)	-16.0% (-19.1 to -12.6)	0.38 (0.26 to 0.49)	-56.9% (-70.3 to 8.7)	1.43 (0.97 to 1.98)	-18.4% (-25.0 to -11.5)	6.14 (4.84 to 7.15)	-62.6% (-74.0 to -14.6)
Iceland	31.26 (27.94 to 34.74)	-20.4% (-23.8 to -16.5)	0.45 (0.35 to 0.53)	10.3% (-43.8 to 69.4)	2.47 (1.68 to 3.50)	-21.4% (-31.9 to -10.0)	7.74 (6.71 to 9.52)	-13.3% (-46.4 to 22.0)
Ireland	26.89 (24.24 to 29.80)	-19.1% (-22.4 to -16.0)	0.93 (0.38 to 1.33)	107.1% (-66.9 to 416.9)	2.05 (1.37 to 2.92)	-20.8% (-31.9 to -8.7)	14.02 (5.92 to 19.55)	44.2% (-62.0 to 161.1)
Israel	25.60 (22.99 to 28.34)	-22.0% (-25.3 to -18.5)	0.27 (0.21 to 0.38)	-27.9% (-41.3 to -10.4)	1.97 (1.35 to 2.76)	-23.8% (-33.1 to -13.8)	7.06 (5.57 to 8.61)	-40.2% (-55.2 to -21.5)
Italy	24.53 (22.42 to 26.79)	-20.1% (-24.4 to -15.5)	0.75 (0.20 to 1.15)	173.3% (-78.9 to 1213.8)	1.83 (1.26 to 2.52)	-21.9% (-29.1 to -14.8)	8.03 (2.99 to 11.02)	70.1% (-74.6 to 352.4)
Luxembourg	40.99 (36.73 to 45.53)	-21.0% (-25.2 to -16.3)	1.13 (0.80 to 1.42)	24.2% (-45.9 to 109.2)	3.24 (2.18 to 4.47)	-21.7% (-31.5 to -9.9)	14.66 (10.64 to 17.61)	-14.9% (-58.1 to 27.5)
Malta	57.39 (51.38 to 63.84)	-25.4% (-29.2 to -21.0)	1.65 (1.23 to 1.99)	-0.7% (-65.8 to 69.5)	4.65 (3.20 to 6.51)	-25.9% (-34.9 to -15.0)	29.80 (22.98 to 34.56)	-6.2% (-54.8 to 41.3)
Netherlands	27.82 (25.00 to 30.81)	-17.3% (-21.0 to -13.4)	0.76 (0.55 to 0.91)	-23.0% (-53.1 to 8.0)	2.11 (1.44 to 2.94)	-19.1% (-28.1 to -9.5)	10.85 (8.78 to 13.48)	-42.7% (-53.9 to -19.1)
Norway	34.15 (30.84 to 37.60)	-16.8% (-21.0 to -12.5)	0.61 (0.51 to 0.67)	-10.4% (-16.5 to -2.6)	2.60 (1.79 to 3.58)	-17.9% (-23.2 to -12.5)	10.21 (9.01 to 11.79)	-30.6% (-38.8 to -23.3)
Portugal	20.31 (18.44 to 22.35)	-21.4% (-25.1 to -17.6)	0.40 (0.28 to 0.49)	38.7% (-52.9 to 174.0)	1.48 (1.00 to 2.08)	-23.4% (-31.2 to -15.0)	6.03 (4.68 to 7.11)	-12.5% (-62.1 to 41.8)
Spain	17.76 (16.11 to 19.49)	-17.8% (-20.8 to -14.9)	0.44 (0.21 to 0.62)	113.6% (-52.3 to 418.7)	1.25 (0.86 to 1.75)	-21.3% (-26.9 to -15.2)	6.82 (3.77 to 8.59)	24.8% (-55.6 to 108.5)
Sweden	49.15 (43.96 to 55.02)	-19.9% (-22.8 to -16.9)	0.81 (0.56 to 0.97)	124.7% (-22.9 to 540.6)	3.88 (2.63 to 5.42)	-20.9% (-30.0 to -10.0)	19.16 (11.54 to 24.21)	83.3% (-41.0 to 371.2)
Switzerland	25.05 (22.78 to 27.79)	-21.2% (-25.3 to -17.0)	0.35 (0.28 to 0.49)	-34.8% (-52.3 to -1.5)	1.95 (1.33 to 2.71)	-22.5% (-32.0 to -11.6)	6.34 (5.32 to 8.21)	-51.4% (-63.6 to -15.4)
United Kingdom	25.90 (23.39 to 28.64)	-17.3% (-19.6 to -15.2)	0.61 (0.43 to 0.69)	46.0% (4.0 to 76.0)	1.97 (1.36 to 2.73)	-18.5% (-21.4 to -15.7)	13.53 (9.84 to 15.14)	17.7% (-18.3 to 40.7)
Australasia	28.11 (25.22 to 31.13)	-19.3% (-22.0 to -16.5)	0.31 (0.27 to 0.39)	-24.5% (-47.3 to -3.5)	2.20 (1.50 to 3.12)	-20.7% (-29.5 to -11.6)	12.72 (10.82 to 16.10)	-30.0% (-49.7 to -10.8)
Australia	26.50 (23.71 to 29.41)	-19.8% (-23.2 to -16.6)	0.30 (0.25 to 0.38)	-24.6% (-48.3 to -1.1)	2.08 (1.41 to 2.97)	-21.3% (-31.7 to -9.9)	11.86 (9.76 to 15.50)	-28.6% (-50.3 to -6.4)
New Zealand	36.81 (32.88 to 40.98)	-15.7% (-19.7 to -11.8)	0.42 (0.35 to 0.48)	-22.8% (-44.1 to -7.3)	2.87 (1.90 to 4.03)	-17.1% (-28.6 to -4.0)	17.44 (15.10 to 19.74)	-32.4% (-49.8 to -19.3)

Data in parentheses are 95% uncertainty intervals. YLDs= years lived with disability; YLLs= years of life lost.

Supplementary Table 6. Age-standardised prevalence, death, YLD, and YLL rates of alcoholic cardiomyopathy for both sexes in 2017, and their percentage changes from 1990 to 2017, by location.

	Prevalence		Deaths		YLDs		YLLs	
	2017 age-standardised rate per 100 000 people	Percentage change in age-standardised rates, 1990–2017	2017 age-standardised rate per 100 000 people	Percentage change in age-standardised rates, 1990–2017	2017 age-standardised rate per 100 000 people	Percentage change in age-standardised rates, 1990–2017	2017 age-standardised rate per 100 000 people	Percentage change in age-standardised rates, 1990–2017
Central Sub-Saharan Africa	19.79 (15.65 to 24.41)	3.4% (-3.1 to 10.2)	0.66 (0.40 to 0.97)	-21.6% (-42.2 to 11.0)	1.66 (1.10 to 2.39)	4.1% (-11.0 to 20.0)	18.41 (11.28 to 27.10)	-23.5% (-42.8 to 8.0)
Angola	22.61 (17.88 to 27.96)	5.4% (-3.6 to 15.1)	0.78 (0.48 to 1.19)	-21.7% (-52.2 to 37.4)	1.89 (1.20 to 2.78)	5.1% (-14.1 to 28.4)	21.54 (13.53 to 33.39)	-23.5% (-52.4 to 40.9)
Central African Republic	20.97 (16.34 to 26.13)	6.9% (-1.8 to 16.7)	0.90 (0.47 to 1.51)	-31.4% (-52.8 to 4.7)	1.75 (1.11 to 2.55)	6.8% (-15.4 to 32.1)	26.45 (13.59 to 44.28)	-31.4% (-53.4 to 4.7)
Congo	21.33 (16.56 to 26.52)	5.1% (-4.7 to 15.4)	0.84 (0.52 to 1.35)	-36.2% (-65.6 to 22.8)	1.79 (1.15 to 2.68)	6.0% (-13.6 to 30.5)	23.98 (14.56 to 38.83)	-39.6% (-67.4 to 12.8)
Democratic Republic of the Congo	18.49 (14.48 to 22.87)	1.4% (-7.6 to 11.4)	0.59 (0.31 to 0.99)	-16.9% (-41.9 to 21.8)	1.56 (1.01 to 2.27)	2.5% (-17.8 to 25.0)	16.35 (8.44 to 27.10)	-18.8% (-42.9 to 19.0)
Equatorial Guinea	25.76 (20.38 to 31.73)	15.3% (3.2 to 27.5)	0.76 (0.43 to 1.19)	-46.4% (-72.9 to 4.3)	2.16 (1.38 to 3.11)	15.9% (-6.1 to 42.9)	20.51 (11.28 to 33.87)	-51.9% (-76.3 to -5.8)
Gabon	24.57 (19.15 to 30.61)	1.1% (-7.4 to 10.1)	0.81 (0.57 to 1.14)	-43.0% (-67.1 to 18.2)	2.07 (1.33 to 3.01)	1.6% (-16.6 to 23.6)	22.69 (15.84 to 32.25)	-44.8% (-69.7 to 16.1)
Eastern Sub-Saharan Africa	22.03 (17.35 to 27.09)	5.3% (1.8 to 8.9)	0.55 (0.37 to 0.97)	-39.1% (-58.5 to -3.3)	1.86 (1.22 to 2.66)	5.9% (0.0 to 12.1)	15.74 (10.79 to 28.42)	-40.9% (-60.1 to -8.6)
Burundi	26.86 (20.54 to 34.07)	-2.2% (-10.6 to 7.3)	0.81 (0.41 to 1.41)	-64.5% (-76.4 to -44.3)	2.27 (1.45 to 3.36)	-2.1% (-18.8 to 18.4)	23.43 (11.62 to 42.25)	-66.9% (-78.0 to -47.6)
Comoros	14.62 (11.29 to 18.41)	1.7% (-7.1 to 11.5)	0.39 (0.18 to 0.77)	-50.9% (-73.0 to -6.0)	1.25 (0.79 to 1.86)	2.5% (-17.4 to 24.7)	10.85 (4.93 to 21.89)	-54.3% (-75.6 to -12.3)
Djibouti	15.20 (11.71 to 19.31)	12.7% (2.5 to 23.9)	0.50 (0.22 to 1.15)	-34.9% (-63.4 to 12.5)	1.30 (0.82 to 1.92)	12.3% (-9.1 to 40.3)	13.98 (5.67 to 33.53)	-37.4% (-65.6 to 9.9)
Eritrea	12.58 (9.68 to 16.00)	2.3% (-6.1 to 11.7)	0.63 (0.35 to 1.18)	-47.7% (-74.7 to 16.1)	1.07 (0.68 to 1.59)	3.1% (-17.2 to 29.8)	19.06 (9.85 to 36.83)	-48.0% (-76.4 to 15.9)
Ethiopia	36.29 (29.03 to 43.61)	4.6% (-1.9 to 11.2)	0.40 (0.19 to 0.84)	-52.1% (-77.6 to 12.2)	3.05 (1.98 to 4.33)	6.0% (-3.6 to 17.2)	10.57 (5.14 to 22.44)	-56.1% (-80.0 to -0.9)
Kenya	11.98 (9.10 to 15.27)	6.2% (3.8 to 8.4)	0.58 (0.37 to 0.86)	-13.5% (-37.1 to 10.7)	1.03 (0.67 to 1.51)	6.0% (1.7 to 10.4)	16.55 (10.52 to 24.84)	-13.0% (-36.9 to 11.7)
Madagascar	15.30 (11.65 to 19.44)	1.8% (-7.4 to 11.9)	0.93 (0.53 to 1.72)	-25.4% (-52.4 to 12.8)	1.31 (0.84 to 1.95)	2.3% (-16.9 to 27.4)	28.58 (15.74 to 54.83)	-29.5% (-55.7 to 7.0)
Malawi	16.15 (12.28 to 20.53)	12.9% (2.5 to 22.9)	0.39 (0.21 to 0.76)	-20.8% (-48.3 to 51.0)	1.38 (0.88 to 2.03)	13.1% (-9.7 to 40.7)	11.23 (6.07 to 22.79)	-18.4% (-48.5 to 79.6)
Mozambique	10.34 (7.81 to 13.38)	10.6% (0.8 to 21.6)	0.61 (0.26 to 1.09)	-3.2% (-42.3 to 92.6)	0.90 (0.55 to 1.37)	10.3% (-14.6 to 41.7)	18.45 (7.88 to 33.49)	0.0% (-41.3 to 103.6)
Rwanda	21.08 (16.30 to 26.64)	0.1% (-8.1 to 8.7)	0.54 (0.34 to 0.88)	-70.0% (-83.4 to -43.3)	1.79 (1.11 to 2.64)	0.7% (-19.9 to 25.4)	14.45 (9.04 to 24.21)	-72.8% (-85.6 to -47.5)
Somalia	15.79 (12.12 to 20.09)	7.0% (-2.3 to 16.8)	0.54 (0.23 to 1.09)	-39.6% (-65.3 to 16.9)	1.34 (0.84 to 1.97)	6.5% (-14.1 to 34.3)	15.41 (6.42 to 31.26)	-40.3% (-67.1 to 28.4)
South Sudan	15.64 (11.91 to 19.68)	4.5% (-6.2 to 15.5)	0.48 (0.21 to 1.00)	-40.3% (-63.3 to 8.3)	1.31 (0.83 to 1.97)	4.3% (-15.2 to 31.0)	13.26 (5.56 to 28.15)	-39.9% (-63.9 to 13.9)
Tanzania	19.94 (15.24 to 25.18)	12.0% (1.6 to 23.5)	0.59 (0.34 to 1.17)	-32.7% (-59.9 to 36.2)	1.69 (1.09 to 2.50)	12.2% (-10.0 to 41.6)	16.48 (9.45 to 33.71)	-33.1% (-61.4 to 38.2)
Uganda	25.59 (19.81 to 32.07)	-1.3% (-9.5 to 7.3)	0.59 (0.36 to 1.08)	-41.7% (-62.5 to 5.2)	2.15 (1.36 to 3.21)	-0.4% (-17.7 to 22.1)	16.96 (10.10 to 31.61)	-43.0% (-63.4 to 1.9)
Zambia	15.50 (11.84 to 19.69)	17.7% (7.7 to 29.2)	0.50 (0.30 to 0.83)	-29.4% (-55.1 to 15.5)	1.32 (0.83 to 1.97)	16.9% (-6.3 to 47.3)	14.27 (8.31 to 24.10)	-30.2% (-56.4 to 16.9)
Southern Sub-Saharan Africa	24.51 (19.58 to 30.10)	10.9% (6.6 to 15.3)	0.83 (0.72 to 0.95)	-17.0% (-30.2 to -1.3)	2.02 (1.32 to 2.90)	10.3% (3.4 to 17.7)	15.17 (12.99 to 17.36)	-26.1% (-37.5 to -11.5)
Botswana	21.16 (16.71 to 26.22)	7.2% (-3.3 to 18.6)	0.70 (0.46 to 1.09)	-36.9% (-61.2 to 2.2)	1.75 (1.11 to 2.60)	7.5% (-11.7 to 29.5)	11.53 (6.97 to 19.23)	-42.8% (-65.2 to -8.4)
Lesotho	18.52 (14.55 to 22.99)	3.9% (-6.5 to 15.5)	1.04 (0.60 to 1.69)	-21.7% (-48.0 to 25.7)	1.52 (0.97 to 2.22)	3.2% (-14.9 to 25.5)	18.72 (11.01 to 30.18)	-20.7% (-47.1 to 29.3)
Namibia	19.82 (15.63 to 24.45)	5.7% (-5.0 to 17.0)	0.72 (0.44 to 1.08)	-38.5% (-61.6 to 6.5)	1.63 (1.07 to 2.41)	6.0% (-13.1 to 28.1)	13.04 (7.56 to 21.06)	-36.0% (-62.6 to 25.4)
South Africa	27.17 (21.65 to 33.31)	9.0% (4.3 to 13.9)	0.84 (0.72 to 0.96)	-18.7% (-33.4 to -2.2)	2.24 (1.46 to 3.21)	8.5% (1.4 to 16.7)	14.97 (12.45 to 17.36)	-31.1% (-43.9 to -14.0)
Swaziland	19.16 (15.03 to 23.82)	3.2% (-5.9 to 13.4)	0.79 (0.48 to 1.22)	-50.7% (-68.4 to -20.8)	1.56 (1.00 to 2.29)	2.7% (-16.5 to 24.8)	14.77 (8.89 to 22.38)	-51.0% (-71.0 to -18.2)
Zimbabwe	8.97 (7.06 to 11.16)	-4.5% (-16.6 to 9.4)	0.69 (0.46 to 0.95)	9.7% (-24.8 to 63.8)	0.78 (0.49 to 1.15)	-4.4% (-23.5 to 18.3)	16.03 (10.08 to 22.46)	23.7% (-18.1 to 93.2)
Western Sub-Saharan Africa	23.19 (18.51 to 28.48)	2.3% (-4.5 to 9.1)	0.41 (0.27 to 0.62)	-59.2% (-71.0 to -37.0)	1.95 (1.28 to 2.84)	2.8% (-9.7 to 17.4)	11.09 (7.31 to 17.24)	-58.5% (-70.7 to -36.0)
Benin	15.25 (11.99 to 19.14)	6.2% (-3.3 to 16.1)	0.27 (0.13 to 0.52)	-62.1% (-76.2 to -41.7)	1.30 (0.84 to 1.93)	7.6% (-13.9 to 32.9)	7.09 (3.21 to 13.87)	-60.7% (-75.8 to -38.9)
Burkina Faso	21.89 (17.28 to 27.35)	5.5% (-4.0 to 16.8)	0.47 (0.25 to 0.84)	-55.3% (-70.6 to -31.6)	1.85 (1.16 to 2.70)	7.9% (-13.5 to 33.0)	11.91 (6.52 to 21.80)	-55.5% (-71.9 to -32.2)
Cameroon	20.45 (16.23 to 25.38)	2.0% (-14.1 to 20.3)	0.49 (0.33 to 0.70)	-59.0% (-73.7 to -33.3)	1.72 (1.07 to 2.55)	2.7% (-22.0 to 31.9)	13.83 (9.31 to 20.05)	-54.6% (-71.2 to -25.2)
Cape Verde	13.69 (10.88 to 17.08)	16.6% (5.9 to 27.8)	0.11 (0.08 to 0.15)	11.6% (-44.2 to 104.1)	1.19 (0.74 to 1.77)	17.3% (-7.7 to 46.2)	3.40 (2.51 to 4.58)	11.2% (-42.6 to 100.6)
Chad	19.96 (15.55 to 25.08)	8.8% (-1.8 to 19.0)	0.35 (0.16 to 0.64)	-53.0% (-69.5 to -26.4)	1.67 (1.04 to 2.43)	8.7% (-13.4 to 34.8)	9.31 (4.26 to 17.02)	-49.4% (-67.6 to -21.3)
Cote d'Ivoire	20.79 (16.41 to 25.74)	2.2% (-7.2 to 12.7)	0.43 (0.21 to 0.72)	-53.2% (-71.0 to -25.1)	1.75 (1.10 to 2.56)	3.2% (-16.6 to 27.4)	12.25 (6.05 to 20.50)	-50.7% (-69.3 to -19.5)
The Gambia	19.36 (15.34 to 24.21)	3.1% (-6.0 to 13.4)	0.36 (0.18 to 0.57)	-56.5% (-73.4 to -30.8)	1.64 (1.06 to 2.42)	3.8% (-16.6 to 28.2)	9.90 (4.63 to 16.60)	-53.9% (-72.5 to -25.4)
Ghana	18.84 (14.63 to 23.67)	3.0% (-10.0 to 18.6)	0.89 (0.61 to 1.40)	38.4% (-15.9 to 187.5)	1.60 (1.02 to 2.30)	2.9% (-19.0 to 30.0)	22.80 (15.57 to 35.76)	42.0% (-15.7 to 185.9)
Guinea	11.58 (8.92 to 14.96)	15.9% (2.2 to 31.7)	0.31 (0.14 to 0.54)	-56.5% (-72.1 to -32.2)	1.00 (0.61 to 1.50)	15.9% (-10.8 to 50.0)	8.41 (3.59 to 14.63)	-53.4% (-70.2 to -26.6)
Guinea-Bissau	19.78 (15.44 to 24.44)	11.5% (2.1 to 21.2)	0.43 (0.25 to 0.81)	-65.7% (-79.2 to -45.3)	1.67 (1.07 to 2.48)	11.8% (-8.4 to 36.5)	12.18 (6.94 to 23.48)	-63.5% (-78.9 to -41.1)
Liberia	24.84 (19.58 to 31.07)	9.5% (-0.3 to 20.9)	0.33 (0.14 to 0.63)	-69.1% (-83.0 to -47.9)	2.06 (1.35 to 3.03)	9.2% (-10.5 to 33.4)	8.85 (3.82 to 16.66)	-68.6% (-83.4 to -46.6)
Mali	16.87 (13.26 to 21.10)	6.0% (-3.2 to 15.9)	0.29 (0.10 to 0.57)	-65.9% (-85.6 to -46.3)	1.43 (0.89 to 2.12)	7.4% (-14.5 to 35.0)	7.30 (2.51 to 14.30)	-66.8% (-87.4 to -45.7)

Mauritania	16.59 (12.99 to 20.93)	-0.8% (-9.9 to 9.2)	0.26 (0.10 to 0.54)	-67.0% (-85.0 to -40.5)	1.41 (0.91 to 2.08)	-0.3% (-19.5 to 25.5)	6.71 (2.27 to 14.59)	-67.9% (-85.7 to -42.0)
Niger	16.21 (12.86 to 20.08)	3.4% (-5.1 to 13.3)	0.24 (0.08 to 0.52)	-64.5% (-80.4 to -46.7)	1.39 (0.87 to 2.04)	4.0% (-17.9 to 30.2)	6.08 (1.93 to 13.23)	-64.0% (-81.1 to -44.9)
Nigeria	27.84 (22.00 to 34.67)	2.2% (-9.3 to 13.9)	0.38 (0.18 to 0.81)	-68.1% (-82.6 to -37.0)	2.34 (1.50 to 3.51)	2.5% (-17.6 to 27.2)	10.30 (4.49 to 22.75)	-67.9% (-83.0 to -35.6)
Sao Tome and Principe	27.51 (21.64 to 34.43)	14.5% (5.1 to 25.2)	0.43 (0.29 to 0.63)	-37.9% (-59.2 to -2.9)	2.32 (1.46 to 3.44)	13.7% (-7.2 to 39.9)	12.30 (7.86 to 19.25)	-36.1% (-58.5 to 1.1)
Senegal	21.01 (16.83 to 25.95)	5.4% (-4.5 to 16.0)	0.24 (0.09 to 0.42)	-58.4% (-77.1 to -30.9)	1.76 (1.13 to 2.56)	5.1% (-14.4 to 30.4)	6.11 (2.20 to 11.43)	-57.5% (-77.1 to -28.7)
Sierra Leone	29.00 (22.78 to 36.24)	12.6% (3.6 to 23.8)	0.47 (0.30 to 0.71)	-51.6% (-69.2 to -15.2)	2.43 (1.56 to 3.58)	13.4% (-7.9 to 40.2)	13.29 (8.38 to 19.95)	-48.9% (-67.4 to -7.4)
Togo	17.28 (13.56 to 21.53)	5.7% (-3.9 to 14.8)	0.30 (0.18 to 0.45)	-63.6% (-76.6 to -43.5)	1.47 (0.92 to 2.17)	6.4% (-15.2 to 32.4)	8.06 (4.62 to 12.73)	-61.1% (-76.1 to -37.7)
Andean Latin America	3.46 (2.80 to 4.27)	23.2% (14.3 to 32.9)	0.08 (0.07 to 0.10)	-37.0% (-52.5 to -14.0)	0.32 (0.21 to 0.48)	22.6% (13.0 to 33.0)	1.96 (1.60 to 2.33)	-39.5% (-54.5 to -16.4)
Bolivia	2.33 (1.56 to 3.23)	90.9% (54.0 to 155.5)	0.16 (0.11 to 0.22)	-44.4% (-63.2 to -9.0)	0.21 (0.12 to 0.34)	88.1% (48.8 to 153.2)	3.50 (2.32 to 4.97)	-49.1% (-66.6 to -12.2)
Ecuador	2.36 (1.92 to 2.90)	15.6% (5.1 to 27.3)	0.11 (0.09 to 0.13)	55.8% (14.5 to 114.3)	0.22 (0.14 to 0.32)	15.4% (4.7 to 27.4)	2.45 (1.95 to 3.04)	48.1% (7.5 to 104.3)
Peru	4.37 (3.54 to 5.34)	21.4% (10.4 to 33.6)	0.05 (0.03 to 0.06)	-57.4% (-73.0 to -34.2)	0.40 (0.26 to 0.59)	20.9% (9.2 to 33.9)	1.28 (0.86 to 1.70)	-56.7% (-72.1 to -31.8)
Tropical Latin America	14.57 (11.44 to 18.04)	18.3% (9.0 to 33.3)	0.58 (0.48 to 0.81)	-53.9% (-63.4 to -17.1)	1.27 (0.81 to 1.85)	18.5% (7.4 to 35.8)	18.25 (15.07 to 25.72)	-53.8% (-63.1 to -13.9)
Brazil	14.78 (11.58 to 18.32)	18.4% (9.1 to 33.5)	0.60 (0.49 to 0.83)	-54.1% (-63.6 to -17.1)	1.29 (0.83 to 1.88)	18.7% (7.4 to 36.1)	18.63 (15.36 to 26.26)	-53.8% (-63.1 to -13.8)
Paraguay	6.22 (5.11 to 7.53)	16.9% (6.8 to 29.4)	0.17 (0.12 to 0.22)	8.5% (-32.2 to 101.8)	0.55 (0.35 to 0.79)	16.8% (-3.9 to 41.7)	3.62 (2.63 to 4.74)	7.0% (-34.4 to 107.8)
Central Latin America	6.63 (5.37 to 7.96)	18.0% (11.9 to 25.8)	0.16 (0.14 to 0.20)	-52.9% (-62.5 to -41.6)	0.60 (0.39 to 0.86)	17.5% (9.9 to 26.4)	4.55 (3.75 to 5.27)	-50.9% (-61.0 to -38.0)
Colombia	5.06 (4.24 to 5.94)	6.1% (-0.9 to 15.3)	0.05 (0.03 to 0.10)	-89.1% (-92.7 to -67.4)	0.47 (0.31 to 0.66)	5.9% (-2.0 to 15.8)	1.30 (0.89 to 2.83)	-88.4% (-92.9 to -61.5)
Costa Rica	12.13 (10.11 to 14.36)	2.6% (-6.5 to 12.4)	0.32 (0.24 to 0.43)	-49.8% (-64.9 to -29.6)	1.06 (0.68 to 1.55)	2.9% (-18.4 to 31.7)	8.42 (5.89 to 11.40)	-45.2% (-62.4 to -20.8)
El Salvador	1.69 (1.40 to 2.03)	17.8% (7.4 to 27.9)	0.05 (0.04 to 0.07)	-24.6% (-48.7 to 15.1)	0.16 (0.10 to 0.23)	17.8% (7.4 to 27.9)	1.26 (0.91 to 1.89)	-25.6% (-50.9 to 17.9)
Guatemala	2.99 (2.49 to 3.61)	12.9% (4.4 to 23.4)	0.09 (0.07 to 0.11)	-36.3% (-58.0 to -5.4)	0.28 (0.18 to 0.40)	12.9% (4.4 to 23.4)	2.09 (1.65 to 2.68)	-41.7% (-61.7 to -17.4)
Honduras	7.75 (6.37 to 9.40)	22.8% (11.9 to 35.9)	0.38 (0.26 to 0.53)	-37.0% (-58.7 to -9.2)	0.69 (0.45 to 1.02)	22.5% (3.7 to 47.0)	10.41 (6.83 to 15.23)	-43.9% (-64.5 to -15.0)
Mexico	6.30 (5.09 to 7.59)	14.9% (10.4 to 21.2)	0.15 (0.11 to 0.17)	-16.2% (-42.9 to 3.3)	0.57 (0.37 to 0.84)	14.2% (9.1 to 21.6)	4.32 (3.12 to 4.95)	-14.3% (-44.9 to 7.2)
Nicaragua	8.28 (6.94 to 9.82)	27.3% (16.9 to 38.1)	0.18 (0.14 to 0.25)	-30.6% (-50.6 to -5.1)	0.74 (0.47 to 1.08)	26.2% (8.6 to 46.4)	4.51 (3.43 to 6.37)	-33.3% (-52.7 to -8.4)
Panama	8.04 (6.72 to 9.49)	2.8% (-4.6 to 11.2)	0.23 (0.18 to 0.36)	0.6% (-29.7 to 43.3)	0.71 (0.47 to 1.03)	2.9% (-10.4 to 19.2)	5.38 (4.08 to 8.54)	4.3% (-27.3 to 49.6)
Venezuela	11.64 (8.72 to 14.95)	36.5% (13.1 to 73.5)	0.43 (0.32 to 0.62)	-60.5% (-73.6 to -43.6)	1.01 (0.61 to 1.53)	36.9% (4.7 to 92.9)	10.71 (7.87 to 14.01)	-59.9% (-74.3 to -38.4)
Southern Latin America	11.84 (8.72 to 15.24)	-9.2% (-17.9 to 1.0)	0.27 (0.22 to 0.42)	-79.2% (-85.5 to -47.7)	1.02 (0.63 to 1.55)	-8.2% (-23.4 to 9.5)	7.36 (5.84 to 11.62)	-79.9% (-86.2 to -47.2)
Argentina	9.79 (6.81 to 12.97)	7.5% (-10.6 to 32.7)	0.21 (0.16 to 0.37)	-83.3% (-89.8 to -45.2)	0.85 (0.50 to 1.31)	8.1% (-21.9 to 51.4)	6.11 (4.49 to 10.35)	-83.8% (-90.1 to -46.7)
Chile	12.64 (9.24 to 16.27)	-19.0% (-29.0 to -8.5)	0.26 (0.19 to 0.43)	-75.8% (-82.3 to -38.7)	1.10 (0.66 to 1.65)	-17.6% (-36.6 to 5.6)	7.45 (5.66 to 11.85)	-74.5% (-81.4 to -39.4)
Uruguay	29.93 (24.12 to 37.24)	-23.1% (-30.3 to -14.5)	0.89 (0.67 to 1.11)	-56.0% (-73.1 to -40.8)	2.53 (1.63 to 3.74)	-22.6% (-35.4 to -6.5)	20.40 (15.51 to 26.70)	-57.9% (-75.0 to -40.0)
Caribbean	25.30 (21.09 to 30.12)	7.0% (-0.1 to 15.4)	1.25 (0.84 to 1.45)	33.4% (-12.0 to 64.5)	2.19 (1.45 to 3.16)	6.9% (-5.5 to 21.1)	35.13 (23.49 to 41.04)	27.9% (-10.6 to 59.6)
Antigua and Barbuda	7.17 (5.85 to 8.65)	1.6% (-7.7 to 11.6)	0.45 (0.31 to 0.54)	51.8% (-6.0 to 107.3)	0.64 (0.41 to 0.93)	0.9% (-16.6 to 22.2)	12.21 (8.05 to 14.98)	49.6% (-7.3 to 106.5)
The Bahamas	13.78 (11.37 to 16.62)	-3.8% (-12.5 to 6.3)	1.51 (1.14 to 1.85)	-15.9% (-49.9 to 37.8)	1.20 (0.76 to 1.78)	-4.1% (-23.9 to 19.7)	46.74 (34.63 to 57.88)	-17.8% (-52.0 to 39.3)
Barbados	26.85 (22.28 to 32.13)	-0.6% (-8.9 to 7.8)	1.23 (0.97 to 1.49)	9.6% (-21.0 to 43.3)	2.31 (1.50 to 3.32)	-0.9% (-16.8 to 17.8)	35.65 (27.70 to 43.19)	9.1% (-21.2 to 41.3)
Belize	8.88 (7.42 to 10.65)	5.7% (-3.4 to 14.2)	0.55 (0.40 to 0.65)	41.5% (-20.0 to 96.4)	0.79 (0.51 to 1.18)	5.1% (-13.1 to 24.8)	16.17 (11.64 to 19.32)	50.6% (-17.8 to 111.4)
Bermuda	13.97 (11.75 to 16.60)	-6.7% (-13.7 to 0.7)	0.37 (0.22 to 0.47)	-20.3% (-71.8 to 28.7)	1.22 (0.78 to 1.79)	-6.3% (-26.1 to 16.8)	9.45 (5.94 to 12.24)	-21.5% (-74.5 to 31.7)
Cuba	50.00 (41.59 to 59.87)	19.3% (8.5 to 32.8)	2.28 (1.31 to 2.76)	160.0% (27.0 to 243.5)	4.33 (2.83 to 6.24)	19.2% (0.3 to 41.2)	64.80 (36.86 to 79.43)	134.8% (26.1 to 210.0)
Dominica	8.09 (6.71 to 9.72)	1.6% (-5.9 to 10.0)	0.96 (0.68 to 1.19)	49.5% (-14.1 to 116.2)	0.72 (0.47 to 1.07)	1.1% (-19.3 to 25.6)	27.16 (19.23 to 33.83)	49.8% (-15.9 to 120.6)
Dominican Republic	4.30 (3.63 to 5.05)	15.9% (7.2 to 25.3)	0.20 (0.15 to 0.26)	26.9% (-18.1 to 115.8)	0.40 (0.25 to 0.57)	15.3% (6.4 to 25.2)	5.35 (3.89 to 7.41)	34.6% (-13.6 to 134.0)
Grenada	18.00 (14.86 to 21.68)	6.7% (-2.6 to 16.6)	0.98 (0.82 to 1.20)	-32.9% (-51.3 to -6.8)	1.57 (1.02 to 2.28)	6.6% (-13.5 to 31.0)	28.38 (23.53 to 34.45)	-33.5% (-52.7 to -5.8)
Guyana	13.53 (11.20 to 16.33)	-0.6% (-8.7 to 8.1)	1.23 (0.83 to 1.57)	-44.1% (-62.8 to -22.1)	1.18 (0.77 to 1.69)	-0.1% (-19.3 to 24.9)	35.98 (24.45 to 46.77)	-44.5% (-63.7 to -20.0)
Haiti	12.21 (10.05 to 14.74)	6.4% (-1.8 to 15.9)	1.29 (0.78 to 1.92)	-29.9% (-51.7 to 8.3)	1.06 (0.68 to 1.53)	5.9% (-12.6 to 31.5)	36.43 (21.99 to 54.61)	-33.7% (-54.4 to 3.6)
Jamaica	12.97 (10.65 to 15.72)	13.1% (2.5 to 24.2)	0.62 (0.40 to 1.06)	18.4% (-30.7 to 85.1)	1.13 (0.72 to 1.63)	13.0% (-12.1 to 44.9)	16.60 (10.51 to 30.16)	27.4% (-27.7 to 112.6)
Puerto Rico	20.08 (16.81 to 23.81)	4.2% (-5.0 to 13.6)	0.39 (0.30 to 0.60)	-66.0% (-80.0 to -44.5)	1.73 (1.13 to 2.49)	4.3% (-15.7 to 28.1)	10.31 (7.50 to 17.78)	-63.6% (-79.0 to -34.6)
Saint Lucia	28.46 (23.93 to 33.94)	-0.1% (-8.5 to 8.5)	1.98 (1.35 to 2.41)	11.2% (-47.0 to 51.1)	2.45 (1.57 to 3.53)	0.3% (-17.0 to 20.4)	57.31 (37.94 to 70.19)	15.2% (-46.8 to 57.3)
Saint Vincent and the Grenadines	13.51 (11.02 to 16.56)	7.0% (-2.3 to 18.2)	1.23 (0.90 to 1.47)	66.6% (-5.0 to 121.9)	1.17 (0.75 to 1.71)	5.7% (-17.2 to 32.7)	36.16 (25.53 to 43.59)	73.8% (-2.0 to 131.0)
Suriname	7.28 (6.04 to 8.76)	2.2% (-5.8 to 11.8)	0.47 (0.34 to 0.57)	20.6% (-20.1 to 61.9)	0.66 (0.43 to 0.97)	1.9% (-12.7 to 19.9)	13.77 (9.86 to 17.02)	24.7% (-16.8 to 66.3)
Trinidad and Tobago	11.61 (9.55 to 14.03)	0.5% (-8.4 to 11.8)	0.66 (0.49 to 0.91)	-12.9% (-38.4 to 20.4)	1.00 (0.64 to 1.46)	-0.1% (-21.1 to 24.5)	16.78 (12.39 to 24.36)	-10.5% (-36.6 to 23.2)
Virgin Islands, U.S.	36.05 (30.25 to 42.63)	8.6% (-0.2 to 18.5)	2.90 (2.17 to 3.66)	22.0% (-14.1 to 65.6)	3.09 (2.02 to 4.40)	7.8% (-10.6 to 28.6)	80.63 (59.58 to 102.69)	27.3% (-11.8 to 72.4)
Central Europe	58.08 (49.30 to 67.92)	5.2% (0.7 to 9.9)	2.66 (1.92 to 3.10)	-8.1% (-45.7 to 16.2)	4.88 (3.31 to 6.84)	5.6% (-1.7 to 13.2)	74.87 (51.10 to 84.34)	-2.7% (-38.9 to 21.5)
Albania	22.22 (18.55 to 25.93)	2.4% (-4.9 to 10.7)	0.61 (0.38 to 0.87)	-53.0% (-75.9 to -25.3)	1.89 (1.24 to 2.71)	2.7% (-14.3 to 22.2)	12.02 (6.83 to 17.69)	-50.9% (-75.0 to -24.6)
Bosnia and Herzegovina	57.39 (47.88 to 67.90)	-1.1% (-8.8 to 7.0)	2.02 (1.06 to 3.07)	17.5% (-20.3 to 74.5)	4.77 (3.21 to 6.66)	-2.1% (-15.4 to 14.7)	48.78 (24.01 to 78.29)	20.5% (-19.8 to 78.3)
Bulgaria	6.06 (5.10 to 7.18)	-7.8% (-14.1 to -0.7)	0.27 (0.22 to 0.36)	31.4% (-36.4 to 177.0)	0.56 (0.37 to 0.80)	-7.2% (-15.2 to 1.5)	7.82 (6.19 to 10.83)	34.8% (-36.2 to 175.8)
Croatia	73.27 (63.63 to 83.62)	-26.5% (-34.7 to -16.8)	2.44 (1.69 to 2.84)	76.9% (-39.0 to 137.8)	6.05 (4.04 to 8.43)	-26.6% (-37.5 to -14.5)	52.20 (38.32 to 60.23)	94.2% (-42.2 to 174.9)

Czech Republic	23.67 (19.40 to 28.68)	4.0% (-7.7 to 19.4)	0.60 (0.25 to 0.79)	379.3% (-24.5 to 652.6)	2.03 (1.30 to 2.98)	3.9% (-16.1 to 31.8)	19.43 (7.50 to 25.84)	376.0% (-23.9 to 664.6)
Hungary	139.79 (116.96 to 166.39)	9.2% (0.9 to 18.0)	6.66 (4.57 to 7.51)	-5.5% (-55.6 to 18.7)	11.68 (7.84 to 16.28)	10.0% (-2.4 to 25.0)	184.78 (124.18 to 208.92)	-5.2% (-52.8 to 16.7)
Macedonia	34.74 (29.22 to 40.88)	-13.7% (-20.5 to -7.1)	1.33 (0.77 to 2.06)	-63.7% (-73.4 to -49.9)	2.90 (1.94 to 4.08)	-13.3% (-26.5 to 3.0)	25.12 (14.95 to 37.22)	-59.1% (-71.2 to -42.9)
Montenegro	356.08 (300.91 to 419.63)	1.9% (-5.1 to 9.9)	11.90 (9.14 to 14.76)	12.4% (-8.9 to 39.7)	29.21 (19.57 to 40.72)	1.5% (-7.2 to 11.2)	221.68 (165.69 to 284.83)	8.9% (-8.4 to 28.4)
Poland	60.76 (50.28 to 71.84)	22.2% (10.9 to 35.4)	3.11 (1.63 to 3.70)	170.0% (37.0 to 297.0)	5.13 (3.38 to 7.37)	22.3% (3.2 to 44.3)	94.79 (47.63 to 112.45)	156.1% (42.1 to 274.6)
Romania	63.04 (52.55 to 75.69)	0.6% (-7.8 to 10.7)	3.08 (2.46 to 4.30)	-46.6% (-63.9 to -29.0)	5.32 (3.57 to 7.57)	1.2% (-14.3 to 18.8)	90.47 (72.23 to 125.80)	-49.2% (-66.3 to -31.4)
Serbia	27.06 (22.99 to 31.86)	-29.4% (-37.1 to -20.5)	1.75 (0.86 to 4.06)	-38.5% (-61.1 to -10.1)	2.25 (1.48 to 3.14)	-28.7% (-40.0 to -14.5)	28.60 (16.94 to 51.03)	-40.2% (-57.9 to -18.6)
Slovakia	31.11 (25.82 to 36.80)	25.1% (11.4 to 41.3)	1.56 (1.09 to 1.88)	264.3% (93.5 to 451.6)	2.63 (1.74 to 3.85)	24.6% (1.0 to 57.2)	48.17 (34.39 to 58.32)	228.9% (67.4 to 411.8)
Slovenia	83.71 (72.09 to 97.71)	-8.6% (-16.5 to 1.6)	0.95 (0.69 to 1.81)	-94.6% (-96.2 to -92.8)	6.88 (4.72 to 9.67)	-8.3% (-19.9 to 5.3)	13.09 (9.78 to 29.90)	-95.2% (-96.9 to -91.7)
Eastern Europe	115.61 (97.13 to 135.80)	0.9% (-1.2 to 3.2)	17.17 (16.20 to 19.10)	96.9% (57.6 to 160.4)	9.79 (6.74 to 13.77)	1.1% (-2.3 to 4.9)	643.71 (606.61 to 718.49)	114.2% (70.2 to 187.8)
Belarus	97.82 (81.72 to 115.16)	10.6% (2.6 to 18.6)	6.55 (5.41 to 8.18)	11.2% (-13.9 to 53.1)	8.30 (5.66 to 11.68)	10.4% (-3.8 to 26.8)	240.91 (197.18 to 302.77)	20.1% (-9.9 to 67.9)
Estonia	43.51 (36.03 to 51.64)	21.8% (11.4 to 33.9)	4.34 (3.24 to 6.59)	93.5% (33.9 to 179.1)	3.70 (2.45 to 5.24)	21.9% (2.7 to 45.9)	135.19 (100.49 to 211.58)	74.8% (21.3 to 156.1)
Latvia	55.77 (47.20 to 65.53)	11.4% (2.0 to 21.5)	15.60 (10.48 to 18.80)	720.1% (153.9 to 1333.6)	4.71 (3.08 to 6.72)	11.3% (-4.9 to 31.4)	581.55 (381.63 to 698.94)	711.1% (154.9 to 1318.2)
Lithuania	91.13 (76.46 to 108.01)	15.9% (7.2 to 26.1)	5.61 (4.64 to 7.81)	96.6% (46.6 to 151.0)	7.73 (5.14 to 10.78)	15.7% (0.4 to 34.6)	200.70 (165.91 to 284.11)	93.5% (43.5 to 148.7)
Moldova	38.97 (31.77 to 46.64)	36.9% (26.0 to 50.4)	3.86 (1.93 to 4.46)	872.6% (61.9 to 1635.2)	3.34 (2.22 to 4.75)	36.4% (11.5 to 64.5)	128.89 (63.77 to 149.42)	834.7% (47.2 to 1622.1)
Russian Federation	118.64 (99.64 to 139.11)	-2.8% (-4.3 to -1.2)	18.66 (17.33 to 21.46)	110.9% (47.4 to 184.9)	10.05 (6.85 to 14.01)	-2.4% (-4.9 to 0.1)	693.16 (635.52 to 801.03)	127.4% (55.0 to 216.5)
Ukraine	122.75 (102.53 to 145.58)	7.3% (-0.1 to 15.2)	16.98 (14.95 to 21.60)	62.5% (27.3 to 107.1)	10.41 (7.12 to 15.00)	7.3% (-4.6 to 21.1)	657.66 (570.14 to 846.20)	82.9% (42.7 to 138.9)
North Africa and Middle East	5.00 (4.26 to 5.83)	13.1% (10.2 to 15.7)	0.17 (0.14 to 0.19)	-42.3% (-60.8 to -21.5)	0.45 (0.30 to 0.64)	13.2% (9.1 to 17.4)	4.06 (3.25 to 4.87)	-45.2% (-60.9 to -26.2)
Afghanistan	5.25 (4.37 to 6.26)	1.2% (-6.0 to 8.7)	0.40 (0.21 to 0.69)	-40.7% (-58.2 to 12.4)	0.48 (0.32 to 0.69)	1.2% (-6.5 to 9.7)	11.72 (4.82 to 19.35)	-42.3% (-61.4 to 30.1)
Algeria	5.74 (4.79 to 6.73)	10.7% (1.5 to 20.4)	0.18 (0.10 to 0.33)	-39.1% (-63.8 to -10.7)	0.51 (0.33 to 0.74)	10.0% (-6.0 to 27.8)	3.87 (1.99 to 6.72)	-44.3% (-66.7 to -19.8)
Bahrain	14.16 (11.90 to 16.94)	-3.4% (-10.7 to 5.1)	0.29 (0.23 to 0.37)	-54.6% (-72.1 to -30.1)	1.21 (0.79 to 1.76)	-3.1% (-19.8 to 17.3)	5.28 (4.13 to 6.74)	-59.0% (-75.0 to -37.8)
Egypt	5.54 (4.63 to 6.56)	33.0% (23.9 to 44.0)	0.23 (0.14 to 0.42)	-34.7% (-54.6 to -6.4)	0.51 (0.34 to 0.73)	32.0% (21.5 to 43.4)	5.81 (3.58 to 10.09)	-35.9% (-54.0 to -9.8)
Iran	3.74 (3.11 to 4.45)	6.9% (4.5 to 9.3)	0.14 (0.11 to 0.17)	-31.9% (-60.2 to -5.8)	0.34 (0.23 to 0.49)	6.9% (4.1 to 9.8)	2.96 (2.37 to 3.70)	-37.7% (-63.2 to -13.8)
Iraq	4.57 (3.82 to 5.46)	10.8% (2.9 to 19.4)	0.05 (0.03 to 0.06)	-86.6% (-91.0 to -75.1)	0.42 (0.28 to 0.60)	10.8% (2.9 to 19.4)	1.36 (1.01 to 1.68)	-88.1% (-92.2 to -75.8)
Jordan	2.45 (2.04 to 2.87)	5.5% (-2.3 to 13.7)	0.04 (0.03 to 0.06)	-58.9% (-78.9 to -27.8)	0.23 (0.15 to 0.33)	5.5% (-2.3 to 13.7)	1.21 (0.94 to 1.57)	-64.2% (-82.2 to -35.1)
Kuwait	3.65 (3.07 to 4.30)	1.5% (-5.8 to 9.5)	0.07 (0.05 to 0.09)	-32.3% (-56.3 to -8.9)	0.34 (0.22 to 0.48)	1.7% (-6.9 to 11.7)	1.67 (1.17 to 2.18)	-38.9% (-62.2 to -18.1)
Lebanon	5.63 (4.77 to 6.66)	2.1% (-4.6 to 10.1)	0.14 (0.09 to 0.23)	-56.0% (-74.5 to -31.5)	0.51 (0.34 to 0.73)	1.8% (-7.3 to 12.2)	3.28 (2.12 to 5.45)	-56.8% (-75.6 to -32.9)
Libya	5.26 (4.41 to 6.19)	21.7% (12.9 to 31.5)	0.16 (0.10 to 0.26)	-28.6% (-57.4 to 18.9)	0.48 (0.32 to 0.68)	20.8% (8.5 to 35.0)	4.02 (2.46 to 6.75)	-23.7% (-53.1 to 23.2)
Morocco	5.55 (4.66 to 6.59)	18.7% (9.6 to 28.0)	0.20 (0.14 to 0.28)	-43.1% (-61.1 to -14.5)	0.51 (0.33 to 0.73)	18.6% (7.2 to 31.0)	4.63 (3.32 to 6.52)	-48.7% (-64.4 to -20.1)
Palestine	11.40 (9.63 to 13.44)	13.1% (5.6 to 21.2)	0.26 (0.20 to 0.35)	-60.5% (-74.5 to -35.4)	0.99 (0.66 to 1.43)	12.4% (-3.5 to 30.1)	5.45 (4.34 to 7.51)	-62.4% (-76.6 to -38.8)
Oman	15.48 (13.07 to 18.21)	31.8% (19.1 to 45.4)	0.29 (0.19 to 0.48)	-34.9% (-65.4 to 48.5)	1.33 (0.86 to 1.94)	30.1% (2.1 to 65.8)	7.44 (4.58 to 12.08)	-34.3% (-65.7 to 47.4)
Qatar	11.14 (9.34 to 13.05)	4.4% (-3.9 to 12.9)	0.16 (0.12 to 0.23)	-75.1% (-86.6 to -56.5)	0.98 (0.64 to 1.40)	4.6% (-12.4 to 24.4)	3.45 (2.31 to 5.08)	-72.6% (-86.0 to -46.2)
Saudi Arabia	15.76 (13.24 to 18.71)	3.2% (-4.9 to 11.9)	0.58 (0.28 to 1.11)	16.8% (-63.2 to 201.9)	1.34 (0.89 to 1.91)	3.4% (-13.8 to 25.5)	10.23 (4.89 to 19.54)	9.8% (-65.0 to 179.4)
Sudan	6.17 (5.14 to 7.27)	10.1% (2.0 to 18.5)	0.28 (0.17 to 0.47)	-44.9% (-62.2 to -13.9)	0.56 (0.37 to 0.80)	9.6% (-0.9 to 21.2)	6.95 (4.29 to 11.03)	-51.7% (-67.1 to -20.1)
Syria	7.14 (6.04 to 8.41)	21.8% (13.2 to 30.4)	0.22 (0.15 to 0.32)	-39.9% (-66.6 to -2.4)	0.64 (0.41 to 0.92)	20.4% (6.9 to 35.4)	4.91 (3.24 to 6.89)	-44.3% (-68.4 to -10.0)
Tunisia	5.20 (4.37 to 6.12)	13.4% (4.6 to 23.9)	0.17 (0.10 to 0.27)	-37.0% (-68.1 to 11.8)	0.47 (0.31 to 0.68)	12.2% (-0.7 to 27.1)	3.29 (1.90 to 5.18)	-36.0% (-67.2 to 10.4)
Turkey	1.94 (1.67 to 2.23)	3.7% (-5.1 to 13.6)	0.04 (0.03 to 0.04)	-37.5% (-65.4 to 0.9)	0.18 (0.12 to 0.25)	3.7% (-5.1 to 13.6)	1.01 (0.81 to 1.26)	-37.0% (-65.8 to 0.7)
United Arab Emirates	8.59 (7.21 to 10.20)	-10.7% (-17.3 to -3.2)	0.33 (0.14 to 0.96)	-47.1% (-78.2 to 4.4)	0.77 (0.50 to 1.09)	-9.8% (-22.6 to 4.0)	7.87 (3.34 to 22.94)	-49.5% (-81.0 to 8.0)
Yemen	4.98 (4.20 to 5.92)	18.0% (9.3 to 26.4)	0.25 (0.16 to 0.37)	-49.5% (-70.6 to -4.7)	0.45 (0.30 to 0.65)	16.4% (5.8 to 27.3)	6.09 (3.85 to 9.02)	-56.2% (-74.1 to -11.1)
Central Asia	29.71 (24.88 to 35.09)	-2.0% (-6.9 to 3.2)	3.02 (1.50 to 3.69)	145.4% (2.7 to 219.4)	2.54 (1.69 to 3.56)	-2.1% (-10.3 to 7.1)	102.86 (50.56 to 127.33)	162.2% (6.3 to 243.2)
Armenia	74.49 (63.18 to 87.31)	-2.8% (-9.6 to 4.2)	0.63 (0.45 to 1.26)	-86.6% (-91.3 to -71.4)	6.30 (4.30 to 8.97)	-2.5% (-15.5 to 12.2)	14.22 (10.42 to 34.82)	-85.0% (-89.9 to -58.6)
Azerbaijan	29.21 (24.37 to 34.56)	-1.6% (-9.9 to 6.3)	1.40 (0.91 to 2.24)	-23.8% (-42.5 to 3.8)	2.51 (1.63 to 3.59)	-2.0% (-19.3 to 19.1)	42.16 (24.05 to 70.34)	-28.8% (-45.2 to -4.8)
Georgia	5.21 (4.39 to 6.04)	-0.4% (-7.5 to 7.4)	0.45 (0.29 to 0.86)	102.2% (39.5 to 290.6)	0.48 (0.32 to 0.68)	-0.4% (-7.9 to 7.8)	13.93 (8.44 to 27.05)	102.5% (32.8 to 310.5)
Kazakhstan	61.33 (50.16 to 73.37)	8.4% (-0.7 to 18.4)	9.91 (3.60 to 12.89)	988.5% (58.9 to 1722.3)	5.22 (3.40 to 7.43)	8.5% (-7.7 to 27.6)	346.69 (126.07 to 454.36)	946.3% (58.9 to 1647.4)
Kyrgyzstan	75.29 (62.39 to 90.20)	27.5% (16.7 to 38.9)	5.79 (3.20 to 6.77)	31.3% (-7.8 to 55.2)	6.46 (4.23 to 9.15)	27.8% (7.7 to 51.8)	207.80 (113.63 to 243.90)	29.0% (-10.6 to 53.1)
Mongolia	9.87 (8.18 to 11.87)	18.3% (8.0 to 28.9)	0.71 (0.48 to 1.21)	-3.1% (-34.0 to 35.9)	0.88 (0.56 to 1.27)	16.9% (-5.3 to 44.8)	20.97 (14.56 to 32.48)	7.2% (-30.4 to 54.9)

Tajikistan	1.41 (1.17 to 1.69)	1.0% (-6.8 to 8.6)	0.06 (0.05 to 0.10)	-15.6% (-38.6 to 17.9)	0.13 (0.09 to 0.19)	1.0% (-6.8 to 8.6)	2.31 (1.66 to 3.78)	-18.2% (-40.2 to 18.7)
Turkmenistan	53.52 (44.79 to 63.74)	3.4% (-4.2 to 12.2)	3.87 (3.03 to 4.65)	-15.4% (-33.9 to 6.3)	4.57 (3.02 to 6.61)	3.7% (-11.9 to 22.4)	134.34 (103.24 to 161.14)	-10.8% (-30.8 to 12.6)
Uzbekistan	1.51 (1.26 to 1.77)	9.4% (1.1 to 17.8)	0.09 (0.07 to 0.15)	141.9% (47.9 to 420.4)	0.14 (0.09 to 0.20)	9.4% (1.1 to 17.8)	3.19 (2.36 to 5.24)	150.8% (48.0 to 438.1)
South Asia	7.53 (6.24 to 8.95)	5.7% (3.5 to 7.9)	0.42 (0.27 to 0.63)	-7.8% (-41.3 to 32.4)	0.68 (0.45 to 0.99)	5.4% (1.7 to 8.9)	11.00 (6.56 to 16.68)	-10.2% (-42.1 to 27.4)
Bangladesh	10.05 (8.42 to 11.89)	10.1% (2.0 to 19.1)	0.31 (0.15 to 0.52)	-26.1% (-53.9 to 23.2)	0.89 (0.59 to 1.28)	9.8% (-6.8 to 30.2)	8.02 (3.32 to 14.68)	-31.3% (-58.9 to 20.5)
Bhutan	10.80 (9.05 to 12.81)	16.0% (7.1 to 25.7)	0.37 (0.18 to 0.67)	-32.1% (-65.9 to 34.3)	0.95 (0.61 to 1.36)	15.3% (-6.7 to 43.5)	8.90 (3.94 to 16.54)	-37.5% (-69.1 to 29.9)
India	6.96 (5.70 to 8.34)	6.7% (4.9 to 8.6)	0.42 (0.28 to 0.62)	-6.9% (-43.0 to 34.5)	0.63 (0.42 to 0.92)	6.1% (3.4 to 8.9)	11.05 (6.94 to 16.30)	-9.0% (-43.4 to 29.6)
Nepal	8.55 (7.16 to 10.15)	11.6% (3.2 to 20.3)	0.32 (0.13 to 0.56)	-0.2% (-34.7 to 69.7)	0.76 (0.50 to 1.11)	10.1% (-6.7 to 29.7)	7.88 (3.01 to 13.91)	-8.7% (-41.5 to 54.7)
Pakistan	10.28 (8.60 to 12.11)	5.0% (-3.3 to 13.3)	0.58 (0.26 to 1.00)	6.0% (-31.4 to 63.6)	0.90 (0.59 to 1.30)	4.3% (-13.0 to 23.9)	14.20 (5.97 to 25.61)	-0.9% (-34.3 to 51.1)
Southeast Asia	6.64 (5.56 to 7.84)	16.5% (13.6 to 19.6)	0.35 (0.28 to 0.45)	3.7% (-22.6 to 34.6)	0.60 (0.40 to 0.86)	15.9% (11.1 to 21.0)	9.61 (7.67 to 12.57)	4.7% (-22.6 to 34.8)
Cambodia	6.48 (5.37 to 7.81)	10.5% (2.6 to 18.5)	0.36 (0.25 to 0.51)	-21.4% (-49.0 to 27.7)	0.59 (0.39 to 0.85)	9.9% (-0.3 to 20.2)	9.37 (6.52 to 13.23)	-25.4% (-51.4 to 25.1)
Indonesia	7.12 (5.85 to 8.63)	23.5% (20.0 to 26.8)	0.32 (0.20 to 0.55)	-4.0% (-28.2 to 33.4)	0.65 (0.43 to 0.93)	22.2% (17.4 to 27.2)	8.33 (4.84 to 14.90)	-8.0% (-33.4 to 28.1)
Laos	7.48 (6.24 to 8.95)	16.2% (7.7 to 25.5)	0.49 (0.34 to 0.73)	-46.3% (-64.1 to -10.9)	0.68 (0.44 to 0.98)	15.2% (3.2 to 29.7)	13.79 (9.23 to 20.41)	-49.2% (-66.8 to -15.2)
Malaysia	9.01 (7.50 to 10.60)	14.4% (6.5 to 22.3)	0.29 (0.22 to 0.39)	-21.5% (-46.7 to 16.2)	0.81 (0.53 to 1.16)	13.7% (-0.8 to 32.1)	7.77 (5.79 to 11.52)	-20.1% (-47.6 to 19.5)
Maldives	4.38 (3.61 to 5.22)	36.3% (25.3 to 49.4)	0.11 (0.08 to 0.15)	-73.9% (-85.0 to -17.6)	0.41 (0.27 to 0.60)	36.4% (25.3 to 49.4)	2.33 (1.89 to 2.83)	-80.1% (-88.5 to -22.4)
Mauritius	8.64 (7.24 to 10.19)	-0.4% (-7.3 to 7.4)	0.38 (0.29 to 0.60)	0.7% (-26.8 to 72.6)	0.79 (0.52 to 1.13)	-0.9% (-11.5 to 12.2)	12.79 (9.64 to 22.11)	7.8% (-25.6 to 95.8)
Myanmar	4.32 (3.58 to 5.15)	11.8% (4.0 to 20.5)	0.24 (0.15 to 0.36)	-31.3% (-61.9 to 13.7)	0.40 (0.26 to 0.58)	11.8% (4.1 to 20.6)	6.53 (4.00 to 9.86)	-34.2% (-62.9 to 11.7)
Philippines	6.21 (5.20 to 7.37)	1.5% (-6.8 to 11.2)	0.70 (0.51 to 1.07)	49.4% (-2.6 to 194.4)	0.56 (0.37 to 0.81)	1.9% (-9.3 to 14.4)	17.05 (13.18 to 22.11)	43.0% (-11.7 to 173.0)
Sri Lanka	21.33 (17.52 to 25.36)	20.5% (10.3 to 31.0)	1.30 (0.78 to 1.77)	32.7% (-22.8 to 123.1)	1.83 (1.19 to 2.62)	19.9% (-2.1 to 50.3)	36.59 (22.08 to 50.25)	29.8% (-26.1 to 117.6)
Seychelles	16.58 (13.71 to 19.91)	-3.7% (-11.9 to 5.9)	1.88 (1.58 to 2.24)	-25.8% (-46.4 to 7.1)	1.44 (0.94 to 2.08)	-3.7% (-22.2 to 17.7)	52.44 (42.38 to 63.51)	-23.6% (-45.0 to 6.1)
Thailand	1.85 (1.54 to 2.20)	11.8% (3.5 to 20.7)	0.04 (0.03 to 0.05)	-1.7% (-29.7 to 41.2)	0.17 (0.11 to 0.25)	11.8% (3.5 to 20.7)	1.24 (1.01 to 1.54)	12.2% (-20.5 to 65.5)
Timor-Leste	6.95 (5.81 to 8.26)	24.3% (14.3 to 34.9)	0.37 (0.14 to 0.76)	-16.8% (-55.3 to 55.3)	0.63 (0.42 to 0.90)	22.9% (11.4 to 36.2)	9.70 (3.20 to 21.69)	-21.3% (-63.2 to 50.9)
Vietnam	7.11 (5.95 to 8.40)	14.7% (5.3 to 25.3)	0.35 (0.22 to 0.52)	19.1% (-38.1 to 151.9)	0.65 (0.42 to 0.94)	14.5% (0.7 to 30.5)	9.72 (5.88 to 15.32)	31.0% (-37.8 to 188.0)
East Asia	4.57 (3.75 to 5.48)	20.4% (17.6 to 23.4)	0.16 (0.13 to 0.22)	89.4% (20.5 to 153.4)	0.42 (0.28 to 0.60)	20.3% (16.3 to 24.8)	4.94 (4.11 to 6.35)	82.9% (18.6 to 147.3)
China	4.24 (3.43 to 5.14)	22.1% (19.4 to 24.9)	0.16 (0.13 to 0.22)	106.0% (25.9 to 181.4)	0.39 (0.26 to 0.56)	21.9% (18.4 to 25.7)	4.88 (4.03 to 6.29)	94.5% (22.6 to 168.5)
North Korea	5.31 (4.37 to 6.29)	0.7% (-6.8 to 9.4)	0.18 (0.13 to 0.24)	8.8% (-24.4 to 54.1)	0.49 (0.32 to 0.70)	0.8% (-6.8 to 9.4)	5.90 (3.93 to 8.30)	12.4% (-26.7 to 63.5)
Taiwan (Province of China)	22.34 (19.70 to 24.96)	11.8% (-2.8 to 28.9)	0.20 (0.15 to 0.26)	-47.1% (-62.5 to -34.3)	1.98 (1.31 to 2.82)	12.6% (-12.1 to 45.9)	7.02 (5.09 to 9.13)	-32.4% (-51.8 to -13.6)
Oceania	3.99 (3.32 to 4.75)	8.4% (3.5 to 13.2)	0.61 (0.42 to 0.87)	-30.1% (-44.5 to -8.9)	0.36 (0.24 to 0.52)	7.7% (2.0 to 13.5)	18.08 (11.77 to 25.86)	-30.7% (-46.9 to -4.0)
American Samoa	8.75 (7.31 to 10.37)	3.9% (-3.6 to 12.5)	0.42 (0.34 to 0.52)	-51.1% (-65.1 to -31.1)	0.76 (0.49 to 1.10)	3.2% (-13.5 to 23.5)	10.43 (8.37 to 12.95)	-48.0% (-61.6 to -28.6)
Federated States of Micronesia	5.33 (4.43 to 6.36)	17.1% (8.5 to 26.6)	0.54 (0.38 to 0.74)	-34.7% (-53.3 to -7.0)	0.47 (0.31 to 0.68)	15.6% (-2.5 to 37.1)	14.03 (9.32 to 19.72)	-37.3% (-58.1 to -9.1)
Fiji	2.77 (2.30 to 3.35)	3.1% (-4.9 to 11.3)	0.35 (0.28 to 0.43)	-9.3% (-35.6 to 28.2)	0.26 (0.17 to 0.37)	3.1% (-5.1 to 11.5)	8.62 (6.94 to 10.75)	-9.2% (-34.6 to 25.5)
Guam	8.02 (6.67 to 9.49)	3.5% (-4.2 to 11.5)	0.39 (0.32 to 0.49)	-54.3% (-67.4 to -31.0)	0.70 (0.46 to 1.03)	2.8% (-14.6 to 22.3)	10.62 (8.61 to 13.58)	-47.8% (-63.5 to -15.0)
Kiribati	5.76 (4.76 to 6.88)	1.5% (-5.8 to 10.1)	0.79 (0.56 to 1.10)	-8.9% (-33.0 to 23.7)	0.51 (0.33 to 0.73)	0.9% (-15.0 to 19.4)	23.05 (15.62 to 33.55)	-8.8% (-33.9 to 26.3)
Marshall Islands	6.22 (5.12 to 7.41)	9.8% (0.2 to 20.0)	0.85 (0.57 to 1.22)	-19.2% (-41.5 to 13.3)	0.55 (0.35 to 0.80)	9.0% (-8.5 to 30.1)	23.39 (14.44 to 33.84)	-12.9% (-37.3 to 23.2)
Northern Mariana Islands	13.30 (11.17 to 15.68)	-3.9% (-11.5 to 4.9)	0.39 (0.31 to 0.50)	-50.0% (-64.8 to -30.7)	1.15 (0.75 to 1.67)	-3.9% (-21.3 to 18.9)	9.08 (7.11 to 11.49)	-50.6% (-64.7 to -31.0)
Papua New Guinea	3.62 (2.98 to 4.33)	11.2% (3.4 to 19.8)	0.69 (0.43 to 1.03)	-34.3% (-51.7 to -6.4)	0.33 (0.22 to 0.47)	10.1% (0.9 to 20.2)	20.56 (12.35 to 30.81)	-36.1% (-53.8 to -4.0)
Samoa	5.94 (4.93 to 7.06)	10.4% (2.1 to 18.9)	0.45 (0.25 to 0.73)	-11.1% (-36.0 to 20.2)	0.52 (0.34 to 0.76)	9.4% (-7.5 to 29.7)	10.58 (5.69 to 17.19)	-17.7% (-42.6 to 11.4)
Solomon Islands	4.00 (3.32 to 4.76)	8.0% (-0.4 to 16.4)	0.42 (0.23 to 0.71)	-24.5% (-44.1 to 4.6)	0.36 (0.24 to 0.53)	7.3% (-2.6 to 18.3)	10.96 (6.48 to 17.77)	-28.0% (-49.1 to 3.3)
Tonga	2.42 (2.02 to 2.87)	8.9% (1.0 to 18.4)	0.12 (0.07 to 0.16)	-17.6% (-45.4 to 21.2)	0.22 (0.15 to 0.32)	8.7% (0.3 to 18.3)	2.72 (1.61 to 3.89)	-9.8% (-38.8 to 28.3)
Vanuatu	4.73 (3.89 to 5.67)	3.1% (-5.3 to 11.9)	0.65 (0.20 to 1.32)	-11.0% (-46.4 to 41.5)	0.42 (0.28 to 0.61)	2.6% (-10.2 to 17.2)	16.97 (5.23 to 34.33)	-15.8% (-50.6 to 35.4)
High-income Asia Pacific	16.40 (13.98 to 18.87)	-12.5% (-17.1 to -7.2)	0.10 (0.08 to 0.12)	-51.0% (-60.9 to -38.9)	1.45 (0.99 to 2.05)	-12.6% (-18.7 to -5.8)	2.86 (2.19 to 3.62)	-52.8% (-63.6 to -37.6)
Brunei	55.38 (46.95 to 65.13)	-22.8% (-29.2 to -16.0)	1.64 (1.38 to 1.95)	-24.2% (-45.6 to -1.6)	4.76 (3.15 to 6.68)	-22.5% (-34.1 to -8.3)	45.21 (37.28 to 55.41)	-24.3% (-44.4 to -1.8)
Japan	19.81 (16.85 to 22.99)	-4.9% (-10.9 to 1.9)	0.10 (0.08 to 0.13)	-51.3% (-60.8 to -39.3)	1.75 (1.20 to 2.48)	-5.3% (-12.6 to 2.9)	3.22 (2.45 to 3.97)	-51.7% (-61.8 to -38.3)
Singapore	30.33 (25.64 to 35.52)	-6.5% (-13.9 to 1.0)	0.17 (0.11 to 0.38)	-73.4% (-86.3 to -34.5)	2.65 (1.72 to 3.78)	-6.1% (-23.2 to 12.9)	5.37 (3.57 to 11.84)	-73.2% (-87.0 to -36.4)
South Korea	8.32 (6.97 to 9.83)	-13.1% (-19.7 to -5.7)	0.09 (0.07 to 0.11)	-16.3% (-44.5 to 26.8)	0.75 (0.47 to 1.06)	-12.6% (-29.0 to 7.3)	1.80 (1.35 to 2.35)	-30.5% (-52.8 to 11.1)
High-income North America	46.64 (40.10 to 53.91)	-7.7% (-16.0 to 2.4)	1.08 (0.87 to 1.28)	-43.8% (-60.7 to -29.2)	3.94 (2.73 to 5.45)	-8.2% (-16.8 to 2.2)	28.66 (23.33 to 34.61)	-47.8% (-62.7 to -26.5)
Canada	56.29 (46.26 to 66.77)	-2.8% (-12.4 to 6.9)	0.59 (0.45 to 0.73)	-28.7% (-51.2 to -13.6)	4.83 (3.20 to 6.93)	-2.9% (-18.3 to 12.7)	15.69 (12.16 to 19.31)	-30.1% (-50.6 to -13.7)
Greenland	25.14 (20.79 to 30.21)	-5.1% (-14.5 to 5.6)	1.04 (0.78 to 1.34)	-15.2% (-41.6 to 25.8)	2.17 (1.42 to 3.27)	-5.0% (-24.4 to 19.9)	29.68 (22.57 to 37.52)	-24.3% (-48.7 to 10.3)
United States	45.52 (39.11 to 52.69)	-8.4% (-17.6 to 3.2)	1.14 (0.92 to 1.34)	-44.1% (-61.1 to -29.0)	3.84 (2.65 to 5.30)	-9.0% (-18.2 to 3.1)	30.21 (24.64 to 36.36)	-48.4% (-63.3 to -26.9)
Western Europe	47.03 (39.93 to 54.89)	-21.9% (-25.9 to -17.4)	0.75 (0.58 to 0.85)	-58.5% (-71.6 to -49.2)	4.02 (2.75 to 5.62)	-21.8% (-27.2 to -15.8)	19.12 (16.02 to 21.49)	-56.3% (-65.7 to -48.4)

Andorra	41.52 (34.59 to 49.28)	-27.1% (-33.2 to -20.1)	0.58 (0.28 to 0.88)	-48.5% (-66.6 to -11.5)	3.57 (2.30 to 5.11)	-27.1% (-38.8 to -14.5)	17.02 (8.10 to 25.52)	-50.3% (-69.1 to -13.5)
Austria	36.96 (32.33 to 42.07)	2.6% (-10.2 to 19.1)	0.82 (0.45 to 1.03)	161.1% (-32.3 to 462.6)	3.14 (2.14 to 4.50)	2.5% (-15.2 to 26.1)	18.24 (9.83 to 22.26)	174.7% (-41.9 to 431.1)
Belgium	46.44 (38.40 to 54.81)	-7.1% (-18.3 to 7.3)	0.58 (0.43 to 0.72)	-38.9% (-64.3 to -19.8)	3.96 (2.62 to 5.70)	-7.2% (-23.8 to 11.1)	14.36 (10.85 to 17.66)	-30.8% (-55.0 to -12.0)
Cyprus	10.33 (8.79 to 12.14)	-18.0% (-24.7 to -9.2)	0.24 (0.16 to 0.40)	-27.4% (-53.7 to 13.2)	0.91 (0.59 to 1.31)	-17.5% (-33.1 to 0.8)	5.39 (3.93 to 8.75)	-26.9% (-52.9 to 13.4)
Denmark	32.68 (26.64 to 39.24)	-17.3% (-26.0 to -6.3)	0.43 (0.30 to 0.52)	-38.7% (-51.8 to -15.1)	2.81 (1.84 to 4.14)	-17.7% (-32.1 to 0.4)	11.91 (8.64 to 14.22)	-41.8% (-54.8 to -18.7)
Finland	94.05 (77.61 to 114.06)	-12.2% (-21.7 to 1.1)	2.61 (1.55 to 3.10)	-16.0% (-32.4 to 6.9)	8.00 (5.27 to 11.36)	-12.3% (-24.9 to 4.3)	78.18 (48.98 to 91.21)	-22.9% (-37.3 to 0.5)
France	39.73 (33.44 to 47.24)	-23.9% (-29.6 to -17.3)	0.40 (0.29 to 0.50)	-69.9% (-83.0 to -61.1)	3.42 (2.26 to 4.89)	-23.5% (-35.6 to -10.3)	10.98 (7.47 to 13.80)	-67.1% (-80.2 to -57.2)
Germany	100.01 (83.09 to 119.28)	-23.3% (-29.9 to -15.4)	2.08 (1.52 to 2.47)	-52.5% (-62.8 to -39.7)	8.48 (5.55 to 12.00)	-23.2% (-32.5 to -11.8)	50.54 (39.47 to 58.97)	-52.1% (-61.6 to -39.7)
Greece	6.31 (5.23 to 7.46)	-18.9% (-26.2 to -11.2)	0.11 (0.08 to 0.17)	-33.9% (-54.7 to 4.4)	0.56 (0.36 to 0.85)	-18.2% (-36.3 to 4.4)	3.20 (2.43 to 5.17)	-33.9% (-56.3 to -2.0)
Iceland	19.07 (16.04 to 22.68)	-22.1% (-29.2 to -13.5)	0.17 (0.14 to 0.22)	-59.2% (-71.4 to -46.0)	1.65 (1.08 to 2.36)	-21.9% (-36.1 to -2.9)	3.94 (3.05 to 5.92)	-59.3% (-74.0 to -36.1)
Ireland	43.60 (36.28 to 51.12)	-18.1% (-24.3 to -11.1)	0.76 (0.60 to 0.99)	-37.9% (-66.8 to -12.8)	3.74 (2.50 to 5.36)	-18.0% (-29.9 to -4.2)	19.31 (14.08 to 28.23)	-39.0% (-65.9 to -18.0)
Israel	7.52 (6.29 to 8.91)	-28.7% (-34.7 to -22.1)	0.06 (0.04 to 0.08)	-74.8% (-84.6 to -60.8)	0.67 (0.44 to 1.00)	-28.2% (-41.9 to -10.4)	1.72 (1.10 to 2.33)	-74.4% (-84.2 to -59.5)
Italy	38.52 (32.69 to 43.97)	-9.7% (-22.1 to 3.0)	0.10 (0.07 to 0.20)	-92.6% (-97.0 to -87.5)	3.31 (2.23 to 4.63)	-9.6% (-26.7 to 10.2)	2.87 (1.66 to 5.71)	-89.6% (-95.9 to -84.7)
Luxembourg	46.84 (38.90 to 55.56)	-15.4% (-24.2 to -3.5)	0.51 (0.39 to 0.93)	-62.5% (-77.4 to -40.6)	3.98 (2.58 to 5.69)	-15.0% (-28.3 to 3.0)	11.57 (7.61 to 23.22)	-63.1% (-80.3 to -24.4)
Malta	16.55 (13.82 to 19.67)	-14.4% (-23.5 to -2.8)	0.22 (0.17 to 0.28)	-58.5% (-74.2 to -42.8)	1.43 (0.93 to 2.09)	-14.7% (-31.2 to 5.3)	5.51 (3.99 to 7.27)	-55.1% (-71.8 to -41.0)
Netherlands	48.88 (40.56 to 58.18)	-6.5% (-15.8 to 3.1)	0.49 (0.36 to 0.71)	-78.8% (-87.8 to -66.6)	4.19 (2.77 to 5.95)	-6.5% (-20.8 to 10.4)	12.47 (7.92 to 19.56)	-76.4% (-86.6 to -58.9)
Norway	18.75 (16.12 to 21.39)	-31.0% (-38.3 to -22.0)	0.27 (0.23 to 0.35)	-57.1% (-64.2 to -35.0)	1.64 (1.11 to 2.26)	-30.3% (-38.0 to -20.8)	7.14 (6.10 to 9.78)	-59.5% (-66.3 to -37.1)
Portugal	23.15 (19.34 to 27.44)	-23.6% (-31.2 to -15.7)	0.38 (0.27 to 0.50)	-36.1% (-76.1 to -8.0)	2.01 (1.25 to 2.92)	-22.9% (-37.7 to -6.1)	10.77 (7.94 to 13.69)	-33.3% (-74.3 to -4.2)
Spain	20.66 (16.88 to 24.81)	-25.0% (-32.1 to -17.6)	0.28 (0.22 to 0.43)	-71.8% (-84.2 to -62.2)	1.80 (1.18 to 2.58)	-24.7% (-38.5 to -9.5)	7.35 (5.64 to 11.19)	-66.1% (-76.0 to -54.4)
Sweden	49.48 (39.76 to 60.22)	-18.9% (-25.5 to -12.0)	0.66 (0.42 to 0.80)	-11.8% (-38.0 to 14.9)	4.26 (2.86 to 6.13)	-18.4% (-28.8 to -6.2)	18.29 (12.23 to 22.28)	-18.9% (-41.7 to 7.7)
Switzerland	47.53 (40.93 to 55.03)	-22.4% (-31.6 to -10.6)	0.47 (0.29 to 0.58)	-71.4% (-80.0 to -51.4)	4.03 (2.75 to 5.72)	-22.7% (-35.3 to -7.4)	10.78 (7.72 to 13.48)	-73.1% (-82.3 to -50.3)
United Kingdom	24.18 (20.70 to 27.99)	-22.7% (-27.1 to -17.9)	0.51 (0.45 to 0.60)	-16.8% (-25.7 to -9.1)	2.12 (1.45 to 2.94)	-22.3% (-27.1 to -17.2)	15.55 (13.82 to 18.27)	-19.6% (-26.1 to -11.9)
Australasia	61.97 (51.89 to 72.89)	-20.1% (-25.5 to -14.2)	1.05 (0.81 to 1.31)	-42.2% (-60.2 to -29.2)	5.26 (3.52 to 7.57)	-20.1% (-29.7 to -9.5)	29.37 (22.49 to 36.42)	-42.3% (-59.5 to -28.9)
Australia	62.71 (52.35 to 74.13)	-21.0% (-27.3 to -14.2)	1.01 (0.75 to 1.28)	-42.7% (-62.1 to -27.7)	5.33 (3.50 to 7.73)	-21.0% (-32.3 to -8.6)	28.28 (20.50 to 35.77)	-42.1% (-61.0 to -26.8)
New Zealand	58.03 (48.57 to 68.97)	-15.0% (-23.3 to -6.2)	1.23 (1.02 to 1.55)	-40.3% (-55.0 to -27.6)	4.90 (3.36 to 6.95)	-15.3% (-27.5 to -1.0)	35.10 (29.56 to 43.47)	-43.0% (-55.0 to -31.6)

Data in parentheses are 95% uncertainty intervals. YLDs= years lived with disability; YLLs= years of life lost.

Supplementary Table 7. Age-standardised prevalence, death, YLD, and YLL rates of other cardiomyopathy for both sexes in 2017, and their percentage changes from 1990 to 2017, by location.

	Prevalence		Deaths		YLDs		YLLs	
	2017 age-standardised rate per 100 000 people	Percentage change in age-standardised rates, 1990–2017	2017 age-standardised rate per 100 000 people	Percentage change in age-standardised rates, 1990–2017	2017 age-standardised rate per 100 000 people	Percentage change in age-standardised rates, 1990–2017	2017 age-standardised rate per 100 000 people	Percentage change in age-standardised rates, 1990–2017
Central Sub-Saharan Africa	97.08 (77.90 to 119.62)	4.2% (-2.2 to 11.3)	5.07 (3.23 to 7.68)	-11.2% (-25.0 to 10.6)	7.91 (5.30 to 11.35)	5.0% (-4.4 to 14.8)	135.04 (86.44 to 200.36)	-17.9% (-30.4 to 1.8)
Angola	106.17 (84.71 to 129.64)	8.7% (-0.8 to 19.1)	5.54 (3.73 to 7.69)	-13.3% (-36.6 to 28.3)	8.67 (5.68 to 12.34)	9.0% (-4.0 to 23.1)	143.08 (97.78 to 195.78)	-25.5% (-44.7 to 10.5)
Central African Republic	91.95 (72.64 to 113.29)	7.8% (-1.4 to 18.5)	6.06 (3.64 to 9.53)	-11.6% (-29.6 to 16.0)	7.48 (4.89 to 10.67)	8.4% (-3.5 to 22.6)	184.01 (114.17 to 284.65)	-7.4% (-26.2 to 26.9)
Congo	104.81 (83.29 to 130.75)	4.5% (-4.7 to 15.1)	6.47 (4.41 to 8.90)	-12.7% (-32.8 to 14.7)	8.58 (5.62 to 12.41)	5.4% (-6.9 to 19.5)	163.25 (109.97 to 224.46)	-19.7% (-39.5 to 5.1)
Democratic Republic of the Congo	93.27 (74.26 to 115.55)	2.5% (-6.3 to 12.5)	4.73 (2.73 to 7.86)	-10.2% (-27.8 to 13.7)	7.59 (5.02 to 11.00)	3.3% (-9.7 to 17.4)	127.20 (73.99 to 204.70)	-15.7% (-31.2 to 5.2)
Equatorial Guinea	111.81 (89.42 to 137.81)	20.2% (8.5 to 33.1)	5.66 (3.97 to 7.83)	-15.9% (-51.0 to 56.7)	9.15 (6.02 to 13.12)	21.2% (5.9 to 39.2)	131.37 (89.28 to 189.82)	-34.2% (-61.6 to 18.6)
Gabon	115.98 (93.21 to 144.65)	0.3% (-8.6 to 10.2)	6.01 (4.54 to 7.74)	-12.1% (-32.1 to 13.5)	9.50 (6.19 to 13.61)	0.8% (-10.4 to 12.7)	150.40 (112.86 to 192.16)	-14.7% (-34.0 to 10.3)
Eastern Sub-Saharan Africa	86.30 (69.20 to 104.72)	9.8% (5.8 to 14.3)	3.51 (2.51 to 4.11)	-23.0% (-38.6 to -7.3)	7.16 (4.72 to 10.26)	10.5% (5.6 to 15.7)	102.99 (75.33 to 122.39)	-30.7% (-47.5 to -14.5)
Burundi	57.96 (45.07 to 73.34)	-0.5% (-8.9 to 10.3)	3.32 (2.28 to 4.30)	-44.8% (-56.1 to -29.9)	4.84 (3.15 to 7.04)	-0.4% (-13.2 to 14.0)	100.66 (71.33 to 131.17)	-47.5% (-58.7 to -32.8)
Comoros	78.14 (61.72 to 97.38)	4.9% (-3.8 to 14.5)	3.25 (2.29 to 4.30)	-39.7% (-54.5 to -21.6)	6.50 (4.20 to 9.32)	5.6% (-7.0 to 20.3)	91.97 (65.35 to 123.52)	-48.1% (-60.8 to -33.6)
Djibouti	81.54 (64.06 to 101.11)	11.5% (1.1 to 21.9)	3.95 (2.57 to 5.61)	-28.2% (-50.4 to 3.6)	6.83 (4.49 to 9.92)	11.5% (-2.3 to 26.4)	108.03 (68.65 to 160.25)	-35.1% (-55.6 to -5.9)
Eritrea	67.86 (52.81 to 85.81)	5.2% (-3.9 to 15.4)	4.98 (3.10 to 6.74)	-32.6% (-50.8 to -8.2)	5.60 (3.60 to 8.27)	6.3% (-6.4 to 20.9)	148.50 (88.76 to 203.29)	-36.6% (-56.5 to -8.0)
Ethiopia	145.40 (117.47 to 175.16)	5.1% (-2.0 to 12.5)	2.66 (1.89 to 3.32)	-38.6% (-58.8 to -11.4)	12.03 (7.84 to 17.13)	6.8% (-1.4 to 15.5)	76.01 (55.38 to 95.62)	-47.3% (-65.2 to -23.4)
Kenya	54.41 (42.96 to 67.78)	9.6% (6.7 to 12.2)	3.62 (2.36 to 4.47)	-6.9% (-20.5 to 9.7)	4.55 (3.01 to 6.54)	9.4% (5.7 to 12.7)	98.22 (64.01 to 115.63)	-11.7% (-25.3 to 4.9)
Madagascar	69.89 (54.61 to 87.14)	3.7% (-5.4 to 13.6)	6.18 (4.23 to 8.24)	-15.7% (-32.2 to 4.0)	5.84 (3.76 to 8.66)	3.9% (-9.5 to 17.8)	193.81 (134.29 to 258.43)	-24.8% (-40.1 to -7.2)
Malawi	71.96 (55.82 to 90.29)	16.0% (5.3 to 27.5)	2.79 (2.15 to 3.49)	-14.7% (-36.4 to 34.5)	5.98 (3.80 to 8.73)	16.7% (1.6 to 33.1)	83.72 (63.95 to 107.02)	-29.8% (-50.1 to 21.3)
Mozambique	41.53 (32.14 to 52.71)	12.3% (2.3 to 23.3)	3.93 (2.97 to 5.13)	1.7% (-25.8 to 49.1)	3.45 (2.19 to 5.13)	12.5% (-3.8 to 32.9)	117.10 (88.05 to 150.80)	-20.0% (-42.2 to 20.5)
Rwanda	65.51 (51.23 to 82.00)	0.6% (-7.7 to 9.7)	3.37 (2.30 to 4.40)	-44.4% (-58.1 to -27.8)	5.47 (3.51 to 7.95)	1.1% (-11.0 to 14.8)	89.40 (60.74 to 116.82)	-49.7% (-63.3 to -33.5)
Somalia	69.73 (54.67 to 87.19)	8.1% (-0.7 to 17.3)	3.80 (2.56 to 5.41)	-32.2% (-52.3 to 6.4)	5.80 (3.87 to 8.51)	8.5% (-4.7 to 23.7)	116.33 (73.77 to 173.12)	-38.7% (-59.6 to 9.6)
South Sudan	72.35 (56.32 to 90.39)	6.6% (-3.0 to 17.1)	3.78 (2.67 to 5.13)	-30.2% (-48.6 to 1.8)	5.91 (3.86 to 8.63)	7.1% (-6.2 to 23.0)	121.57 (84.06 to 168.33)	-27.1% (-47.3 to 11.5)
Tanzania	76.10 (59.54 to 96.09)	13.9% (3.8 to 25.5)	3.65 (2.39 to 4.78)	-18.6% (-38.9 to 8.6)	6.35 (4.11 to 9.25)	14.4% (1.0 to 31.1)	108.28 (73.18 to 146.94)	-22.2% (-43.2 to 10.7)
Uganda	70.38 (55.27 to 87.98)	-1.2% (-9.6 to 8.2)	3.23 (2.08 to 4.18)	-3.6% (-32.5 to 40.2)	5.83 (3.81 to 8.53)	0.1% (-12.5 to 14.6)	91.64 (59.09 to 120.56)	-12.5% (-38.3 to 24.9)
Zambia	70.60 (54.85 to 88.33)	21.3% (11.3 to 33.6)	3.51 (2.32 to 4.46)	-18.2% (-40.5 to 9.8)	5.87 (3.81 to 8.53)	21.2% (6.1 to 39.3)	99.87 (67.28 to 128.01)	-28.9% (-49.4 to -1.2)
Southern Sub-Saharan Africa	150.35 (120.52 to 184.03)	9.8% (5.8 to 14.0)	7.38 (6.97 to 7.85)	-3.3% (-14.6 to 9.3)	12.27 (8.05 to 17.32)	9.5% (5.0 to 14.3)	155.88 (146.95 to 166.35)	-13.3% (-22.2 to -3.3)
Botswana	157.56 (126.37 to 194.08)	7.5% (-2.6 to 18.0)	6.97 (5.76 to 8.21)	-11.6% (-32.4 to 14.1)	12.79 (8.25 to 18.38)	7.3% (-5.4 to 20.3)	127.54 (104.39 to 150.86)	-20.8% (-39.4 to 3.4)
Lesotho	126.46 (100.43 to 156.30)	6.0% (-4.5 to 17.9)	9.77 (7.46 to 12.39)	8.1% (-22.8 to 46.9)	10.19 (6.70 to 14.73)	4.9% (-7.2 to 19.4)	205.98 (160.72 to 258.52)	9.7% (-20.9 to 52.5)
Namibia	143.66 (114.71 to 175.96)	8.1% (-2.0 to 19.4)	6.89 (5.04 to 8.51)	-26.9% (-46.6 to 5.7)	11.69 (7.64 to 16.49)	8.4% (-3.6 to 21.5)	137.41 (98.29 to 170.63)	-29.6% (-48.9 to 2.4)
South Africa	160.50 (128.99 to 197.34)	9.7% (5.0 to 14.6)	6.97 (6.58 to 7.45)	-4.4% (-14.2 to 7.3)	13.11 (8.60 to 18.55)	9.5% (4.1 to 14.9)	146.00 (136.94 to 156.71)	-20.2% (-26.8 to -11.7)
Swaziland	128.80 (102.04 to 159.37)	4.9% (-4.5 to 15.3)	8.51 (6.73 to 10.77)	-19.4% (-39.6 to 8.3)	10.38 (6.85 to 14.89)	4.3% (-8.2 to 17.1)	186.03 (147.12 to 238.61)	-16.8% (-37.2 to 7.7)
Zimbabwe	85.67 (67.91 to 107.64)	-7.3% (-17.9 to 5.4)	9.86 (8.08 to 12.07)	8.7% (-15.4 to 39.5)	7.00 (4.50 to 10.28)	-7.6% (-20.0 to 7.4)	215.20 (172.17 to 261.09)	20.1% (-7.1 to 53.4)
Western Sub-Saharan Africa	97.93 (79.56 to 119.73)	11.0% (5.2 to 16.5)	3.39 (2.72 to 4.13)	-24.2% (-38.9 to -5.2)	8.07 (5.37 to 11.61)	11.5% (4.2 to 19.4)	79.85 (63.82 to 96.59)	-30.5% (-42.4 to -13.3)
Benin	88.72 (70.81 to 110.55)	5.2% (-4.4 to 15.1)	3.17 (2.02 to 4.62)	-28.4% (-47.4 to -4.3)	7.33 (4.80 to 10.60)	6.3% (-6.9 to 19.8)	75.78 (48.51 to 110.62)	-32.9% (-49.3 to -12.3)
Burkina Faso	83.23 (65.55 to 103.20)	5.6% (-4.2 to 17.0)	3.66 (2.51 to 4.97)	-12.7% (-35.0 to 16.3)	6.88 (4.51 to 10.13)	7.6% (-5.0 to 22.4)	88.15 (61.22 to 119.44)	-20.5% (-39.1 to 3.1)
Cameroon	93.11 (74.22 to 114.82)	1.4% (-13.9 to 17.4)	4.03 (3.03 to 5.17)	-28.7% (-47.7 to -2.7)	7.69 (4.99 to 11.09)	2.6% (-15.2 to 22.0)	97.11 (71.47 to 126.67)	-30.9% (-49.4 to -8.3)
Cape Verde	114.90 (91.04 to 141.78)	11.5% (1.1 to 22.4)	1.27 (1.11 to 1.46)	19.4% (-12.4 to 69.6)	9.63 (6.16 to 13.94)	11.8% (-1.1 to 26.2)	31.33 (27.09 to 36.41)	-0.5% (-24.3 to 34.4)
Chad	85.97 (67.55 to 107.22)	6.1% (-3.7 to 16.1)	2.96 (1.84 to 4.50)	-23.0% (-40.4 to 2.5)	7.05 (4.53 to 10.12)	6.5% (-6.7 to 20.9)	74.26 (46.91 to 111.32)	-26.9% (-42.8 to -4.0)
Cote d'Ivoire	96.51 (76.64 to 121.53)	2.6% (-6.1 to 12.7)	3.66 (2.42 to 4.87)	-16.6% (-37.1 to 15.9)	7.94 (5.21 to 11.49)	4.2% (-8.3 to 18.0)	91.07 (60.39 to 122.45)	-20.1% (-40.1 to 8.1)
The Gambia	100.94 (79.87 to 126.23)	6.7% (-2.6 to 17.0)	3.43 (2.31 to 4.62)	-18.5% (-38.3 to 13.3)	8.32 (5.44 to 12.07)	6.9% (-4.5 to 21.8)	82.20 (55.21 to 112.57)	-21.3% (-40.0 to 8.9)
Ghana	90.85 (71.17 to 113.79)	6.7% (-4.7 to 19.3)	7.27 (6.14 to 8.49)	99.4% (47.9 to 156.8)	7.55 (4.96 to 10.91)	7.2% (-6.6 to 23.8)	166.75 (140.45 to 194.24)	78.7% (34.5 to 128.8)
Guinea	58.44 (45.53 to 74.00)	15.3% (2.7 to 28.9)	3.34 (2.12 to 4.93)	-26.0% (-41.7 to 1.7)	4.86 (3.18 to 7.10)	15.9% (-1.3 to 34.2)	84.22 (53.39 to 122.79)	-33.6% (-46.9 to -11.3)
Guinea-Bissau	89.44 (69.97 to 110.17)	11.4% (1.3 to 22.1)	3.91 (2.77 to 5.62)	-35.5% (-52.1 to -14.5)	7.37 (4.83 to 10.62)	12.2% (-0.6 to 26.3)	99.99 (70.81 to 144.20)	-38.8% (-54.8 to -19.0)
Liberia	94.48 (75.37 to 118.41)	11.0% (1.1 to 22.0)	2.93 (1.71 to 4.20)	-34.0% (-48.4 to -14.8)	7.67 (4.97 to 11.03)	11.2% (-1.4 to 25.4)	69.92 (41.66 to 100.01)	-45.3% (-57.5 to -29.6)
Mali	85.20 (67.36 to 106.11)	5.6% (-4.0 to 16.1)	3.40 (1.90 to 5.49)	-34.1% (-47.4 to -17.1)	6.99 (4.53 to 10.16)	6.8% (-6.4 to 21.4)	84.23 (48.68 to 130.19)	-42.0% (-54.1 to -26.7)

Mauritania	99.11 (78.63 to 125.35)	-0.5% (-9.0 to 9.3)	3.29 (2.11 to 4.78)	-35.8% (-49.7 to -15.6)	8.18 (5.42 to 11.95)	0.3% (-11.4 to 13.4)	72.61 (45.90 to 105.88)	-43.1% (-55.1 to -24.6)
Niger	86.00 (68.40 to 107.58)	7.3% (-1.1 to 17.3)	2.54 (1.37 to 4.21)	-37.4% (-50.0 to -18.4)	7.11 (4.66 to 10.25)	7.6% (-5.2 to 21.8)	61.71 (33.35 to 100.06)	-47.0% (-57.9 to -32.3)
Nigeria	105.77 (84.48 to 130.55)	15.3% (4.8 to 26.2)	2.82 (1.93 to 4.00)	-38.6% (-56.3 to -10.2)	8.71 (5.72 to 12.70)	15.4% (2.3 to 30.4)	63.60 (44.40 to 92.14)	-44.3% (-59.9 to -18.3)
Sao Tome and Principe	97.63 (76.98 to 121.52)	14.7% (5.3 to 25.8)	3.20 (2.52 to 4.03)	-3.3% (-28.7 to 36.9)	8.11 (5.28 to 11.60)	15.0% (1.8 to 31.1)	80.69 (62.94 to 104.18)	-18.4% (-38.8 to 12.5)
Senegal	122.88 (98.24 to 152.14)	6.7% (-3.7 to 17.5)	2.52 (1.53 to 3.66)	-27.0% (-41.5 to -5.8)	10.09 (6.67 to 14.47)	7.1% (-5.0 to 20.2)	58.69 (35.92 to 84.28)	-33.1% (-46.6 to -14.3)
Sierra Leone	90.87 (71.76 to 112.56)	12.9% (3.4 to 23.8)	3.24 (2.35 to 4.24)	-20.6% (-40.4 to 12.4)	7.46 (4.88 to 11.01)	12.7% (-0.3 to 27.0)	82.83 (60.66 to 107.51)	-30.4% (-46.1 to -4.5)
Togo	93.99 (73.39 to 116.87)	6.6% (-1.9 to 16.1)	2.92 (2.07 to 3.94)	-32.8% (-47.9 to -11.0)	7.77 (5.07 to 11.41)	7.3% (-4.8 to 20.9)	70.21 (49.97 to 94.67)	-36.3% (-50.3 to -17.3)
Andean Latin America	26.96 (22.50 to 32.06)	21.2% (14.2 to 28.4)	1.06 (0.97 to 1.18)	-25.8% (-35.9 to -13.6)	2.31 (1.52 to 3.28)	21.1% (9.3 to 36.0)	33.33 (29.89 to 37.50)	-45.7% (-55.3 to -32.8)
Bolivia	17.38 (12.66 to 22.75)	73.8% (47.8 to 114.4)	1.86 (1.50 to 2.27)	-22.1% (-38.7 to 1.2)	1.46 (0.88 to 2.20)	76.3% (40.0 to 131.2)	54.76 (42.81 to 68.09)	-43.4% (-58.0 to -20.3)
Ecuador	25.38 (21.03 to 30.46)	13.9% (4.7 to 23.5)	1.48 (1.30 to 1.65)	63.6% (20.5 to 117.4)	2.18 (1.43 to 3.14)	14.1% (-1.8 to 32.5)	43.38 (37.48 to 49.89)	46.0% (6.2 to 97.8)
Peru	30.62 (25.66 to 36.13)	19.7% (11.1 to 29.4)	0.67 (0.56 to 0.81)	-52.9% (-61.3 to -40.5)	2.63 (1.71 to 3.77)	19.6% (3.7 to 39.6)	21.41 (17.58 to 26.31)	-66.6% (-74.3 to -54.8)
Tropical Latin America	106.37 (87.53 to 126.69)	8.6% (3.9 to 14.5)	7.54 (7.18 to 8.50)	-16.8% (-22.6 to 0.4)	8.86 (5.79 to 12.68)	9.4% (3.9 to 16.1)	178.30 (168.12 to 195.61)	-18.0% (-23.4 to -3.8)
Brazil	107.74 (88.61 to 128.33)	8.3% (3.6 to 14.3)	7.68 (7.31 to 8.66)	-17.2% (-23.0 to 0.0)	8.98 (5.87 to 12.84)	9.1% (3.7 to 15.9)	182.22 (171.78 to 200.00)	-18.0% (-23.5 to -3.9)
Paraguay	48.44 (40.43 to 58.37)	14.7% (5.8 to 24.7)	1.72 (1.43 to 2.06)	34.0% (4.9 to 72.7)	4.07 (2.69 to 5.80)	15.2% (1.7 to 32.9)	38.17 (31.96 to 45.49)	24.3% (-2.4 to 62.1)
Central Latin America	36.20 (30.67 to 42.36)	12.2% (8.5 to 16.5)	1.48 (1.32 to 1.58)	-5.3% (-12.0 to 1.9)	3.11 (2.08 to 4.35)	12.3% (6.3 to 18.7)	42.62 (40.23 to 45.38)	-2.3% (-8.6 to 4.5)
Colombia	49.19 (41.63 to 57.31)	6.3% (-0.7 to 14.4)	1.72 (1.50 to 1.92)	-11.8% (-22.4 to 4.2)	4.22 (2.77 to 5.94)	7.1% (-7.1 to 23.4)	47.66 (41.04 to 59.94)	-19.4% (-31.4 to 1.9)
Costa Rica	85.64 (72.62 to 99.57)	4.5% (-2.9 to 12.2)	3.59 (2.96 to 4.07)	-11.5% (-25.2 to 7.4)	7.30 (4.87 to 10.40)	4.9% (-6.7 to 18.7)	88.47 (77.28 to 101.11)	-10.7% (-23.3 to 4.2)
El Salvador	14.44 (12.12 to 17.05)	16.6% (7.9 to 25.2)	0.64 (0.49 to 0.88)	-4.1% (-27.2 to 35.0)	1.25 (0.82 to 1.76)	15.9% (-1.4 to 36.2)	17.74 (13.60 to 25.26)	-14.5% (-36.0 to 21.0)
Guatemala	17.03 (14.32 to 20.15)	11.9% (4.4 to 21.0)	0.92 (0.71 to 1.06)	48.6% (4.7 to 104.3)	1.47 (0.96 to 2.12)	12.5% (-2.8 to 31.2)	28.05 (23.34 to 32.57)	53.0% (0.4 to 94.2)
Honduras	38.07 (31.84 to 45.38)	20.8% (11.8 to 30.8)	2.87 (2.29 to 3.52)	-6.5% (-28.2 to 19.0)	3.22 (2.13 to 4.64)	20.4% (3.8 to 42.1)	72.32 (58.08 to 88.52)	-22.4% (-40.4 to -0.7)
Mexico	27.73 (23.21 to 32.81)	12.2% (9.5 to 15.9)	1.05 (0.96 to 1.12)	22.1% (-0.3 to 32.5)	2.41 (1.60 to 3.40)	12.0% (7.8 to 16.9)	37.38 (32.71 to 39.60)	38.4% (5.8 to 52.6)
Nicaragua	38.25 (32.79 to 44.52)	24.8% (16.1 to 33.4)	1.38 (1.19 to 1.57)	-12.8% (-25.9 to 3.1)	3.28 (2.15 to 4.64)	25.2% (8.4 to 43.2)	36.27 (31.64 to 41.54)	-26.2% (-38.0 to -12.9)
Panama	83.13 (70.07 to 97.96)	2.6% (-4.9 to 10.3)	3.97 (2.81 to 4.46)	76.9% (30.6 to 104.5)	7.06 (4.72 to 9.99)	2.7% (-9.1 to 16.7)	110.51 (84.89 to 124.17)	74.8% (25.9 to 105.0)
Venezuela	43.42 (35.74 to 52.28)	14.4% (3.9 to 27.6)	2.24 (1.72 to 2.70)	-42.0% (-54.5 to -24.1)	3.68 (2.42 to 5.23)	15.2% (-1.2 to 35.6)	46.32 (38.95 to 54.88)	-44.6% (-56.7 to -24.5)
Southern Latin America	82.27 (66.31 to 100.42)	-12.6% (-19.2 to -5.2)	5.68 (5.16 to 6.58)	-23.6% (-33.4 to -6.1)	6.85 (4.45 to 9.83)	-12.1% (-19.8 to -3.0)	119.98 (109.05 to 143.64)	-30.3% (-38.6 to -12.6)
Argentina	83.54 (66.76 to 102.94)	-6.6% (-16.7 to 4.8)	6.93 (6.18 to 7.95)	-21.1% (-32.8 to -4.0)	6.94 (4.50 to 10.13)	-6.1% (-17.8 to 8.4)	142.28 (127.01 to 170.22)	-30.5% (-40.3 to -13.9)
Chile	75.61 (61.19 to 91.62)	-21.1% (-27.9 to -14.0)	3.15 (2.75 to 3.94)	0.5% (-13.4 to 21.8)	6.35 (4.14 to 8.99)	-20.3% (-29.3 to -9.7)	74.18 (65.27 to 88.45)	-5.9% (-19.7 to 17.6)
Uruguay	96.89 (79.67 to 117.05)	-23.1% (-29.1 to -16.7)	3.90 (3.35 to 5.31)	-47.9% (-57.5 to -11.7)	8.08 (5.29 to 11.67)	-22.7% (-31.0 to -13.6)	82.66 (70.32 to 114.62)	-52.2% (-61.2 to -16.5)
Caribbean	50.19 (43.16 to 58.38)	5.0% (0.9 to 9.3)	3.20 (2.83 to 3.58)	0.4% (-7.5 to 8.9)	4.26 (2.83 to 6.01)	4.7% (-1.9 to 11.0)	95.43 (82.66 to 110.28)	-7.8% (-18.1 to 4.8)
Antigua and Barbuda	52.56 (44.12 to 61.59)	2.1% (-5.9 to 10.2)	4.11 (3.43 to 4.62)	20.9% (1.9 to 44.6)	4.45 (2.94 to 6.24)	1.6% (-10.4 to 15.5)	97.55 (82.59 to 109.52)	14.3% (-2.7 to 33.1)
The Bahamas	54.47 (45.69 to 64.03)	-3.9% (-11.0 to 3.1)	6.91 (6.15 to 7.79)	2.5% (-13.7 to 24.6)	4.64 (3.08 to 6.61)	-3.9% (-15.2 to 8.7)	187.91 (164.91 to 211.90)	-2.7% (-19.9 to 22.9)
Barbados	79.63 (67.73 to 93.08)	-2.6% (-9.3 to 4.8)	4.71 (4.14 to 5.32)	7.7% (-5.7 to 24.3)	6.75 (4.45 to 9.61)	-2.8% (-13.7 to 9.1)	113.71 (98.78 to 128.46)	1.0% (-11.7 to 17.6)
Belize	68.35 (58.30 to 79.87)	5.6% (-2.3 to 12.6)	5.36 (4.74 to 5.93)	35.4% (10.4 to 66.3)	5.79 (3.81 to 8.24)	5.4% (-6.8 to 18.9)	142.79 (127.04 to 157.20)	27.3% (5.0 to 55.8)
Bermuda	103.60 (88.29 to 120.36)	-6.4% (-12.7 to 0.4)	4.09 (3.24 to 4.64)	-15.3% (-38.4 to 7.1)	8.79 (5.82 to 12.35)	-6.2% (-15.9 to 4.8)	86.77 (73.61 to 99.48)	-25.5% (-48.3 to -2.3)
Cuba	55.74 (48.03 to 64.75)	12.1% (3.4 to 20.9)	2.32 (1.94 to 2.65)	31.0% (-4.9 to 55.8)	4.75 (3.16 to 6.67)	11.9% (-1.0 to 26.6)	53.42 (45.89 to 61.22)	0.7% (-17.7 to 19.8)
Dominica	113.49 (95.77 to 133.18)	-2.2% (-8.6 to 5.3)	17.56 (15.49 to 19.31)	42.2% (13.7 to 70.3)	9.58 (6.41 to 13.55)	-1.9% (-12.1 to 8.5)	445.68 (394.15 to 489.88)	43.1% (14.6 to 67.8)
Dominican Republic	32.16 (27.41 to 37.52)	14.0% (6.0 to 21.9)	1.99 (1.70 to 2.29)	33.3% (6.0 to 62.0)	2.76 (1.81 to 3.97)	13.4% (-2.5 to 30.3)	57.14 (48.77 to 65.96)	5.6% (-13.1 to 27.9)
Grenada	78.40 (66.15 to 91.78)	-1.2% (-8.8 to 7.4)	6.63 (5.84 to 7.56)	-19.6% (-31.7 to -7.3)	6.61 (4.43 to 9.33)	-1.1% (-12.7 to 12.1)	160.67 (143.76 to 180.79)	-22.2% (-32.4 to -11.4)
Guyana	45.14 (37.83 to 53.29)	0.3% (-7.1 to 7.7)	6.45 (5.48 to 7.36)	22.7% (-4.5 to 52.4)	3.81 (2.52 to 5.38)	0.1% (-12.3 to 14.5)	174.58 (147.75 to 200.84)	14.2% (-9.5 to 42.1)
Haiti	35.64 (30.09 to 42.15)	7.1% (-0.4 to 15.3)	6.08 (4.21 to 8.56)	-2.9% (-23.1 to 22.7)	3.01 (1.98 to 4.25)	7.1% (-6.9 to 22.9)	178.83 (130.48 to 247.60)	-16.7% (-34.3 to 9.2)
Jamaica	58.50 (50.17 to 68.30)	10.2% (2.7 to 17.7)	3.34 (2.63 to 4.13)	8.3% (-15.3 to 38.2)	4.99 (3.32 to 6.92)	9.7% (-4.4 to 24.9)	94.15 (72.74 to 118.05)	10.1% (-15.1 to 43.4)
Puerto Rico	68.35 (57.98 to 80.37)	3.3% (-4.6 to 11.7)	1.80 (1.60 to 2.29)	-56.8% (-64.8 to -16.5)	5.79 (3.84 to 8.19)	3.0% (-7.9 to 15.4)	44.63 (39.21 to 62.92)	-55.6% (-63.8 to -16.4)
Saint Lucia	75.62 (64.47 to 87.79)	-2.1% (-8.5 to 5.4)	6.84 (6.02 to 7.67)	2.9% (-13.3 to 17.9)	6.40 (4.31 to 8.94)	-1.6% (-12.1 to 11.3)	166.00 (144.96 to 186.45)	0.6% (-16.6 to 14.8)
Saint Vincent and the Grenadines	29.71 (24.90 to 35.17)	1.0% (-6.7 to 8.7)	2.92 (2.43 to 3.30)	42.1% (-6.1 to 79.0)	2.53 (1.69 to 3.63)	1.1% (-11.7 to 16.2)	78.02 (64.77 to 87.70)	48.2% (1.0 to 86.7)
Suriname	57.10 (48.07 to 66.98)	3.8% (-3.5 to 12.1)	4.94 (4.33 to 5.62)	18.2% (1.3 to 39.0)	4.82 (3.18 to 6.83)	3.3% (-10.0 to 17.9)	137.49 (121.08 to 155.78)	3.8% (-10.8 to 21.9)
Trinidad and Tobago	53.17 (45.36 to 61.86)	-0.8% (-7.5 to 6.9)	3.65 (2.97 to 4.40)	-9.0% (-28.6 to 14.1)	4.50 (3.01 to 6.33)	-0.8% (-12.4 to 12.6)	107.88 (85.65 to 131.62)	-6.2% (-25.9 to 17.9)
Virgin Islands, U.S.	82.96 (70.87 to 97.52)	8.0% (0.3 to 16.7)	7.29 (5.97 to 8.58)	17.9% (-5.2 to 47.5)	7.03 (4.71 to 9.81)	7.9% (-3.9 to 20.5)	164.11 (136.10 to 193.61)	9.5% (-11.8 to 36.1)
Central Europe	146.65 (124.35 to 172.90)	6.0% (0.4 to 11.1)	10.40 (9.57 to 11.04)	-24.5% (-31.3 to -10.5)	11.91 (7.98 to 16.55)	6.3% (0.0 to 12.0)	156.54 (142.29 to 166.96)	-24.7% (-30.2 to -14.0)
Albania	90.44 (76.02 to 106.47)	3.1% (-4.6 to 11.5)	4.21 (3.34 to 5.53)	-32.5% (-46.4 to -14.4)	7.48 (5.04 to 10.50)	3.0% (-8.3 to 15.6)	89.05 (73.09 to 111.17)	-29.0% (-43.7 to -12.1)
Bosnia and Herzegovina	122.20 (102.94 to 143.86)	-2.4% (-10.0 to 6.3)	8.40 (6.67 to 10.34)	60.4% (27.1 to 111.8)	9.96 (6.73 to 13.98)	-2.7% (-12.4 to 7.8)	131.09 (101.70 to 166.29)	39.7% (15.2 to 77.3)
Bulgaria	28.66 (24.41 to 33.70)	-8.3% (-14.3 to -1.8)	2.66 (2.23 to 3.40)	183.6% (9.2 to 403.0)	2.42 (1.61 to 3.41)	-8.1% (-20.3 to 5.8)	64.40 (54.88 to 72.56)	154.7% (7.0 to 269.4)
Croatia	51.25 (44.79 to 58.46)	-25.5% (-32.6 to -17.9)	3.35 (2.53 to 5.33)	-28.1% (-44.5 to 5.6)	4.29 (2.87 to 5.93)	-24.7% (-34.4 to -13.2)	43.93 (36.12 to 69.64)	-44.8% (-55.8 to -12.2)

Czech Republic	98.64 (83.72 to 116.24)	-2.4% (-11.2 to 8.4)	3.12 (2.79 to 4.07)	-31.4% (-40.3 to -12.2)	8.17 (5.51 to 11.63)	-2.3% (-14.4 to 12.8)	68.07 (61.59 to 78.78)	-45.7% (-52.9 to -26.2)
Hungary	77.16 (64.24 to 92.52)	2.3% (-7.0 to 11.8)	3.43 (2.74 to 5.90)	-76.2% (-82.1 to -44.7)	6.28 (4.21 to 9.09)	3.8% (-8.4 to 16.1)	53.59 (42.59 to 92.27)	-76.3% (-82.4 to -42.7)
Macedonia	121.04 (102.46 to 143.17)	-14.1% (-21.0 to -7.3)	10.75 (6.96 to 14.32)	-2.0% (-20.3 to 29.7)	10.00 (6.66 to 14.06)	-13.8% (-22.3 to -4.2)	201.32 (131.50 to 275.62)	5.2% (-12.5 to 23.9)
Montenegro	124.02 (105.70 to 145.47)	2.0% (-5.2 to 9.8)	7.74 (6.21 to 9.73)	39.7% (5.3 to 85.7)	10.10 (6.89 to 13.99)	1.5% (-8.0 to 12.2)	113.90 (92.01 to 140.63)	9.7% (-9.3 to 35.0)
Poland	241.35 (200.92 to 289.79)	11.8% (2.7 to 21.8)	14.02 (12.24 to 15.46)	-20.9% (-31.7 to -6.6)	19.46 (12.87 to 27.33)	12.3% (0.9 to 23.8)	185.21 (166.97 to 206.47)	-29.8% (-38.7 to -16.1)
Romania	129.22 (105.12 to 156.02)	1.6% (-7.4 to 12.2)	16.13 (13.98 to 17.54)	-27.9% (-37.7 to -15.3)	10.51 (6.92 to 15.17)	2.1% (-8.6 to 13.0)	270.06 (228.45 to 299.74)	-3.5% (-17.0 to 16.0)
Serbia	103.95 (89.14 to 121.55)	-28.8% (-36.2 to -20.4)	15.35 (10.94 to 18.92)	36.2% (6.2 to 93.1)	8.56 (5.78 to 11.84)	-28.3% (-36.9 to -17.9)	211.66 (156.68 to 255.84)	6.0% (-10.2 to 26.5)
Slovakia	90.69 (78.51 to 104.14)	16.1% (7.0 to 27.0)	2.99 (2.65 to 3.62)	4.9% (-14.7 to 24.0)	7.61 (5.14 to 10.57)	15.7% (0.9 to 32.6)	83.23 (71.12 to 93.63)	-15.9% (-31.6 to -1.5)
Slovenia	280.28 (236.17 to 330.55)	-8.4% (-17.9 to 3.5)	10.82 (9.43 to 12.65)	-79.6% (-84.2 to -42.2)	22.48 (15.18 to 31.08)	-8.2% (-18.0 to 3.8)	125.81 (110.42 to 154.77)	-80.2% (-84.4 to -37.9)
Eastern Europe	29.95 (25.82 to 34.39)	-0.7% (-2.8 to 1.5)	7.77 (5.08 to 8.69)	166.7% (42.8 to 237.3)	2.55 (1.72 to 3.49)	-0.4% (-3.6 to 3.1)	223.05 (135.21 to 256.60)	181.1% (35.6 to 271.7)
Belarus	37.62 (32.36 to 43.22)	9.7% (3.6 to 16.4)	2.44 (1.42 to 3.85)	-31.6% (-53.0 to 36.0)	3.23 (2.10 to 4.59)	9.5% (-6.2 to 28.2)	89.17 (50.50 to 126.45)	-24.1% (-47.6 to 41.6)
Estonia	96.41 (82.15 to 111.92)	16.1% (8.3 to 24.4)	5.25 (3.96 to 6.78)	-41.0% (-58.7 to 26.9)	8.19 (5.48 to 11.49)	16.1% (2.1 to 32.9)	159.94 (118.91 to 206.12)	-48.4% (-64.8 to 13.0)
Latvia	103.21 (88.75 to 119.99)	12.2% (3.8 to 20.8)	8.96 (6.79 to 12.25)	-31.8% (-46.2 to 15.8)	8.68 (5.75 to 12.17)	12.1% (-0.6 to 26.3)	262.87 (190.41 to 381.01)	-46.3% (-58.4 to 2.9)
Lithuania	65.02 (56.02 to 74.72)	15.6% (8.1 to 24.1)	4.85 (3.25 to 5.79)	16.0% (-6.6 to 44.9)	5.51 (3.67 to 7.69)	15.3% (0.0 to 33.5)	153.67 (104.15 to 185.25)	6.5% (-13.9 to 34.3)
Moldova	49.96 (42.61 to 57.92)	30.9% (23.0 to 40.2)	2.31 (1.87 to 3.53)	-23.0% (-53.4 to 8.0)	4.30 (2.86 to 6.11)	30.2% (11.0 to 52.8)	77.58 (63.10 to 118.08)	-29.8% (-54.9 to 4.4)
Russian Federation	26.35 (22.60 to 30.57)	-5.7% (-7.5 to -3.9)	8.59 (4.81 to 9.79)	251.6% (31.1 to 347.8)	2.24 (1.51 to 3.06)	-5.1% (-7.5 to -2.7)	250.11 (123.42 to 296.46)	310.3% (17.0 to 474.7)
Ukraine	30.45 (26.07 to 35.42)	5.7% (-1.0 to 13.0)	7.16 (6.14 to 8.31)	115.2% (66.7 to 173.9)	2.59 (1.72 to 3.62)	5.6% (-5.8 to 19.0)	182.03 (148.75 to 245.09)	107.0% (52.4 to 167.2)
North Africa and Middle East	35.63 (30.79 to 40.93)	11.1% (8.5 to 13.6)	1.87 (1.72 to 2.10)	-10.4% (-25.2 to 13.7)	3.04 (2.07 to 4.20)	11.1% (6.0 to 16.2)	57.16 (52.18 to 64.61)	-25.4% (-37.7 to -5.0)
Afghanistan	13.68 (11.62 to 15.98)	-0.7% (-7.1 to 6.4)	1.65 (1.06 to 2.25)	-3.2% (-28.5 to 71.4)	1.18 (0.79 to 1.66)	-0.5% (-11.9 to 12.6)	45.75 (29.50 to 63.63)	-9.2% (-33.4 to 89.7)
Algeria	35.82 (30.34 to 41.51)	9.2% (1.4 to 17.2)	1.92 (1.47 to 2.42)	-7.4% (-28.5 to 22.2)	3.04 (2.02 to 4.32)	9.4% (-5.7 to 26.4)	51.45 (40.42 to 64.38)	-22.9% (-39.1 to 0.1)
Bahrain	86.46 (74.11 to 101.72)	-4.6% (-10.9 to 2.5)	2.70 (2.38 to 3.08)	-35.0% (-44.6 to -22.9)	7.25 (4.84 to 10.13)	-3.7% (-15.0 to 7.9)	59.43 (52.40 to 70.03)	-45.7% (-53.4 to -35.9)
Egypt	31.04 (26.47 to 36.14)	31.1% (22.9 to 40.1)	2.25 (1.76 to 3.34)	-6.7% (-30.6 to 25.8)	2.63 (1.74 to 3.69)	29.5% (11.8 to 50.9)	62.84 (47.31 to 90.23)	-29.2% (-47.9 to -1.5)
Iran	35.79 (30.31 to 41.79)	8.4% (6.1 to 10.8)	2.10 (1.98 to 2.36)	7.9% (-7.7 to 41.8)	3.05 (2.07 to 4.27)	8.1% (3.2 to 13.2)	55.68 (51.79 to 62.52)	-13.9% (-26.9 to 16.2)
Iraq	53.27 (45.52 to 62.27)	12.2% (4.8 to 19.8)	2.53 (2.27 to 3.02)	-41.9% (-53.1 to -27.0)	4.45 (2.97 to 6.19)	12.3% (-2.9 to 28.6)	96.78 (84.50 to 112.82)	-45.4% (-58.3 to -28.7)
Jordan	14.75 (12.52 to 17.05)	4.9% (-1.7 to 11.5)	0.56 (0.47 to 0.69)	-32.1% (-45.4 to -5.2)	1.31 (0.86 to 1.84)	4.9% (-5.3 to 16.3)	12.61 (10.80 to 15.07)	-55.5% (-65.3 to -32.1)
Kuwait	26.68 (22.83 to 31.08)	2.2% (-3.8 to 8.9)	0.66 (0.57 to 0.85)	-43.9% (-53.9 to -16.5)	2.28 (1.49 to 3.26)	2.0% (-12.6 to 18.6)	17.78 (14.84 to 23.95)	-52.8% (-62.6 to -21.8)
Lebanon	36.94 (31.78 to 43.01)	1.5% (-4.4 to 8.0)	1.60 (1.26 to 2.09)	-22.5% (-43.2 to 4.6)	3.14 (2.11 to 4.42)	1.1% (-12.2 to 16.0)	37.67 (29.65 to 49.39)	-34.8% (-50.8 to -11.7)
Libya	38.57 (32.98 to 44.61)	21.6% (13.8 to 29.7)	2.36 (1.70 to 3.06)	-34.9% (-53.7 to -6.5)	3.27 (2.20 to 4.54)	20.5% (4.4 to 38.5)	96.64 (67.09 to 133.29)	-54.2% (-69.7 to -28.3)
Morocco	27.52 (23.58 to 32.02)	17.5% (10.0 to 25.6)	1.83 (1.44 to 2.34)	-0.3% (-28.2 to 40.0)	2.36 (1.55 to 3.34)	16.8% (0.9 to 34.4)	51.46 (40.15 to 65.61)	-17.7% (-40.5 to 13.1)
Palestine	64.07 (54.97 to 74.28)	12.1% (5.5 to 19.2)	2.53 (2.26 to 2.87)	-32.1% (-47.9 to -4.0)	5.38 (3.64 to 7.51)	12.0% (-2.8 to 28.6)	66.20 (58.89 to 76.59)	-47.7% (-61.2 to -23.9)
Oman	46.41 (39.70 to 53.83)	33.5% (23.3 to 44.0)	2.04 (1.67 to 2.45)	41.1% (-1.9 to 95.1)	3.88 (2.62 to 5.45)	33.1% (12.9 to 55.6)	62.94 (52.01 to 76.17)	2.7% (-25.9 to 51.4)
Qatar	73.07 (62.51 to 84.49)	3.4% (-3.7 to 10.8)	1.94 (1.59 to 2.36)	-59.7% (-70.2 to -39.5)	6.14 (4.11 to 8.50)	4.2% (-9.1 to 19.4)	41.48 (33.47 to 51.09)	-67.4% (-76.4 to -44.1)
Saudi Arabia	96.50 (82.11 to 113.32)	3.6% (-3.8 to 11.4)	4.37 (3.55 to 5.63)	36.3% (-12.4 to 135.4)	8.07 (5.40 to 11.25)	4.4% (-7.2 to 17.4)	91.77 (73.35 to 118.86)	5.3% (-30.7 to 78.0)
Sudan	21.32 (18.20 to 24.74)	11.1% (3.6 to 19.2)	2.05 (1.52 to 2.74)	2.4% (-25.5 to 45.1)	1.84 (1.21 to 2.66)	11.5% (-3.1 to 28.9)	65.04 (49.32 to 84.61)	-10.8% (-38.0 to 32.6)
Syria	44.62 (38.31 to 52.08)	20.8% (13.2 to 28.4)	2.36 (1.90 to 2.92)	-13.0% (-32.8 to 19.7)	3.79 (2.53 to 5.33)	20.6% (5.1 to 38.5)	62.34 (48.81 to 77.88)	-36.2% (-53.0 to -14.1)
Tunisia	34.43 (29.39 to 39.84)	13.6% (6.2 to 21.9)	1.78 (1.33 to 2.32)	-11.3% (-37.4 to 33.5)	2.94 (1.96 to 4.14)	13.2% (-2.3 to 31.4)	39.18 (28.15 to 50.81)	-34.0% (-54.0 to -3.8)
Turkey	35.34 (30.93 to 40.08)	1.7% (-5.6 to 9.9)	0.99 (0.87 to 1.26)	-26.6% (-40.8 to -4.9)	3.08 (2.08 to 4.30)	2.1% (-13.3 to 20.7)	40.33 (34.74 to 47.52)	-28.4% (-46.0 to -3.2)
United Arab Emirates	48.80 (41.60 to 56.95)	-9.7% (-15.7 to -3.1)	3.05 (1.78 to 5.04)	20.7% (-32.3 to 82.6)	4.14 (2.80 to 5.85)	-8.9% (-21.3 to 5.8)	81.55 (51.43 to 129.04)	13.2% (-34.9 to 71.2)
Yemen	20.78 (17.75 to 24.43)	17.7% (10.2 to 25.2)	1.96 (1.41 to 2.69)	1.7% (-30.8 to 66.4)	1.78 (1.19 to 2.48)	17.8% (4.1 to 33.2)	58.93 (42.43 to 81.91)	-20.3% (-50.9 to 43.0)
Central Asia	50.98 (43.76 to 58.97)	-2.1% (-6.2 to 2.4)	6.40 (5.41 to 6.98)	153.3% (87.2 to 191.2)	4.28 (2.84 to 5.90)	-2.1% (-8.3 to 4.5)	165.36 (136.26 to 182.48)	130.8% (60.7 to 163.3)
Armenia	35.65 (30.61 to 41.10)	-3.6% (-9.6 to 2.5)	3.67 (2.92 to 4.05)	88.5% (44.1 to 226.6)	3.04 (2.03 to 4.30)	-3.3% (-14.6 to 11.1)	62.81 (55.34 to 71.18)	81.9% (46.1 to 145.5)
Azerbaijan	98.66 (84.53 to 116.13)	-3.9% (-10.9 to 3.0)	9.51 (8.01 to 12.31)	2.0% (-22.5 to 26.0)	8.21 (5.50 to 11.58)	-4.3% (-15.9 to 7.2)	207.73 (173.56 to 315.56)	-15.6% (-30.1 to 5.2)
Georgia	36.55 (31.29 to 42.03)	-1.8% (-8.2 to 5.6)	3.26 (2.65 to 4.33)	49.6% (11.5 to 90.9)	3.13 (2.06 to 4.42)	-2.1% (-15.0 to 13.1)	87.06 (67.05 to 121.83)	45.7% (5.9 to 87.0)
Kazakhstan	99.85 (84.70 to 116.54)	4.6% (-3.3 to 13.3)	17.00 (11.32 to 19.61)	1265.0% (397.4 to 1746.9)	8.29 (5.44 to 11.47)	5.0% (-7.0 to 18.0)	456.57 (281.66 to 552.50)	1112.8% (277.2 to 1605.1)
Kyrgyzstan	7.35 (6.20 to 8.66)	19.9% (12.5 to 28.2)	0.41 (0.33 to 0.47)	-6.5% (-25.4 to 11.5)	0.66 (0.43 to 0.94)	19.0% (6.8 to 32.4)	14.51 (10.41 to 17.31)	11.1% (-23.2 to 40.2)
Mongolia	63.24 (53.65 to 73.77)	18.1% (10.2 to 27.2)	5.86 (5.01 to 6.99)	1.9% (-17.2 to 27.9)	5.30 (3.51 to 7.39)	17.1% (1.5 to 35.5)	146.71 (124.95 to 187.20)	-1.9% (-19.4 to 20.7)
Tajikistan	8.50 (7.18 to 9.85)	-0.9% (-7.4 to 5.5)	0.66 (0.59 to 0.74)	22.4% (4.8 to 43.8)	0.77 (0.51 to 1.08)	-1.0% (-8.6 to 7.1)	20.13 (17.67 to 23.20)	20.8% (3.4 to 43.1)
Turkmenistan	89.13 (76.09 to 103.98)	1.2% (-5.3 to 8.8)	7.52 (6.39 to 10.96)	-33.7% (-48.0 to 13.2)	7.51 (4.95 to 10.68)	1.5% (-10.0 to 14.6)	264.29 (225.82 to 380.18)	-28.8% (-43.6 to 21.5)
Uzbekistan	12.73 (10.77 to 14.59)	8.6% (1.7 to 15.4)	1.03 (0.86 to 1.42)	133.7% (66.2 to 323.9)	1.16 (0.76 to 1.63)	8.4% (0.5 to 17.0)	35.32 (29.70 to 43.21)	142.9% (59.3 to 292.3)
South Asia	33.95 (28.75 to 39.71)	6.7% (4.4 to 8.7)	2.95 (2.24 to 3.79)	20.1% (1.8 to 55.7)	2.86 (1.94 to 4.03)	7.1% (3.3 to 10.8)	69.26 (52.92 to 89.81)	8.4% (-9.3 to 35.6)
Bangladesh	44.66 (38.00 to 52.08)	11.7% (4.1 to 19.9)	2.25 (1.58 to 3.34)	-1.9% (-20.0 to 25.5)	3.76 (2.50 to 5.30)	12.0% (-2.8 to 29.0)	55.10 (37.03 to 83.14)	-15.6% (-32.8 to 7.6)

Bhutan	48.05 (40.79 to 56.53)	14.3% (7.0 to 22.9)	2.84 (1.96 to 3.87)	-2.9% (-29.9 to 30.3)	4.05 (2.71 to 5.67)	14.6% (0.6 to 31.9)	63.79 (43.34 to 88.44)	-17.7% (-42.8 to 12.8)
India	31.03 (26.10 to 36.62)	10.0% (8.2 to 11.8)	2.90 (2.15 to 3.71)	23.9% (3.7 to 61.4)	2.62 (1.76 to 3.68)	10.2% (6.8 to 13.6)	68.06 (51.18 to 87.67)	9.9% (-8.9 to 38.2)
Nepal	32.07 (27.25 to 37.42)	12.0% (4.5 to 19.4)	1.93 (1.24 to 2.73)	25.7% (-8.4 to 76.0)	2.71 (1.79 to 3.84)	12.5% (-2.4 to 30.1)	43.52 (27.84 to 63.20)	5.3% (-25.0 to 48.0)
Pakistan	51.30 (43.29 to 59.74)	6.1% (-1.6 to 13.5)	4.39 (3.02 to 6.10)	29.7% (-1.9 to 72.0)	4.27 (2.89 to 6.12)	6.5% (-6.7 to 22.0)	99.27 (68.19 to 138.06)	20.1% (-8.6 to 59.9)
Southeast Asia	32.77 (27.98 to 38.14)	15.5% (12.7 to 18.6)	2.76 (2.47 to 3.06)	22.9% (8.7 to 45.0)	2.77 (1.87 to 3.87)	15.6% (10.8 to 20.5)	60.32 (53.91 to 66.97)	8.3% (-7.4 to 26.0)
Cambodia	24.69 (20.84 to 29.45)	9.9% (2.0 to 17.8)	2.57 (2.09 to 3.19)	19.5% (-12.6 to 69.0)	2.05 (1.35 to 3.00)	10.2% (-6.6 to 27.5)	56.63 (46.25 to 68.70)	0.1% (-33.0 to 41.9)
Indonesia	33.95 (28.28 to 40.27)	23.0% (19.4 to 26.3)	2.73 (2.23 to 3.38)	38.7% (20.6 to 62.6)	2.85 (1.90 to 3.97)	22.4% (16.3 to 29.1)	56.30 (45.27 to 69.83)	11.2% (-2.4 to 32.2)
Laos	23.00 (19.53 to 26.97)	16.0% (7.9 to 24.2)	3.22 (2.55 to 3.96)	9.8% (-18.1 to 44.4)	1.95 (1.28 to 2.77)	15.5% (0.7 to 31.6)	84.30 (67.24 to 102.06)	-13.7% (-39.8 to 21.1)
Malaysia	55.94 (47.69 to 64.76)	13.2% (6.1 to 20.1)	2.51 (2.19 to 2.93)	4.8% (-12.3 to 28.7)	4.79 (3.21 to 6.70)	12.9% (-1.0 to 28.6)	73.52 (63.64 to 84.37)	-2.0% (-17.4 to 16.6)
Maldives	18.95 (16.05 to 22.24)	40.5% (31.1 to 51.0)	1.33 (1.13 to 1.54)	-23.6% (-48.4 to 10.5)	1.62 (1.05 to 2.30)	38.8% (22.2 to 55.7)	20.17 (17.63 to 22.72)	-51.7% (-67.9 to -25.6)
Mauritius	29.01 (24.76 to 33.56)	0.5% (-5.8 to 7.2)	2.93 (2.22 to 3.31)	141.5% (34.2 to 197.5)	2.48 (1.67 to 3.44)	0.2% (-11.1 to 13.1)	81.29 (53.39 to 94.12)	148.6% (25.3 to 212.3)
Myanmar	17.62 (14.97 to 20.75)	10.8% (3.5 to 18.8)	2.19 (1.76 to 2.75)	10.1% (-24.3 to 52.8)	1.51 (0.99 to 2.18)	11.0% (-3.7 to 28.6)	57.55 (45.73 to 70.59)	-9.9% (-45.5 to 38.8)
Philippines	30.80 (25.46 to 36.84)	7.0% (-1.2 to 16.1)	7.79 (6.07 to 8.89)	254.4% (123.2 to 352.6)	2.54 (1.69 to 3.62)	6.8% (-4.7 to 21.3)	112.88 (94.97 to 127.22)	174.4% (58.9 to 242.7)
Sri Lanka	120.75 (102.52 to 141.86)	26.9% (17.6 to 37.3)	5.47 (4.42 to 6.98)	-53.0% (-64.8 to -30.1)	10.08 (6.75 to 14.20)	26.8% (13.5 to 41.8)	133.71 (106.78 to 168.12)	-49.8% (-62.3 to -27.7)
Seychelles	35.41 (29.51 to 42.38)	-8.0% (-14.5 to -1.4)	6.90 (6.08 to 7.79)	14.8% (-11.6 to 46.1)	2.97 (1.99 to 4.13)	-7.9% (-17.6 to 2.7)	127.69 (113.98 to 142.30)	12.4% (-12.7 to 42.1)
Thailand	13.61 (11.52 to 15.98)	12.7% (5.0 to 20.8)	0.47 (0.41 to 0.54)	4.2% (-27.1 to 39.2)	1.18 (0.78 to 1.69)	12.6% (-3.7 to 30.2)	14.36 (11.40 to 16.82)	28.0% (-23.6 to 86.7)
Timor-Leste	29.79 (25.17 to 35.51)	24.3% (15.9 to 33.5)	2.96 (1.83 to 4.54)	23.9% (-9.8 to 65.2)	2.48 (1.66 to 3.57)	24.3% (6.5 to 46.2)	66.52 (40.08 to 104.59)	-7.4% (-39.1 to 39.4)
Vietnam	32.34 (27.67 to 37.68)	10.2% (2.2 to 19.1)	2.52 (1.81 to 3.14)	21.5% (-13.7 to 76.8)	2.75 (1.84 to 3.90)	10.7% (-4.4 to 29.5)	53.31 (37.06 to 68.00)	6.8% (-28.6 to 53.3)
East Asia	15.47 (13.10 to 18.12)	21.2% (19.0 to 23.5)	0.81 (0.73 to 1.00)	27.0% (7.6 to 56.9)	1.37 (0.92 to 1.92)	20.2% (16.6 to 24.1)	23.83 (21.44 to 29.12)	-10.1% (-24.5 to 10.3)
China	14.79 (12.43 to 17.44)	23.9% (21.6 to 26.1)	0.80 (0.72 to 0.99)	30.7% (9.3 to 62.5)	1.32 (0.88 to 1.85)	22.7% (19.0 to 26.6)	23.31 (20.85 to 28.73)	-10.6% (-25.6 to 10.2)
North Korea	17.45 (14.79 to 20.20)	-1.3% (-7.4 to 5.6)	1.28 (1.05 to 1.56)	25.4% (-0.2 to 55.3)	1.54 (1.01 to 2.20)	-1.5% (-16.8 to 15.7)	48.84 (38.86 to 59.74)	32.9% (2.8 to 73.2)
Taiwan (Province of China)	49.35 (44.35 to 55.20)	-4.4% (-15.4 to 8.4)	1.03 (0.90 to 1.31)	-35.1% (-45.9 to -6.6)	4.23 (2.90 to 5.87)	-4.5% (-19.8 to 16.0)	27.24 (23.75 to 33.24)	-25.3% (-36.9 to -2.5)
Oceania	23.43 (20.02 to 27.19)	2.7% (-0.7 to 6.1)	5.08 (4.11 to 6.32)	8.3% (-7.3 to 24.7)	1.98 (1.31 to 2.74)	2.3% (-4.1 to 9.1)	165.34 (132.51 to 208.02)	7.8% (-10.5 to 29.0)
American Samoa	96.39 (81.73 to 113.34)	3.7% (-3.1 to 11.4)	8.99 (7.88 to 10.27)	15.0% (-10.7 to 40.7)	7.98 (5.23 to 11.11)	3.3% (-6.9 to 14.8)	228.71 (203.62 to 257.16)	16.1% (-6.1 to 37.9)
Federated States of Micronesia	35.54 (29.98 to 41.71)	16.0% (7.9 to 24.6)	5.45 (4.15 to 6.82)	5.7% (-17.6 to 34.0)	2.95 (1.95 to 4.21)	14.5% (0.8 to 30.7)	150.85 (105.21 to 197.11)	-2.3% (-31.2 to 27.1)
Fiji	31.06 (26.13 to 36.70)	3.1% (-4.0 to 10.3)	5.32 (4.53 to 6.16)	8.4% (-13.1 to 34.2)	2.60 (1.71 to 3.64)	2.8% (-11.1 to 17.5)	153.68 (131.81 to 177.96)	11.1% (-10.5 to 35.2)
Guam	47.18 (40.25 to 54.77)	2.9% (-3.5 to 9.5)	3.91 (3.45 to 4.40)	-4.1% (-20.8 to 17.5)	3.98 (2.67 to 5.58)	2.1% (-10.3 to 16.7)	122.39 (106.97 to 138.78)	10.4% (-9.7 to 34.4)
Kiribati	19.91 (16.69 to 23.52)	1.2% (-5.6 to 9.1)	3.67 (1.90 to 4.81)	5.7% (-15.5 to 29.9)	1.67 (1.11 to 2.35)	0.7% (-13.4 to 17.1)	121.28 (57.90 to 159.06)	-0.6% (-23.2 to 24.5)
Marshall Islands	34.43 (29.14 to 40.29)	9.9% (1.9 to 17.9)	7.46 (5.61 to 10.25)	28.9% (7.3 to 58.0)	2.87 (1.91 to 4.01)	8.9% (-4.5 to 24.1)	212.51 (160.79 to 292.94)	31.3% (9.0 to 59.3)
Northern Mariana Islands	90.62 (77.19 to 105.39)	2.1% (-4.7 to 10.1)	4.32 (3.72 to 4.95)	-1.2% (-21.0 to 21.7)	7.58 (5.15 to 10.56)	1.5% (-8.8 to 14.1)	103.00 (89.51 to 117.57)	-3.6% (-22.9 to 19.1)
Papua New Guinea	17.08 (14.52 to 20.15)	11.2% (4.6 to 18.3)	5.07 (3.87 to 6.78)	9.3% (-11.0 to 35.0)	1.46 (0.96 to 2.06)	10.8% (-2.5 to 25.4)	174.15 (133.21 to 228.91)	4.4% (-17.8 to 32.3)
Samoa	44.72 (37.66 to 52.21)	9.5% (2.1 to 16.8)	4.74 (3.31 to 6.17)	12.1% (-9.8 to 38.9)	3.73 (2.49 to 5.24)	8.4% (-5.6 to 24.6)	115.22 (80.13 to 148.89)	-1.2% (-23.3 to 22.3)
Solomon Islands	21.93 (18.45 to 25.65)	6.5% (-1.0 to 14.2)	3.28 (2.15 to 4.34)	13.5% (-10.2 to 44.8)	1.85 (1.23 to 2.63)	5.4% (-9.4 to 20.6)	86.69 (57.79 to 115.74)	6.9% (-14.5 to 37.0)
Tonga	20.15 (17.17 to 23.36)	7.4% (0.2 to 15.2)	1.44 (1.21 to 1.74)	8.5% (-15.0 to 36.5)	1.73 (1.16 to 2.45)	6.5% (-7.3 to 23.1)	40.46 (34.07 to 48.53)	19.6% (-4.5 to 50.2)
Vanuatu	28.26 (23.85 to 33.30)	3.2% (-4.3 to 11.1)	5.51 (2.92 to 8.94)	24.3% (-4.9 to 62.6)	2.38 (1.59 to 3.43)	3.1% (-10.6 to 19.4)	160.75 (87.90 to 262.79)	22.2% (-9.9 to 68.1)
High-income Asia Pacific	101.54 (88.69 to 115.02)	-17.0% (-21.4 to -12.4)	1.46 (1.24 to 1.60)	-44.9% (-55.9 to -38.0)	8.68 (5.90 to 11.93)	-16.9% (-21.7 to -11.3)	34.30 (29.45 to 39.71)	-45.9% (-56.6 to -36.9)
Brunei	110.37 (94.58 to 128.02)	-21.0% (-25.9 to -15.4)	6.03 (5.39 to 6.81)	0.6% (-14.5 to 18.1)	9.34 (6.25 to 13.01)	-20.7% (-29.2 to -12.1)	167.11 (149.89 to 188.60)	-2.8% (-16.5 to 12.0)
Japan	115.39 (100.89 to 130.72)	-12.9% (-18.2 to -7.3)	1.57 (1.33 to 1.72)	-44.6% (-55.6 to -37.8)	9.88 (6.73 to 13.60)	-12.8% (-18.2 to -6.6)	37.65 (31.78 to 42.79)	-44.6% (-55.0 to -35.4)
Singapore	77.52 (66.61 to 89.73)	-5.5% (-11.8 to 1.2)	1.39 (1.04 to 2.06)	-47.3% (-64.2 to -17.3)	6.66 (4.41 to 9.21)	-4.9% (-16.8 to 8.5)	40.77 (33.12 to 57.46)	-52.4% (-70.2 to -28.9)
South Korea	64.96 (55.21 to 75.85)	-15.6% (-21.3 to -9.2)	1.05 (0.91 to 1.19)	-25.2% (-46.8 to 0.8)	5.56 (3.71 to 7.88)	-15.1% (-25.9 to -2.1)	24.15 (21.11 to 28.67)	-41.5% (-59.2 to -20.1)
High-income North America	100.96 (88.02 to 115.25)	-10.4% (-17.7 to -1.6)	4.02 (3.76 to 4.76)	-23.4% (-31.7 to 7.0)	8.39 (5.76 to 11.43)	-11.1% (-18.4 to -2.2)	97.18 (89.77 to 118.37)	-26.7% (-35.7 to 4.6)
Canada	88.02 (75.09 to 101.45)	-7.1% (-13.8 to -0.3)	1.40 (1.20 to 1.99)	-38.1% (-48.4 to -8.7)	7.45 (4.95 to 10.33)	-7.3% (-18.0 to 4.5)	35.40 (30.59 to 47.04)	-40.1% (-50.3 to -10.3)
Greenland	138.82 (114.98 to 164.15)	-2.2% (-10.1 to 7.0)	8.20 (7.25 to 9.27)	-19.9% (-31.8 to -3.5)	11.57 (7.71 to 16.63)	-1.4% (-12.3 to 10.5)	183.99 (161.34 to 209.58)	-35.1% (-45.6 to -21.7)
United States	102.60 (89.22 to 117.42)	-10.3% (-18.3 to -0.6)	4.34 (4.06 to 5.11)	-21.8% (-30.3 to 8.7)	8.51 (5.83 to 11.61)	-11.1% (-19.0 to -0.9)	104.35 (96.29 to 126.17)	-25.6% (-34.8 to 6.1)
Western Europe	91.04 (78.80 to 105.14)	-25.1% (-28.7 to -21.5)	2.87 (2.64 to 3.13)	-63.9% (-67.6 to -47.8)	7.63 (5.20 to 10.58)	-24.6% (-28.5 to -20.2)	46.46 (43.47 to 55.68)	-59.8% (-63.6 to -41.6)
Andorra	73.15 (62.91 to 85.14)	-30.8% (-35.2 to -26.4)	2.07 (1.49 to 2.65)	-26.4% (-42.5 to -3.3)	6.17 (4.16 to 8.54)	-30.4% (-38.2 to -22.5)	44.87 (32.41 to 58.97)	-37.4% (-52.2 to -15.3)
Austria	166.75 (142.83 to 191.70)	-16.5% (-25.0 to -6.7)	6.09 (4.92 to 6.78)	-82.2% (-84.3 to -76.3)	13.73 (9.27 to 18.93)	-16.4% (-26.1 to -5.7)	65.06 (57.50 to 81.29)	-82.6% (-85.6 to -70.3)
Belgium	88.57 (75.26 to 102.30)	-22.5% (-29.3 to -14.0)	2.95 (2.47 to 3.35)	-50.3% (-58.8 to -29.5)	7.32 (4.94 to 10.32)	-22.2% (-31.0 to -10.5)	37.76 (33.38 to 46.56)	-57.7% (-64.8 to -34.7)
Cyprus	77.02 (66.55 to 89.23)	-21.5% (-27.1 to -14.5)	2.42 (2.09 to 2.85)	-26.2% (-37.4 to -11.9)	6.56 (4.43 to 9.24)	-21.2% (-30.5 to -10.8)	56.05 (47.67 to 63.33)	-30.3% (-40.6 to -18.1)
Denmark	42.79 (36.94 to 49.38)	-27.2% (-31.5 to -22.8)	1.32 (1.17 to 1.55)	-8.0% (-26.8 to 12.5)	3.66 (2.46 to 5.14)	-27.3% (-38.3 to -15.4)	29.17 (25.71 to 33.06)	-24.2% (-40.9 to -7.2)

Finland	57.31 (47.90 to 69.12)	-21.0% (-28.4 to -10.0)	2.15 (1.60 to 2.47)	17.8% (-22.5 to 55.2)	4.81 (3.15 to 6.84)	-20.8% (-31.1 to -7.1)	47.34 (38.09 to 53.44)	-1.8% (-33.5 to 32.7)
France	99.11 (83.89 to 117.04)	-30.7% (-35.3 to -25.5)	2.79 (2.51 to 3.10)	-50.0% (-56.6 to -35.4)	8.32 (5.54 to 11.77)	-30.1% (-37.4 to -22.2)	43.32 (38.33 to 57.75)	-52.3% (-58.5 to -36.6)
Germany	51.91 (44.02 to 60.96)	-38.7% (-43.5 to -33.8)	2.86 (2.25 to 3.31)	-34.1% (-46.1 to -9.0)	4.37 (2.95 to 6.15)	-37.8% (-45.4 to -30.0)	43.79 (38.31 to 49.91)	-39.7% (-50.8 to -17.8)
Greece	88.58 (74.91 to 103.51)	-27.9% (-33.0 to -22.8)	3.16 (2.51 to 3.54)	-38.7% (-46.9 to -29.4)	7.45 (4.83 to 10.48)	-27.5% (-35.7 to -19.1)	59.09 (48.27 to 65.27)	-28.3% (-39.1 to -17.0)
Iceland	42.99 (36.57 to 50.16)	-22.9% (-28.5 to -16.1)	0.97 (0.75 to 1.12)	-57.4% (-65.5 to -23.4)	3.64 (2.44 to 5.07)	-22.6% (-31.1 to -12.5)	15.04 (13.24 to 19.44)	-60.1% (-67.9 to -19.6)
Ireland	87.61 (73.83 to 103.27)	-22.7% (-28.2 to -17.4)	2.49 (2.02 to 3.29)	-79.3% (-84.4 to -58.0)	7.30 (4.96 to 10.20)	-22.4% (-30.2 to -13.5)	37.40 (29.42 to 61.75)	-76.8% (-82.7 to -48.9)
Israel	70.55 (60.04 to 82.52)	-31.3% (-35.9 to -26.5)	1.41 (1.23 to 1.61)	-46.7% (-54.3 to -25.4)	5.96 (4.02 to 8.48)	-30.6% (-38.3 to -21.9)	28.90 (25.32 to 37.06)	-54.8% (-62.2 to -33.3)
Italy	186.01 (158.36 to 215.77)	-17.3% (-26.9 to -7.4)	3.24 (2.75 to 4.02)	-86.4% (-89.4 to -74.2)	15.50 (10.57 to 21.37)	-16.9% (-27.7 to -5.6)	45.24 (38.50 to 69.54)	-83.6% (-86.8 to -65.0)
Luxembourg	74.33 (63.06 to 87.58)	-21.4% (-28.5 to -11.8)	2.15 (1.82 to 2.63)	-64.1% (-72.1 to -17.5)	6.17 (4.16 to 8.76)	-20.6% (-29.8 to -8.7)	28.09 (22.62 to 50.08)	-69.7% (-77.3 to -9.7)
Malta	95.49 (80.69 to 111.05)	-25.1% (-31.5 to -17.5)	2.67 (2.26 to 3.29)	-77.9% (-82.6 to -56.9)	7.95 (5.36 to 11.10)	-24.8% (-33.5 to -14.0)	43.75 (36.19 to 68.71)	-74.3% (-80.3 to -44.8)
Netherlands	56.75 (48.10 to 66.42)	-22.6% (-29.1 to -15.6)	2.14 (1.87 to 2.59)	-67.0% (-72.8 to -42.6)	4.77 (3.18 to 6.71)	-21.9% (-31.4 to -11.0)	31.61 (27.06 to 46.41)	-65.0% (-71.5 to -34.1)
Norway	50.52 (44.32 to 57.18)	-28.7% (-35.3 to -20.7)	1.58 (1.47 to 1.74)	0.5% (-14.3 to 9.6)	4.28 (2.95 to 5.86)	-28.4% (-35.5 to -20.6)	31.47 (28.38 to 34.71)	-21.2% (-34.3 to -13.2)
Portugal	50.34 (43.15 to 58.55)	-34.2% (-39.4 to -28.8)	2.08 (1.75 to 2.34)	-54.6% (-61.4 to -44.1)	4.24 (2.87 to 5.92)	-33.2% (-40.8 to -24.1)	33.78 (29.42 to 38.43)	-57.3% (-62.9 to -46.5)
Spain	99.87 (84.28 to 117.72)	-31.3% (-35.9 to -26.2)	4.07 (3.46 to 4.56)	-49.7% (-56.2 to -38.8)	8.43 (5.57 to 11.70)	-30.8% (-37.9 to -23.2)	70.28 (62.60 to 80.34)	-42.9% (-50.4 to -33.7)
Sweden	84.36 (71.30 to 99.18)	-26.3% (-30.6 to -21.7)	1.86 (1.68 to 2.17)	-49.1% (-58.5 to -16.5)	7.17 (4.83 to 10.01)	-25.9% (-32.7 to -17.9)	38.25 (33.89 to 46.89)	-59.2% (-67.3 to -30.4)
Switzerland	56.64 (49.52 to 64.45)	-28.8% (-35.5 to -20.9)	1.74 (1.54 to 1.93)	-35.9% (-46.0 to -16.7)	4.77 (3.28 to 6.60)	-28.0% (-37.3 to -17.7)	28.04 (24.82 to 32.99)	-44.4% (-55.8 to -24.8)
United Kingdom	51.25 (44.64 to 58.57)	-26.6% (-30.4 to -23.0)	2.02 (1.89 to 2.36)	-12.3% (-19.8 to 4.9)	4.36 (2.96 to 5.96)	-26.3% (-30.0 to -22.5)	45.25 (42.46 to 54.15)	-29.0% (-34.7 to -11.9)
Australasia	80.54 (68.95 to 93.54)	-21.3% (-26.0 to -16.4)	2.06 (1.80 to 2.84)	-57.4% (-63.9 to -34.3)	6.75 (4.52 to 9.55)	-20.8% (-28.6 to -12.7)	45.63 (39.28 to 66.56)	-55.6% (-62.9 to -29.5)
Australia	81.10 (69.05 to 94.57)	-22.2% (-27.7 to -16.8)	2.00 (1.72 to 2.75)	-60.1% (-67.1 to -37.5)	6.80 (4.53 to 9.66)	-21.6% (-30.5 to -12.6)	42.20 (35.19 to 63.30)	-58.2% (-66.2 to -30.9)
New Zealand	77.37 (66.41 to 90.30)	-17.0% (-23.9 to -9.9)	2.37 (2.09 to 3.41)	-41.3% (-49.8 to -14.0)	6.47 (4.39 to 8.95)	-17.2% (-27.4 to -5.4)	64.03 (57.27 to 85.33)	-43.2% (-50.9 to -19.5)

Data in parentheses are 95% uncertainty intervals. YLDs= years lived with disability; YLLs= years of life lost.
